# Numerous Transitions of Sex Chromosomes in Diptera

**DOI:** 10.1371/journal.pbio.1002078

**Published:** 2015-04-16

**Authors:** Beatriz Vicoso, Doris Bachtrog

**Affiliations:** Department of Integrative Biology, University of California Berkeley, Berkeley, California, United States of America; Fred Hutchinson Cancer Research Center, UNITED STATES

## Abstract

Many species groups, including mammals and many insects, determine sex using heteromorphic sex chromosomes. Diptera flies, which include the model *Drosophila melanogaster*, generally have XY sex chromosomes and a conserved karyotype consisting of six chromosomal arms (five large rods and a small dot), but superficially similar karyotypes may conceal the true extent of sex chromosome variation. Here, we use whole-genome analysis in 37 fly species belonging to 22 different families of Diptera and uncover tremendous hidden diversity in sex chromosome karyotypes among flies. We identify over a dozen different sex chromosome configurations, and the small dot chromosome is repeatedly used as the sex chromosome, which presumably reflects the ancestral karyotype of higher Diptera. However, we identify species with undifferentiated sex chromosomes, others in which a different chromosome replaced the dot as a sex chromosome or in which up to three chromosomal elements became incorporated into the sex chromosomes, and others yet with female heterogamety (ZW sex chromosomes). Transcriptome analysis shows that dosage compensation has evolved multiple times in flies, consistently through up-regulation of the single X in males. However, X chromosomes generally show a deficiency of genes with male-biased expression, possibly reflecting sex-specific selective pressures. These species thus provide a rich resource to study sex chromosome biology in a comparative manner and show that similar selective forces have shaped the unique evolution of sex chromosomes in diverse fly taxa.

## Abstract 2

Analysis of the genomes of 37 fly species from 22 families of Diptera shows that superficially similar karyotypes conceal the true extent of sex chromosome variation and that sex chromosome transitions are, in fact, frequent in flies.

## Author Summary

A mind-blowing diversity of sex-determining mechanisms exists among eukaryotes, but highly differentiated sex chromosomes—a degenerate, gene-poor Y chromosome, and an often dosage-compensated X—appear to represent an evolutionary dead-end. In our manuscript, we systematically study the genomic composition of sex chromosomes across dipteran insects (flies and mosquitoes), which are generally considered to show stable XY sex chromosomes. Our whole-genome analysis of 37 fly species from 22 families of Diptera uncovers tremendous hidden variation in sex chromosomes. Some species have newly gained or secondarily lost their sex chromosomes; in others, a different chromosome has replaced the original sex chromosome or multiple chromosomal elements have become incorporated into the sex chromosomes; still other species have female heterogametic sex chromosomes. We perform a comparative transcriptome analysis to show that dosage compensation has evolved multiple times, consistently through up-regulation of the single X chromosome in males. These species provide a rich resource to study sex chromosome biology in a comparative manner and show that similar selective forces have shaped the unique evolution of sex chromosomes in diverse fly taxa.

## Introduction

Sex is a universal feature of eukaryotic organisms, yet tremendous variation in sex determination mechanisms exists even among closely related species, or among individuals within a species [1,2]. In several taxa where sex is established at fertilization by genetic factors, such as in many vertebrates and insects, an entire chromosome co-segregates with the sex determining genes (sex chromosomes). Sex chromosomes evolved from ordinary autosomes that acquired a sex-determining function and stopped recombining. Over evolutionary time, sex chromosomes differentiate, and the sex-limited chromosome—which is entirely sheltered from recombination—degenerates [1,2]. The evolutionary forces driving this diversity of sex determination mechanisms are little understood [1,3], and most of our knowledge of sex determination and sex chromosome biology comes from a few model species, including the Diptera fly *Drosophila melanogaster*.

*Drosophila* species have six chromosomal pairs (five large rods and a small dot chromosome), and extensive cytogenetic studies suggest that this chromosomal complement is well conserved and represents the ancestral karyotype in Diptera [4,5]. The gene content of these chromosomal elements, which are commonly referred to as Muller elements A–F (where F is the small dot chromosome) [6], is highly conserved across Diptera [7]. Despite their similar chromosomal architecture and genotypic sex determination in (almost) all flies, however, comparative studies have shown that both the master switch genes responsible for sex determination, as well as the presence and nature of sex chromosomes, varies among groups [4,8].

Diptera flies are generally divided into the paraphyletic lower Diptera (Nematocera) and monophyletic higher Diptera (Brachycera), and a classification based on cytological grounds suggested dividing lower Diptera into four groups [4]. The primitive superfamily Tipuloidea (i.e., crane flies, group I) is characterized by the presence of chiasmata in both sexes and cytologically distinguishable sex chromosomes. A second assemblage of families (group II, which includes mosquitoes, black flies, and sand flies) generally lack cytologically distinguishable sex chromosomes but have retained chiasmata in males. Group III, which includes dung midges, is characterized by heteromorphic sex chromosomes but a lack of chiasmata in males. A fourth cytological group of Nematocera, which contains gall midges, is characterized by a highly specialized chromosome cycle, the complete loss of the Y chromosome, and sex is determined by X chromosome elimination (i.e., all zygotes start with the same number of X chromosomes, and embryos developing into males eliminate their X[s] to become X0). Most families of Diptera, including Drosophilidae, fall within the suborder Brachycera (higher Diptera). All Brachycera appear to lack chiasmata in males, and most species have heteromorphic XY sex chromosomes. How these classifications based on cytology hold up at the molecular level and whether sex chromosomes of different cytogenetic groups are homologous, however, is unclear.

Sex in the *Drosophila* genus is determined by a relatively large X chromosome (element A) that is homologous to the independently evolved X of the distantly related mosquito *Anopheles gambiae* [7,9]. We recently showed that the karyotype of *Drosophila* is in fact derived, and in several outgroup Brachycera species, the small dot chromosome (element F) instead segregates as an X chromosome [10], illustrating the potentially transient nature of sex chromosomes in flies. Here, we greatly expand our phylogenetic sampling to broadly investigate transitions of sex chromosomes in both Nematocera and Brachycera dipterans (Table 1), with members from each major cytological group and a focus on families in which cytological evidence suggested that a transition might have taken place [4,5]. This is combined with a transcriptome analysis in a subset of species to study general features of sex chromosome biology, including the nature and evolutionary dynamics of sex chromosomes, the identification of the general mechanisms of dosage compensation, and sex-specific selection shaping gene content and gene expression evolution of sex chromosomes.

**Table 1:** Overview of groups sequenced.

Species	Family	Genome size	scaffolds>1 kb	N50	sex chromosome
**Nematocera Group I**					
*Tipula oleracea*	Tipulidea	793,197,011	186,880	2,229	dot
Trichoceridae sp.	Trichoceridae	112,595,433	26,751	662	dot
**Nematocera Group II**					
*Chironomus riparius*	Chironomidae	214,074,568	29,688	7,361	undifferentiated
*Chaoborus trivittatus*	Chaoboridae	509,827,186	80,766	2,357	undifferentiated
*Mochlonyx cinctipes*	Culicidae	545,067,436	95,135	6,397	undifferentiated
*Anopheles gambiae*	Culicidae	310,885,381	54,390	3,462	A
*Aedes aegypti*	Culicidae	998,314,895	223,105	3,312	undifferentiated
*Clogmia albipunctata*	Psychodidae	333,829,385	42,895	13,421	undifferentiated
**Nematocera Group III**					
*Coboldia fuscipes*	Scatopsidae	111,584,708	1,745	256,895	dot
**Nematocera Group IV**					
*Mayetiola destructor*	Cecidomyiidae	194,206,451	28,227	6,110	C,D,F
**Brachycera**					
*Hermetia illucens*	Stratiomyidae	1,343,286,617	319,229	2,778	dot
*Megaselia abdita*	Phoridae	574,581,457	121,746	4,725	undifferentiated
*Eristalis dimidiata*	Syrphidae	549,568,617	146,256	958	dot
*Holcocephala fusca*	Asioidea	673,865,293	133,652	4591	dot + B(partial)
*Condylostylus patibulatus*	Dolichopodidae	924,769,508	211,365	1,010	dot
*Bactrocera oleae*	Tephritidae	456,712,509	68,784	5,992	dot
*Tephritis californica*	Tephritidae	755,870,990	192,574	992	dot
*Eutreta diana*	Tephritidae	579,715,143	140,937	807	dot
*Trupanea jonesi*	Tephritidae	313,250,229	75,893	863	dot
*Ephydra hians*	Ephydridae	655,322,950	143,196	1,317	D
*Ephydra gracilis*	Ephydridae	466,899,679	61,533	10,091	D
*Scaptodrosophila lebanonensis(female-only assembly)*	Drosophilidae	214,547,812	14,694	34,356	A+dot
*Drosophila busckii(female-only assembly)*	Drosophilidae	121,101,360	9,640	36,315	A+dot
*Drosophila pseudoobscura*	Drosophilidae	190,559,548	5,004	29,240	A+D
*Drosophila miranda*	Drosophilidae	150,263,482	40,080	11,990	A+C+D
*Drosophila melanogaster*	Drosophilidae	132,143,695	36,617	10,032	A
*Drosophila albomicans*	Drosophilidae	190,867,288	37,078	3,607	A+C+D
*Phortica variegata*	Drosophilidae	183,294,633	19,486	15,396	A
*Teleopsis dalmanni*	Diopsidae	810,849,452	174,724	2,082	B
*Sphyracephala brevicornis*	Diopsidae	544,561,571	135,817	1,259	B
*Themira minor*	Sepsidae	180,267,975	33,581	1,590	D
*Liriomyza trifolii*	Agromyzidae	153,286,579	31,313	947	D
*Sarcophaga bullata*	Sarcophagidae	752,125,458	184,355	1359	dot
Sarcophagidae *sp*.	Sarcophagidae	1,254,254,254	287,426	918	dot
*Glossina morsitans*	Glossinidae	402,913,301	34,347	19,738	A, D, F
*Lucilia sericata*	Calliphoridae	403,051,418	92,090	3,604	dot
*Calliphora erythrocephala*	Calliphoridae	792,453,887	197,518	1,548	undifferentiated

## Results and Discussion

### Identification of Sex Chromosomes in Flies

**Fig 1:**
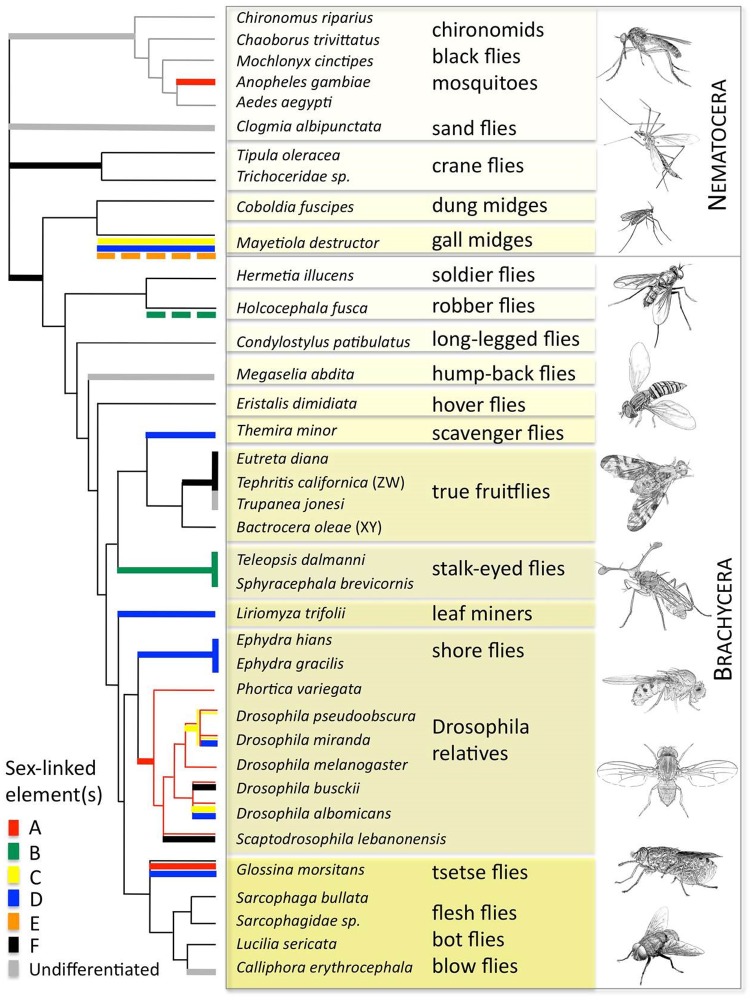
Sex chromosomes in Diptera, mapped along a fly phylogeny [13]. Different chromosomal elements (designated elements A–F) have become sex-linked in different families of Diptera (indicated by different colors along the phylogeny). Elements were classified as X-linked when they were over-represented among our candidate X scaffolds, based on their male/female coverage (see Materials and Methods and S1–S3 Figs.) or if they showed an excess of SNPs in the heterogametic sex (Figs. 2 and S5). Bold lines indicate a transition of a sex chromosome at a particular branch of the tree and was inferred using parsimony. Dashed lines indicate instances where partial Muller elements segregate as a sex chromosome (see Figs. 2 and S5, S6 and S1 Table for more details). Data to generate the phylogeny are to be found in file “S2 Text” and “S3 Text.” Images drawn by Doris Bachtrog.

We use whole-genome analysis in 37 species belonging to 22 different families of Diptera to infer the presence and identity of sex chromosomes (Table 1). While the identification of sex chromosomes traditionally involved the creation of labor-intensive genetic or physical maps, we have previously developed a bioinformatics approach to detect X-derived sequences based on their male and female genomic coverage using next-generation sequencing data [10,11]. We therefore independently sequenced males and females from each species to roughly a similar sequencing depth and used sequence coverage information to identify the absence or presence of differentiated sex chromosomes. In particular, when the Y is fully differentiated, X-linked genes show only half as many reads from genomic DNA in males, where they are present in one copy only, relative to females; autosomes or undifferentiated regions of the sex chromosomes have similar coverage in male and female samples (see Materials and Methods). Additionally, analysis of single nucleotide polymorphisms (SNPs) in males versus females was utilized to identify recently formed sex chromosomes where the X and Y still share considerable homology, through increased SNP density (early X-Y differentiation) at nascent sex chromosomes in the heteromorphic sex, relative to autosomal SNPs (see section “Young Sex Chromosomes”). It should be noted that sequences derived from differentiated Y-chromosomes are notoriously difficult to assemble and generally have much lower mapping scores to known genes than X-derived sequences [12], so that they are not found in our analysis. We further use comparative mapping (to either the *D. melanogaster* genome for Brachycera species, or to both the *D. melanogaster* and the *Anopheles gambiae* genomes for Nematocera; see Materials and Methods for details), to identify which Muller element(s) segregate(s) as sex chromosomes in each species (S1–S3 Figs.). Muller elements that had a significant excess of X-candidate scaffolds (based on sequence coverage) were classified as sex-linked (S3 Fig.). This was combined with SNP information to identify young sex chromosomes (see section “Young sex chromosomes”). Using this approach, we identify over a dozen different sex chromosome configurations in the species investigated (Fig. 1).

**Fig 2:**
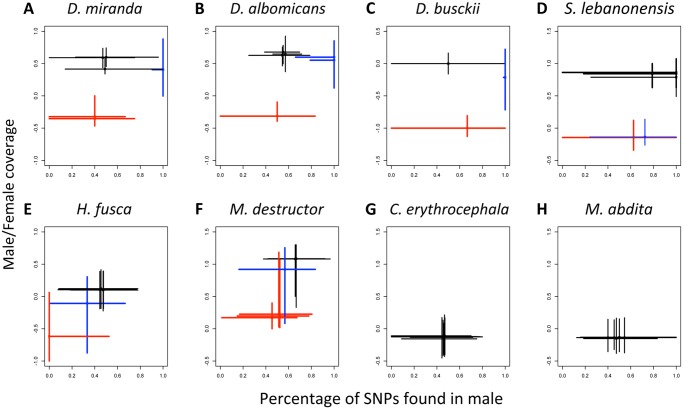
Detecting neo-sex chromosomes. **A,B,C**: Male/female coverage versus percentage of SNPs found in males (SNPs in the male sample/total number of SNPs) for scaffolds from the autosomes (black), fully differentiated (red), and newly evolved (blue) X chromosomes of *Drosophila miranda* (A), *Drosophila albomicans* (B), and *Drosophila busckii* (C). **D**: The neo-X (in blue) of *Scaptodrosophila lebanonensis* (D) is fully degenerated; it shows a 2-fold reduction in male versus female expression but no excess of male SNPs, similar to the ancestral X. **E,F**: The Muller elements of *Holcocephala fusca* (E) and *Mayetiola destructor* (F) that have intermediate levels of male to female coverage, in blue, have SNP patterns that are inconsistent with newly derived sex-chromosomes (no excess of male SNPs). **G,H**: No evidence of differentiated sex chromosomes using coverage or SNPs in *Calliphora erythrocephala* (G) and *Megaselia abdita* (H). The lines intersect at the median, and extend to the 10% and 90% percentiles. Data to generate this graph are to be found in file “S1 Data” and “S2 Data.”

A recent phylogenomic estimate of fly relationships suggests that crane flies (Tipulidae), mosquitoes (Culicidae, Chironimidae, Chaoboridae), and sand flies (Psychodidae) represent the earliest extant fly lineages, and dung midges (Scatopsidae) and gall midges (Cecidomyiidae) are the sister groups to all higher flies (Brachycera) [13], and we used this phylogeny to map sex chromosome karyotypes on the fly tree. We use parsimony to infer sex chromosome transitions along the tree (marked by bold lines along the phylogeney), based on coverage and SNP information. Many sex chromosome transitions appear to occur between families, but some also occur within families.

### A Variety of X-Linked Muller Elements

Within lower Diptera, we find that crane flies (Tipulidae) have differentiated sex chromosomes, with the dot chromosome (element F) segregating as the X. Mosquitoes (Culicidae, Chironimidae, Chaoboridae) and sand flies (Psychodidae) ancestrally lack differentiated sex chromosomes, but the genus *Anopheles* within Culicidae has acquired a new pair of sex chromosomes (corresponding to element A). In dung midges (Scatopsidae) and gall midges (Cecidomyiidae), it is also the dot that segregates as the sex chromosome, but gall midges have incorporated additional chromosomal elements into their sex chromosomes (elements C and D and part of element E; see section “Young Sex Chromosomes”). The phylogenetic relationships among lower Diptera families are not fully resolved (and our own attempt to use the genomic data to generate a tree led to low bootstrap support for these same nodes; see S4 Fig.), precluding us from reconstructing the ancestral sex chromosome complement of flies. Our data indicate that all Diptera either ancestrally had homomorphic sex chromosomes and the dot became a sex chromosome in crane flies and the ancestor of midges and higher Diptera, or that the dot was the ancestral sex chromosome but reverted to an autosomal inheritance in mosquitoes and sand flies.

Higher flies show a similar diversity of sex chromosome karyotypes. The dot segregates as the sex chromosome in Brachycera lineages that branched off early, such as soldier flies (Stratiomyidae) or long-legged flies (Dolichopodidae), and in the sister group of higher Diptera, suggesting that it is the ancestral sex chromosome of all higher Diptera. The dot chromosome is the sex chromosome in the majority of families investigated, and has remained the sex chromosome in several major fly lineages, including true fruit flies (Tephritidae), or the highly derived calyptrate flies, such as blowflies (Calliphoridae) or flesh flies (Sarcophagidae). This suggests that this chromosomal element has been sex-linked for over 200 million years (MY) of evolution in many higher fly families. Note that the gene content of the dot chromosome is remarkably well conserved throughout Diptera evolution, from the basal crane flies up to higher Diptera. This is analogous to “Ohno’s law” which refers to the finding that the gene content of the X differs little among placental mammals [14]. While almost all Brachycera flies have male heterogametic sex determination, a subgroup within true fruit flies (termed Tephritinae) has evolved female heterogamety. In this species group, it is also the dot that segregates as the sex chromosome, but females have only one Z chromosome, and males have two Z chromosomes (Figs. 1 and S1, S3).

In some higher fly families, additional chromosomal elements became incorporated into the ancestral sex chromosome. For example, our data show that the single large X chromosome of tsetse flies (Glossinidae) results from the incorporation of two additional chromosomal elements (elements A and D) to the ancestral X (element F). Similarly, in the robber fly *Holcocephala fusca*, part of Muller element B was added to the ancestral dot sex chromosome (Figs. 1 and S1, S3). In other families, a different chromosomal element has replaced the dot as the sex chromosome altogether. In particular, element D independently became the sex chromosome in several families, including shore flies (Ephydridae), black scavenger flies (Sepsidae), and leaf miners (Agromyzidae); element A is the sex chromosome in fruit flies (Drosophilidae), and element B in stalk-eyed flies (Diopsidae). Finally, other Brachycera species, such as the hump-backed fly *Megaselia abdita* (Phoridae), have completely lost their differentiated sex chromosomes. The closely related species *Megaselia scalaris* has three chromosome pairs, two metacentrics and an acrocentric (note that this species does not have a free dot chromosome). One of the metacentric chromosomes contains the male-determining gene but shows no signs of morphological differentiation (i.e., it is a homomorphic sex chromosome), but in some strains the male-determining locus can be located on either of the two autosomes [15,16]. This suggests that the XY pair in this species is at the earliest stages of evolution, and a single locus (or small region), rather than a chromosome or large chromosomal segment, is differentiated between the sexes [15,16], and a similar situation may be found in *M. abdita*. Similarly, we find that *Calliphora erythrocephala* has lost its differentiated sex chromosomes. Cytogenetic studies have shown that the karyotype in blowflies (Calliphoridae) is highly conserved, comprising of five large rods and a small dot. The dot chromosome segregates as the sex chromosome in most blowflies [17] and controls sexual development via a dominant male-determining factor on the Y [18]. In *C. erythrocephala*, the dot appears homomorphic in both sexes and has lost its sex-determining function, and sex determination was shown to have been taken over by one of the large, formerly autosomal chromosome pairs [18]. A young (and probably small) sex-determining locus is consistent with our genomic approach, which fails to detect differentiated sex chromosomes in *C. erythrocephala*.

While sex chromosome karyotypes are generally conserved within families, many species or species groups have derived sex chromosome configurations. As mentioned above, element A became a sex chromosome in the genus *Anopheles*, while other mosquitoes (family Culicidae) have undifferentiated sex chromosomes. Blowflies (Calliphoridae) have heteromorphic sex chromosomes (element F), including most species of the genus *Calliphora* [19], yet *C. erythrocephala* shows no evidence of having differentiated sex chromosomes. True fruit flies (Tephritids) have both male and female heterogametic sex determination, with the dot segregating as the sex chromosomes in both systems. In *Drosophila* fruit flies, element A is the ancestral sex chromosome, but several Drosophilidae have independently incorporated additional chromosomal elements into their ancestral sex chromosome (termed neo-sex chromosomes) at different time points during their evolution (Fig. 1 and Fig. 2; [20]). In particular, elements C and D became part of the sex chromosome in *D. albomicans* [21], element D became sex-linked in an ancestor of *D. miranda* and *D. pseudoobscura*, and element C became subsequently sex-linked in *D. miranda* only, while the dot chromosome secondarily became incorporated into the sex chromosome independently in both *D. busckii* and *Scaptodrosophila lebanonensis*.

### Young Sex Chromosomes

In some cases, these additions of former autosomes into the sex chromosomes are recent enough that substantial homology between the newly formed X and Y remains (i.e., element F in *D. busckii* or element C in *D. miranda*). In other cases, these fusions happened long enough ago, and no homology between those chromosomes can be detected (i.e., element F in *S. lebanonensis* or element D in *D. miranda*). In some species, newly incorporated sex chromosomes are so recent that the coverage method alone cannot detect them, since most Y-linked reads in males still map to the X (i.e., element C and D in *D. albomicans*, see S1 Fig.). Analysis of single nucleotide polymorphisms (SNPs) in males versus females can be informative to identify recently formed sex chromosomes, through increased SNP density (early X-Y differentiation) at nascent sex chromosomes in the heteromorphic sex relative to autosomes (Fig. 2), and can be used to reconstruct the evolutionary history of young sex chromosomes. For instance, Fig. 2 shows that the very recently formed (<0.1 MY) neo-sex chromosomes of *D. albomicans* show no coverage reduction but rather increased SNP density in males; the somewhat older (0.5–1 MY) and partly differentiated neo-sex chromosomes of *D. busckii* (element F) show somewhat reduced coverage and increased SNP density in males, similar to the younger neo-sex chromosome (element C, approximately 1.5 MY) of *D. miranda*; and the old (>10 MY) and highly differentiated neo-sex chromosomes in *D. miranda* and *D. pseudoobscura* (element D) and *S. lebanonensis* (element F) show no traces of remaining homology (Fig. 2).

We applied the same SNP approach to Brachycera species that appeared to have undifferentiated sex chromosomes based on sequence coverage level (*C. erythrocephala* and *M. abdita*), and to dipteran flies that showed intermediate levels of male to female (M/F) coverage for some Muller elements (element B in the robber fly *H. fusca* and element E in the hessian fly *M. destructor*, Fig. 2), to test for early differentiation of sex chromosomes in these lineages. None of the chromosomes in the homomorphic species showed the marked excess of male SNPs that is diagnostic of a recently formed neo-sex chromosome, suggesting that these species either have fully undifferentiated sex chromosomes or that the differentiated region is too small to shift the distribution of male SNPs for an entire chromosome. Similarly, rather than showing an excess of male SNPs, the Muller elements of *H. fusca* and *M. destructor* that have intermediate patterns of coverage also show a percentage of male SNPs that is intermediate between the ancient X and the autosomes. Rather than reflecting nascent differentiation, this is more likely the result of large-scale rearrangements between Muller elements in these groups. While this cannot be tested directly in *H. fusca*, a partially mapped genome of *M. destructor* is available [22]. When we apply our coverage pipeline to the mapped scaffolds of the hessian fly, we find that both X chromosomes are fully differentiated (X1 corresponds to elements D, E, and F, X2 to element C; S6 Fig. and S1 Table); the intermediate coverage value of element E is a result of its similar distribution between X1 and the autosome A2 (S1 Table).

Thus, our genomic analysis reveals a striking diversity of sex chromosomes in flies hidden underneath morphologically similar karyotypes; sex chromosomes have been lost, gained, rearranged or replaced multiple times over the course of Diptera evolution. Some chromosomal elements, such as element D, became sex-linked independently several times, suggesting that this chromosome may be pre-adapted to evolve into a sex chromosome (for example, it might harbor important genes of the sex determination cascade that can easily take over a master sex-determining function), and only element E is not sex-linked in any of the species investigated. Interestingly, while the dot has reverted back to an autosomal inheritance multiple times, none of the larger sex-linked rods have. This suggests that sex chromosome transitions may become increasingly difficult when more genes are sex-linked. Indeed, while the dot chromosome of *Drosophila* became autosomal >60 MY ago, it has maintained many characteristics of a former X chromosome, such as sex-biased expression patterns and chromosome-specific regulatory mechanisms [10]; such constraints may preclude the reversion of a sex chromosome that contains many hundreds of genes.

### Peculiar Patterns of Gene Expression on the X

A variety of evolutionary forces and functional constraints play out differently on X chromosomes versus autosomes: the X is hemizygous in males and transmitted more often through females [23], and this is thought to have shaped the unusual biology of the X chromosomes of model organisms. In particular, hemizygosity of the X in males drives the evolution of dosage compensation and results in roughly equal expression of X-linked genes in males and females. Hemizygosity of the X in males also increases the efficacy of selection for recessive, male-beneficial mutations and drives the accumulation of male-specific functions [24], while female-biased transmission favors the accumulation of female-beneficial mutations on the X [25]. Finally, sex-biased transmission makes sex chromosomes a hotbed for the invasion of selfish meiotic drivers that bias the sex ratio of progeny. Several taxa transcriptionally silence their sex chromosomes during male meiosis (meiotic X-inactivation), which may have evolved to prevent sex-ratio distorters from being expressed during gamete formation [26].

The joint action of these various forces is expected to shape the gene content and expression patterns of sex chromosomes, and they have all been invoked, to different extents, to explain sex-biased expression patterns in vertebrates and flies. Dosage compensation has evolved in both mammals and *Drosophila*, but by dramatically different mechanisms (up-regulation of the single X in male *Drosophila*, inactivation of one X in female eutherian mammals; [27]). Not all tissues are dosage compensated, however, and the MSL-complex, which mediates dosage compensation in fruit flies, is absent in *Drosophila* testis [28]. Other taxa with heteromorphic sex chromosomes, such as birds or snakes [29–31], completely lack dosage compensation altogether, and expression from their Z chromosome is generally male-biased. In *Drosophila*, the X has acquired higher expression in ovary, but lower expression in testis, relative to autosomes, and this gene expression pattern is consistent with the feminization that might result from being under selection primarily in females, and/or meiotic X-inactivation, and/or a lack of dosage compensation in the male germline. In contrast to this general pattern of feminized expression of the X in *Drosophila*, the mammalian X seems to be enriched for spermatogenesis genes that are expressed before the onset of meiotic X-inactivation [32], as well as for prostate genes, but not for ovary or mammary gland genes [33,34], suggestive of a masculinized X. It is at this point unclear how prevalent the evolution of dosage compensation is and by what mechanism it is achieved, if either masculizined or feminized expression patterns are general features of X-chromosomes, and what evolutionary forces or functional constraints lead to sex-biased expression patterns.

To study if independently formed sex chromosomes have evolved convergent biological properties (such as dosage compensation and sex-biased gene expression), we obtained gene expression profiles from male and female tissues of a subset of the species investigated. We focused on species with independently derived X chromosomes, as they are the most informative to study convergent patterns of X-linked gene expression evolution.

### Dosage Compensation through Up-Regulation of the X in Males

**Fig 3:**
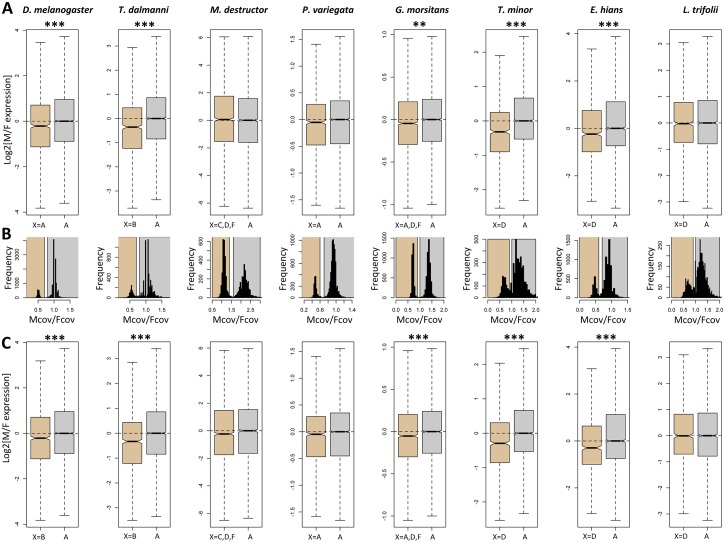
Assessing dosage compensation in Diptera using RNA-seq: Log2 of the male to female ratio of expression for the X (brown) and autosomes (grey) of several fly species. All boxplots represent whole-body male-to-female comparisons. Two approaches were used to identify X-linked and autosomal genes: (1) Genes were considered X-linked if their reciprocal-best-hit in *D. melanogaster* was located on the Muller element(s) that correspond to the X in the species, as shown in Fig. 1B and C, and (2) genes were classified as X-linked if they were located on a scaffold that had reduced male/female coverage, as shown on panel B. Scaffolds in the brown rectangles were classified as X-linked; scaffolds in the grey rectangles were classified as autosomal. The first classification was used to compare the male to female ratio of expression of genes on the X and autosomes in panel A and the second classification was used in panel C. Asterisks denote significant differences between the male to female ratio of expression on the X and the autosomes: * *p* < 0.05, *** *p* < 0.001, estimated using a Wilcoxon test. Data to generate this graph are to be found in file “S3 Data.”

*Drosophila melanogaster* has evolved dosage compensation by up-regulating the expression levels of the single-copy X-linked genes in males relative to females, thereby equalizing expression of the X chromosome between the sexes. To systematically test for the presence of dosage compensation in Diptera, we compared male to female expression ratios (M/F) of X-linked genes relative to autosomes in whole body samples in eight of our fly species, and we found that in all taxa where one of the large rods (elements A–E) has become a sex chromosome, some level of dosage compensation has also evolved, as the log2(M/F) expression of X-linked genes is well over -1, and often close to 0 (Fig. 3).

Assessing how expression on the X changed relative to its ancestral autosomal expression can yield insights into the mechanism of dosage compensation, as well as into possible sources of expression biases on the X. For instance, equal expression of X-linked genes in males and females can be achieved through up-regulation of the X in males (as in *Drosophila*) or down-regulation/inactivation of the X in females (as in mammals). These two strategies to evolve dosage compensation can be distinguished by contrasting current levels of gene expression on the X with the chromosome’s ancestral expression level before it became a sex chromosome, using outgroup species comparisons [35].

**Fig 4:**
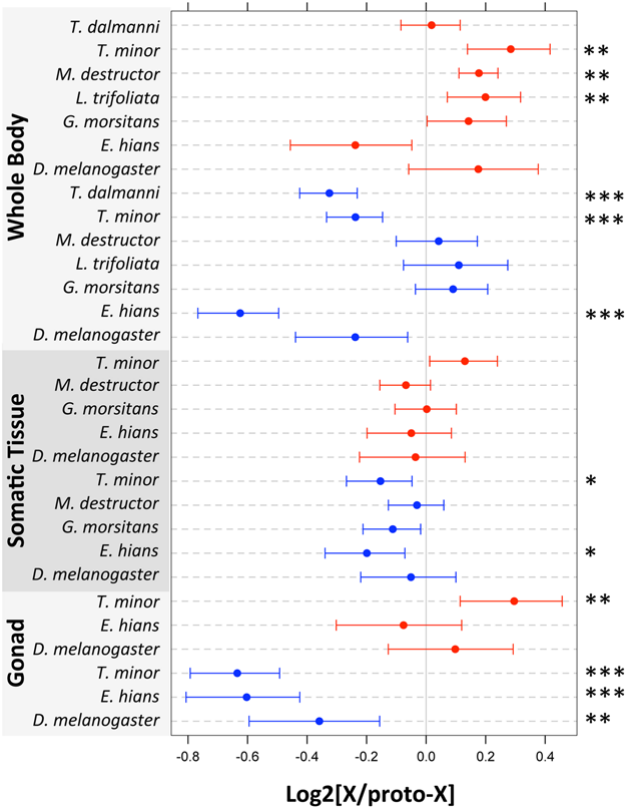
Expression on the X relative to the expression of the proto-X (approximated by the corresponding autosomal value in *D. melanogaster*; for *D. melanogaster*, *E. hians* was used to approximate autosomal expression). Female tissues are in red, male tissues in blue. Dots represent the median and bars their approximate confidence interval (median +/- 1.57 x IQR/√*n*, where IQR is the interquantile range and *n* the sample size; this is equivalent to the notch size of a boxplot). Asterisks denote significant increases or decreases in X-linked expression relative to the ancestral level: * *p* < 0.05, ** *p* < 0.01, *** *p* < 0.001, estimated by comparing X-linked and autosomal values using a Wilcoxon test. Data to generate this graph are to be found in file “S3 Data.”

**Fig 5:**
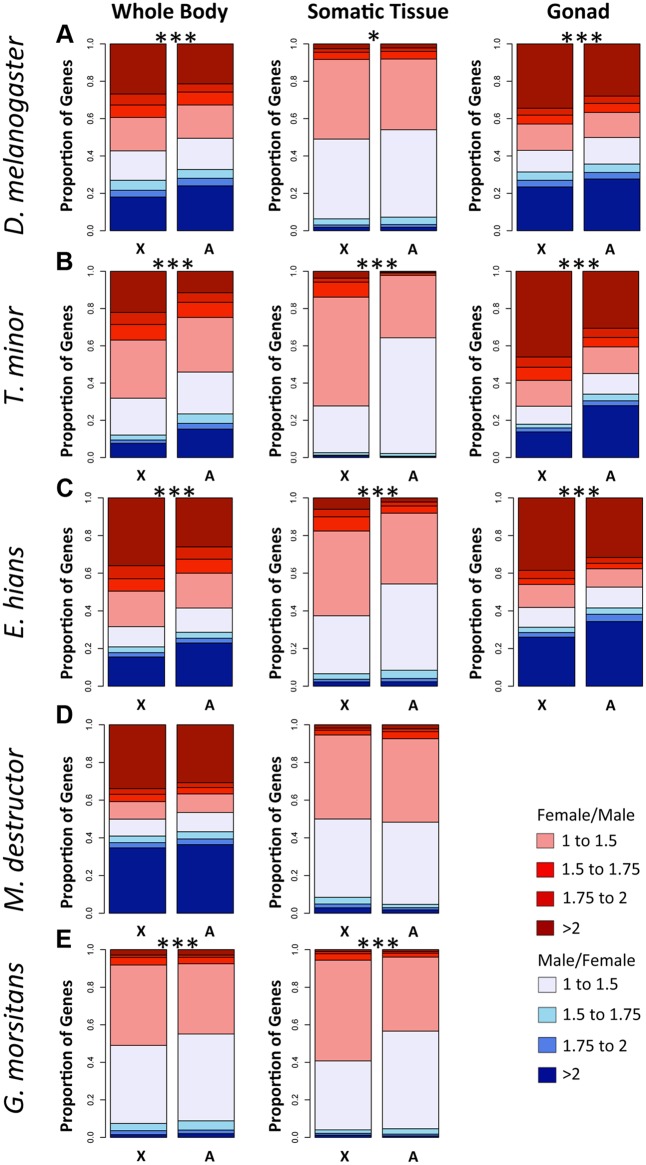
Proportion of male- and female-biased genes on the X and the autosomes , for different tissues of *D. melanogaster* (A), *T. minor* (B), *E. hians* (C), *M. destructor* (D), and *G. morsitans* (E). In *D. melanogaster*, *T. minor*, *E. hians*, and *G. morsitans*, the somatic tissue corresponds to the head; in *M. destructor*, publicly available antenna reads were used. Asterisks denote significant differences between the male to female ratio of expression on the X and the autosomes: * *p* < 0.05, *** *p* < 0.001, estimated using a Wilcoxon test. Data to generate this graph are to be found in file “S3 Data.”

We performed such an analysis by comparing gene expression on the X chromosomes of several dipteran insects to their corresponding autosomal value in *D. melanogaste*r (a proxy for their pre–sex-linked expression), to detect up- or down-regulation of X-linked genes in males and females relative to their ancestral value (genes that are autosomal in both species were used as controls). We similarly used shore fly (*E. hians*) expression values to infer ancestral expression of the X in *D. melanogaster*. Since dosage compensation is likely absent in testis of *Drosophila*, somatic tissues should be the most informative to investigate the evolution of dosage compensation. We find no reduction in female X-linked gene expression relative to ancestral levels for any of the species analyzed (Fig. 4). Instead, in each of these species, dosage compensation is achieved through up-regulation of the single X chromosome of males, consistent with mechanisms similar to the one found in *D. melanogaster* [36]. In some of the species, the up-regulation of the male X is not sufficient to fully re-establish ancestral levels of expression, causing a slight female bias observed for somatic tissues (Fig. 5).

A similar analysis of ancestral expression patterns in placental mammals found that balanced X-linked expression between the sexes was achieved through down-regulation of the female X, coupled with down-regulation of autosomal genes that interact with X-derived products in both sexes [35]. Marsupials, on the other hand, appear to globally up-regulate X-linked genes in both sexes, followed by X inactivation in females [35]. Monotremes, contrary to therian mammals, did not evolve any chromosome-wide mechanisms to equalize X-linked expression levels in males and females [35]. Thus, in contrast to mammals, in which gene dosage alterations associated with the emergence of sex chromosomes have been solved in various ways, dosage compensation in Diptera appears to have evolved relatively uniformly by the up-regulation of the single X in males. Whether this is due to the co-option of an existing ancestral dosage compensation machinery of flies or due to independent acquisition of dosage compensation by a similar mechanism will require the detailed study of dosage compensation in each species. The MSL-complex, which up-regulates the single X in male *Drosophila*, however, does not appear to function in other Diptera families [37].

In each of the species for which we have assayed gene expression in the gonads, the germline is characterized by a strong reduction of X-linked expression in testis relative to ancestral levels (Fig. 4). This is consistent with data from *Drosophila*, and suggests that dosage compensation may be lacking in the testes of all Diptera studied. Imbalances in X-linked and autosomal gene products in males are thought to be ultimately driving the evolution of dosage compensation, and it is not entirely clear why dosage compensation would a priori not be needed in the testes of so many species, which presumably also employ gene networks that contain X-linked and autosomal genes that may require balanced expression.

### Feminization and Demasculinization of the X

Thus, while dosage compensation has evolved repeatedly across flies, in five of the eight species (seven of the eight species before Bonferroni correction), including *D. melanogaster* with its well-characterized dosage compensation machinery, expression from the X is significantly reduced in males, relative to females. This has been interpreted in *Drosophila* as an evolutionary response of moving male-specific functions off the X chromosome (demasculinization), either by relocating genes or have their male-functions taken over by autosomal genes [38]. As discussed, the X may provide an unfavorable environment for genes primarily involved in male functions, due to its female-biased transmission [39–41] and/or its transcriptional silencing during male meiosis [38,42] and/or the lack of dosage compensation in testis [43]. It had also been suggested that the dosage compensation mechanism of *Drosophila* might directly prevent male-biased expression of X-linked genes by interfering with regulatory mechanisms that increase male expression [44,45], though a more recent analysis does not support this [46].

Tissue-specific RNA-seq data in *Drosophila* have shed some light on this expression bias: in tissues that are not sex-specific, such as head, the ratio of M/F expression between the X and the autosomes is much closer to 1, consistent with full dosage compensation (Fig. 5A) [43,47,48]. Interestingly, we detect a small, but significant, excess of female relative to male expression of the X in *Drosophila* head tissue (Fig. 5A), but, as expected, much more dramatic expression differences are found in sex-specific tissues: the X is over-expressed in ovary, but under-expressed in testis (Fig. 5A). To check the generality of these patterns, we obtained RNA-seq data from male and female heads and from ovary and testis from scavenger flies (*T. minor*) and shore flies (*E. hians*), two clades in which element D became independently sex-linked. We also analyzed published head and antenna RNA-seq data for the tsetse fly, *G. morsitans*, and the hessian fly, *M. destructor*, whose X chromosomes consist of multiple Muller elements, and compared the somatic data to gene expression patterns obtained for the whole body (Fig. 5). Consistent with the *Drosophila* data, we find that in each species, gonad tissue contain the highest proportion of strongly sex-biased genes (shown in dark red and dark blue), while somatic tissues contain the lowest (Fig. 5). This observation supports the view that the large amount of sex-biased expression found in whole-body comparisons is largely driven by differences in gene expression between ovaries and testes.

As discussed above, the MSL-complex is absent in *Drosophila* testis [28], and the strong reduction of X-linked expression in the testis relative to ancestral levels observed for both *E. hians* and *T. minor* suggests that dosage compensation is generally absent in the testis of Diptera flies (Fig. 4). A lack of dosage compensation in testes could certainly lead to the observed paucity of testis-biased expression from the X chromosome in Diptera [43]. However, we also observe a general deficiency of genes with testis-specific (or highly testis-biased) expression on the X relative to autosomes, above what would be expected by a simple lack of dosage compensation (which would only result in a deficiency of testis-biased genes that show a 2-fold difference in expression levels or less; S7 Fig. [43]). Meiotic X-inactivation is a well-studied phenomenon in mammals in which the X is epigenetically modified to repress transcription throughout male meiosis [49], but in *Drosophila*, the X remains active throughout spermatogenesis [38,50]. Expression of the X in *Drosophila* is reduced for some genes, potentially reflecting a gene-by-gene version of the chromosome-wide mammalian X-inactivation [38,50]. The X chromosome of shore flies and scavenger flies is widely expressed in testis (though at a lower level), suggesting that meiotic X-inactivation, if present, also acts on a more local scale, as in *Drosophila*. Note that our transcriptome analysis is performed on whole testis, and we might miss important details about the suite of X genes expressed at specific spermatogenic stages. As discussed above, meiotic X-inactivation should result in a deficiency of X-linked testis genes that are expressed during and after meiosis, but testis genes expressed before meiosis may in fact be enriched on the X, as has been found in mammals [32]. Detailed transcriptome analysis of pre- and post-meiotic cell populations should reveal whether a similar pattern exists among flies.

While transcriptional constraints (i.e., lack of dosage compensation in testis, meiotic X-inactivation) may account for sex-biased expression of the X in the gonads [23,50], somatic sex-biases are generally thought to be driven by differential sex-specific or sex-antagonistic selective pressures on the X chromosome [34]. Indeed, the X is female-biased in head samples for four of our five species, although to a much lesser extent than in whole-body or gonad tissue (Fig. 5). Furthermore, genes expressed primarily in the male-specific accessory glands of *D. melanogaster* are also under-represented on the X chromosome [51]. This shows that genes with male function in somatic tissues also avoid the X chromosome, though to a much lower degree than testis genes, and suggests that sex-specific or sexually antagonistic selection is contributing to the observed deficiency of genes with male functions on the X (though it is also possible that dosage compensation is not complete even in somatic tissues). Importantly, these theories are not mutually exclusive, and a combination of them is likely to be influencing the extent to which the X is feminized and demasculinized in different species and tissues. For example, more frequent or stronger sexually antagonistic selection in gonads versus somatic tissue could result in the more pronounced patterns of sex-biased gene expression in gonads and the resulting lack of testis-specific genes on the X, through gene relocation or reassignment of male functions to other autosomal genes, which could in turn mitigate the need for dosage compensation in testis, reinforcing the observed patterns. Similarly, meiotic X-inactivation would select for testis-specific functions to be moved off the X, again diminishing the need for dosage compensation in testis.

Whole-body comparisons yield a variety of sex-biased expression patterns, but are more difficult to interpret due to the mixture of shared somatic and sex-specific tissues (Fig. 4). For instance, *M. destructor* and *L. trifolii* show an up-regulation of the X in the female, but no change in the male; in *T. dalmanni* and *E. hians*, the X is down-regulated in the male samples, but remains unchanged in the female samples; *T. minor* up-regulates the X in females and down-regulates it in males; finally, *G. morsitans* and *D. melanogaster* do not show significant changes in X-linked gene expression in either sex. However, while male and female expression evolved in varying directions in different species after the X became sex-linked, the direction and magnitude of these changes consistently result in a relative deficiency of male expression of the X, relative to females. In *Drosophila*, a combination of selective extinction of male genes on the X chromosome, the creation of new male-biased genes on autosomes, and relocation of existing genes with testis-biased expression off the X contribute to the unusual X chromosome gene content [41,47]. Future detailed comparative studies of high-quality genomes and transcriptomes of fly species with independently formed sex chromosomes should reveal the underlying mutational routes by which X chromosomes have become demasculinized repeatedly across flies.

### Conclusion

To conclude, we uncover a striking diversity of sex chromosome configurations in Diptera, concealed underneath superficially similar karyotypes displaying male heterogamety. This diversity is in sharp contrast to the theory that differentiated sex chromosomes are highly stable and unlikely to be replaced, exemplified by the mammalian X, and emphasizes the importance of studying a variety of clades to obtain a truly global understanding of sex chromosome evolution. Recent sex chromosome additions have already been used extensively in *Drosophila* to study processes of sex chromosome differentiation [52,53], and independently evolved X and Y chromosomes of different fly families can be utilized to study sex chromosome biology in a comparative manner. We show that sex chromosomes of flies have evolved dosage compensation by transcriptional up-regulation of the single male X. However, X chromosomes generally show a deficiency of genes with male-biased expression, particularly in the gonads, suggesting that the evolution of sex chromosomes is likely shaped by the interplay between sex-specific and sexually antagonistic forces, mechanistic constraints, and selective pressures to preserve the regulation of ancestral gene expression.

## Materials and Methods

### Origin of Specimens

The origin and method of preservation of the specimens used is described in S2 Table. Data was already available for six of the species investigated [10,21].

### DNA and RNA Extraction and Sequencing

DNA was extracted from fresh or preserved individuals using the Puregene DNA kit (Qiagen), following the manufacturer’s protocol. RNA was extracted using a standard Trizol extraction with isopropanol precipitation. Illumina paired-end RNA-seq libraries (with a 200 bp fragment size) and DNA-seq libraries (with 500 to 800 bp fragment sizes) were produced using the manufacturer’s standard protocol and sequenced at the QB3 Vincent J. Coates Genomics Sequencing Laboratory at University of California Berkeley. All DNA/RNA-seq reads generated for this study are deposited at http://www.ncbi.nlm.nih.gov/sra under the bioproject accession numbers SRP050301 (genome data) and SRP050303 (transcriptome data), and the accession numbers for each dataset can be found in file S4 Text.

### Coverage Analysis

The first 10 bps of DNA reads were trimmed. For each species, the male and female reads were pooled and assembled using SOAPdenovo [54] (with a kmer of 31; for some species, marked in S1 Table, a better assembly was obtained using the female data only, and this assembly was used instead); only scaffolds longer than 1,000 bps were kept for the coverage analysis. Male and female reads were mapped back to the assembled scaffolds using BWA [55], and male and female coverage were calculated from the resulting SAM files (after filtering for uniquely mapped reads by keeping only lines of the SAM file that match ‘XT:A:U’) using SoapCoverage (http://soap.genomics.org.cn/soapaligner.html). Scaffolds were assigned to a Muller element based on their gene content; *Drosophila melanogaster* CDS sequences were obtained from Flybase, and only the longest CDS was kept for each gene. The filtered CDS were mapped against each of the dipteran genomes using Blat [56] (with a translated query and database, and keeping only hits with a score above 50). When CDS mapped to several locations, only the location with the highest mapping score was kept. Scaffolds were assigned to the Muller element that the majority of the genes they contain belongs to in *D. melanogaster*. Scaffolds containing the same number of genes from different elements were excluded from the analysis.

### Detection of Differentiated Sex Chromosomes

The presence of differentiated sex-linked Muller elements was determined using an R script.

First, the median autosomal Log2(M/F coverage), which we call A_coord, was assumed to correspond to the Log2(M/F coverage) with maximum frequency in the sample. Scaffolds were considered to be X candidates if they had Log2(M/F coverage) within the range [A_coord -1.1, A_coord -0.9]; in the case of Z-candidates, the Log2(M/F coverage) values are [A_coord + 0.9, A_coord + 1.1]. The number of observed X candidate scaffolds derived from each Muller element was then compared to their expected number (based on the overall frequency of each Muller element in the sample and the percentage of scaffolds that fall within the X-linked range) using a Fisher’s exact test. Muller elements that were significantly over-represented (*p* < 0.01) were classified as differentiated sex chromosomes. This pipeline had to be modified for *M. destructor* because in this species the largest peak corresponds to the X chromosomes, as only a minority of the genome is autosomal. We therefore used the second-largest peak to detect the autosomal median Log2(M/F) and to derive the range of Log2(M/F) for the X. The R code used to determine which elements are sex-linked is provided in S1 Text.

### SNP Detection

Male and female reads were mapped separately to the genomic scaffolds described above (after size selection and assignment to a Muller element) using Bowtie [57]. The resulting MAP alignment files were converted to PRO alignment files with Bow2Pro.pl (http://guanine.evolbio.mpg.de/mlRho/), which were filtered to keep only sites that had a minimum coverage depth of six. A SNP was called at a site when at least two different alleles each had a count of at least 0.3 × coverage at that site (as the coverage increases for the site, so does the minimum cut-off to call SNPs, making the SNP calling mostly independent of scaffold coverage).

### RNA-seq Assembly

**All species but *G. morsitans***. The first 15 bps of the Illumina RNA-seq reads were trimmed. For each species, male and female reads were pooled and assembled with SOAPdenovo-Trans [58]; only transcripts longer than 300 bps were kept. Each transcript was mapped to the filtered *D. melanogaster* CDS sequences (described in the previous sections) using Blat (with a translated query and database). When transcripts mapped to more than one *D. melanogaster* CDS, only the location with the largest mapping score was kept. When several transcripts mapped to the same *D. melanogaster* CDS, only the transcript with the largest mapping score was kept if they overlapped by at least 20 bps; otherwise they were concatenated. This reciprocal approach yielded an approximate 1 to 1 correspondence between our Diptera and *D. melanogaster* genes, allowing for the direct comparisons shown in Fig. 4.

***G. morsitans***. Solid ColorSpace reads for whole body and head male and female RNA-seq samples were provided by Karyn Megy at the European Bioinformatics Institute. CDS sequences were extracted from the *G. morsitans* genome, to be used in the expression estimation (next section), through the following steps: (1.) *D. melanogaster* CDS sequences (described in the previous sections) were mapped against the *G. morsitans* genome, using blat with a translated query and database; only reciprocal best hits were kept. (2.) The gene regions identified in step 1 were extracted from the *G. morsitans* genome using a perl script. (3.) Transcript sequences were obtained from the *G. morsitans* gene sequences, using Genewise [59] with the respective *D. melanogaster* protein sequence.

### Expression Estimation

Gene expression values for all *D. melanogaster* genes were obtained from the Berkeley Drosophila Genome Project (http://www.fruitfly.org/sequence/download.html#modencode_expression_scores). For each of the other species, male and female reads were mapped separately to the transcript sequences using Bowtie2 [60] with default parameters, and FPKM values (fragments per kilobase of transcript per million mapped reads) were estimated from the resulting SAM files using eXpress [61]. All analyses exclude genes that have FPKM <1. In Fig. 3 and Fig. 4, quantile normalization between male (M) and female (F) FPKM was performed using the preprocessCore R package. Since in a few cases the median of M/F was not exactly 1 for the autosomes even after the normalization, we divide all M/F values by the median autosomal M/F for that sample before plotting the data. This does not change the rank of the current/ancestral (C/A) expression values, but simply centers the autosomal control at 1. For Fig. 4, the same quantile normalization was used to normalize the current (C) and ancestral (A, approximated by the corresponding *D. melanogaster* FPKM value) data within each sex. As before, since in a few cases the median of C/A was not exactly 1 for the autosomes even after the normalization, we divide all C/A values by the median autosomal C/A for that sample before plotting the data, allowing us to show only the value for the X (since the autosomal control is centered at 1).

### Phylogenomic Tree of Insect Families Used in This Study

In order to minimize the issue of missing data, only one species per family (the species with the best genome assembly) was used to build the tree. Homologues of *D. melanogaster* genes were located on the genomes of each outgroup species by mapping *D. melanogaster* coding sequences with Blat [56] (with a translated query and database, and keeping only reciprocal best hits). Genewise was then used on these homologous DNA segments (using the *D. melanogaster* protein sequence as input) to obtain protein-coding sequences for each of them.

Protein sequences of genes that were found in all the species were aligned using Muscle; all the alignments were then concatenated, and low-quality sections were filtered out using Gblocks [62] (with default parameters, but allowing sections to be kept when they were represented in at least half of the species). The final alignment consists of 129,468 polymorphic sites in 185,388 amino acids. PhyML [63] with default parameters and 100 bootstrap cycles was used to obtain the tree shown in S4 Fig.

## Supporting Information

S1 Data This worksheet contains the male and female coverage values for each scaffold larger than 1000bps in the genome of each species studied, as well as the chromosome of its putative homolog in *D. melanogaster*. (XLSX)

S2 Data This worksheet contains the male and female SNPs numbers and the male and female coverage values for each scaffold larger than 1000bps in the genome, as well as the chromosome of its putative homolog in *D. melanogaster*. (XLSX)

S3 Data This worksheet contains the expression values for each of the tissues examined, as well as their corresponding expression in several *D. melanogaster* tissues. (XLSX)

S4 Data This worksheet contains the male and female coverage values for each scaffold larger than 1000bps in the genome, as well as the chromosome of its putative homolog in *A. gambiae*. (XLSX)

S5 Data This worksheet shows the coverage for scaffolds of the published genome of *Mayetiola destructor*. (XLSX)

S1 Fig Male and female coverage for Muller elements mapped against the *D. melanogaster* genome. Shown is Log2 of Female (in red), Male (in blue), and M/F (in green) coverage for each Muller element for each species investigated. Data to generate this graph are to be found in file “S1 Data.”
(PDF)

S2 Fig Male and female coverage for Muller elements mapped against the *A. gambiae* genome. Shown is Log2 of Female (in red), Male (in blue), and M/F (in green) coverage for each Muller element for each Nematocera species investigated. Data to generate this graph are to be found in file “S1 Data.”
(PDF)

S3 Fig Identification of differentiated sex chromosomes. For each species, the left panel shows the density plot of Log2(M/F coverage) for all scaffolds. The black vertical line shows the maximum frequency, which is assumed to correspond to the median autosomal Log2(M/F coverage) (except in *M. destructor*, for which the second-highest frequency was considered to be the autosomal median; see Materials and Methods). The dotted red lines delimit the interval of Log2(M/F coverage) that we assign to our candidate X/Z scaffolds. The right panel shows the observed/expected number of scaffolds from each Muller element among the Z/X-candidates. Significant excesses (*p* < 0.01) are shown in red, non-significant differences are in blue. Data to generate this graph are to be found in file “S4 Data.”
(PDF)

S4 Fig A phylogeny of the Diptera families used in this study, obtained from their genomic data. To minimize the issue of missing data, only one species was used per family, corresponding to the best genome assembly. *Drosophila* genes were queried against the genomes of all other species to find gene homologs, using a reciprocal best-hit approach. Genes present in all the families were aligned, concatenated, and low-quality sections of the alignment were filtered out. The tree was obtained from this final alignment using PhyML with default parameters, and 100 bootstraps (bootstrap support values are shown next to the nodes). Data to generate the phylogeny are to be found in file “S2 Text” and “S3 Text.”
(PDF)

S5 Fig Female and male SNP densities for the species shown in Fig. 2. SNP densities are shown for: *Drosophila miranda* (A), *Drosophila albomicans* (B), *Drosophila busckii* (C), *Scaptodrosophila lebanonensis* (D), *Holcocephala fusca* (E), *Mayetiola destructor* (F), *Calliphora erythrocephala* (G), and *Megaselia abdita* (H). Data to generate this graph are to be found in file “S2 Data.”
(PDF)

S6 Fig Female, male, and male/female coverage for mapped scaffolds of Mayetiola destructor (obtained by mapping our female and male reads against the published genome scaffolds). Data to generate this graph are to be found in file “S5 Data.”
(PDF)

S7 Fig Percentage of strongly testis-biased and testis-specific genes on the X and the autosomes of *E. hians*, *T. minor*, and *D. melanogaster*. Genes were considered to be strongly testis-biased (A) if their expression was 3-fold higher, and testis-specific (B) if their expression was 10-fold higher in testis than in male head, female head, female body, and ovary. Significant differences in the proportion of testis-biased and testis-specific genes on the X and autosomes were assessed with Pearson Chi-Square tests. Significance levels are represented by asterisks: * *p* < 0.05, ** *p* < 0.01, *** *p* < 0.001. Data to generate this graph are to be found in file “S3 Data.”
(PDF)

S1 Table *Drosophila melanogaster/Mayetiola destructor* synteny table. *D. melanogaster* genes were located on the *M. destructor* chromosomal scaffolds using blat (keeping only reciprocal best hits). The number of genes that maps to the different chromosomes of *M. destructor* is shown for each *D. melanogaster* Muller element. Significant excesses, detected using Pearson Chi-Square tests, are shown (***,*p* < 0.0001; * *p* < 0.05).
(DOCX)

S2 Table Origin and method of preservation of the specimens used. (DOCX)

S1 Text R code used to detect differentiated X-linked elements (1), Z-linked elements (2) and X-linked elements in *M. destructor*, where the majority of the genome is X-linked. (DOCX)

S2 Text Amino-acid alignment to construct a phylogenomic tree of insect families used in this study. (TXT)

S3 Text Phylogenetic tree obtained for the insect families used in this study using PhyML with default parameters. (TXT)

S4 Text List of Accession numbers of genomic reads generated for this study. (TXT)
