# Supplementary material for: Numerous Transitions of Sex Chromosomes in Diptera
Source: PLoS Biol. 2015 Apr 16;13(4):e1002078. doi: 10.1371/journal.pbio.1002078 (PMC4400102; doi:10.1371/journal.pbio.1002078)
Supplement: S1 Fig — Shown is Log2 of Female (in red), Male (in blue), and M/F (in green) coverage for each Muller element for each species investigated. Data to generate this graph are to be found in file “S1 Data.” (PDF) [file pbio.1002078.s006.pdf]

S1.1 Tipula olearacea

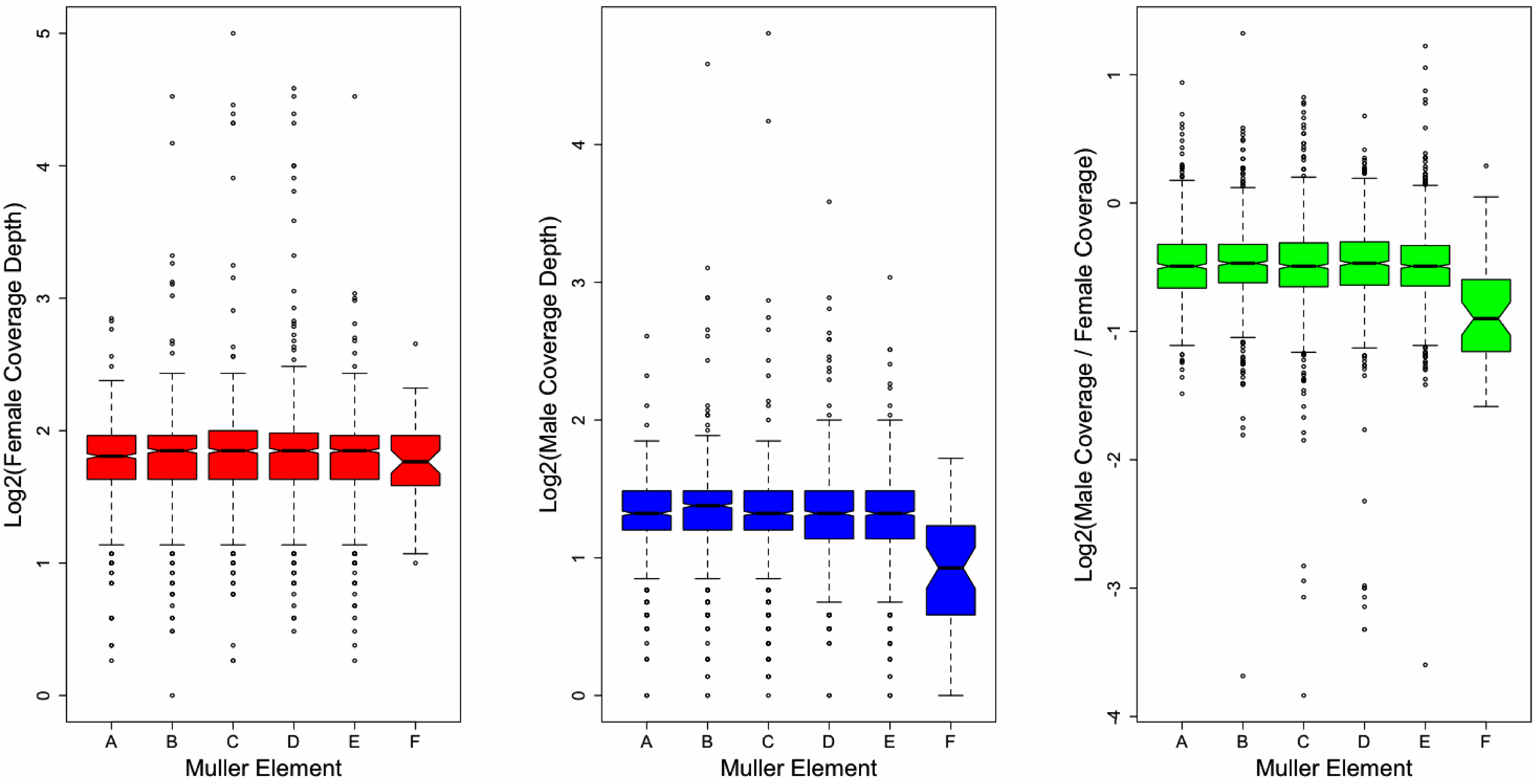

S1.2 Trichoceridae sp

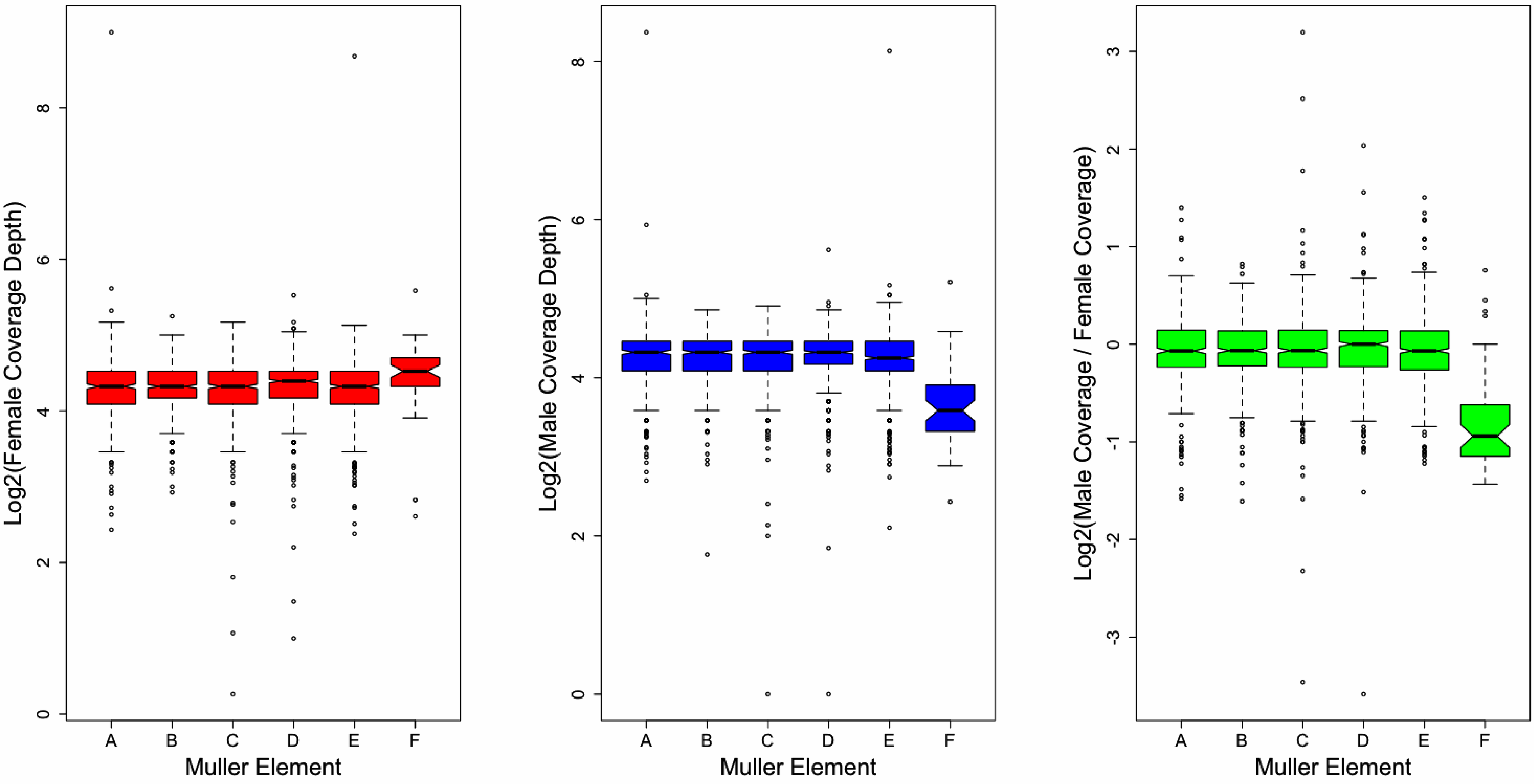

Figure S1

S1.3 Clogmia albipunctata

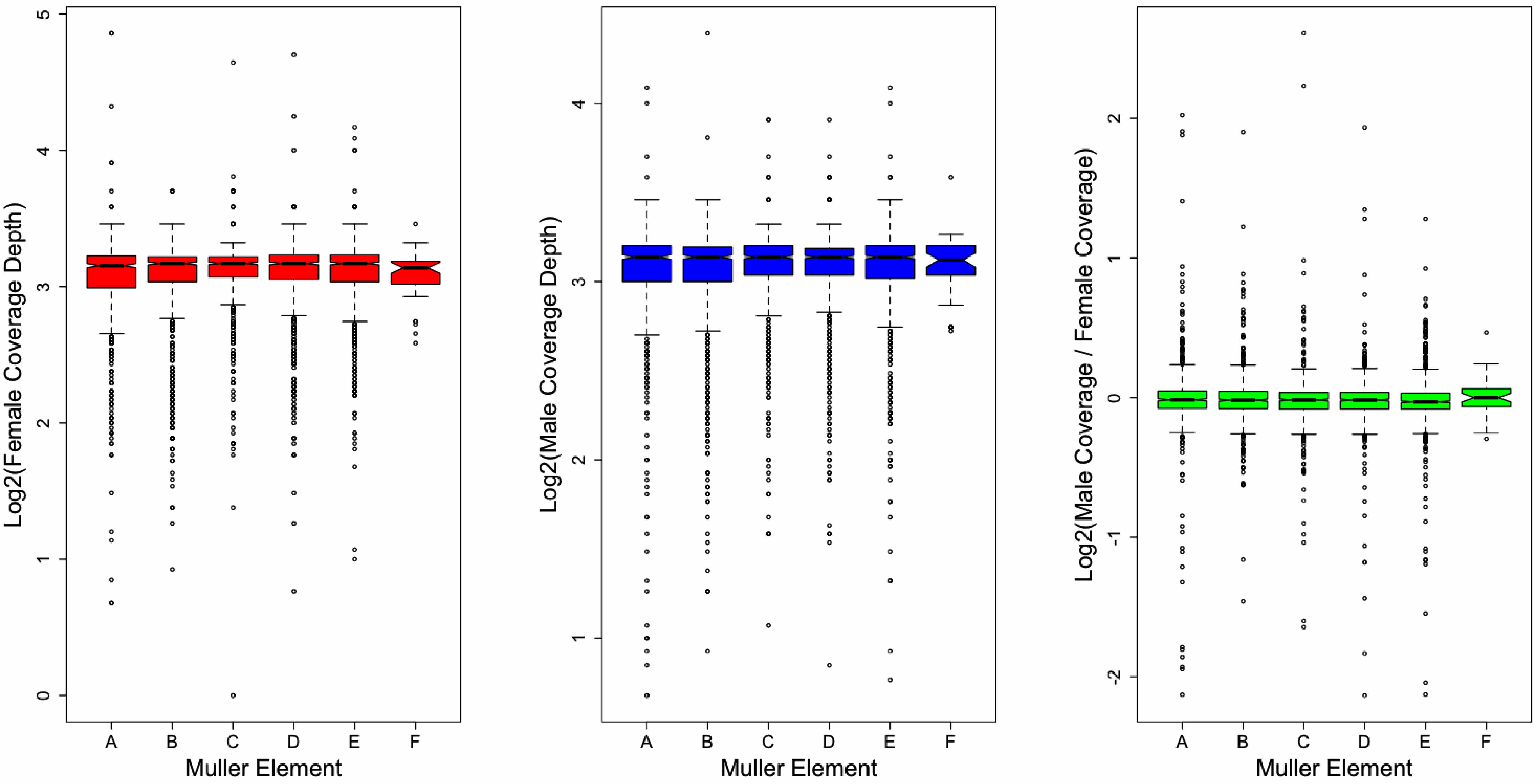

S1.4 Chironomus riparius

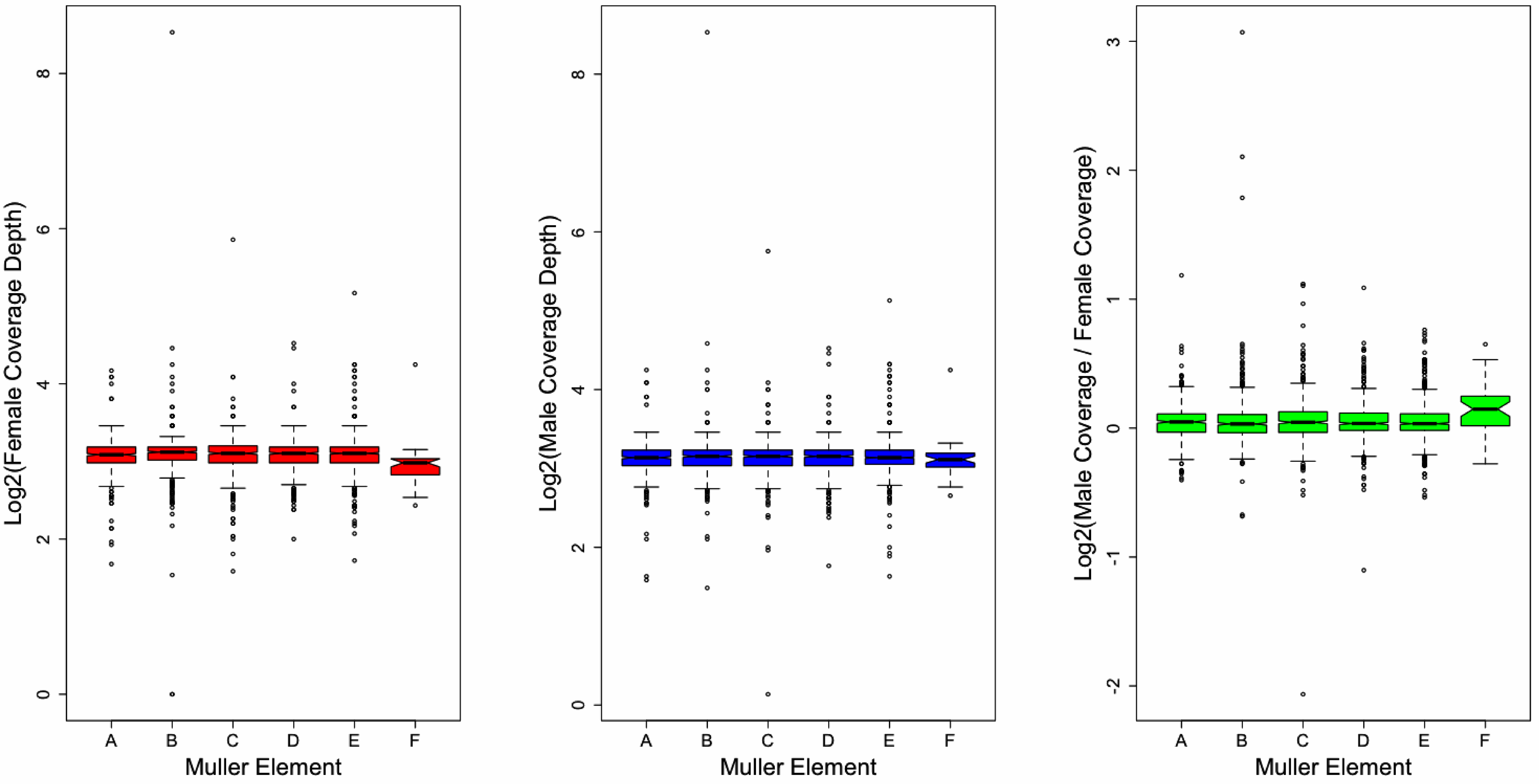

Figure S1

S1.5 Chaoborus trivittatus

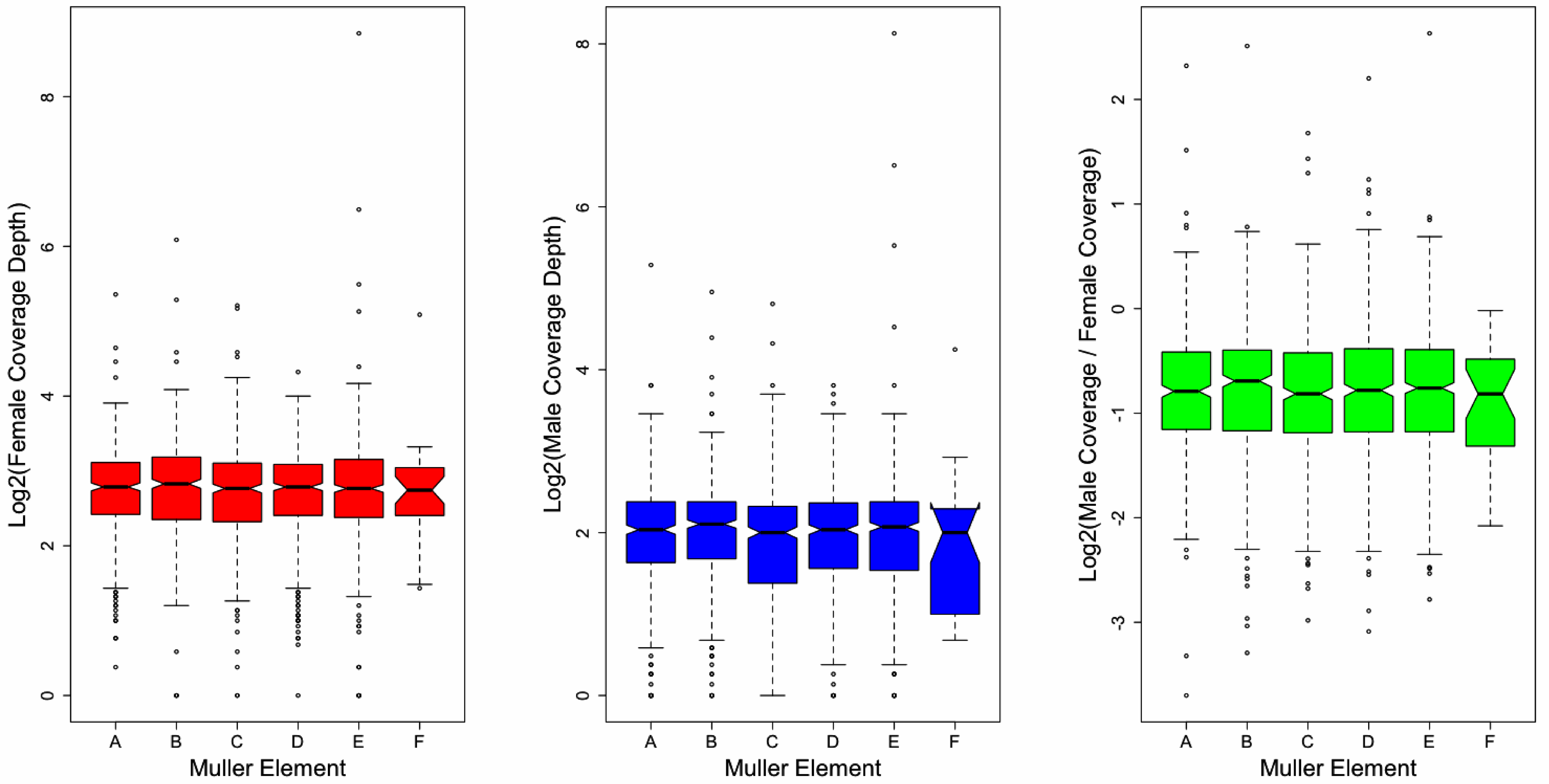

S1.6 Monchlonyx cinctipes

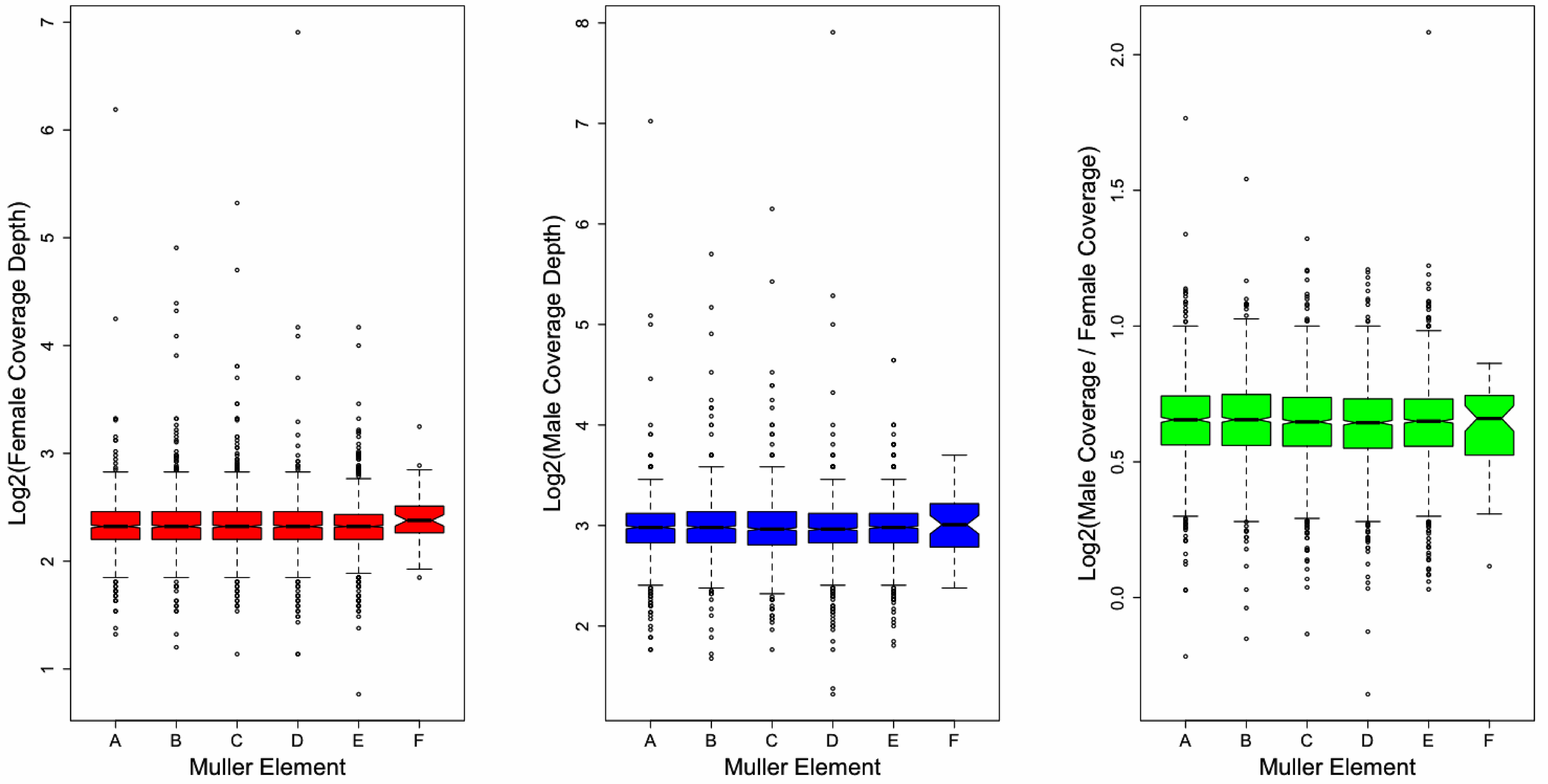

Figure S1

S1.7 Anopheles gambiae

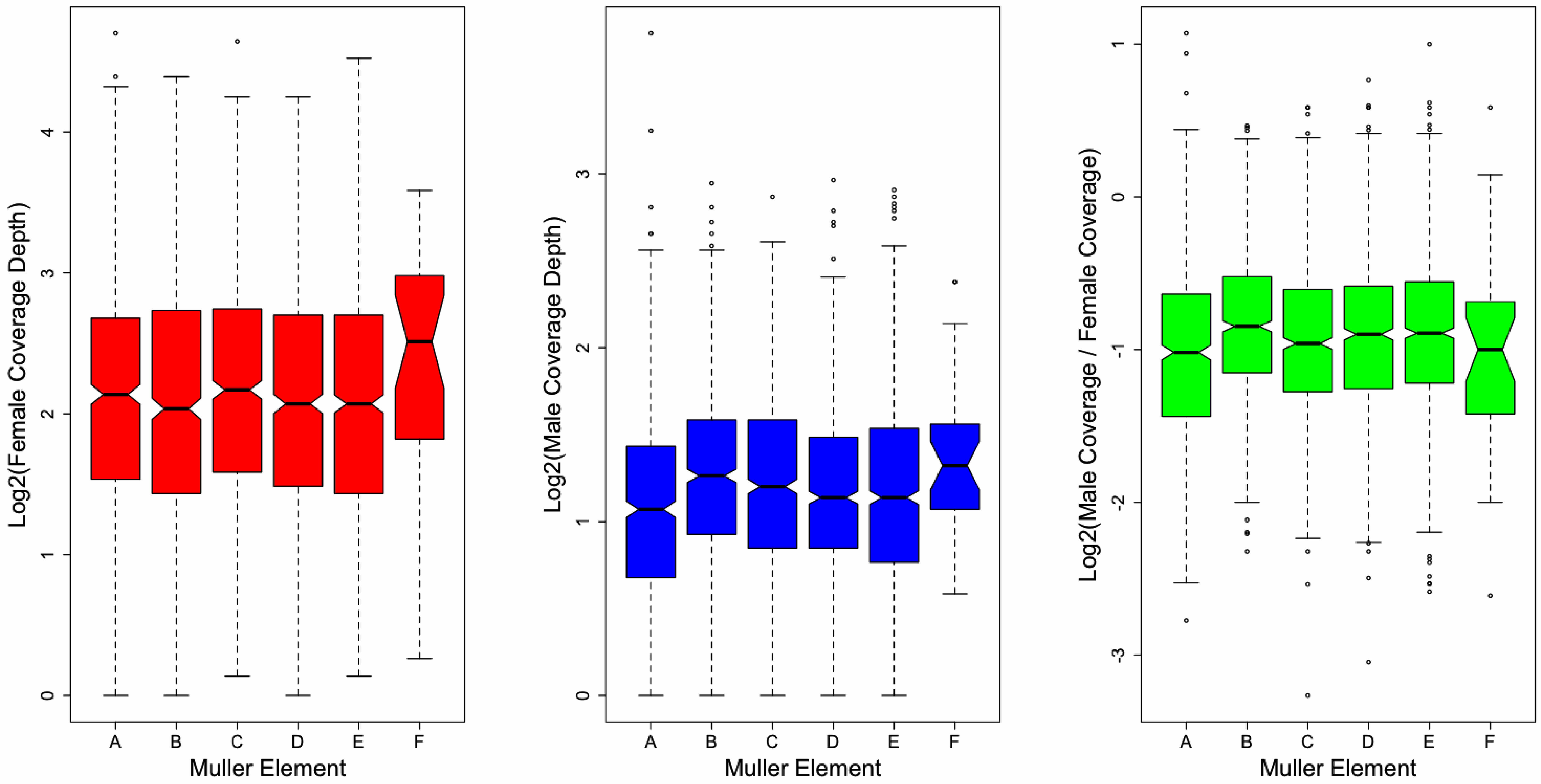

S1.8 Aedes aegypti

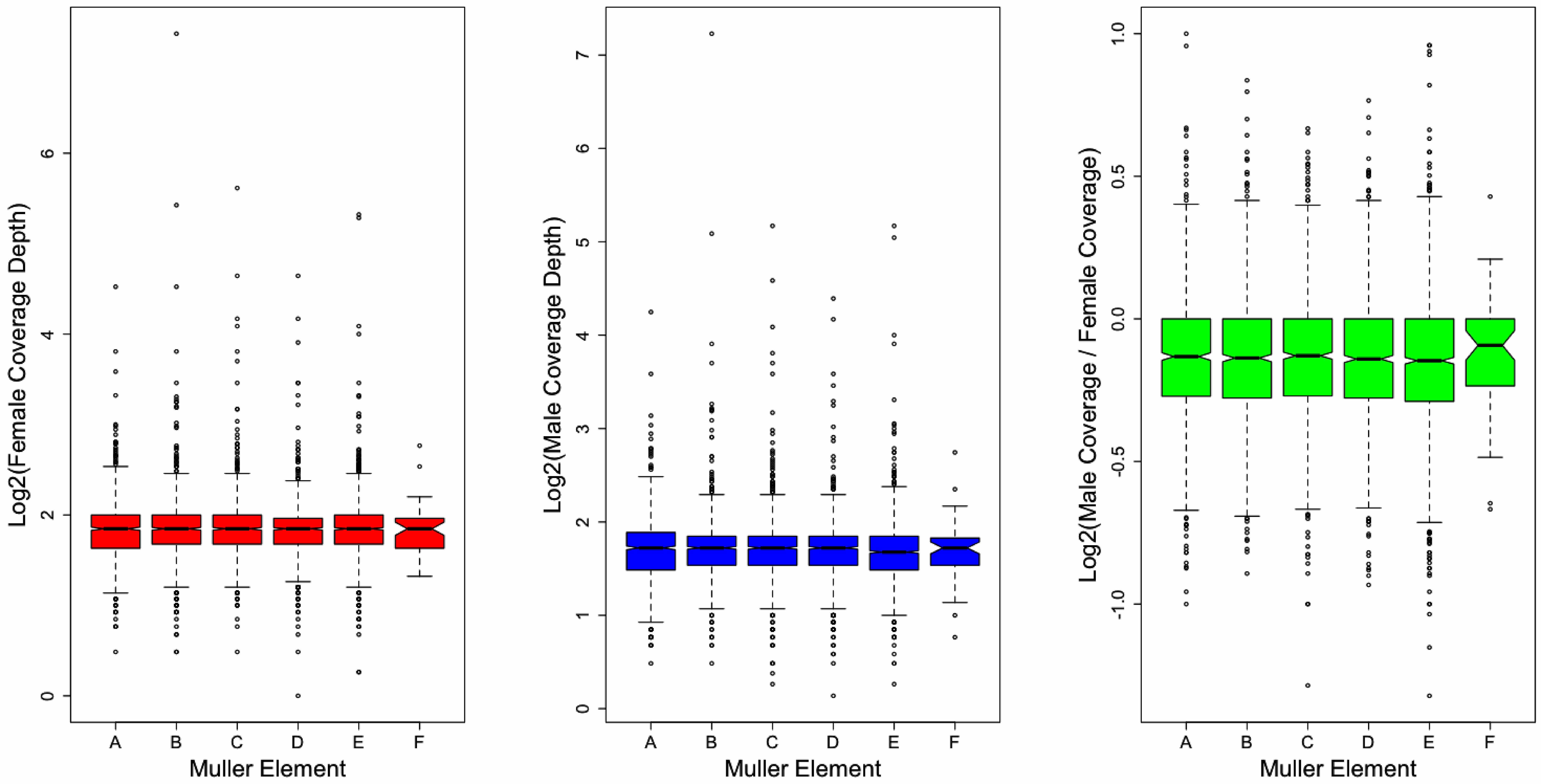

Figure S1

S1.9 Coboldia fuscipes

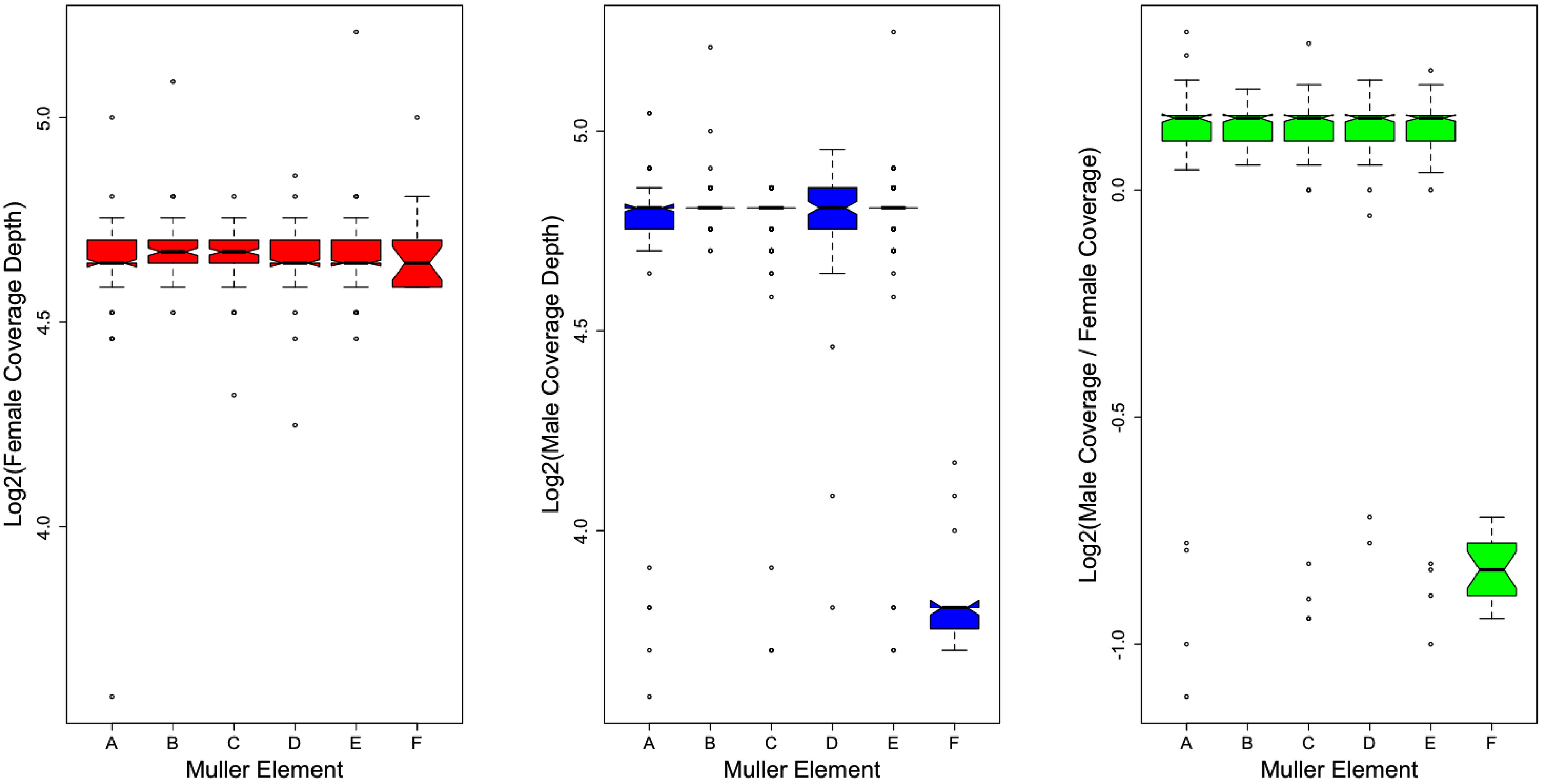

S1.10 Mayetiola destructor

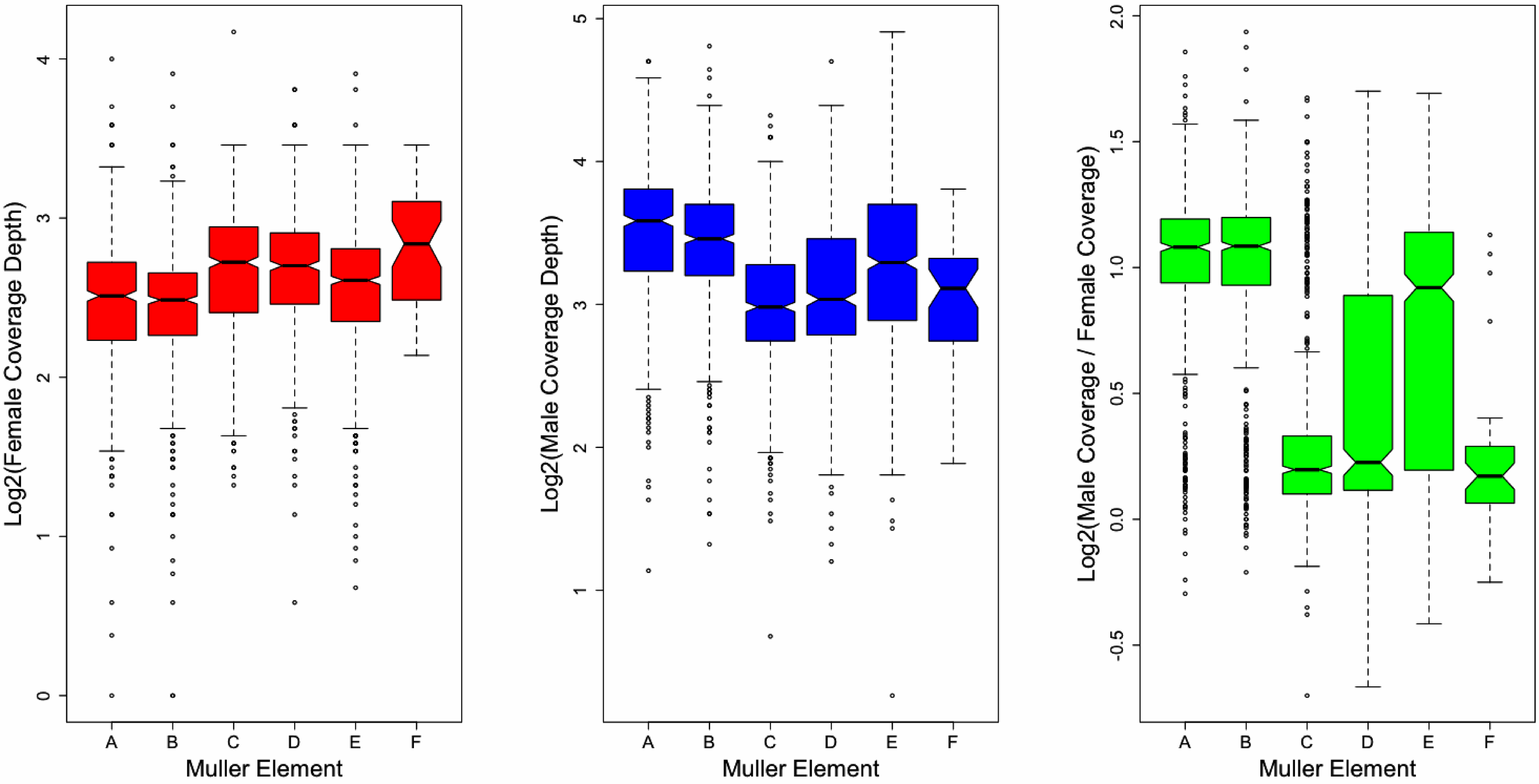

Figure S1

S1.11 Hermetia illucens

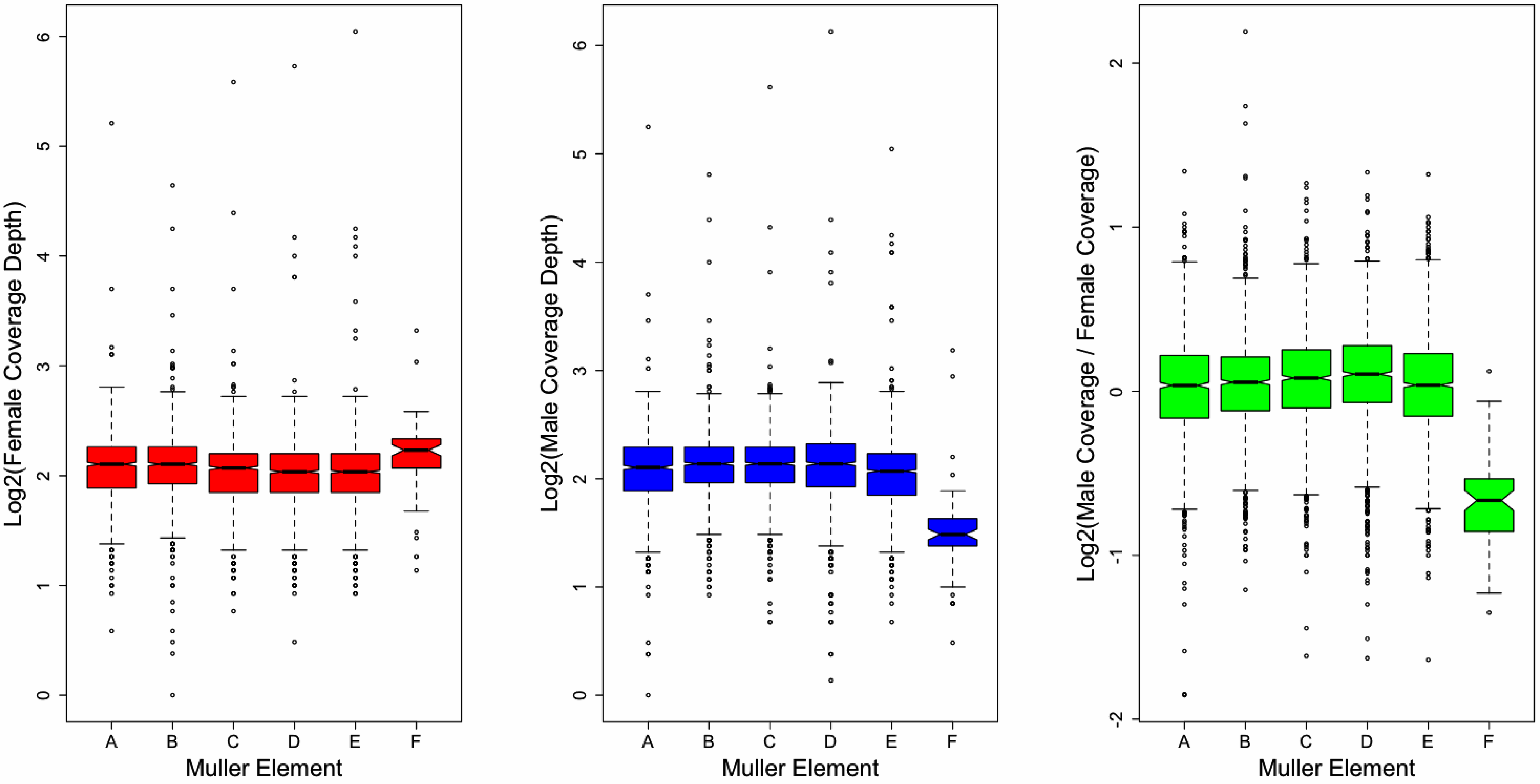

S1.12 Holcocephala fusca

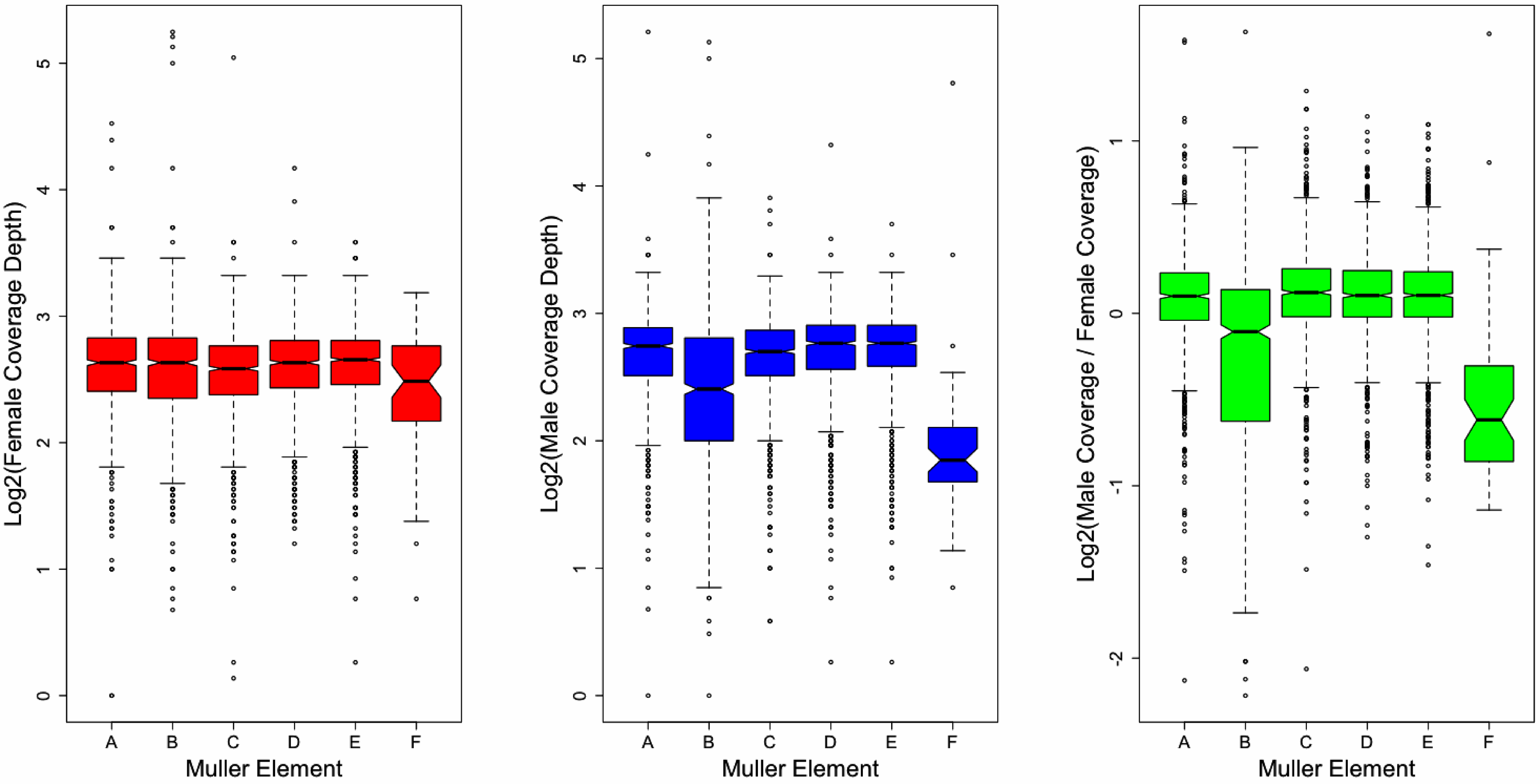

Figure S1

S1.13 *Condylostylus patibulatus*

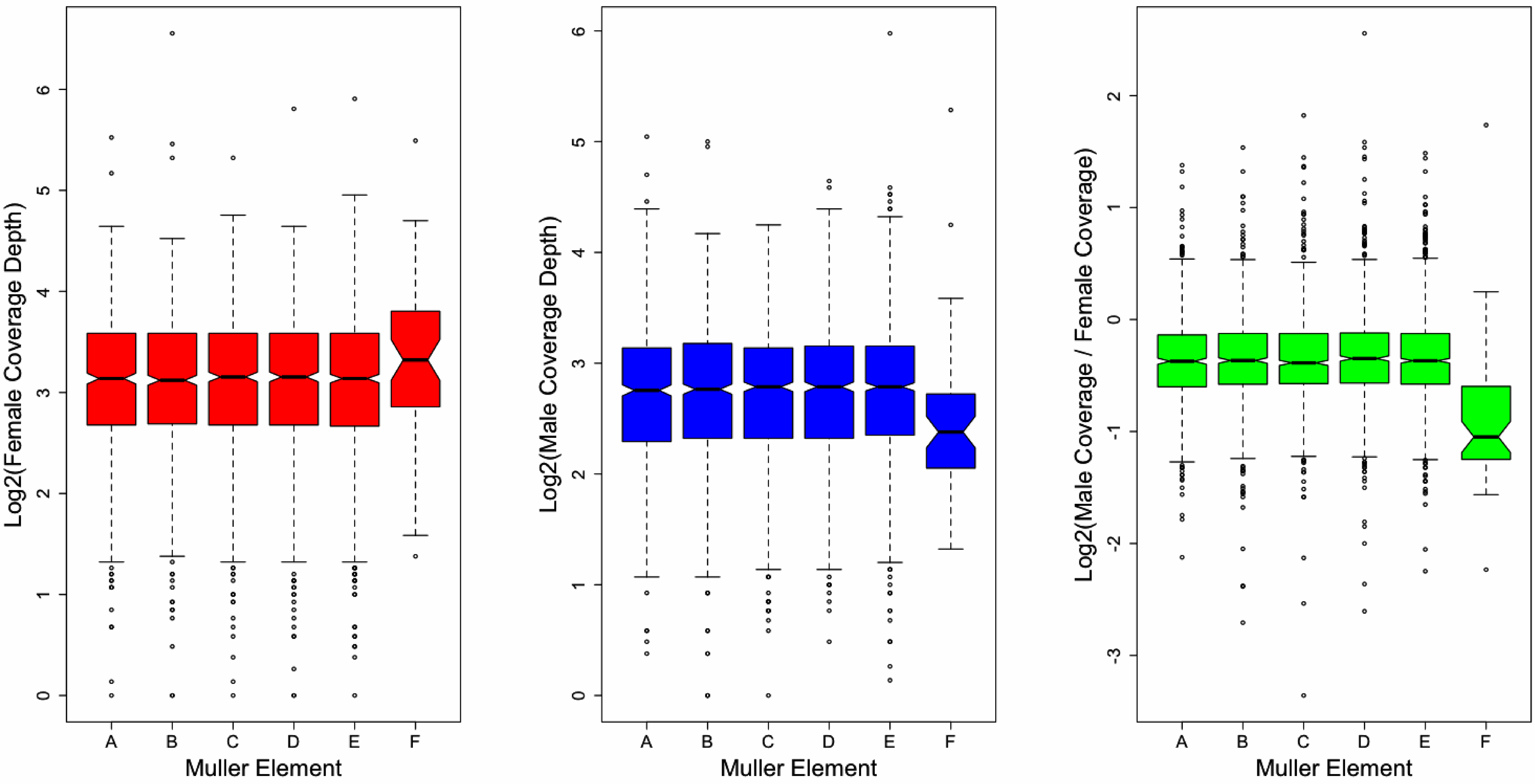

S1.14 *Megaselia abdita*

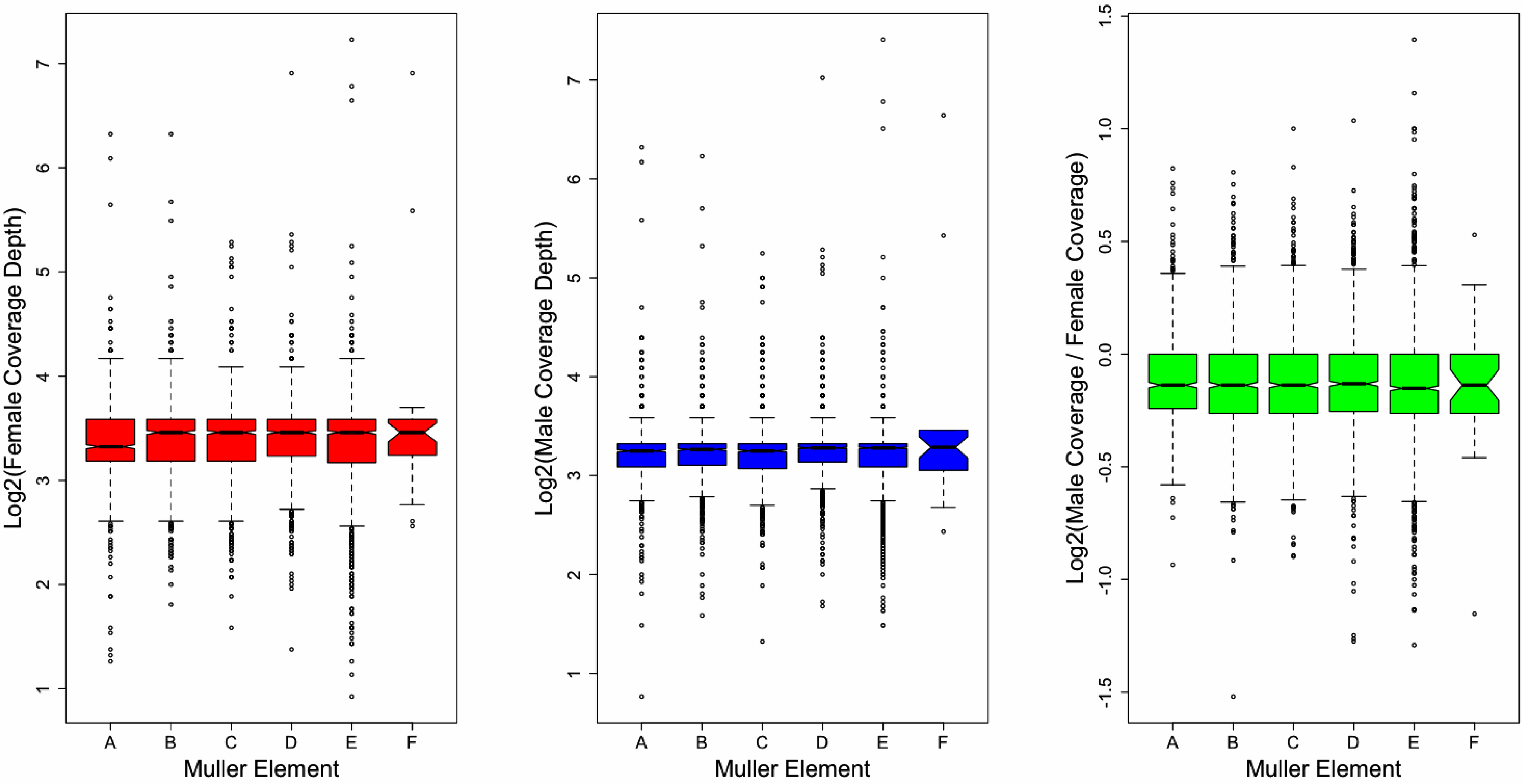

Figure S1

S1.15 Eristalis dimidiata

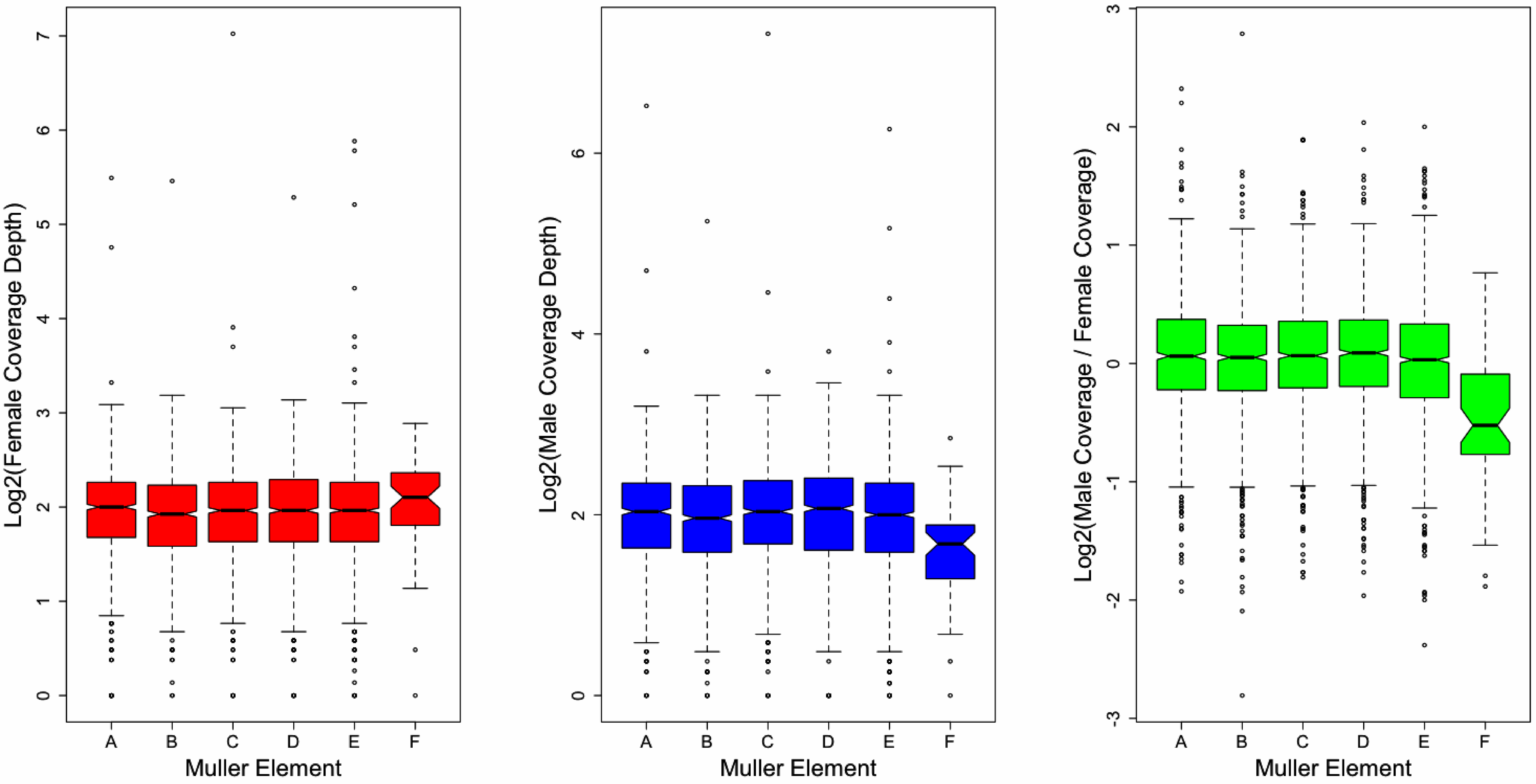

S1.16 Themira minor

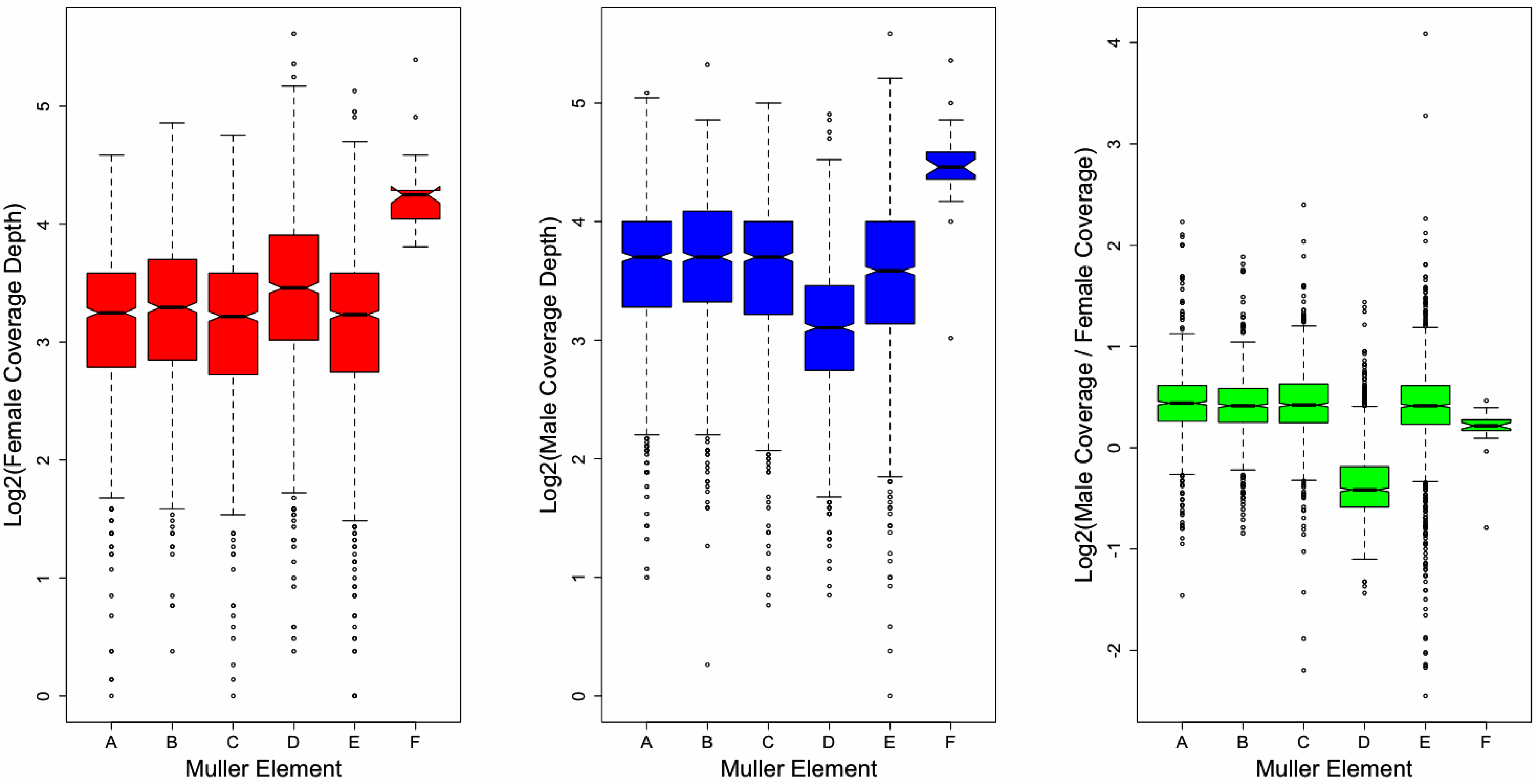

Figure S1

S1.17 Eutreta diana

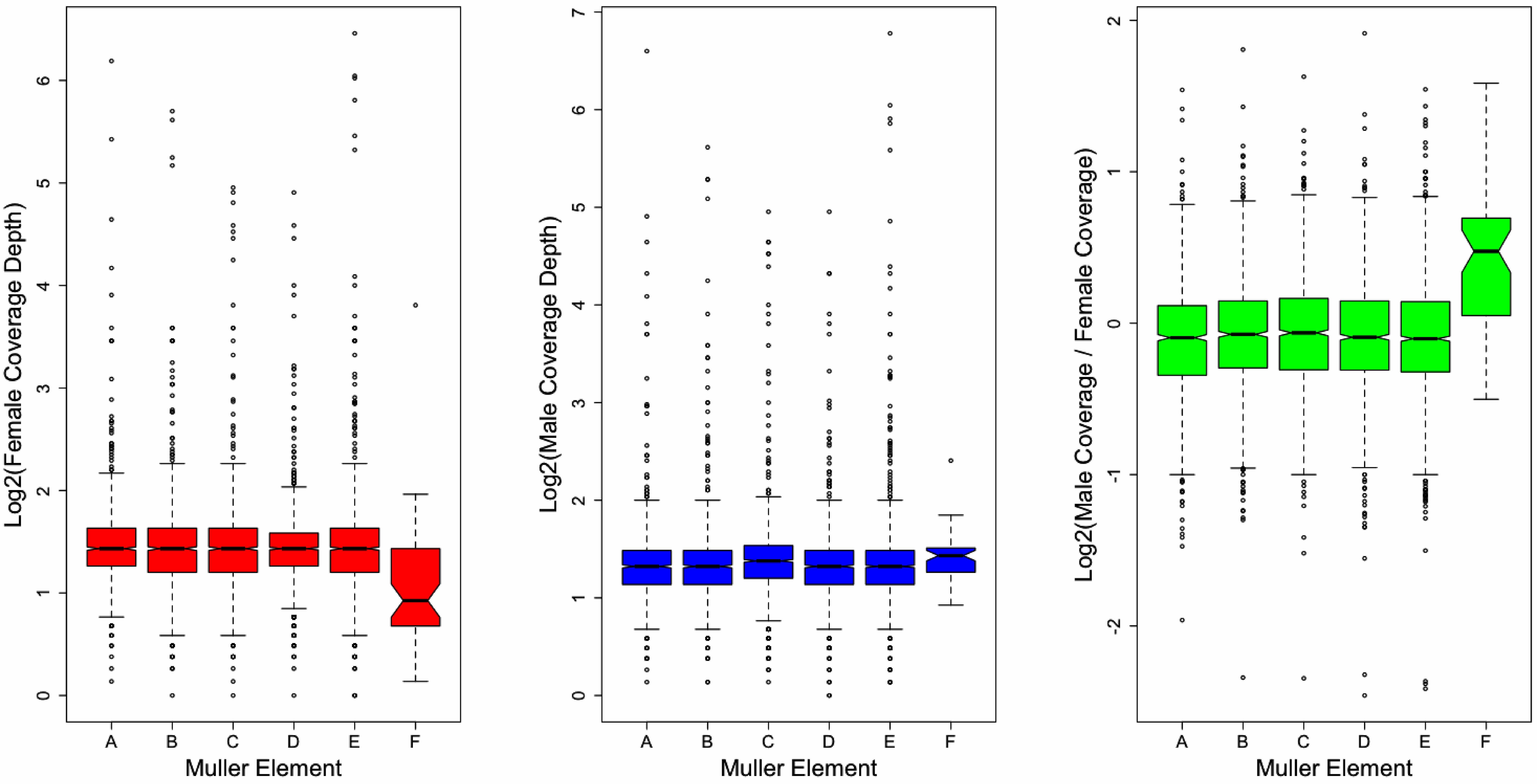

S1.18 Tephritis californica

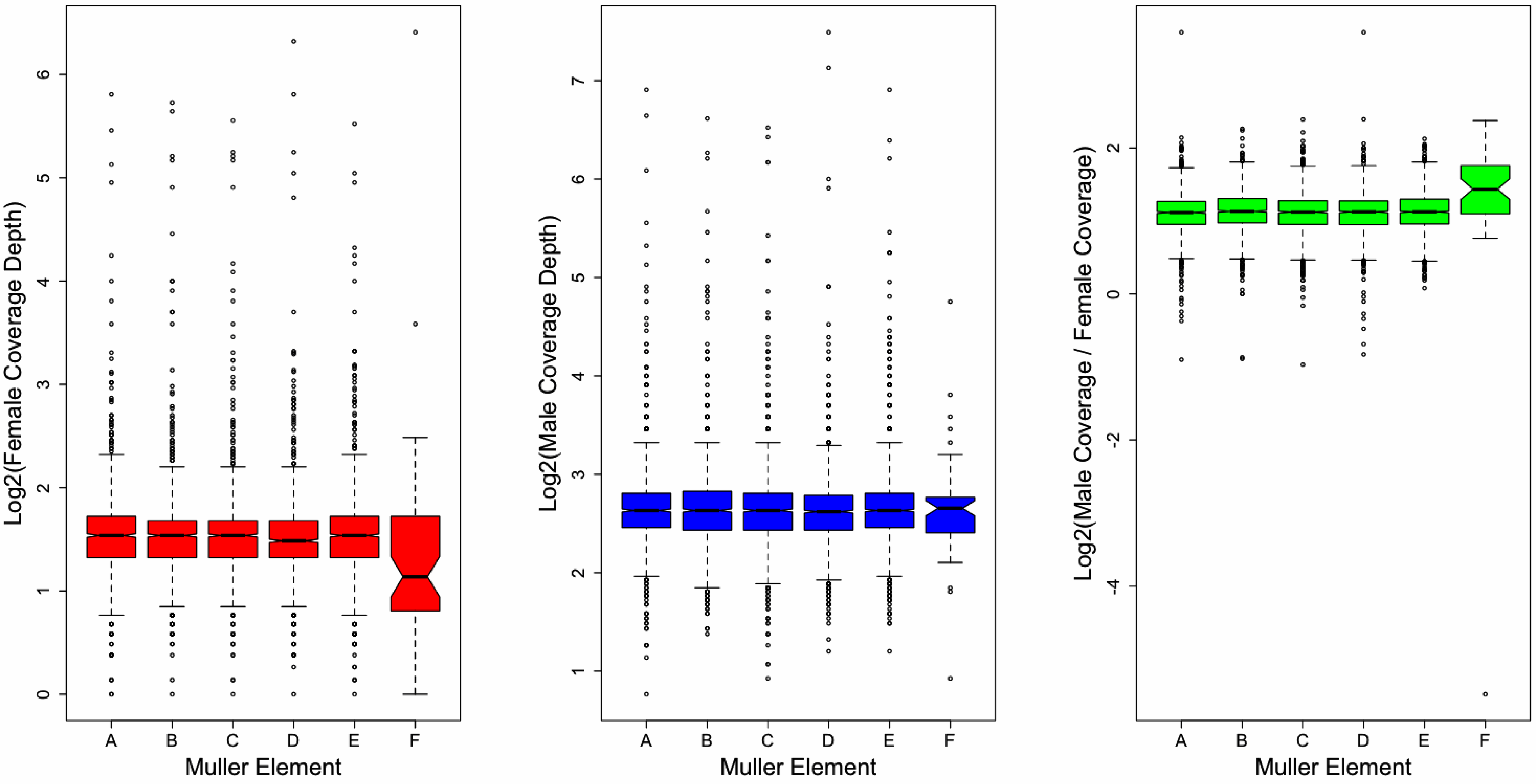

Figure S1

S1.19 Trupanea negronis

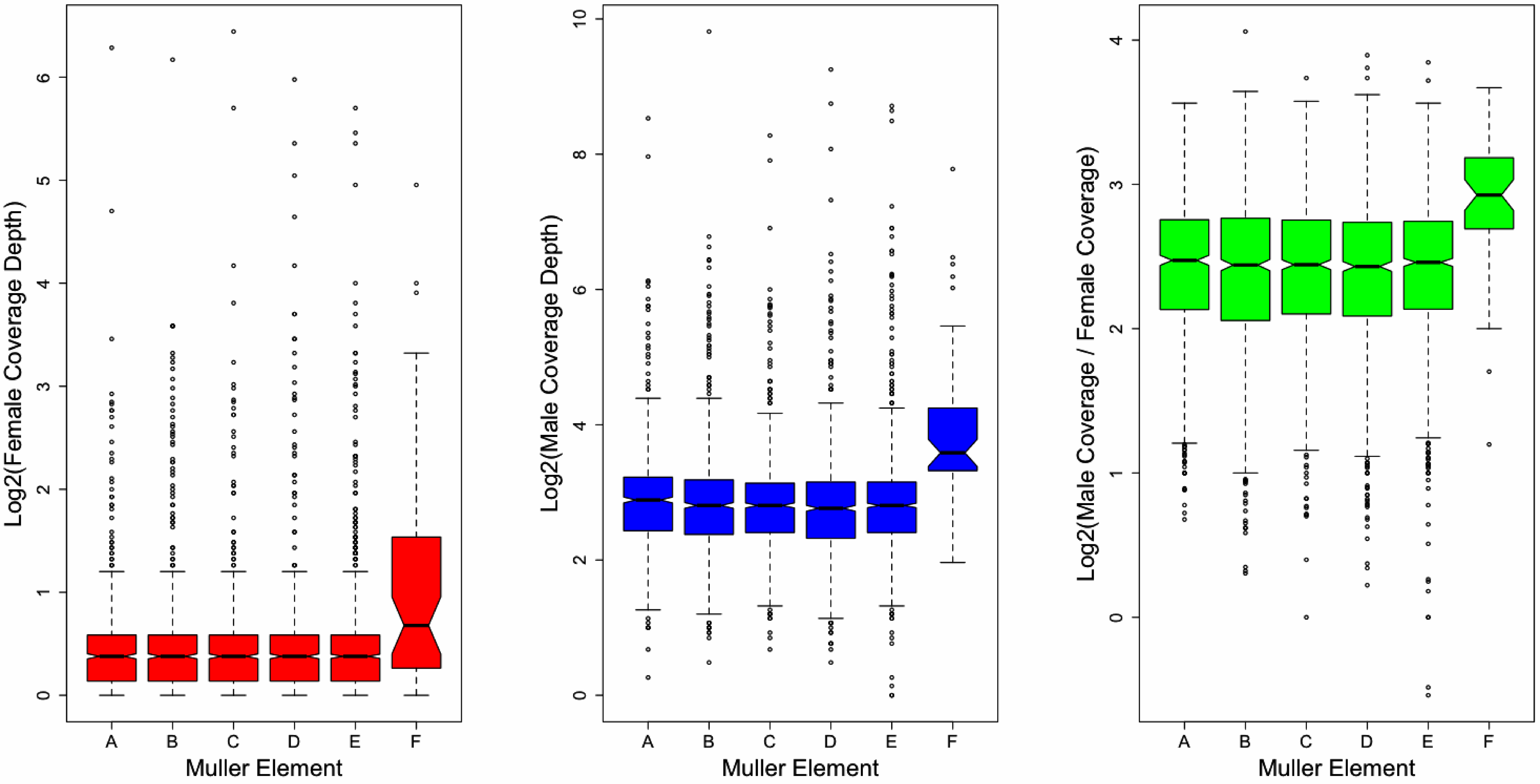

S1.20 Bactrocera oleae

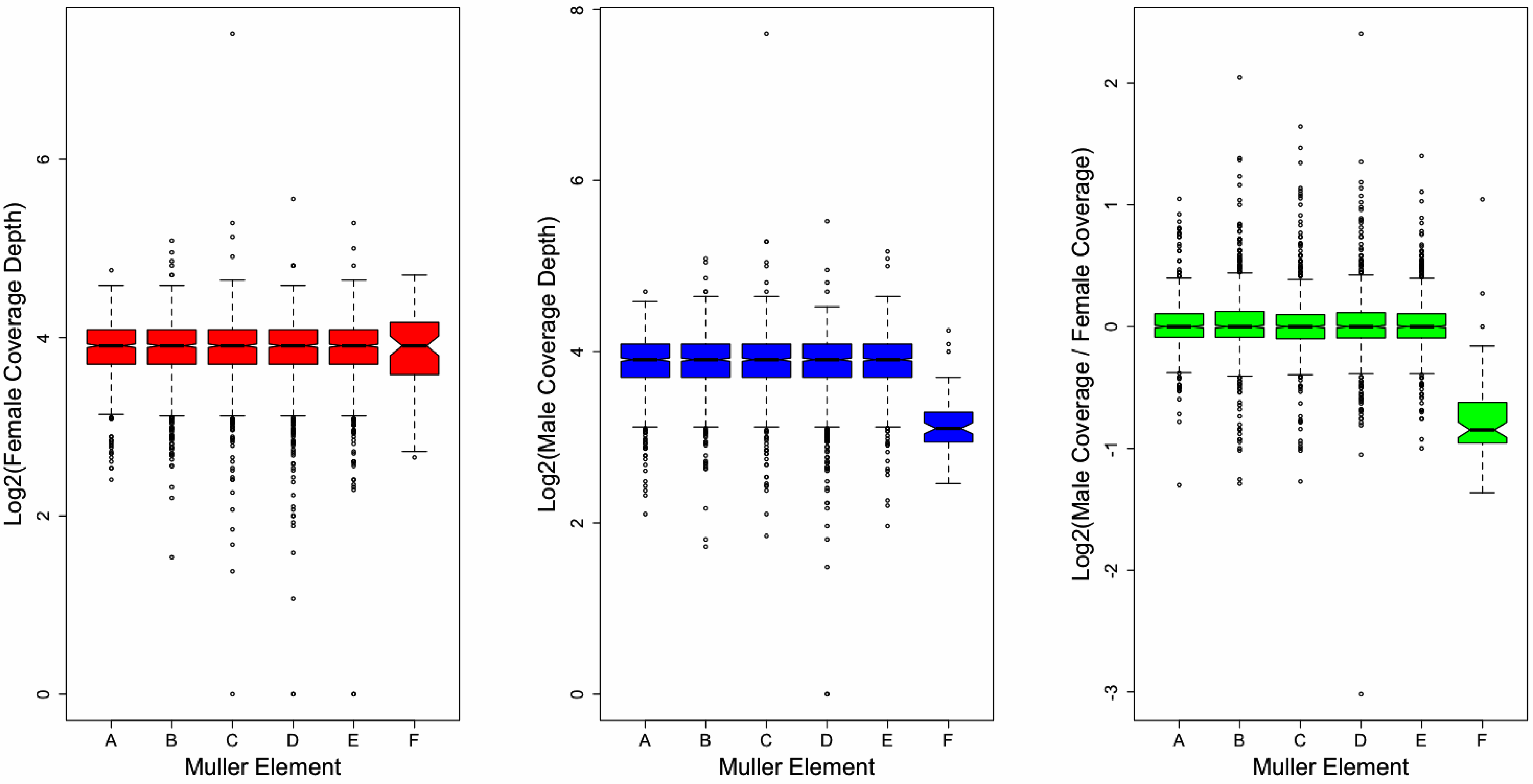

Figure S1

S1.21 Teleopsis dalmanni

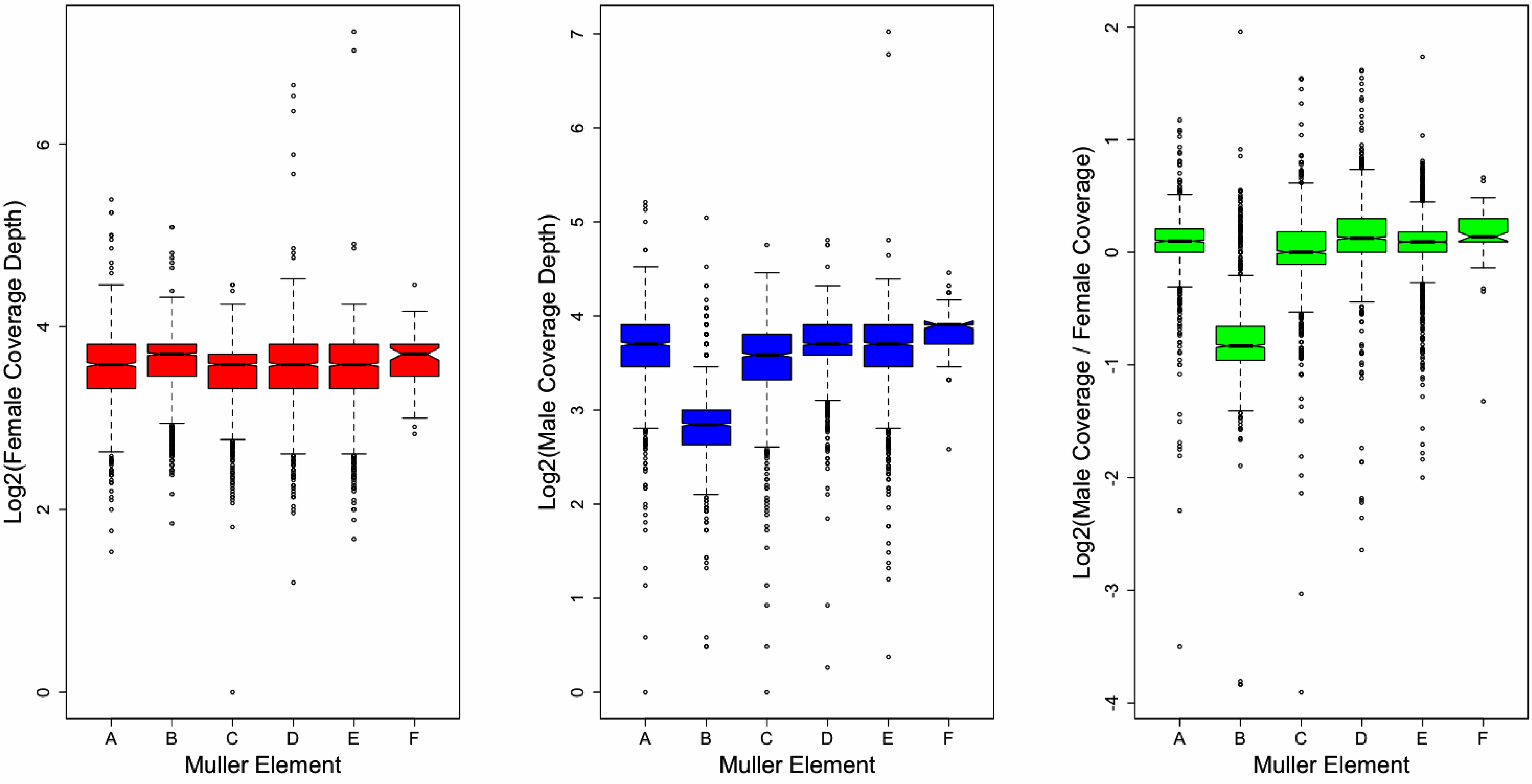

S1.22 Sphyracephala brevicornis

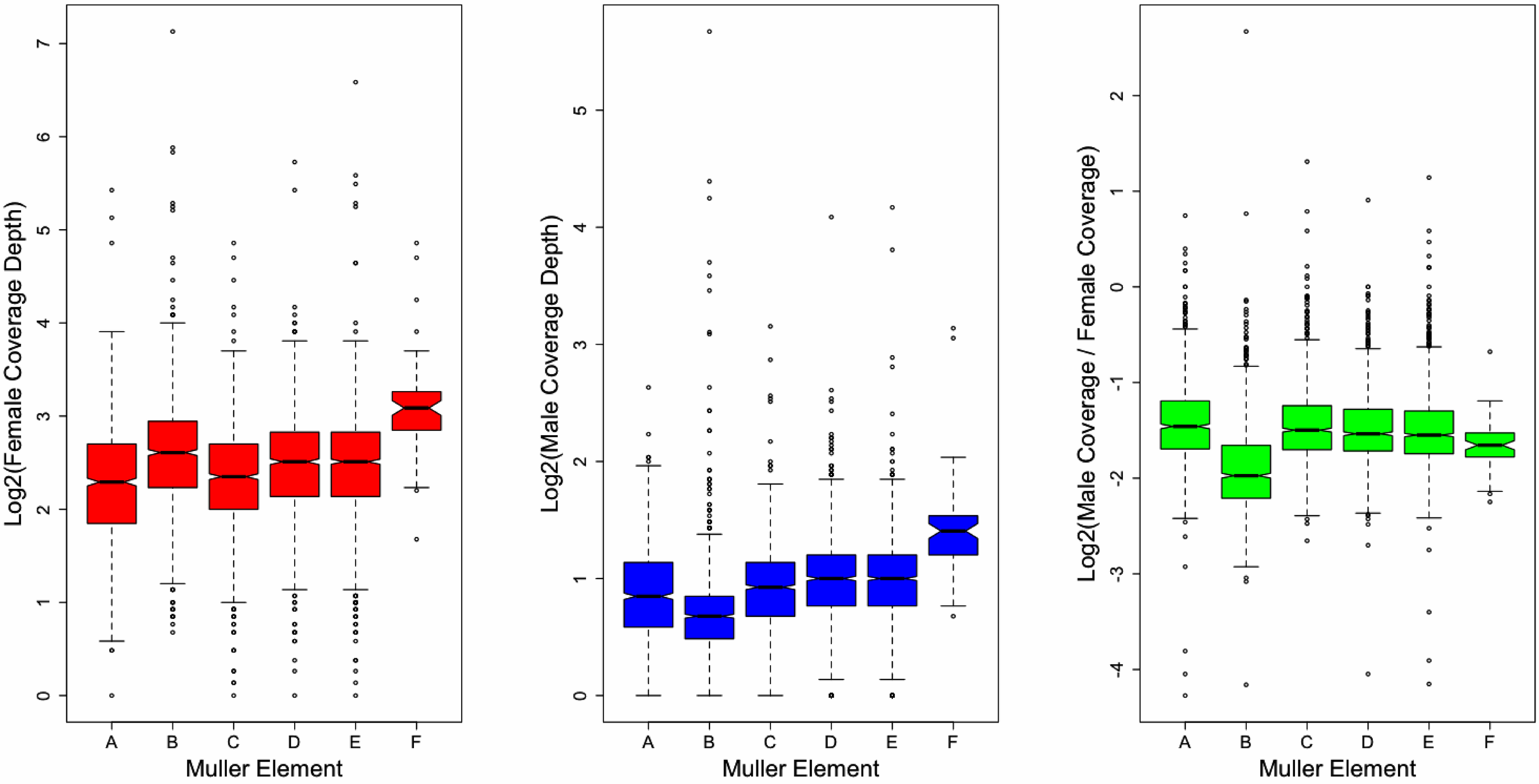

Figure S1

S1.23 Lyriomyza trifolii

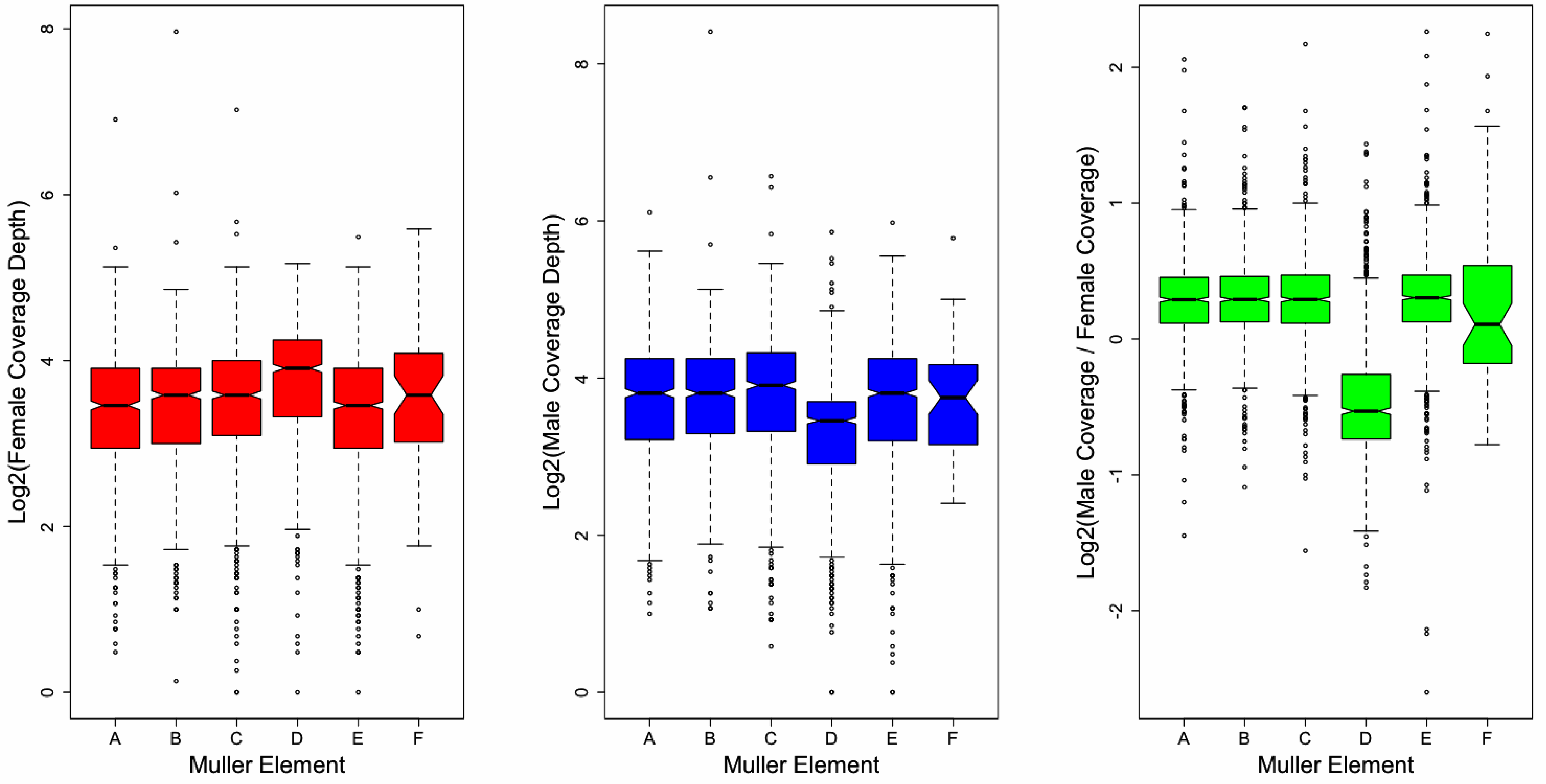

S1.24 Ephydra hians

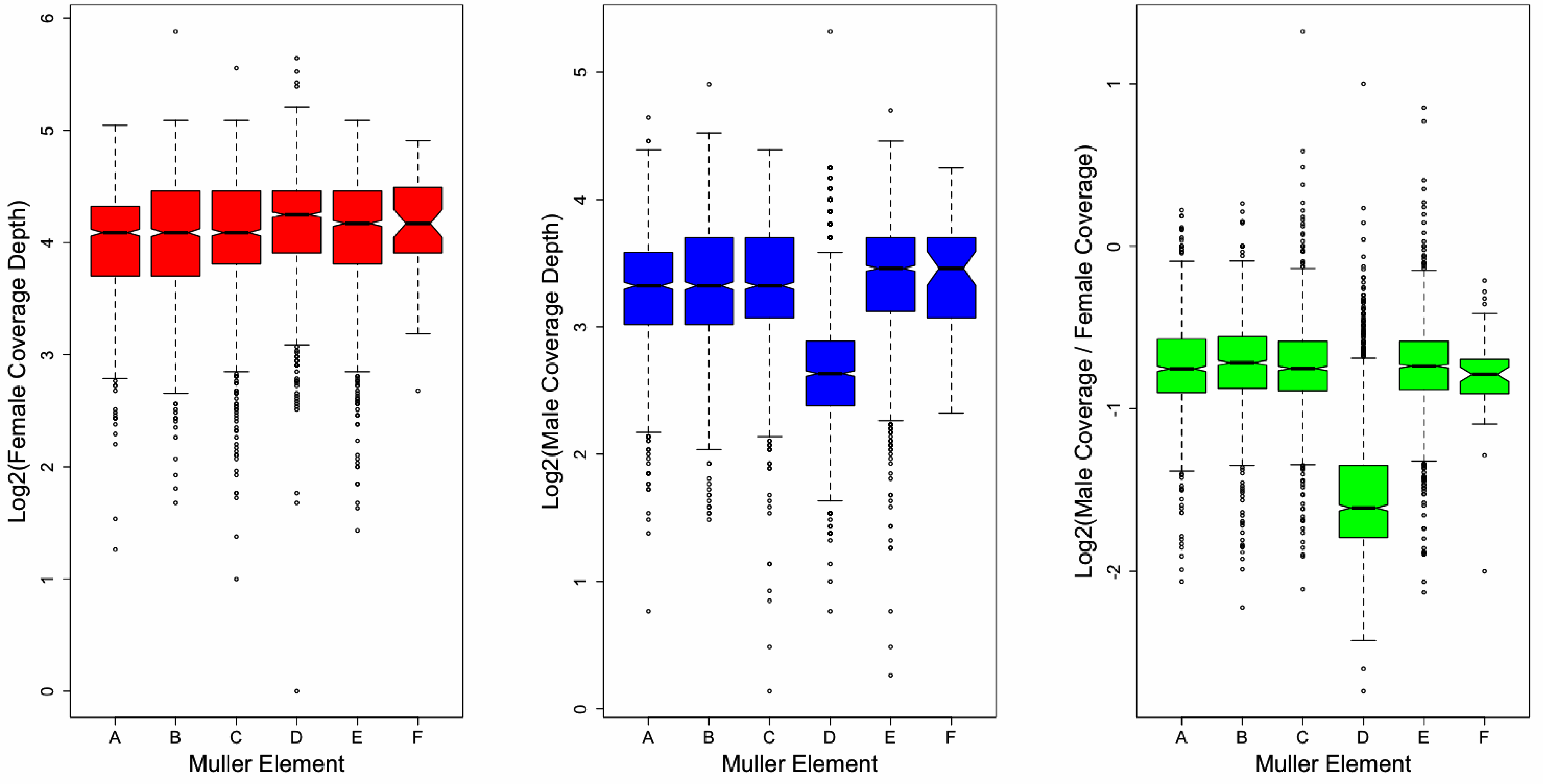

Figure S1

S1.25 Ephydra gracilis

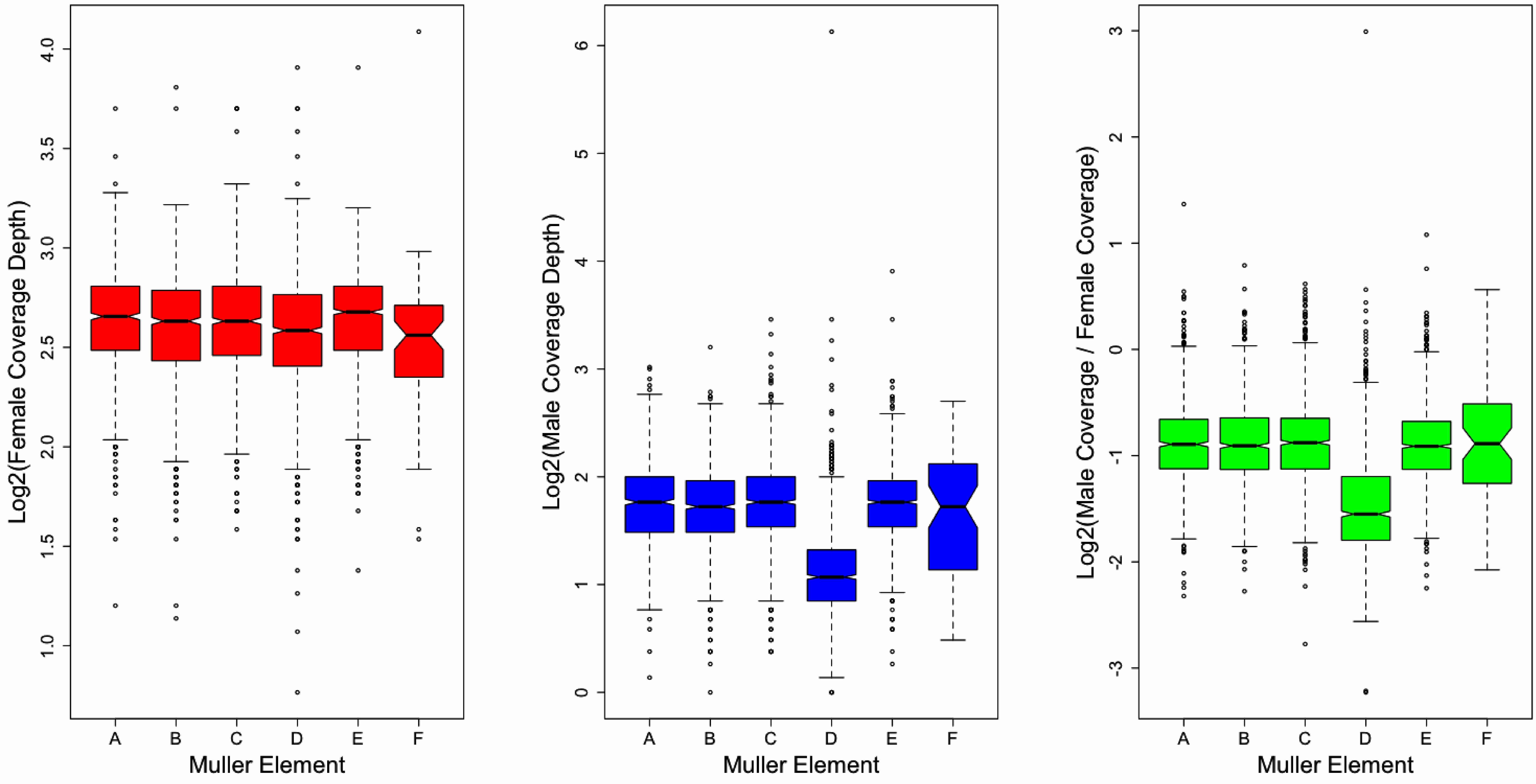

S1.26 Phortica variegata

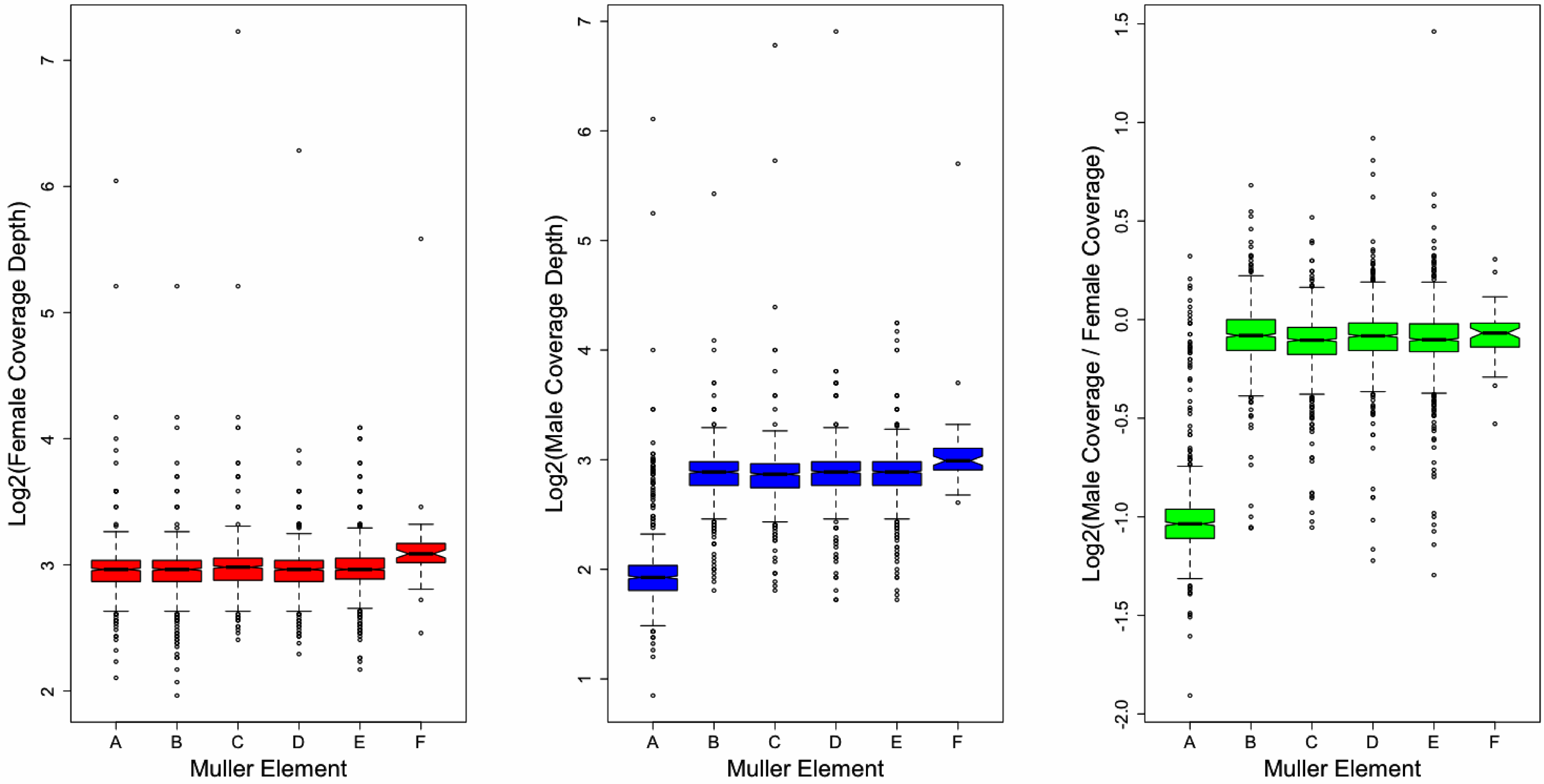

Figure S1

S1.27 Drosophila albomicans

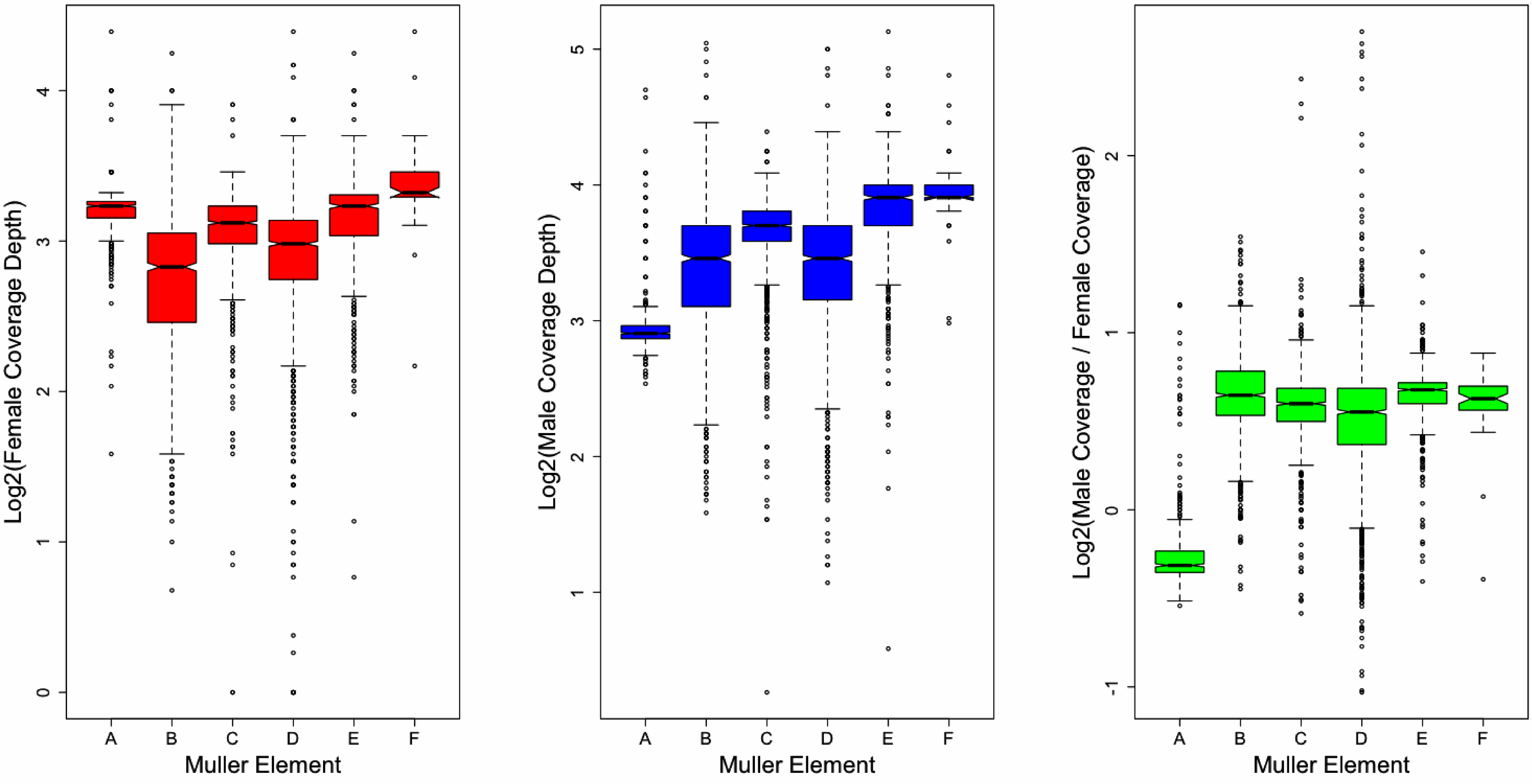

S1.28 Drosophila busckii

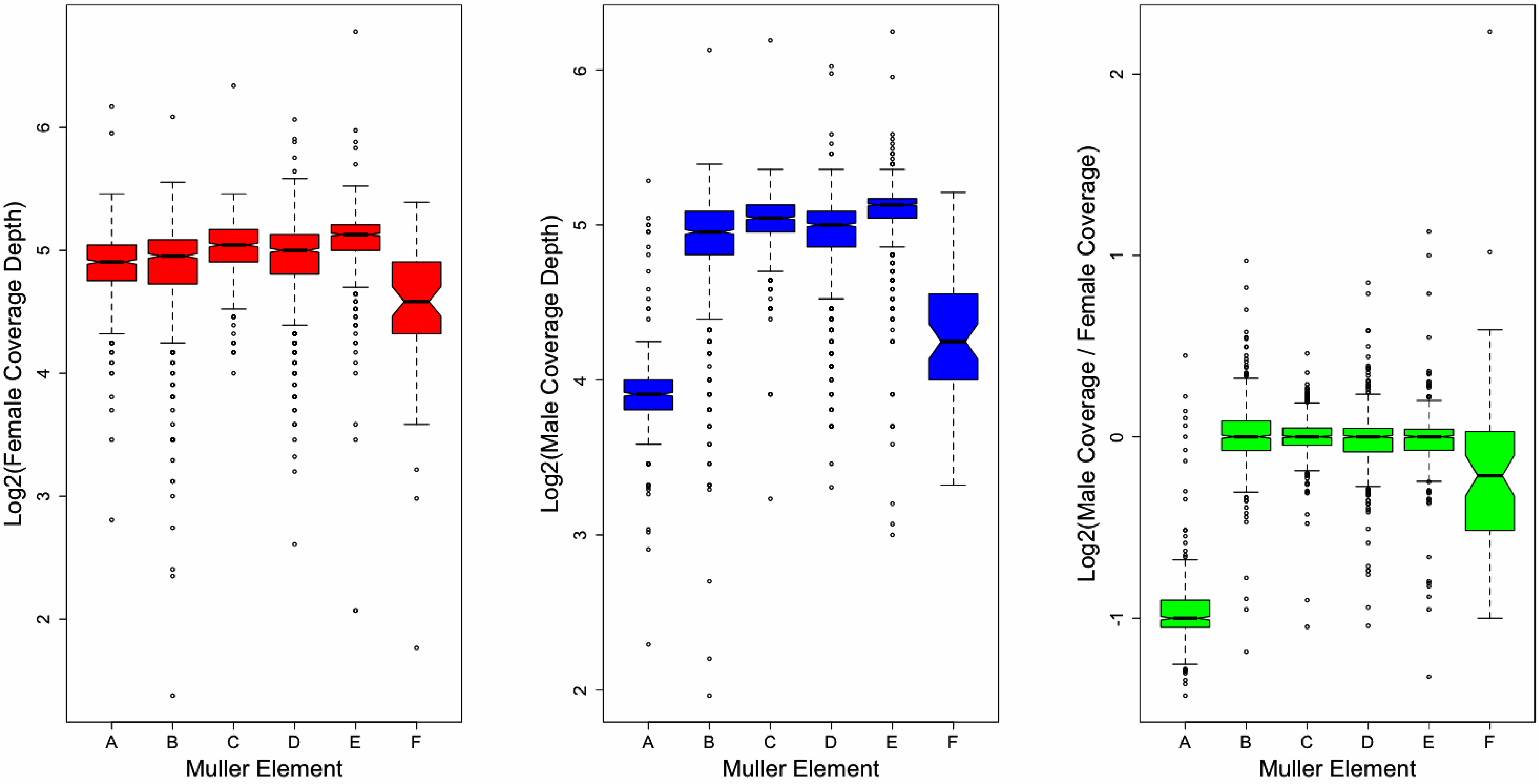

Figure S1

S1.29 Drosophila melanogaster

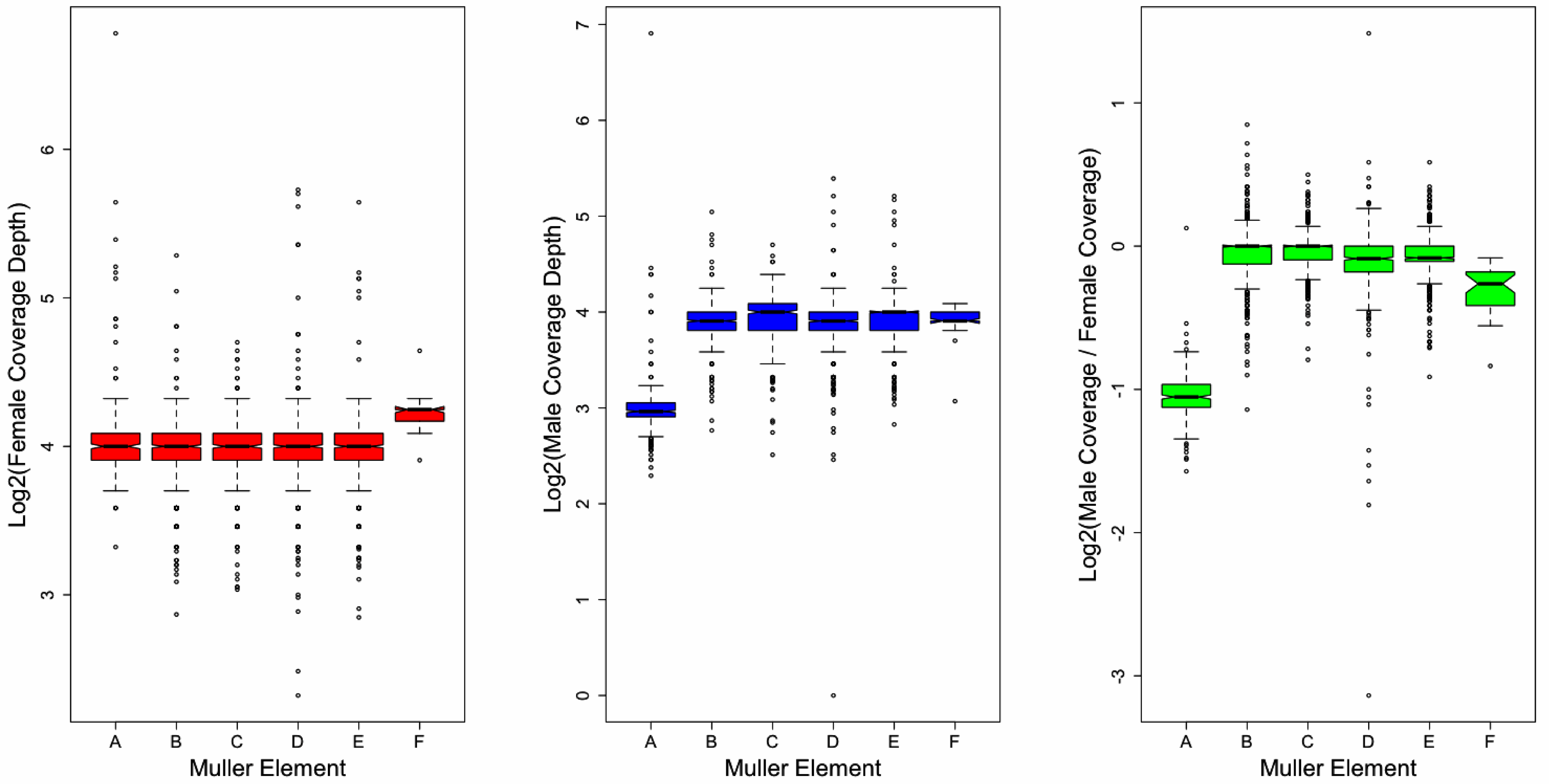

S1.30 Drosophila miranda

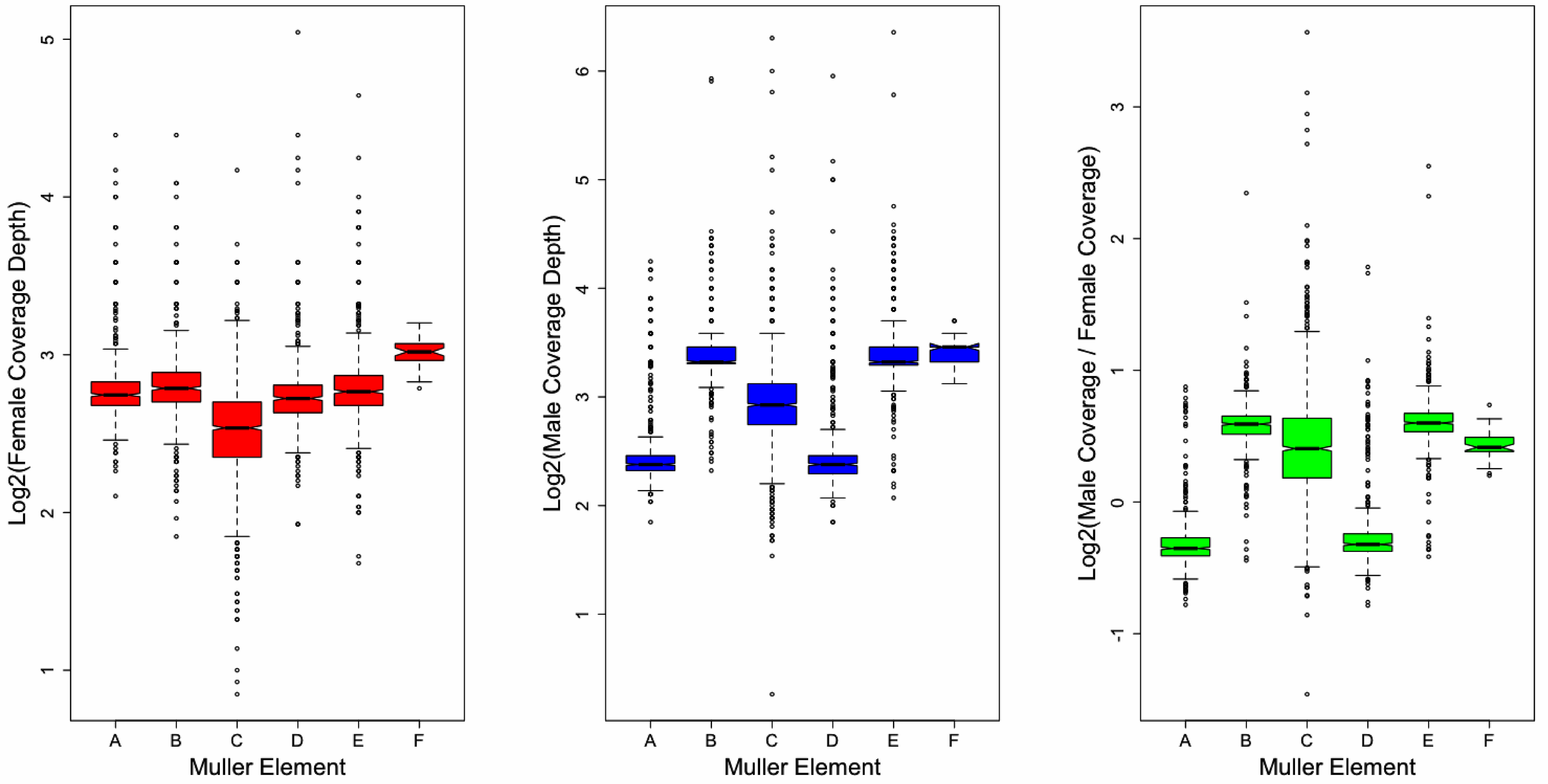

Figure S1

S1.31 Drosophila pseudoobscura

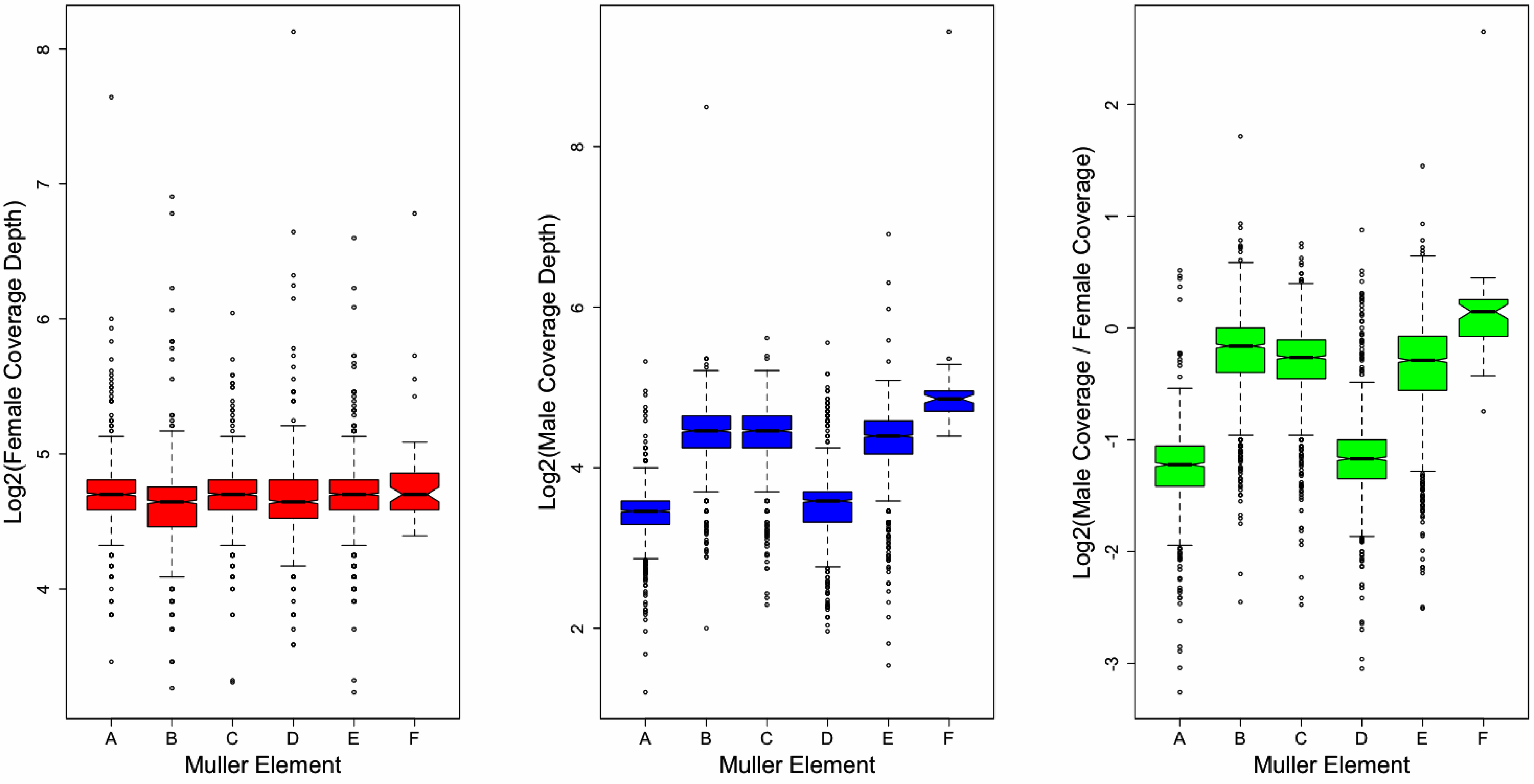

S1.32 Scaptodrosophila lebanonensis

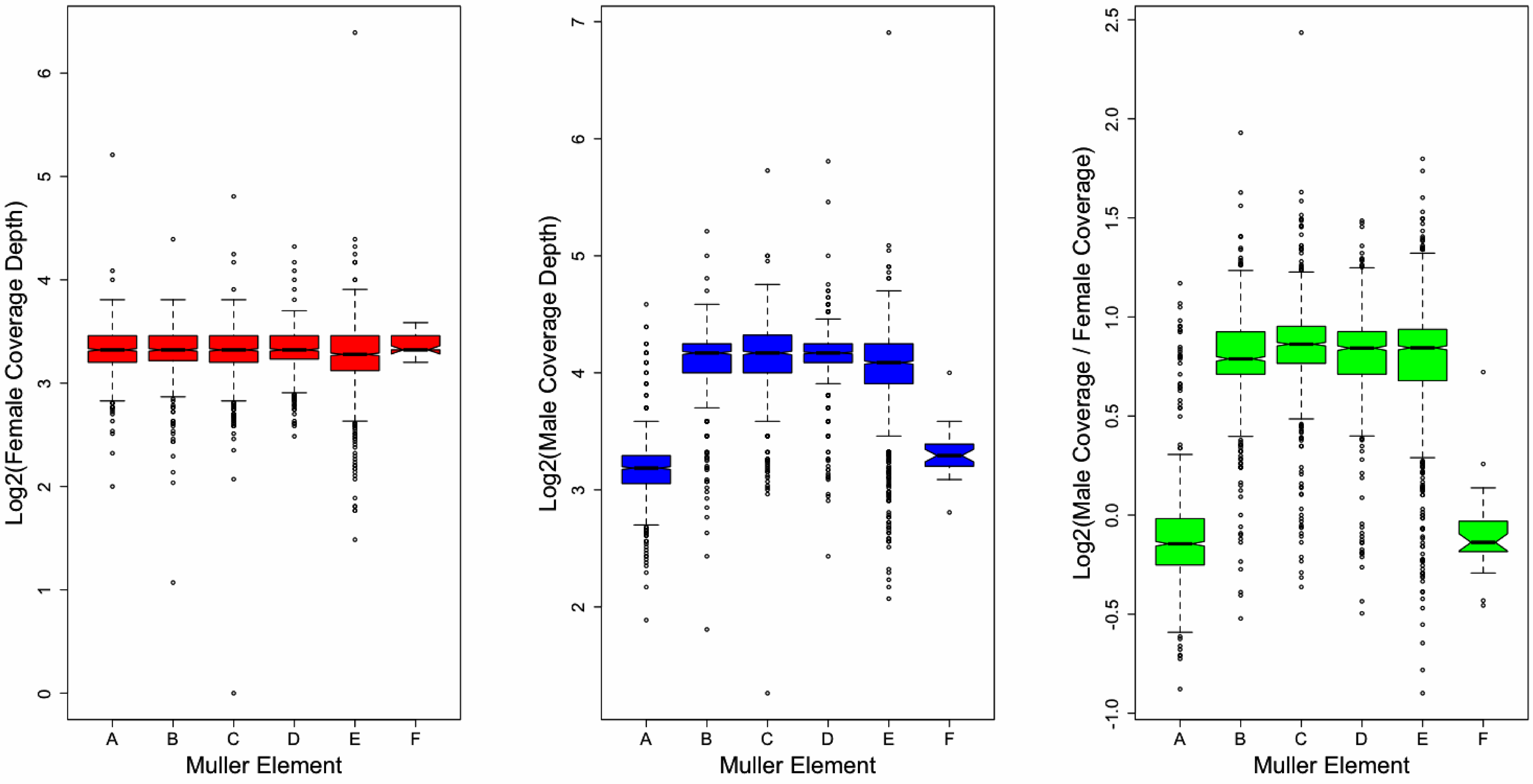

Figure S1

S1.33 Glossina morsitans

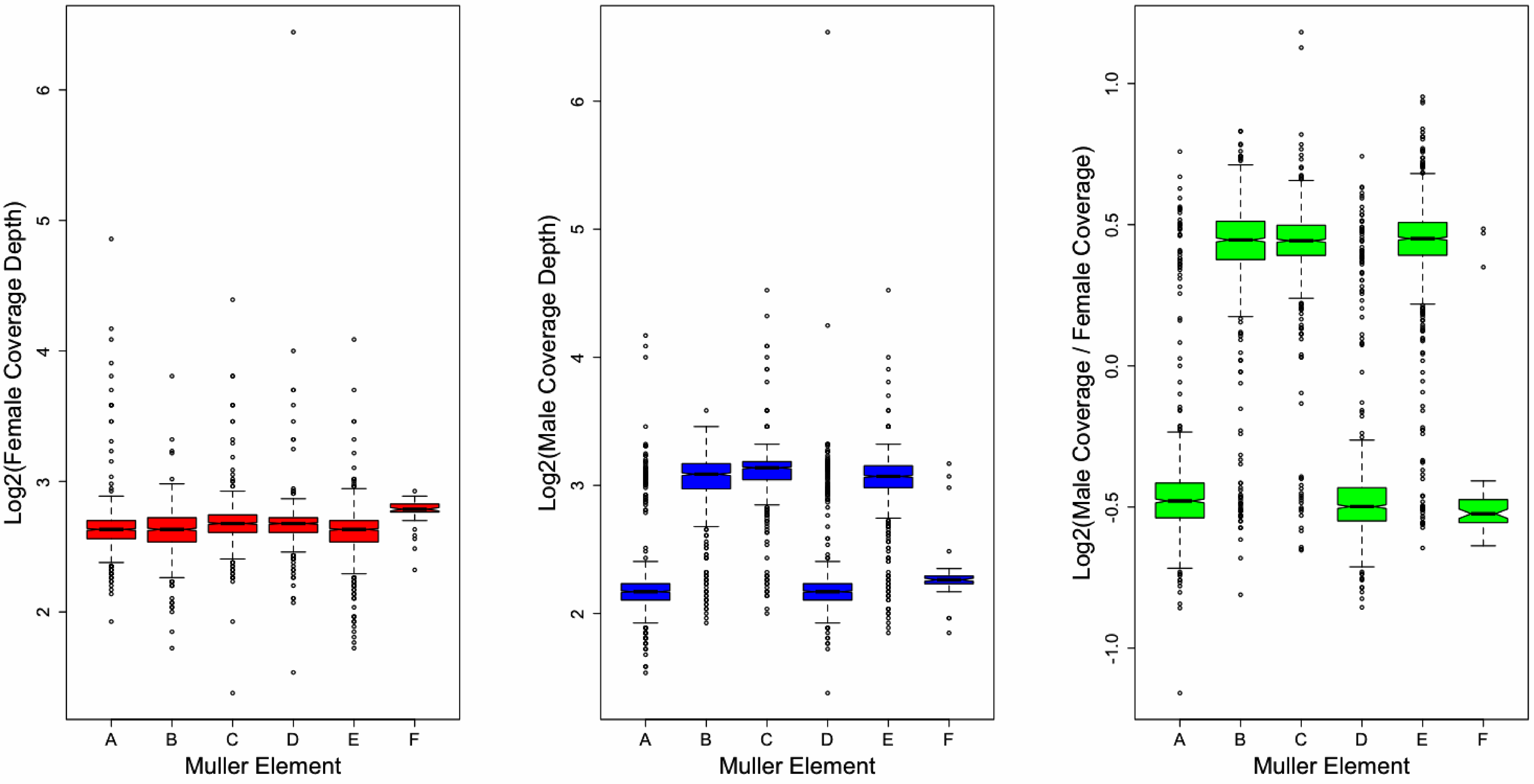

S1.34 Sarcophaga bullata

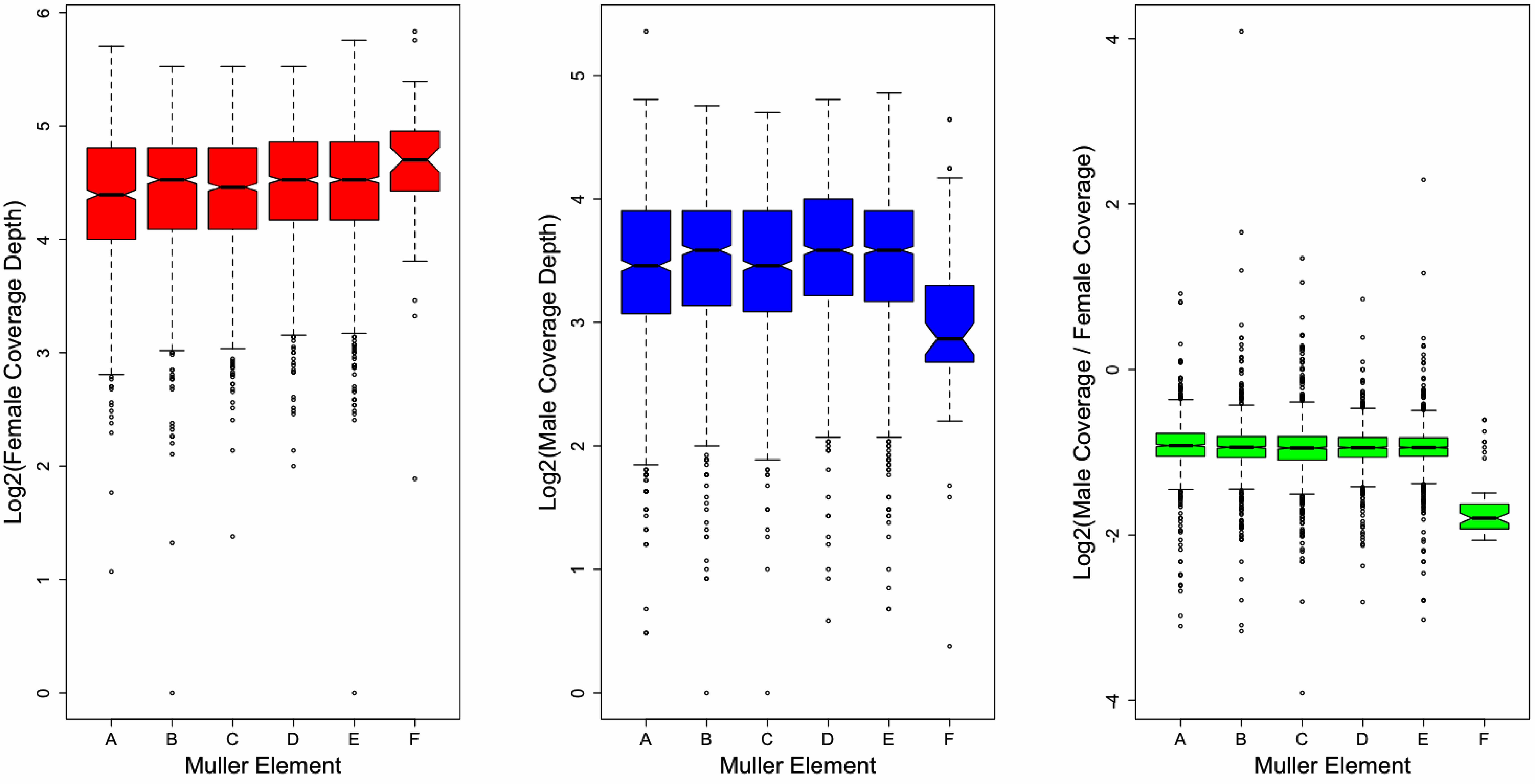

Figure S1

S1.35 Sarcophagidae sp.

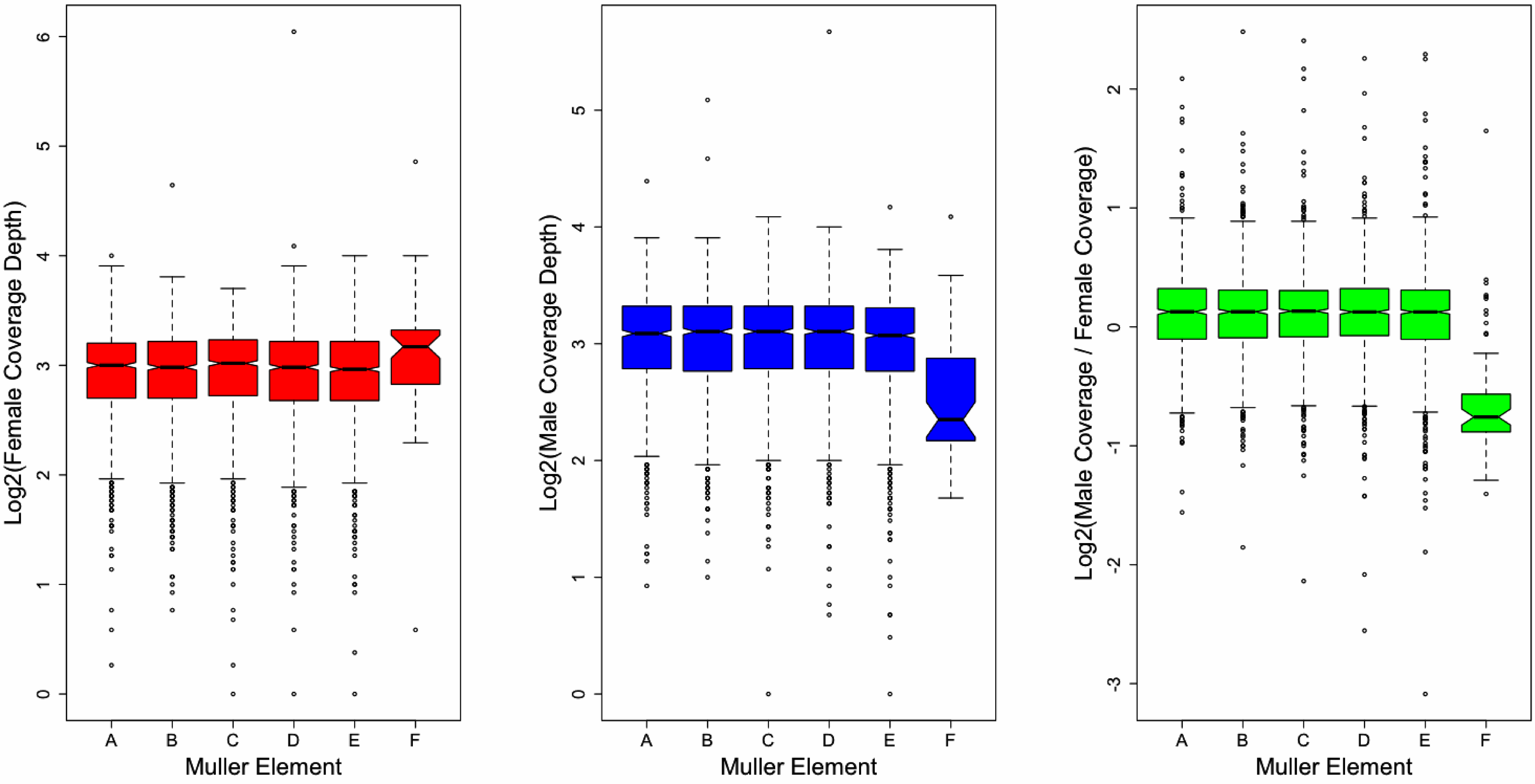

S1.36 Lucilia sericata

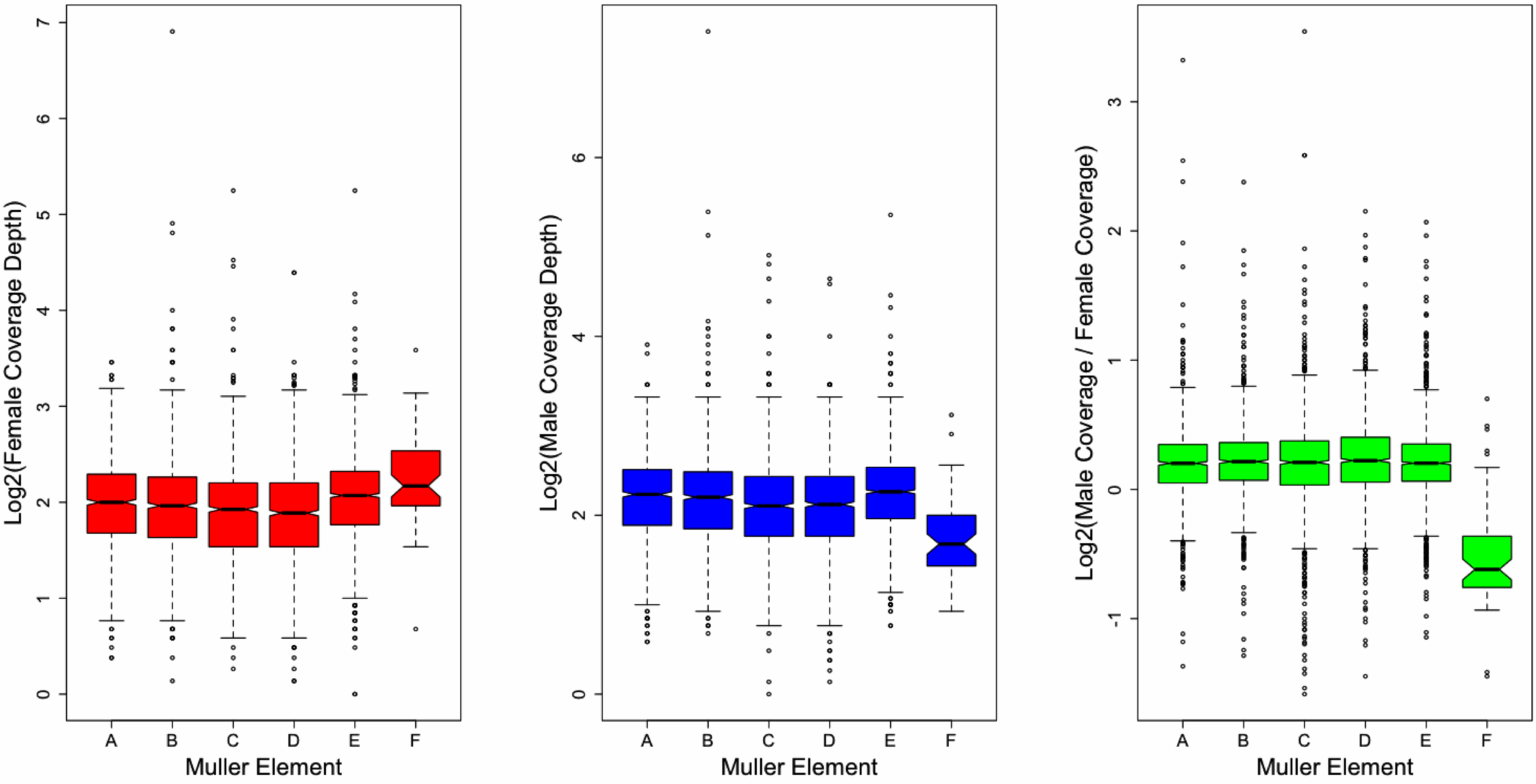

Figure S1

S1.37 Calliphora erythrocephala

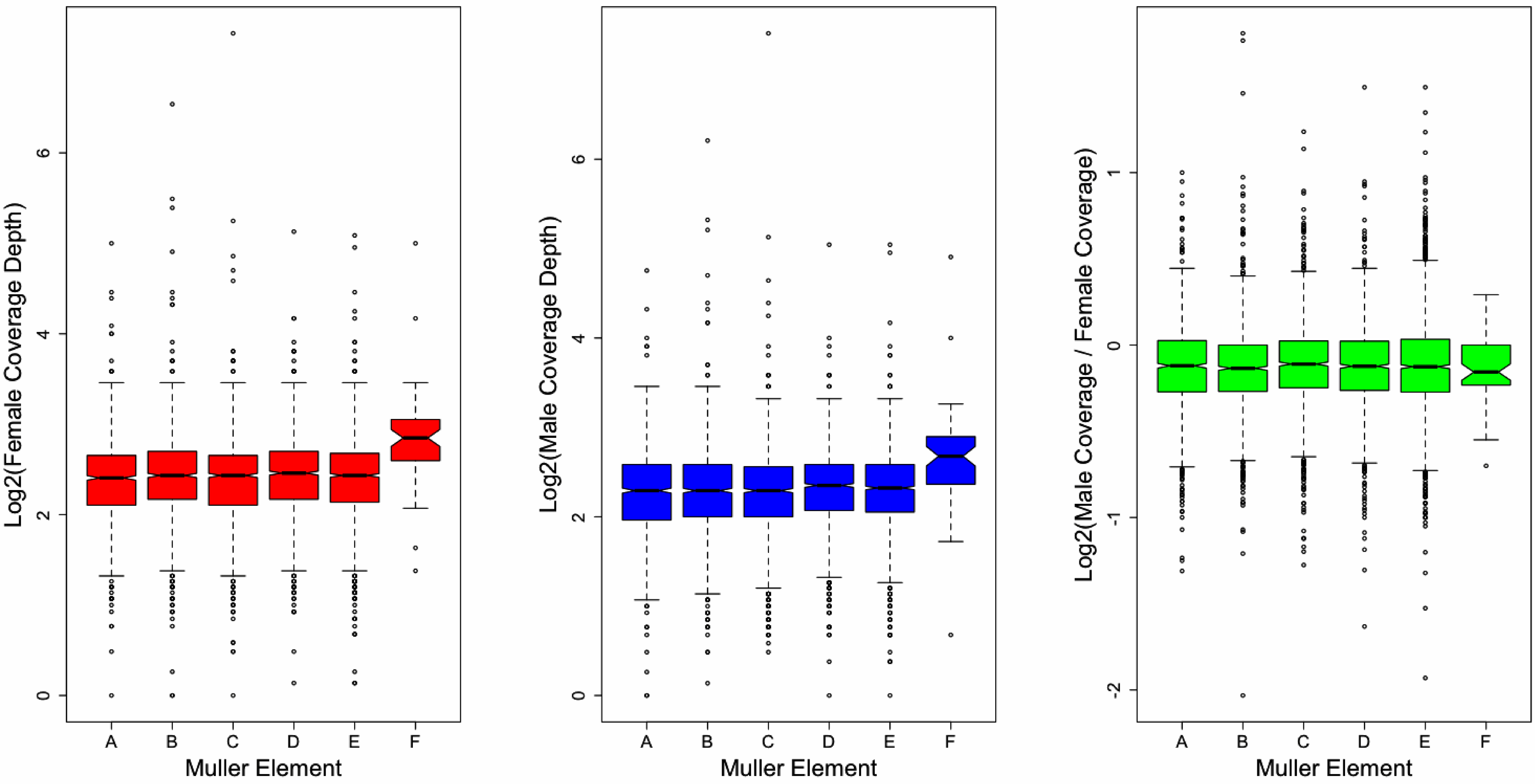

Figure S1
