# Supplementary material for: Numerous Transitions of Sex Chromosomes in Diptera
Source: PLoS Biol. 2015 Apr 16;13(4):e1002078. doi: 10.1371/journal.pbio.1002078 (PMC4400102; doi:10.1371/journal.pbio.1002078)
Supplement: S2 Fig — Shown is Log2 of Female (in red), Male (in blue), and M/F (in green) coverage for each Muller element for each Nematocera species investigated. Data to generate this graph are to be found in file “S1 Data.” (PDF) [file pbio.1002078.s007.pdf]

S2.1 Tipula olearacea

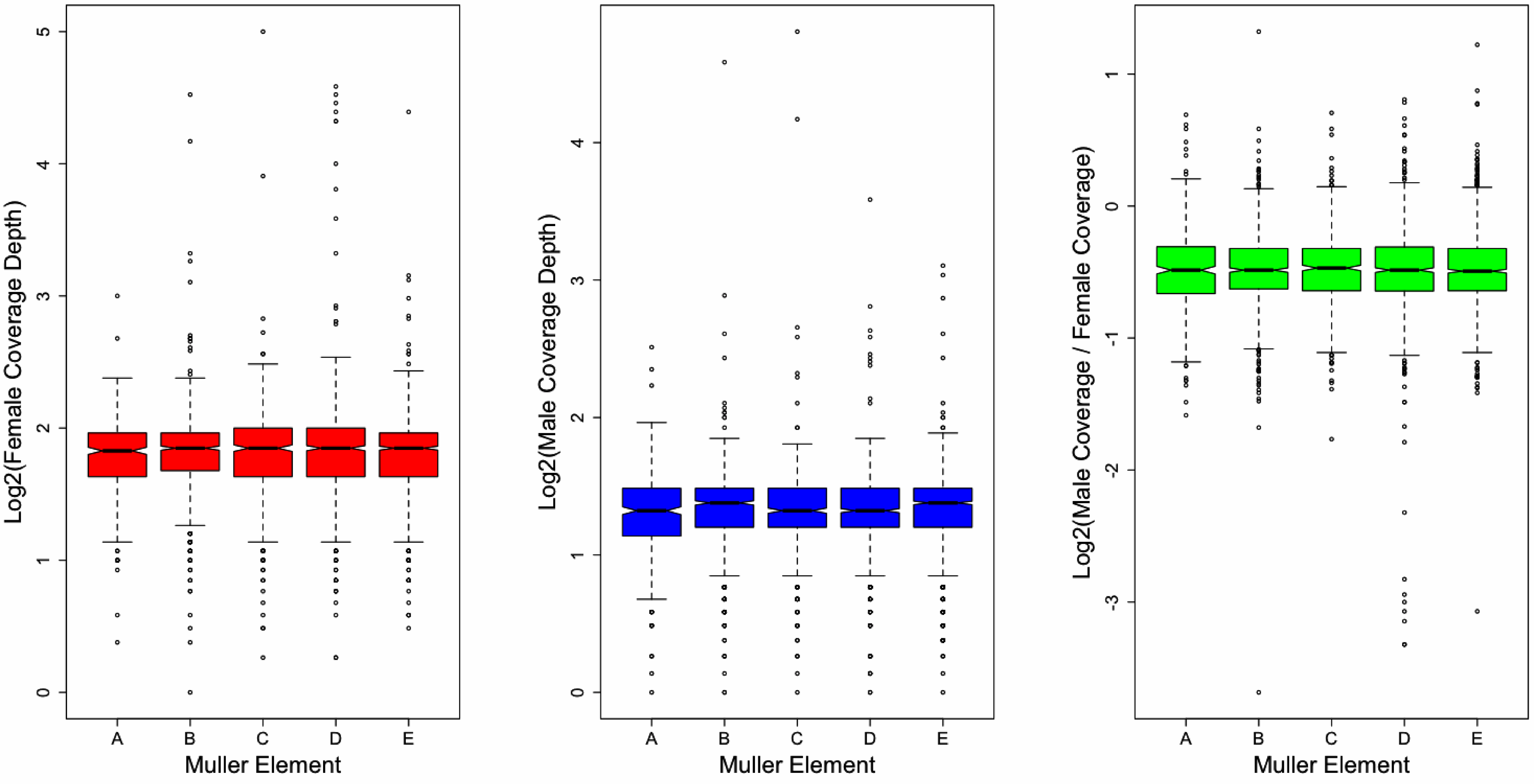

S2.2 Trichoceridae sp

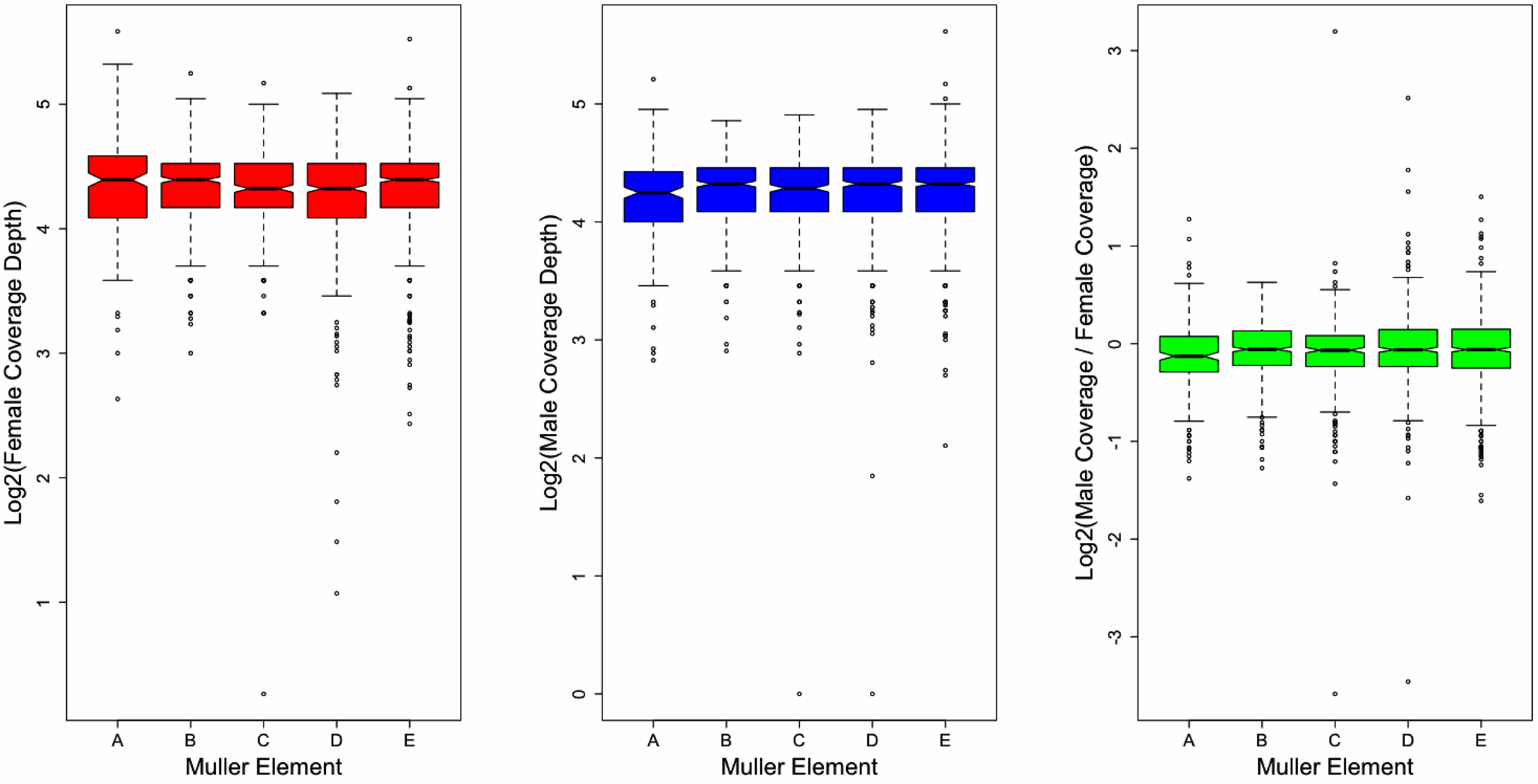

Figure S2

S2.3 Clogmia albipunctata

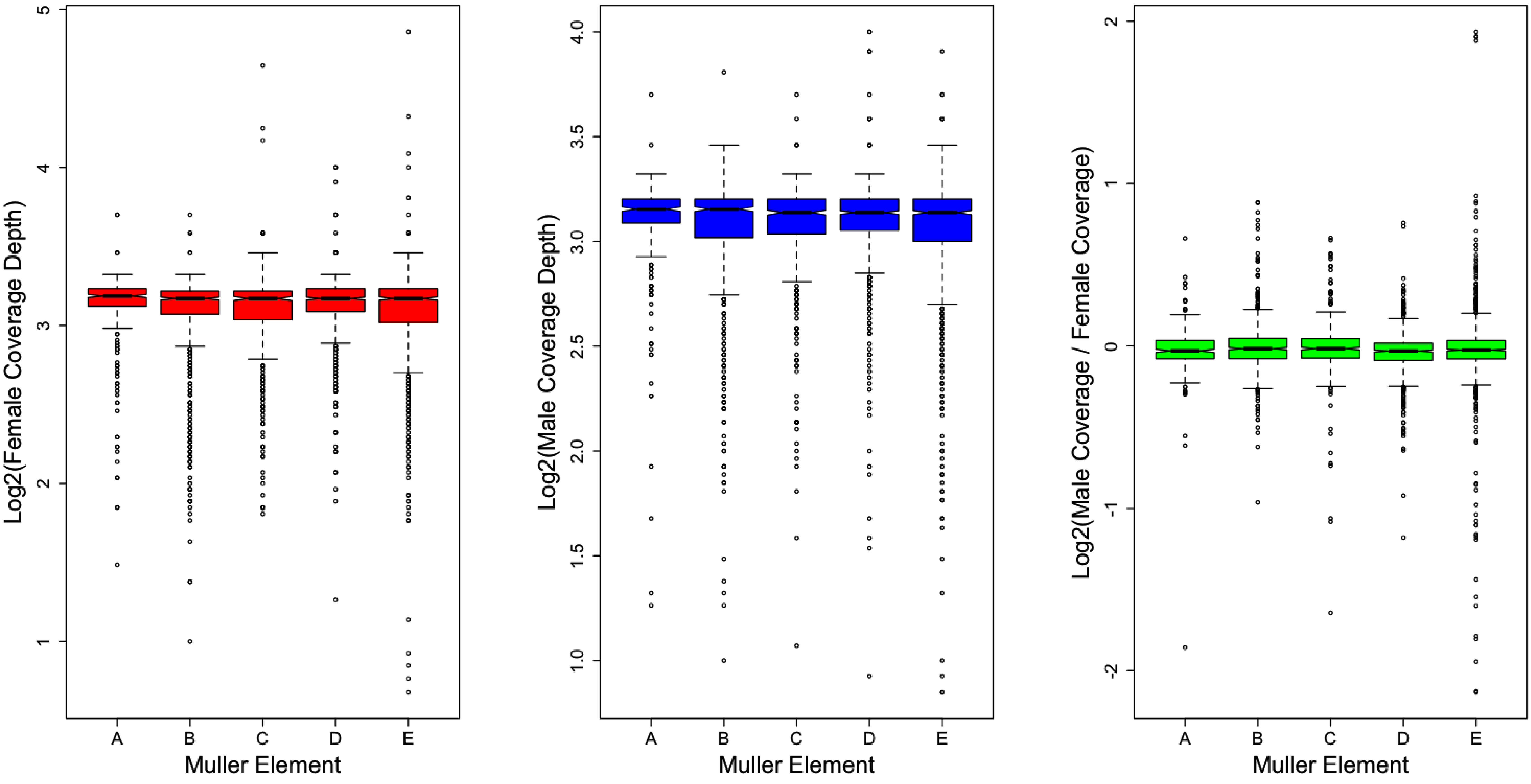

S2.4 Chironomus riparius

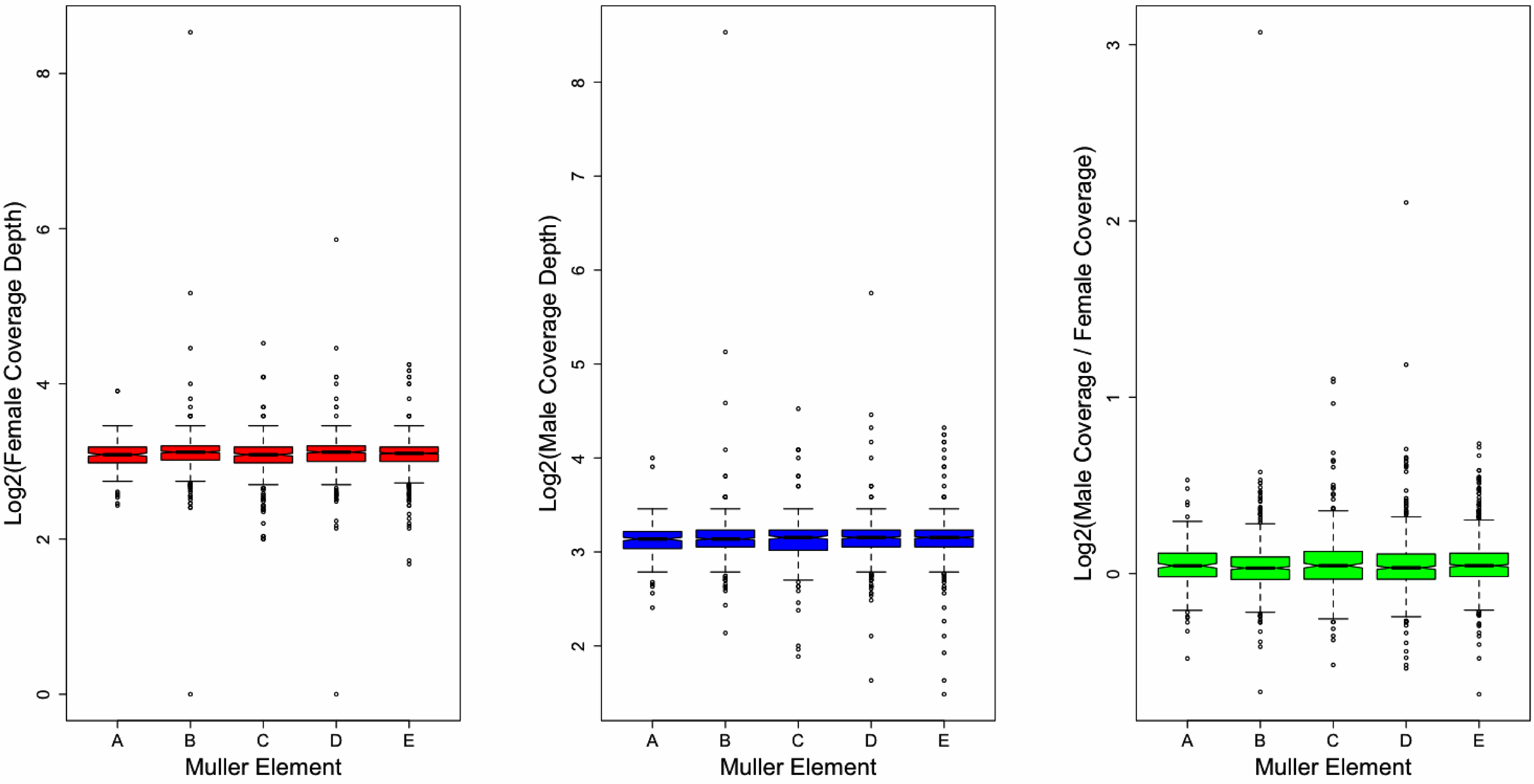

Figure S2

S2.5 Chaoborus trivittatus

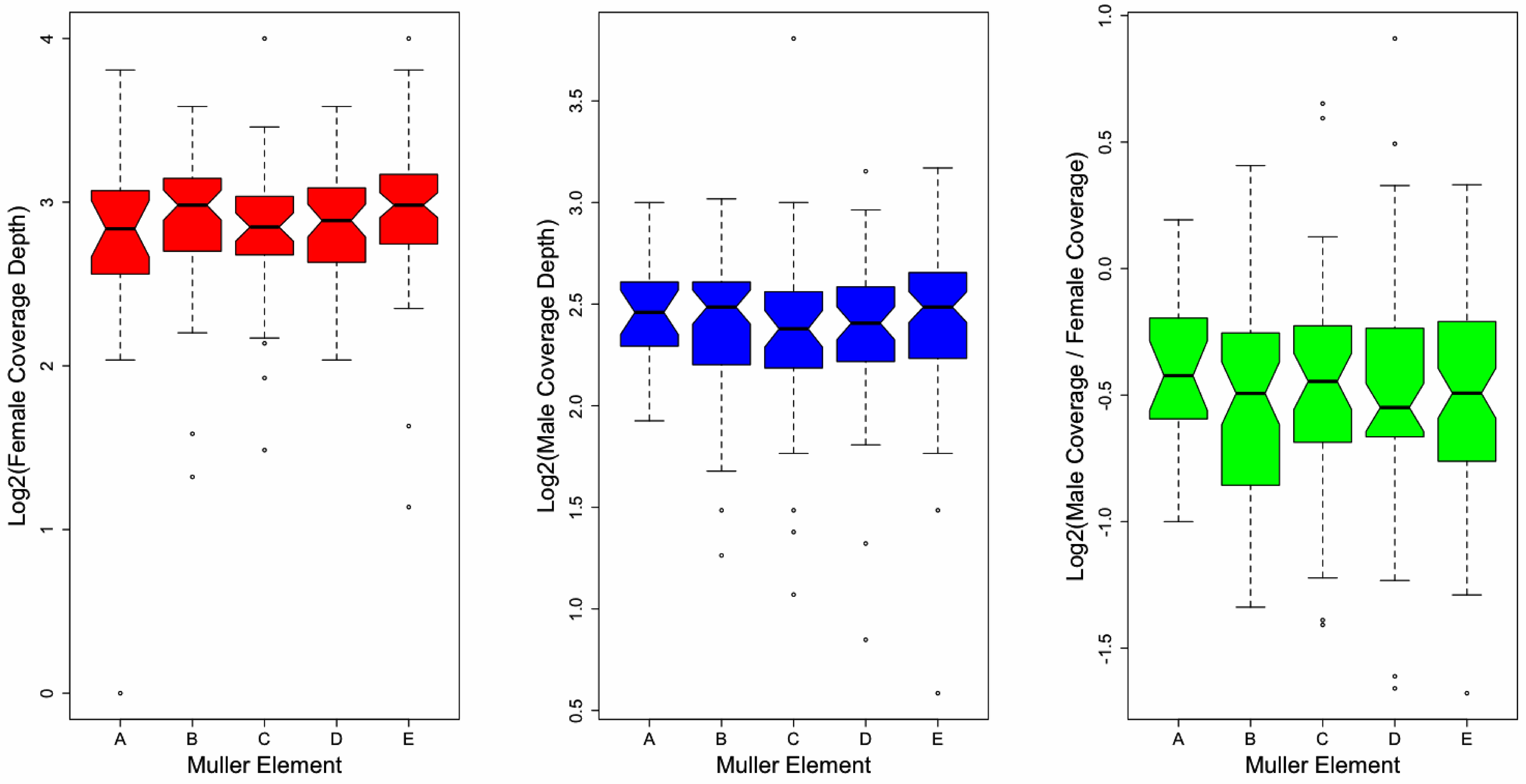

S2.6 Monchlonyx cinctipes

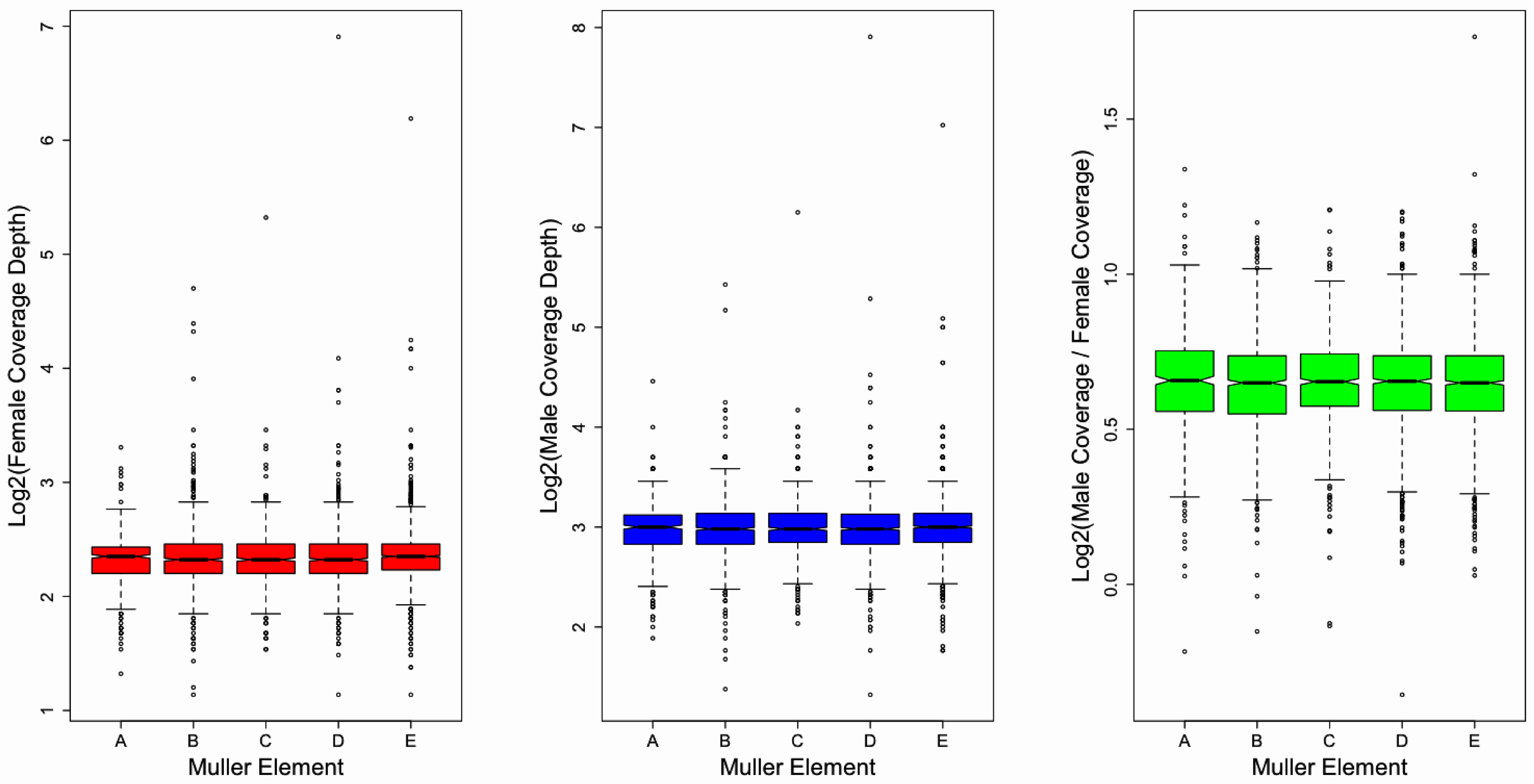

Figure S2

S2.7 Anopheles gambiae

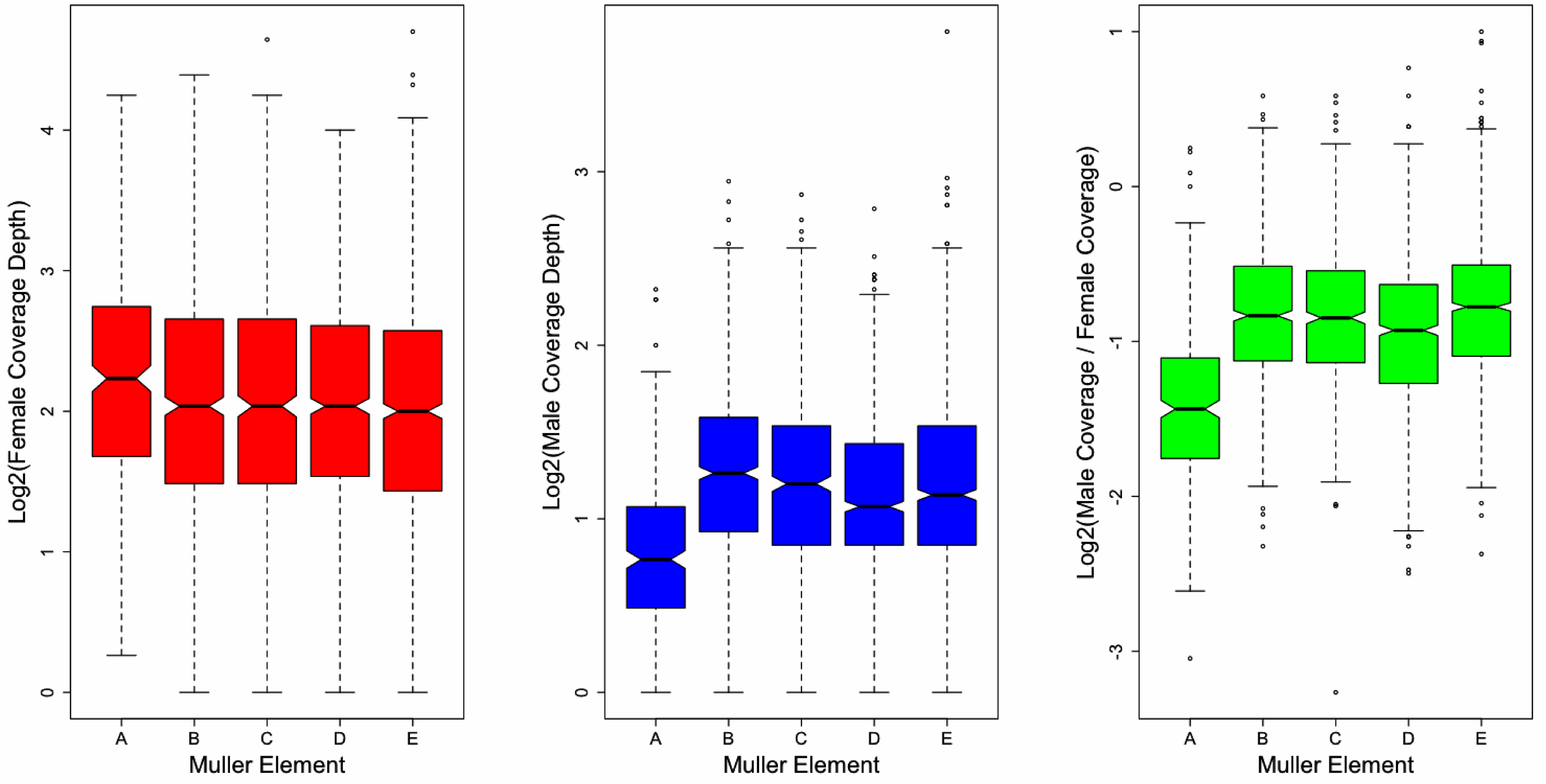

S2.8 Aedes aegyptii

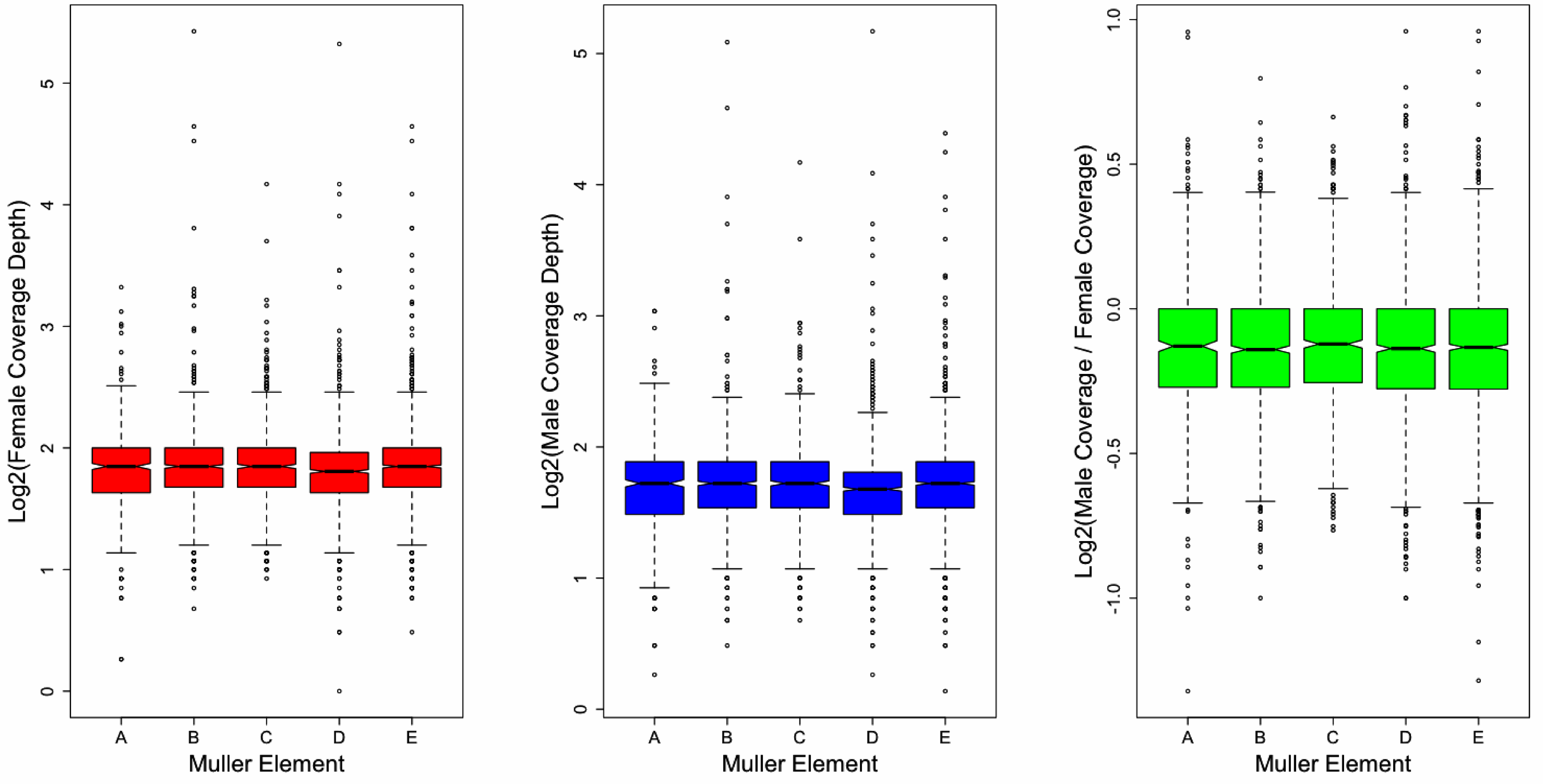

Figure S2

S2.9 Coboldia fuscipes

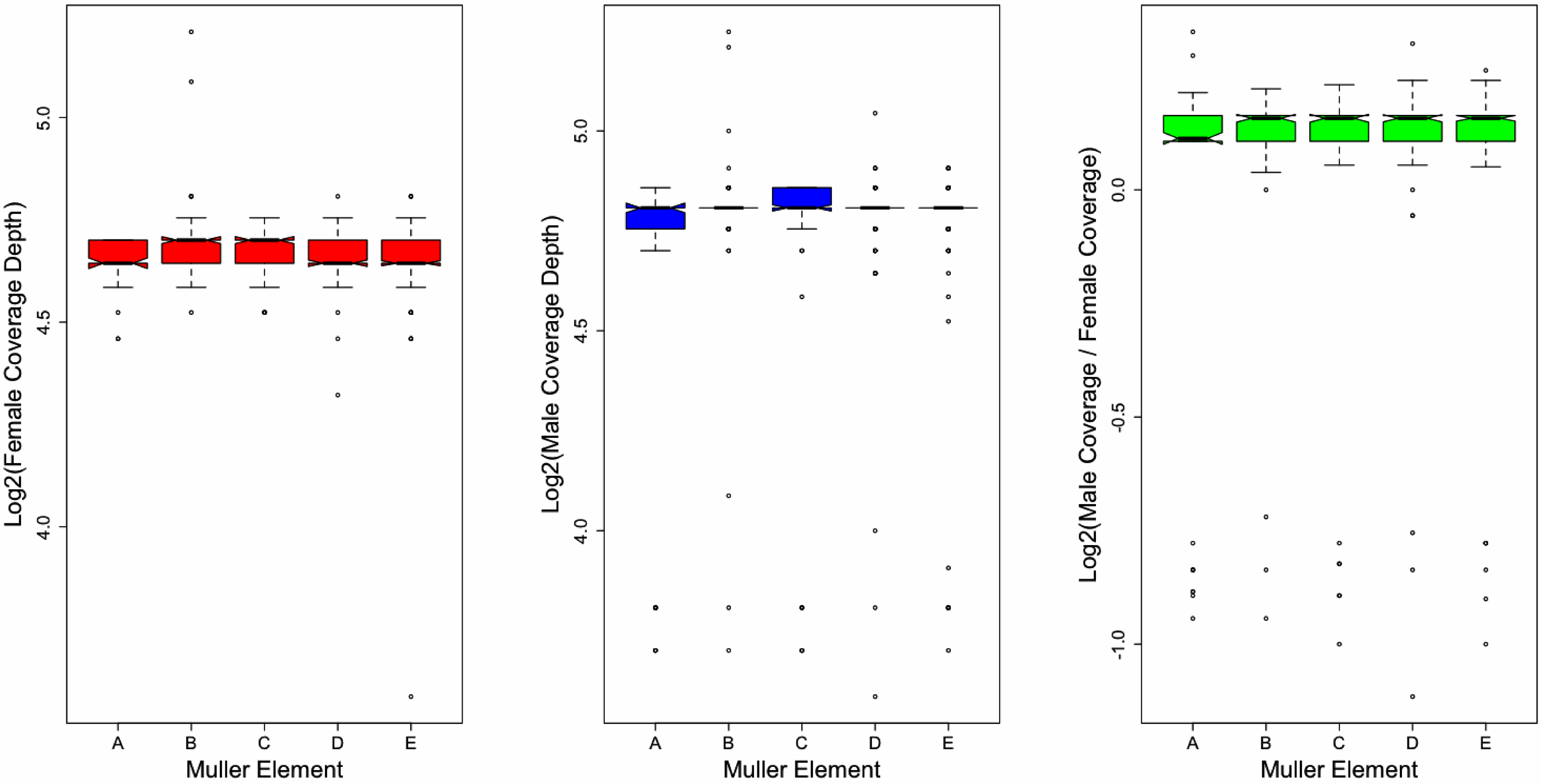

S2.10 Mayetiola destructor

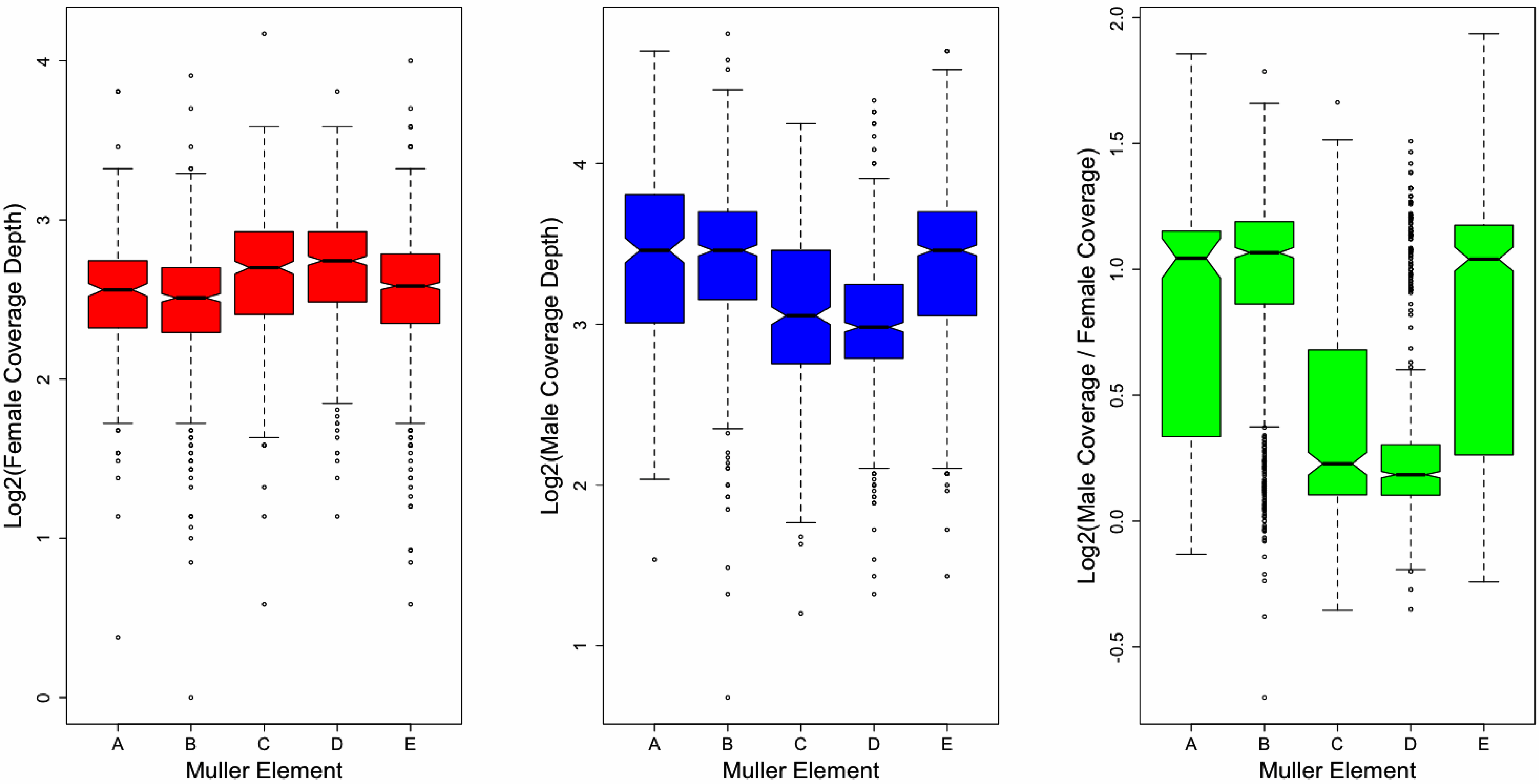

Figure S2
