# Supplementary material for: Numerous Transitions of Sex Chromosomes in Diptera
Source: PLoS Biol. 2015 Apr 16;13(4):e1002078. doi: 10.1371/journal.pbio.1002078 (PMC4400102; doi:10.1371/journal.pbio.1002078)
Supplement: S3 Fig — For each species, the left panel shows the density plot of Log2(M/F coverage) for all scaffolds. The black vertical line shows the maximum frequency, which is assumed to correspond to the median autosomal Log2(M/F coverage) (except in M. destructor, for which the second-highest frequency was considered to be the autosomal median; see Materials and Methods). The dotted red lines delimit the interval of Log2(M/F coverage) that we assign to our candidate X/Z scaffolds. The right panel shows the observed/expected number of scaffolds from each Muller element among the Z/X-candidates. Significant excesses (p < 0.01) are shown in red, non-significant differences are in blue. Data to generate this graph are to be found in file “S4 Data.” (PDF) [file pbio.1002078.s008.pdf]

S3.1 Tipula olearacea

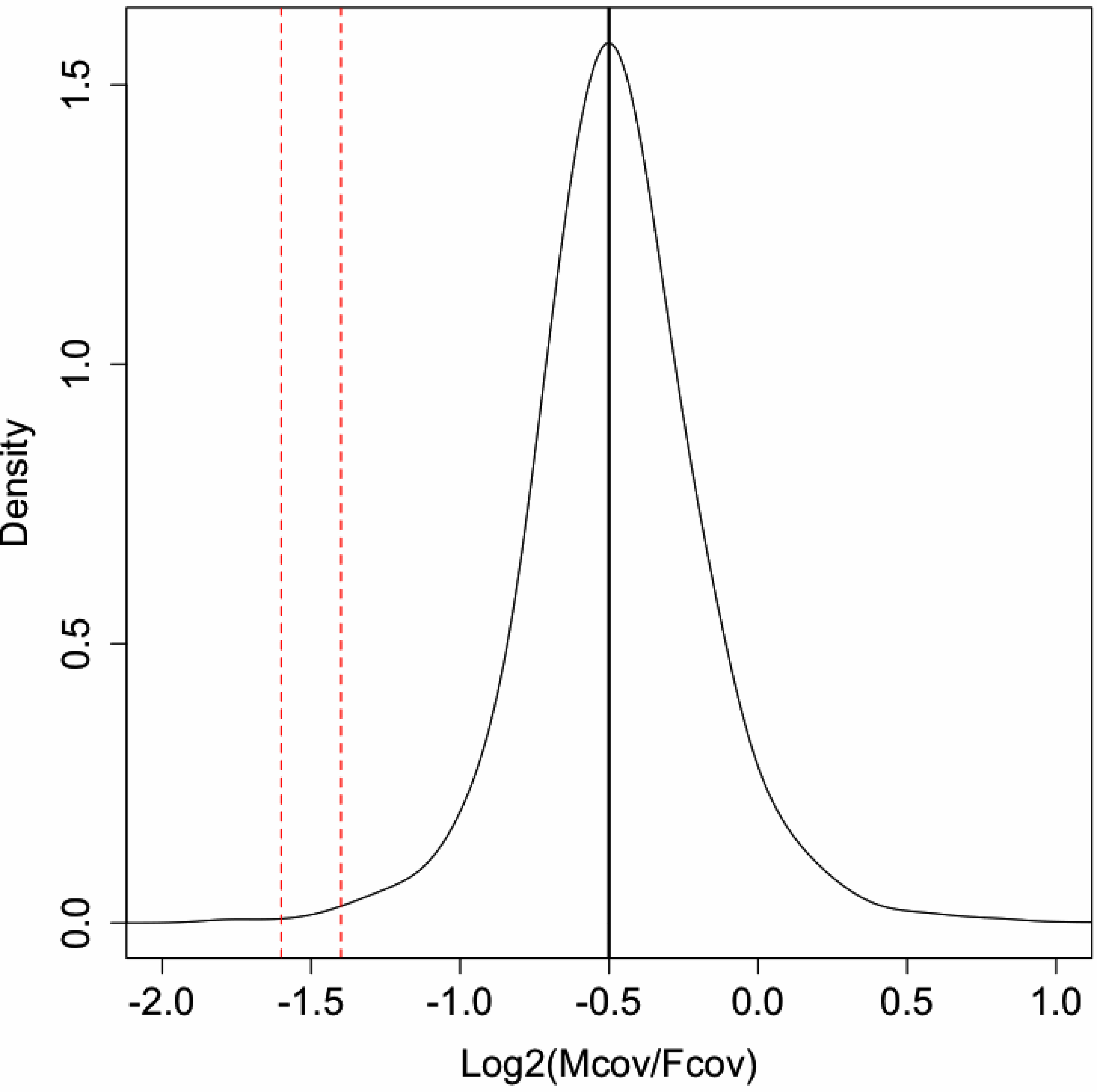

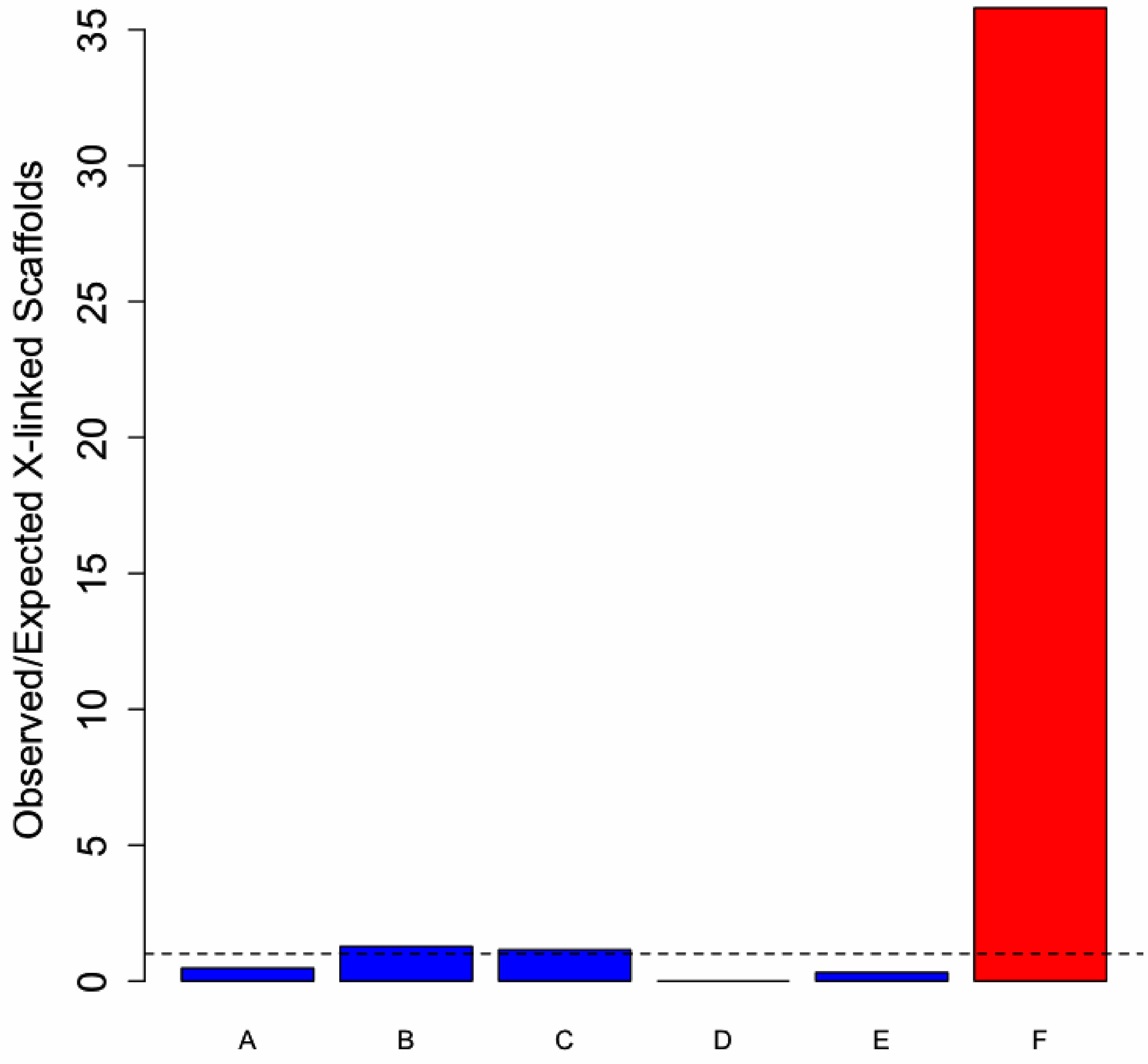

S3.2 Trichoceridae sp

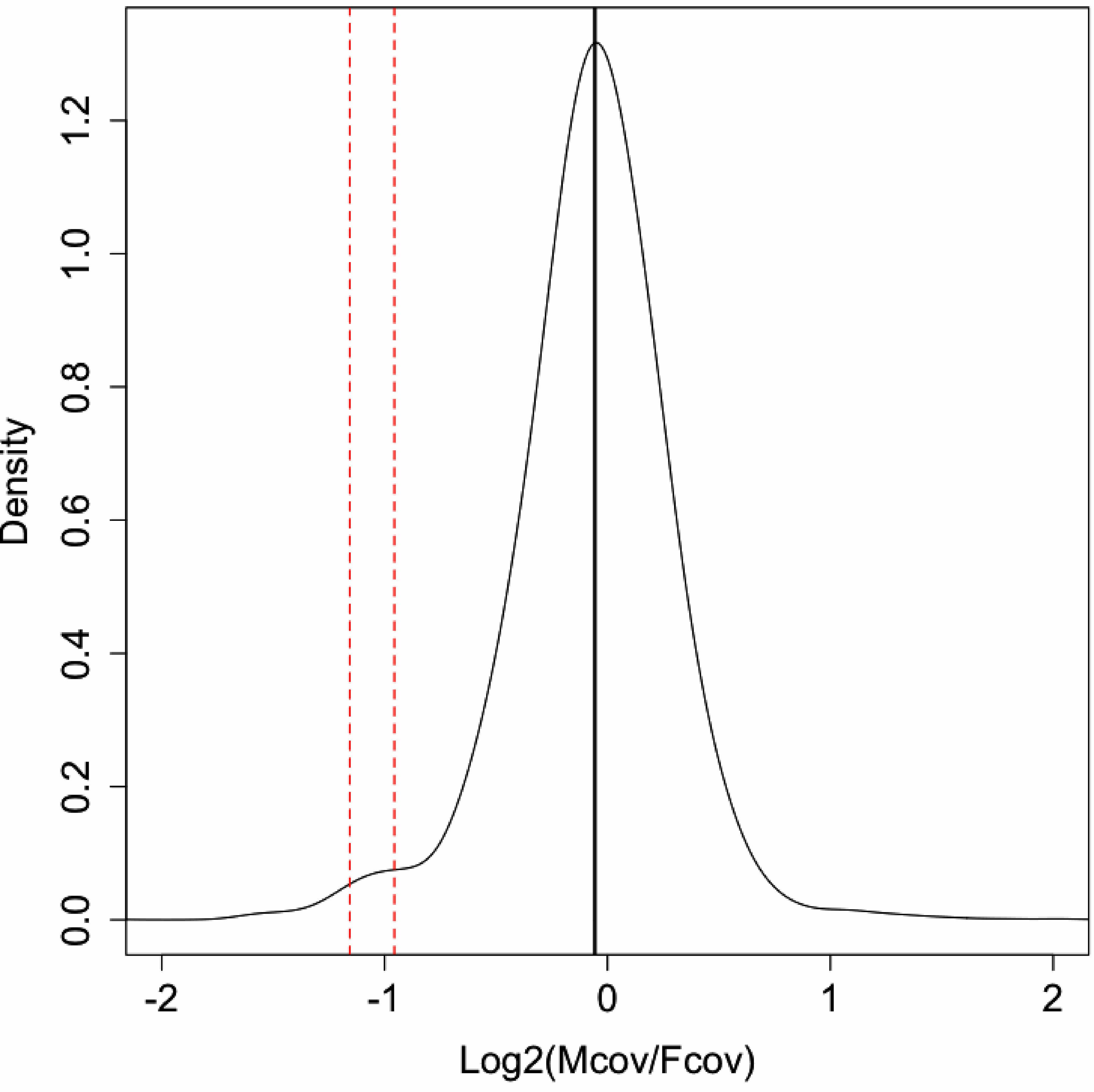

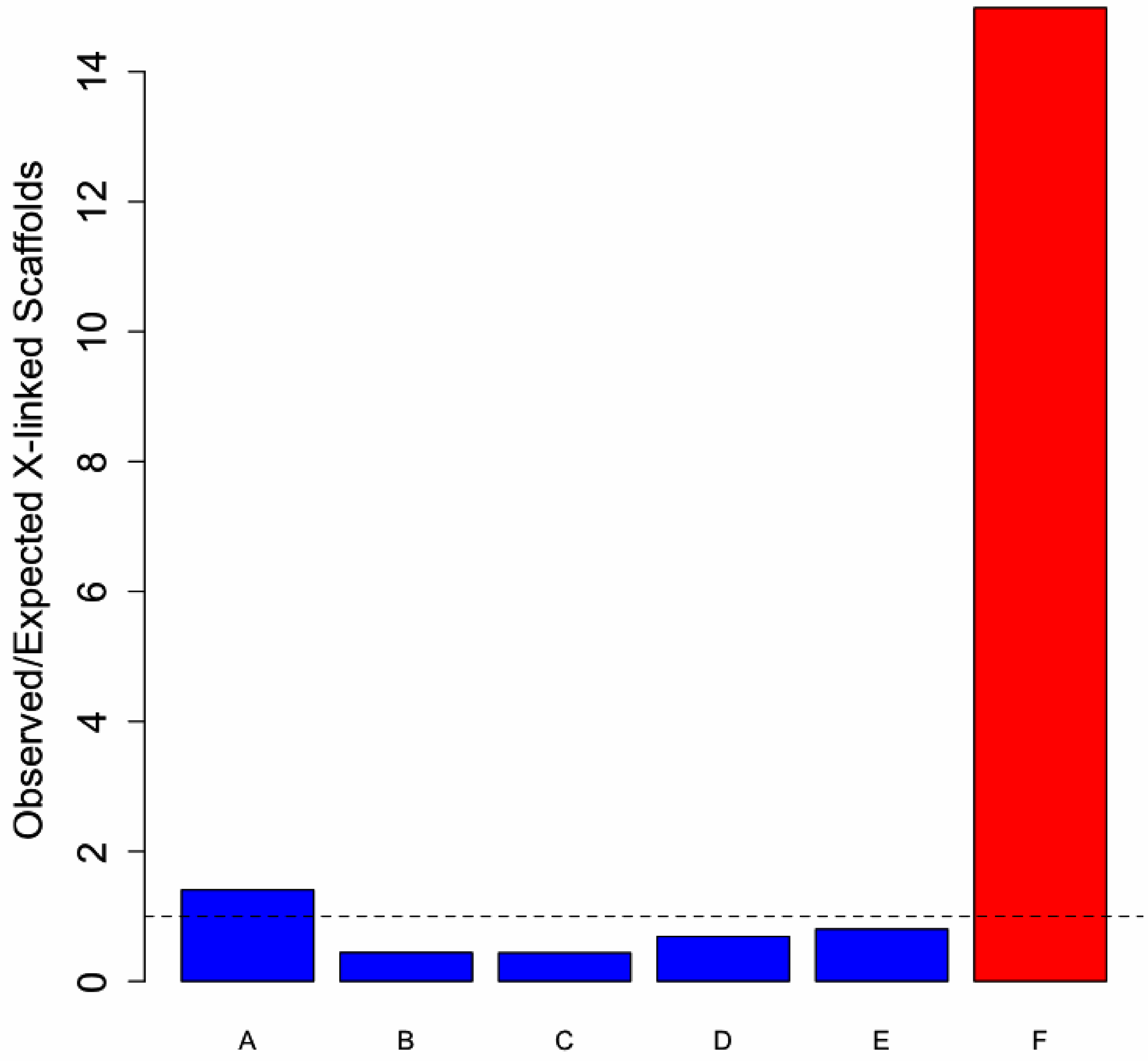

Figure S3

S3.3 Clogmia albipunctata

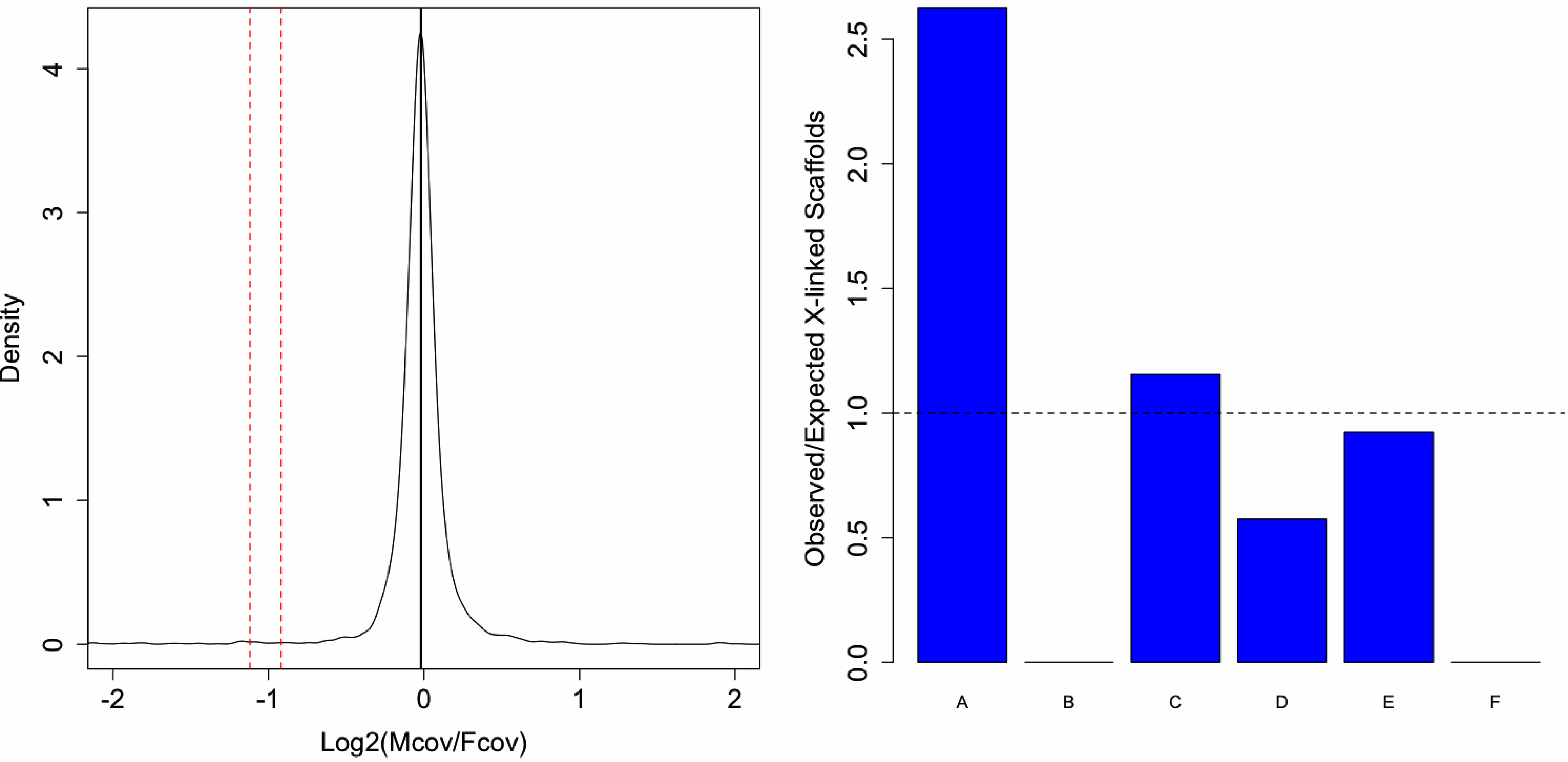

S3.4 Chironomus riparius

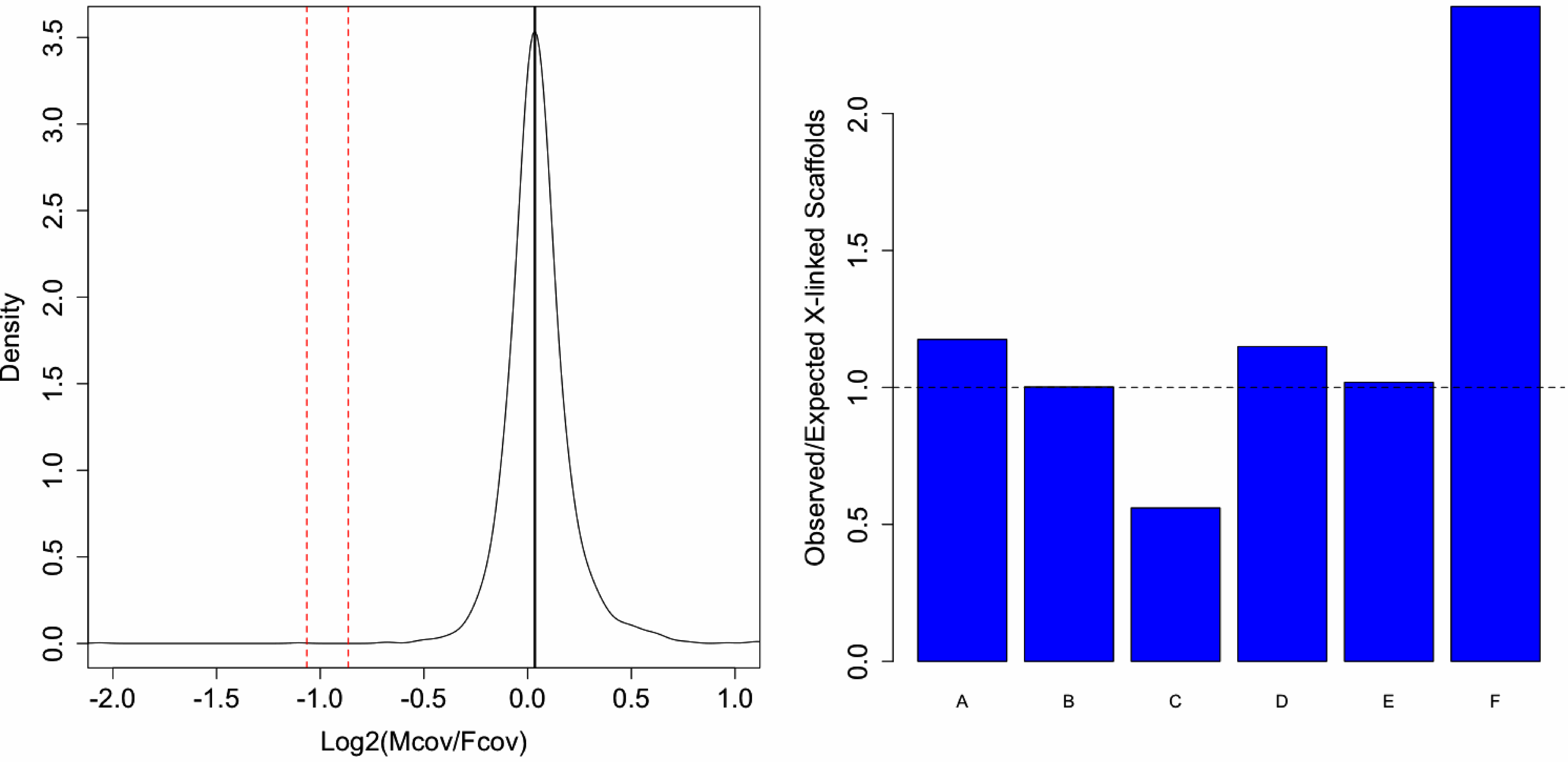

Figure S3

S3.5 Chaoborus trivittatus

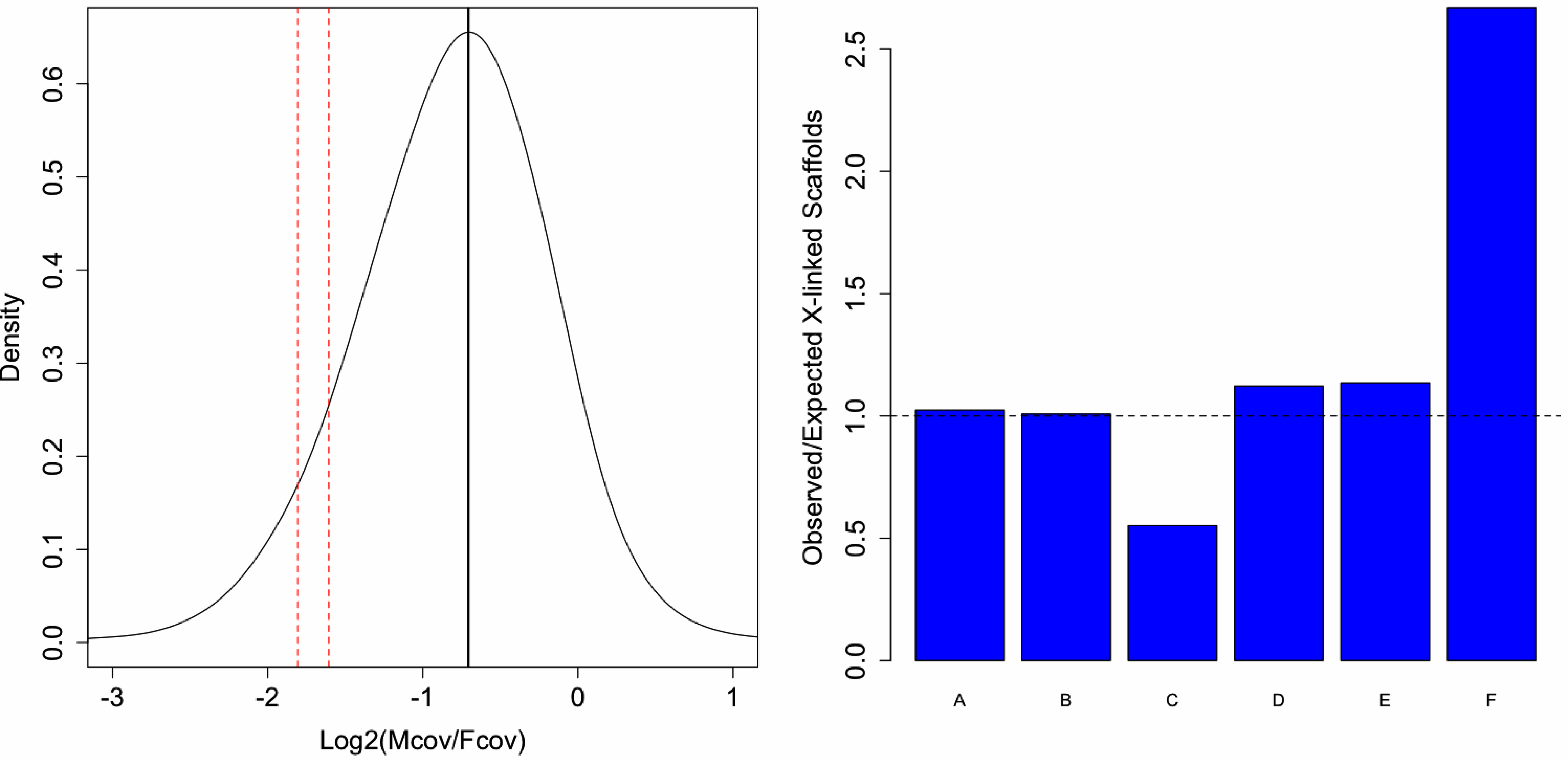

S3.6 Monchlonyx cinctipes

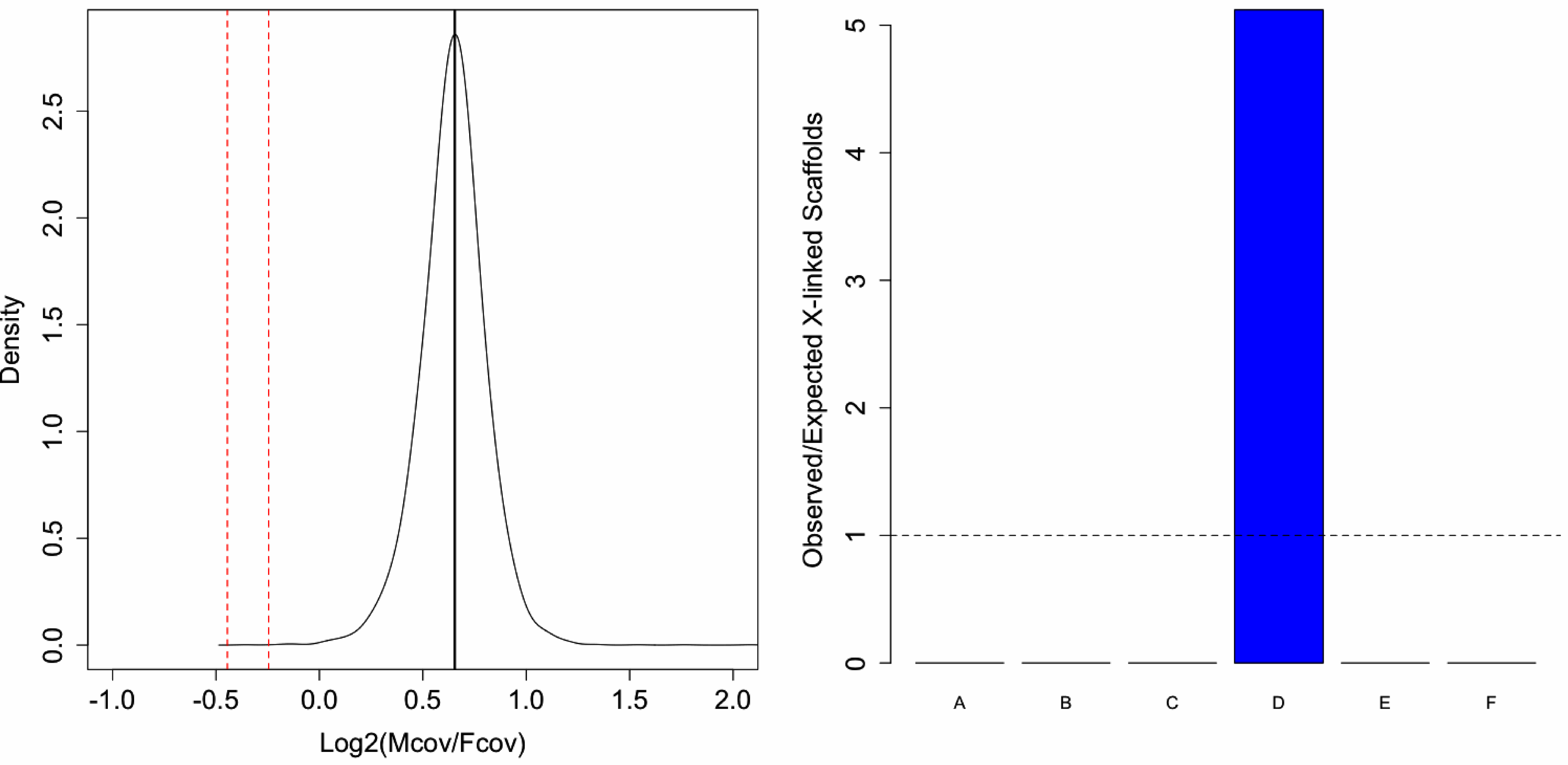

Figure S3

S3.7 Anopheles gambiae

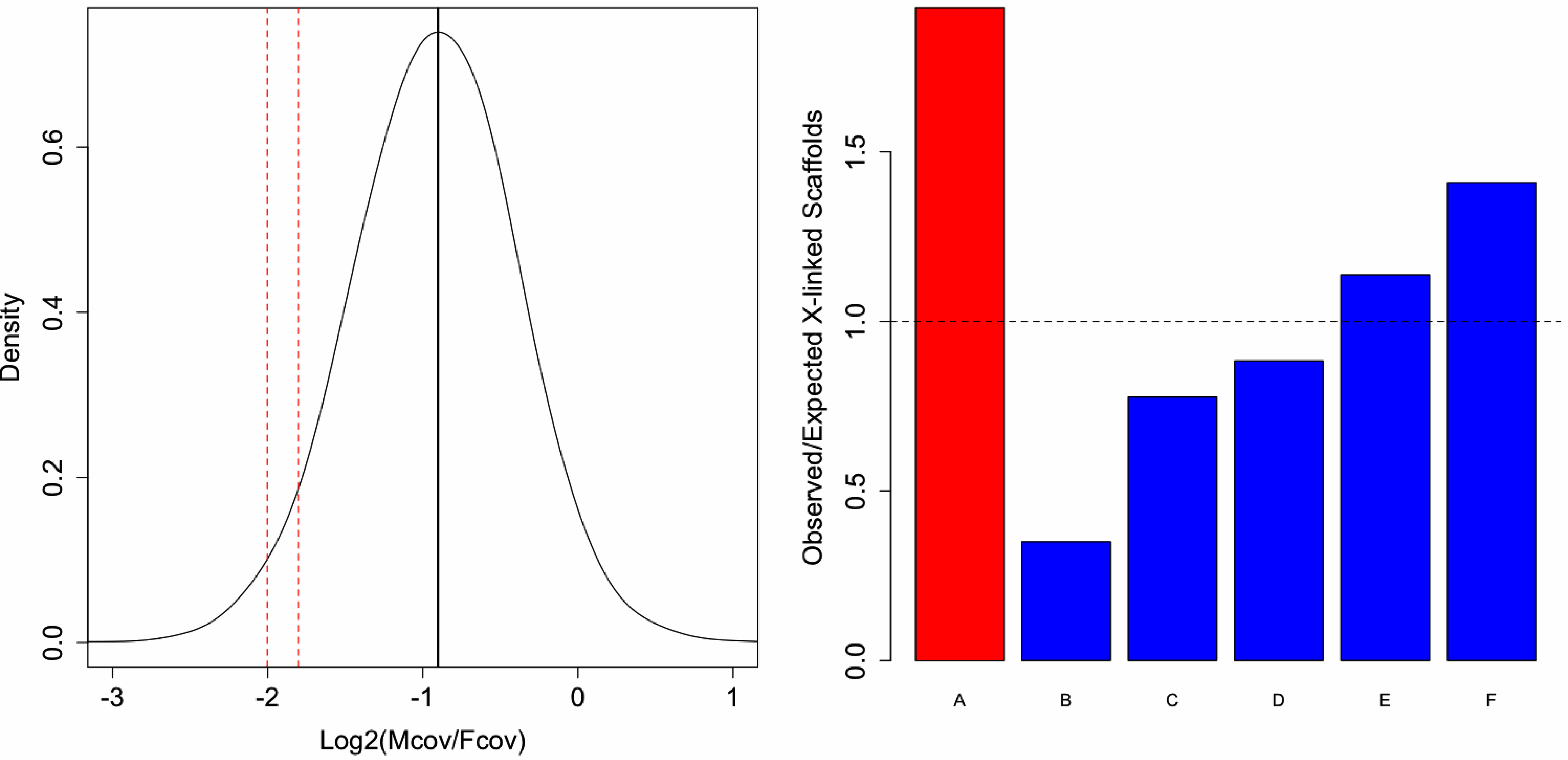

S3.8 Aedes aegypti

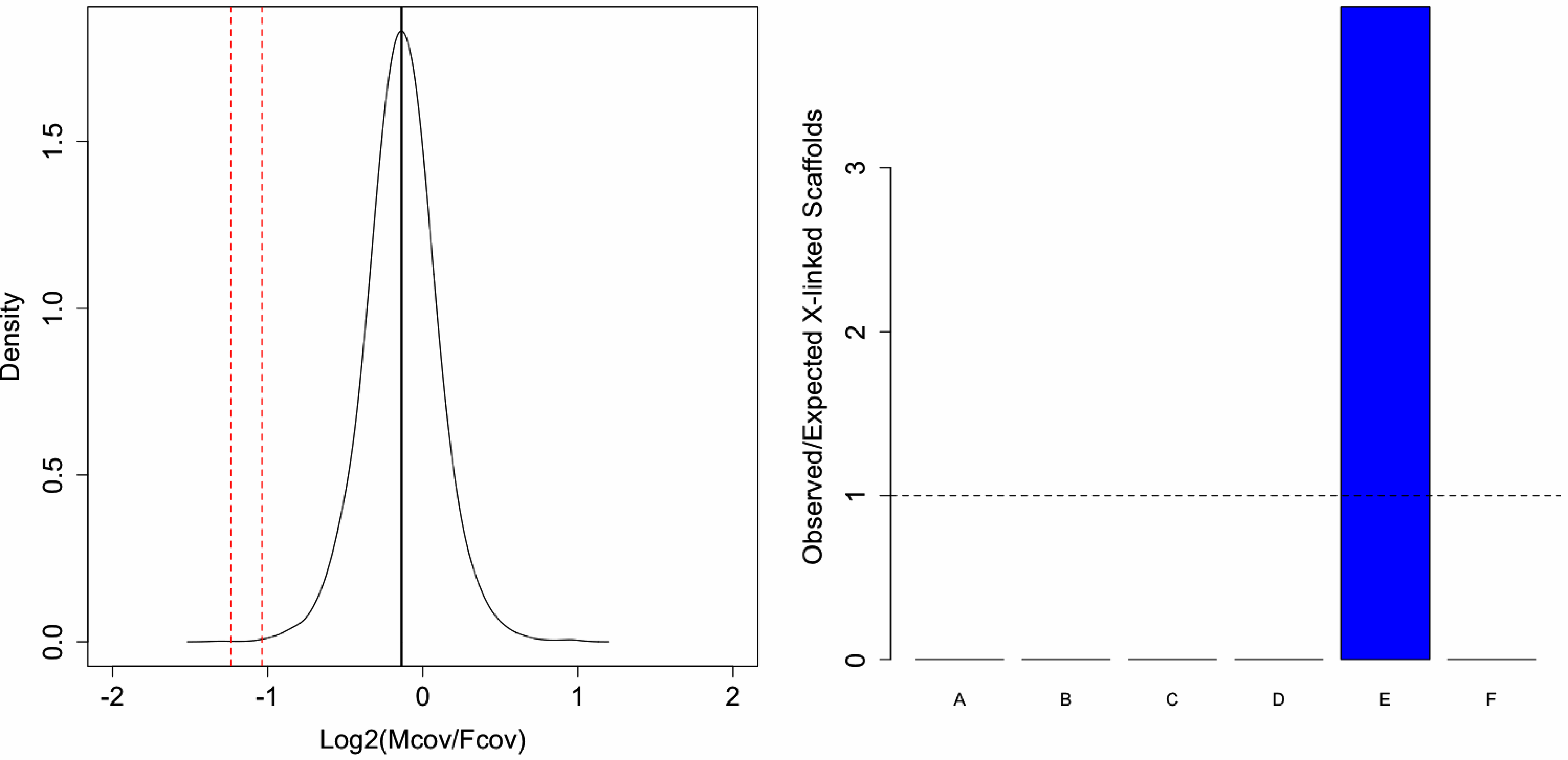

Figure S3

S3.9 Coboldia fuscipes

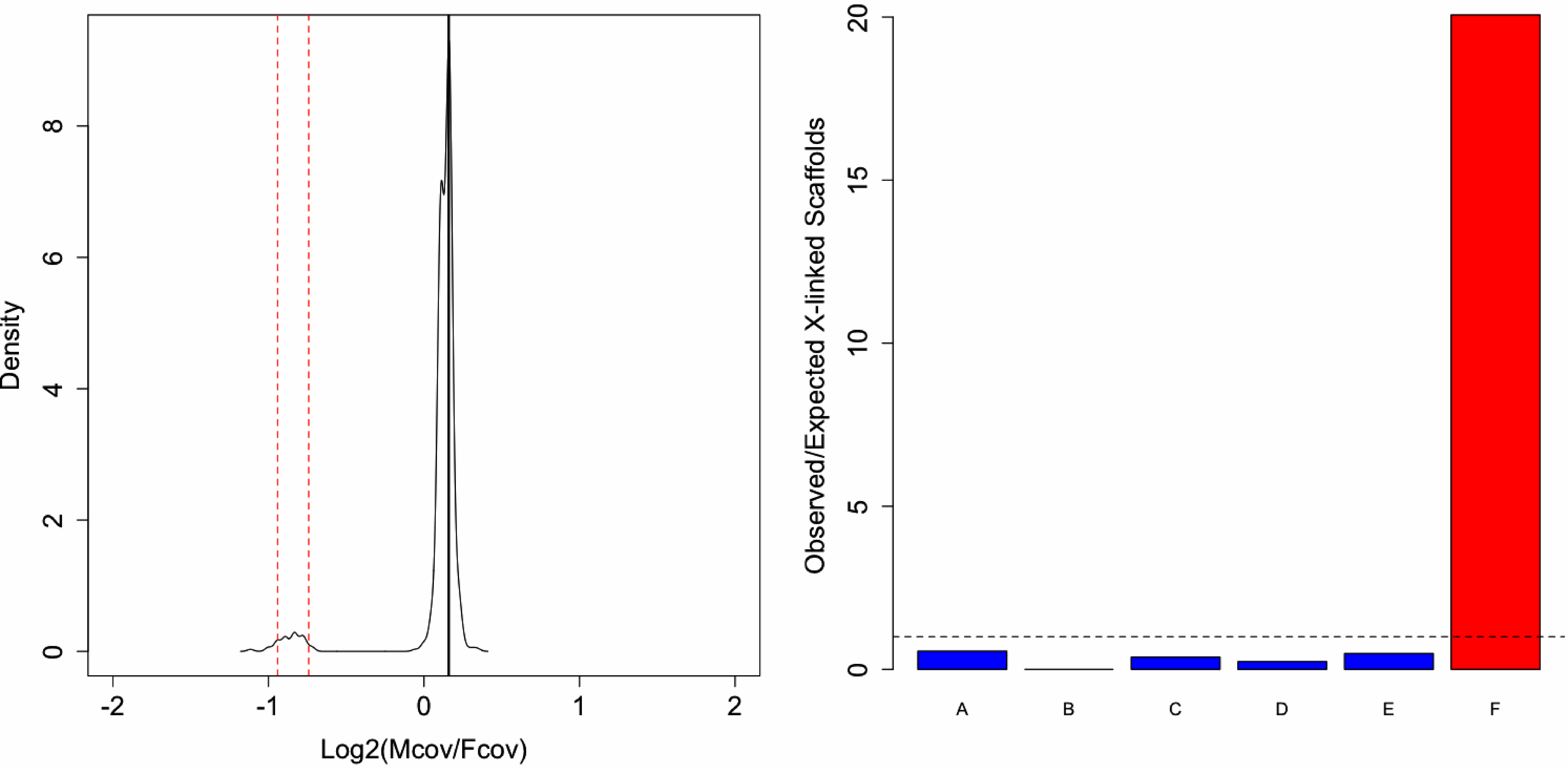

S3.10 Mayetiola destructor

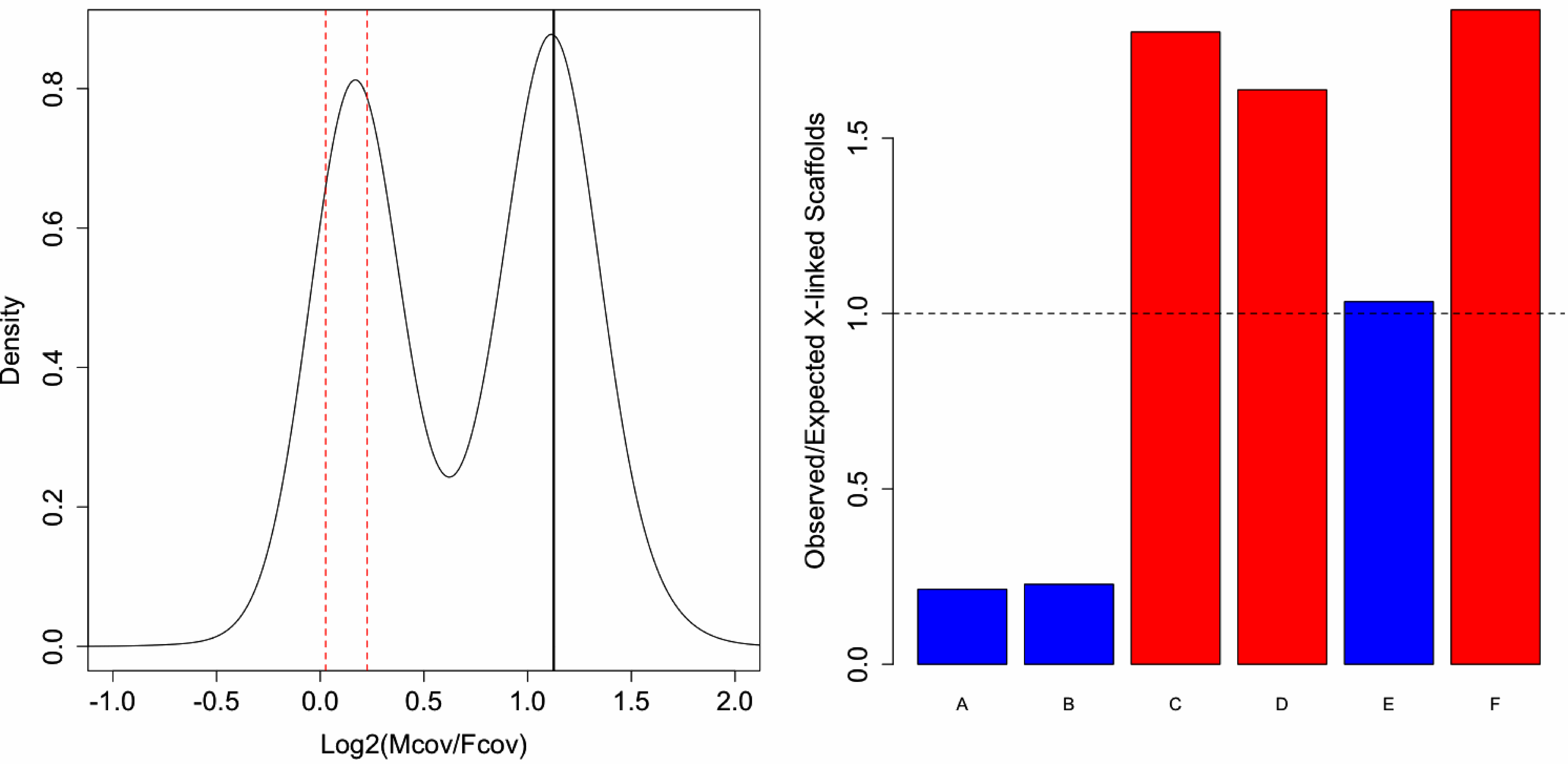

Figure S3

S3.11 Hermetia illucens

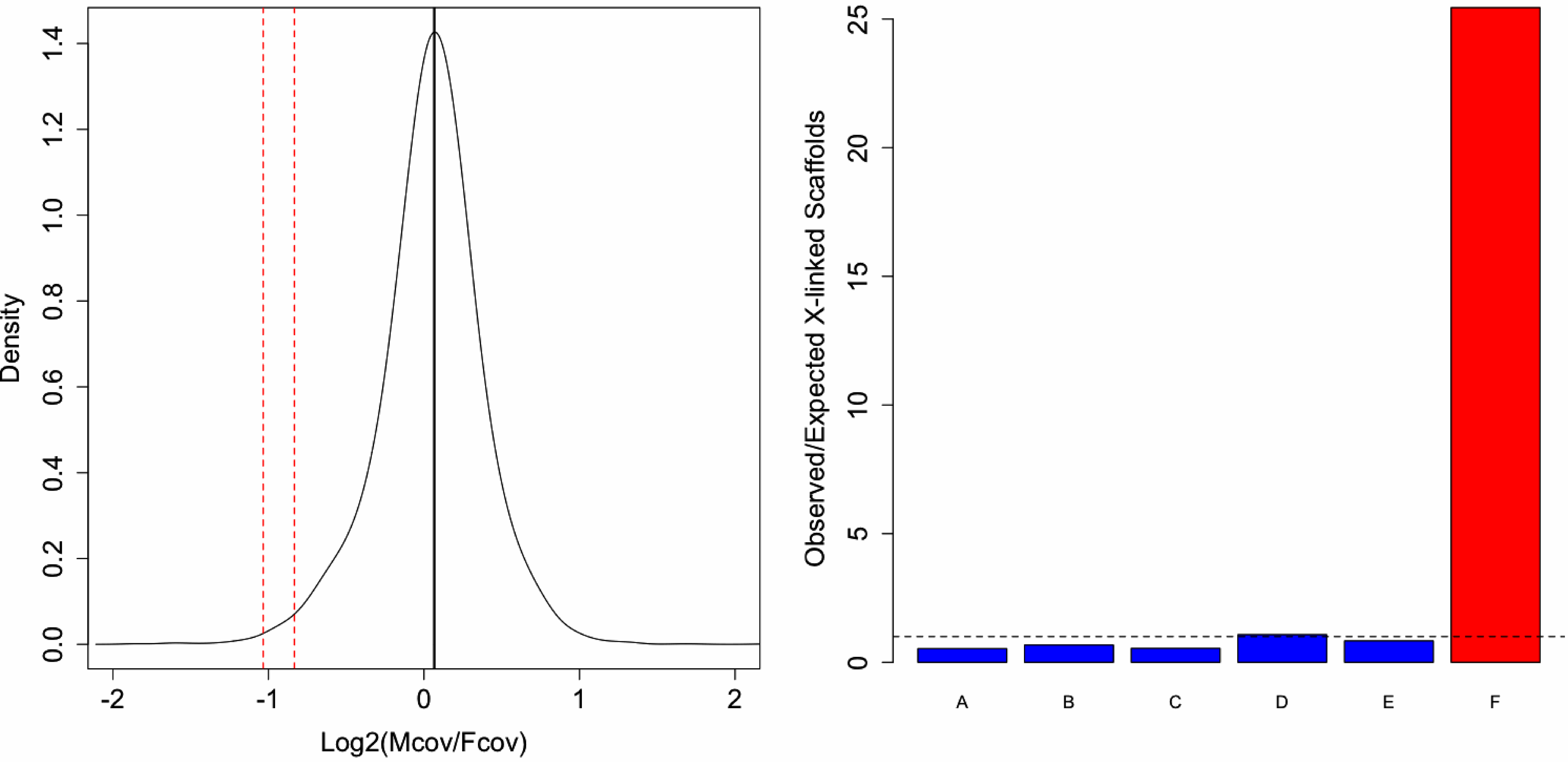

S3.12 Holcocephala fusca

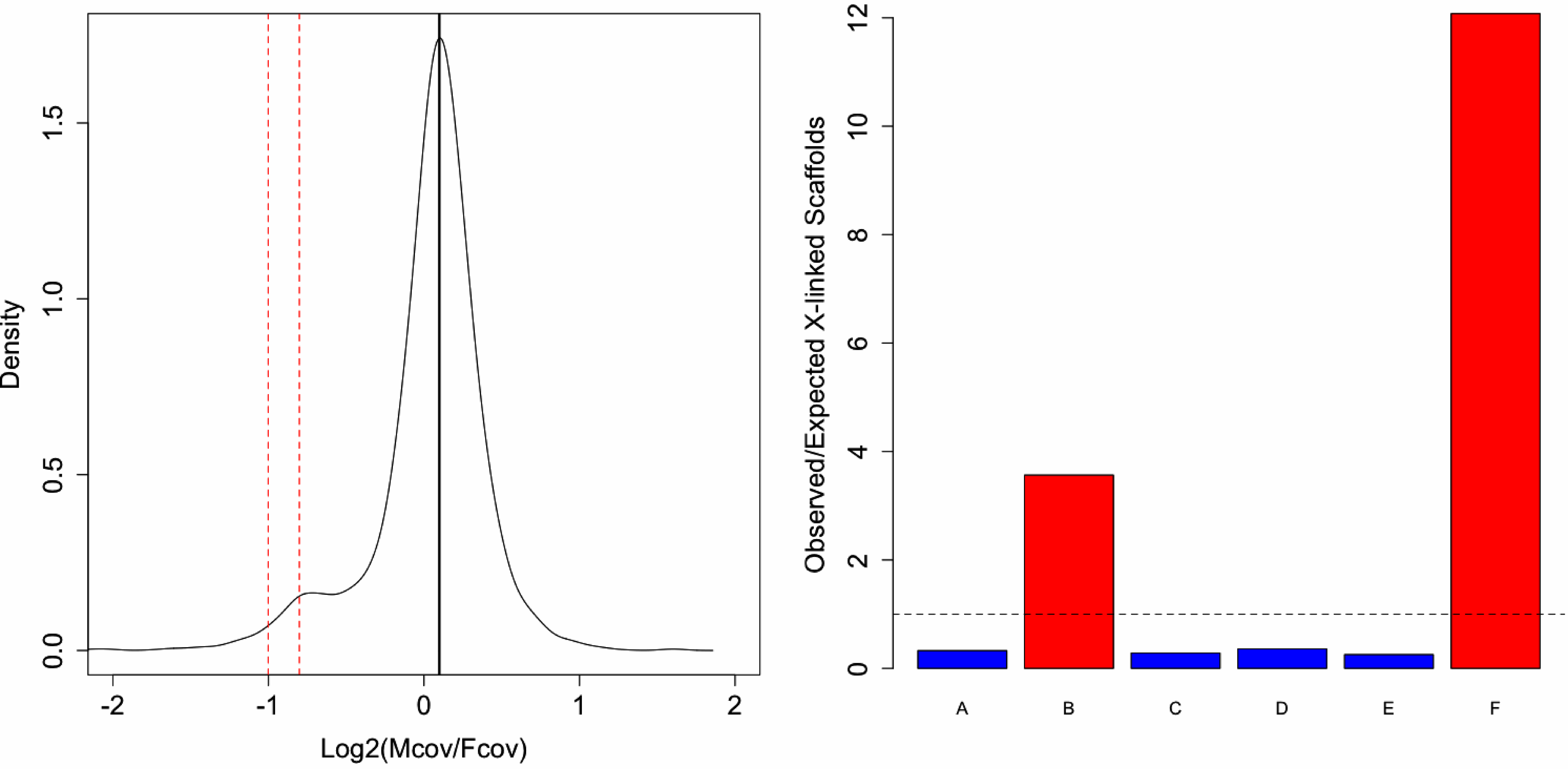

Figure S3

S3.13 Condylostylus patibulatus

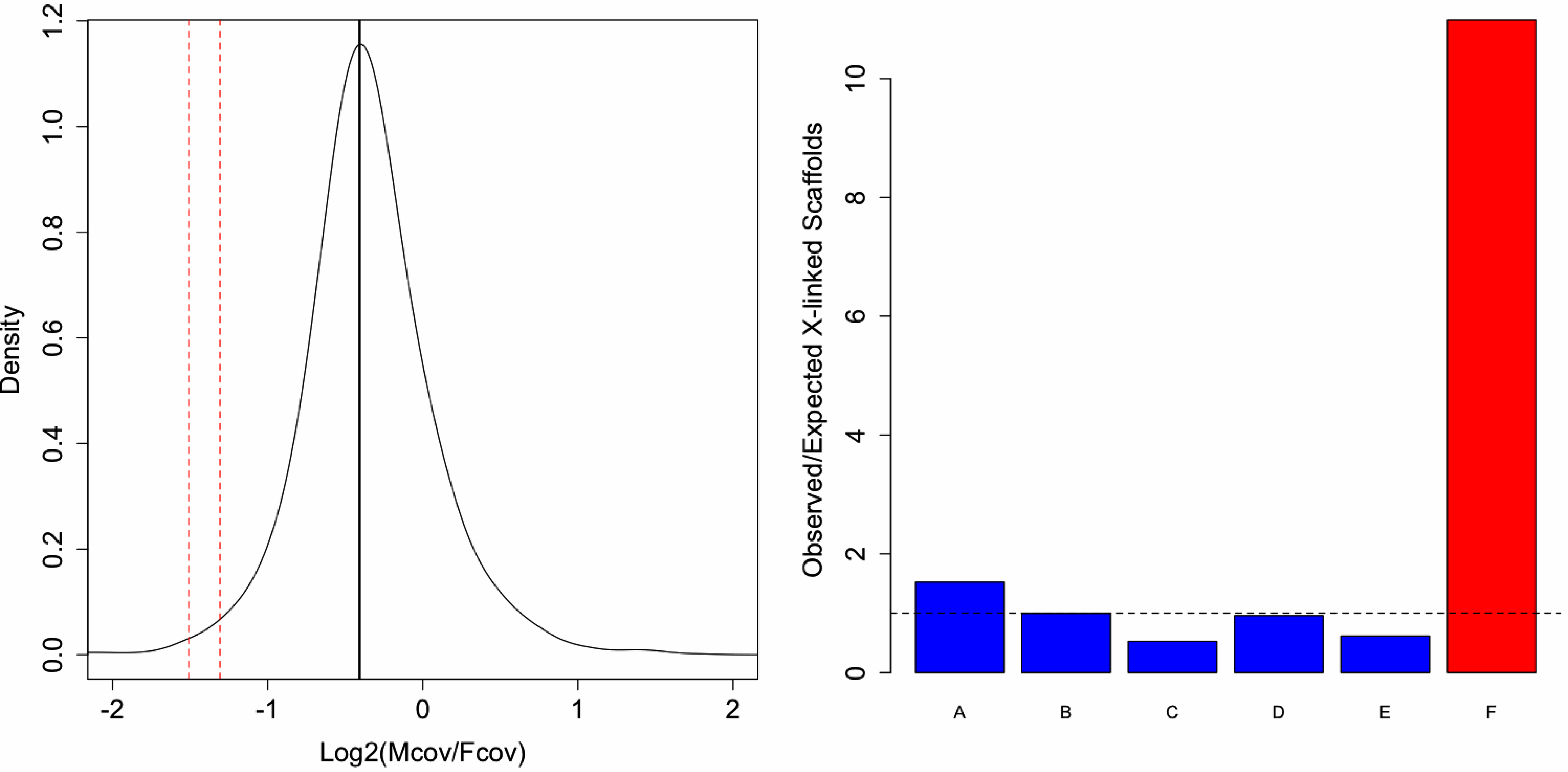

S3.14 Megaselia abdita

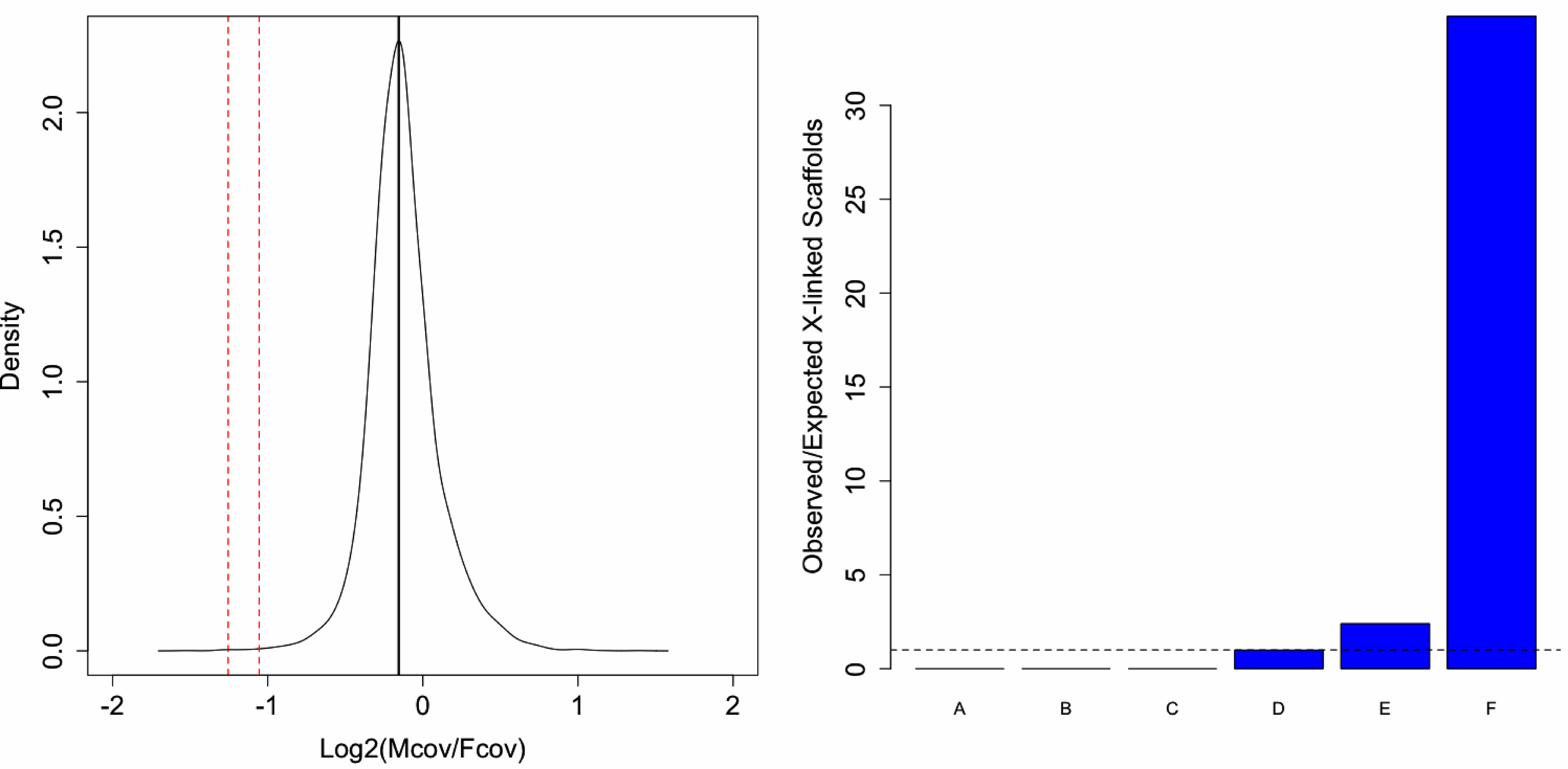

Figure S3

S3.15 Eristalis dimidiata

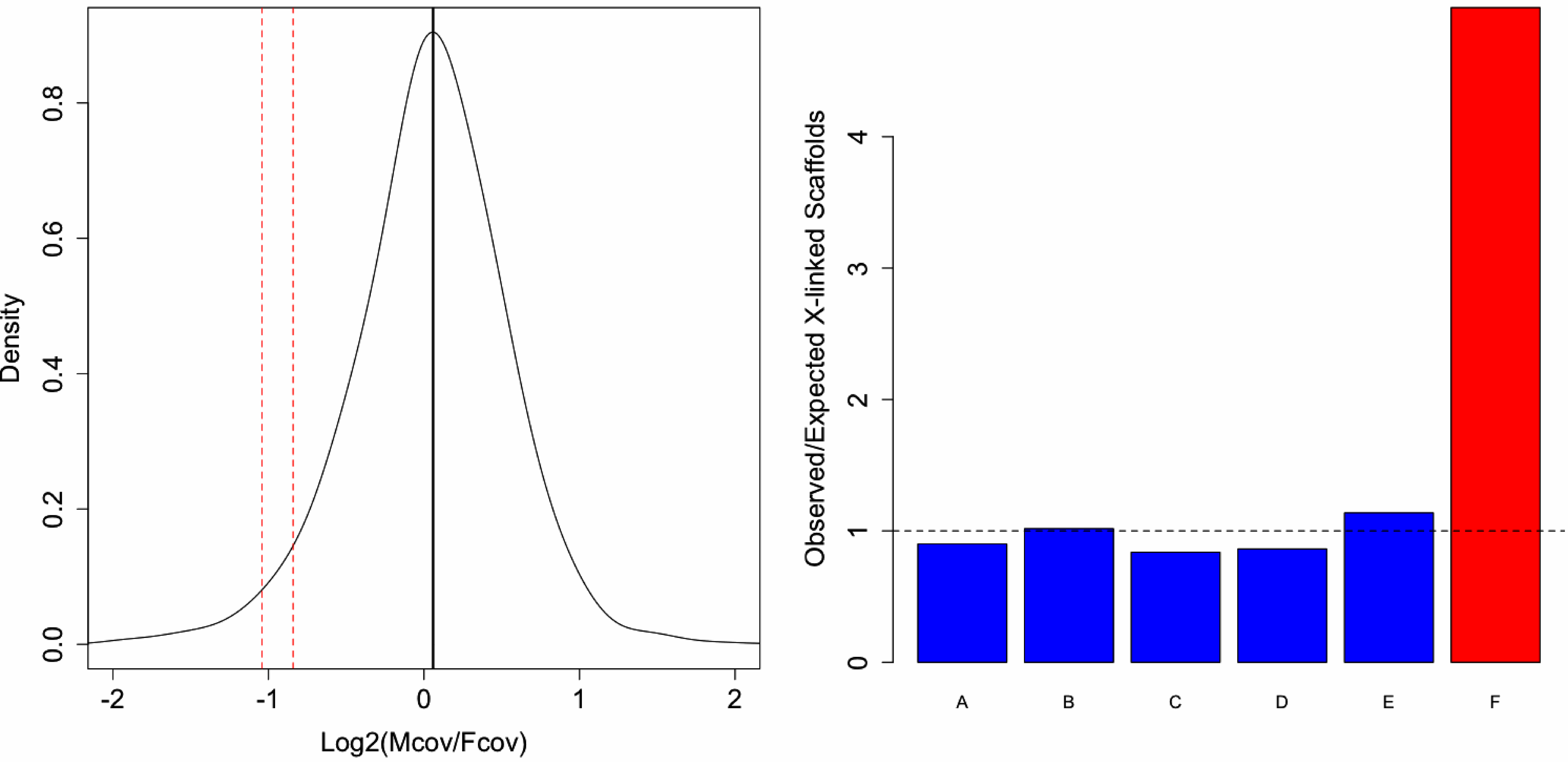

S3.16 Themira minor

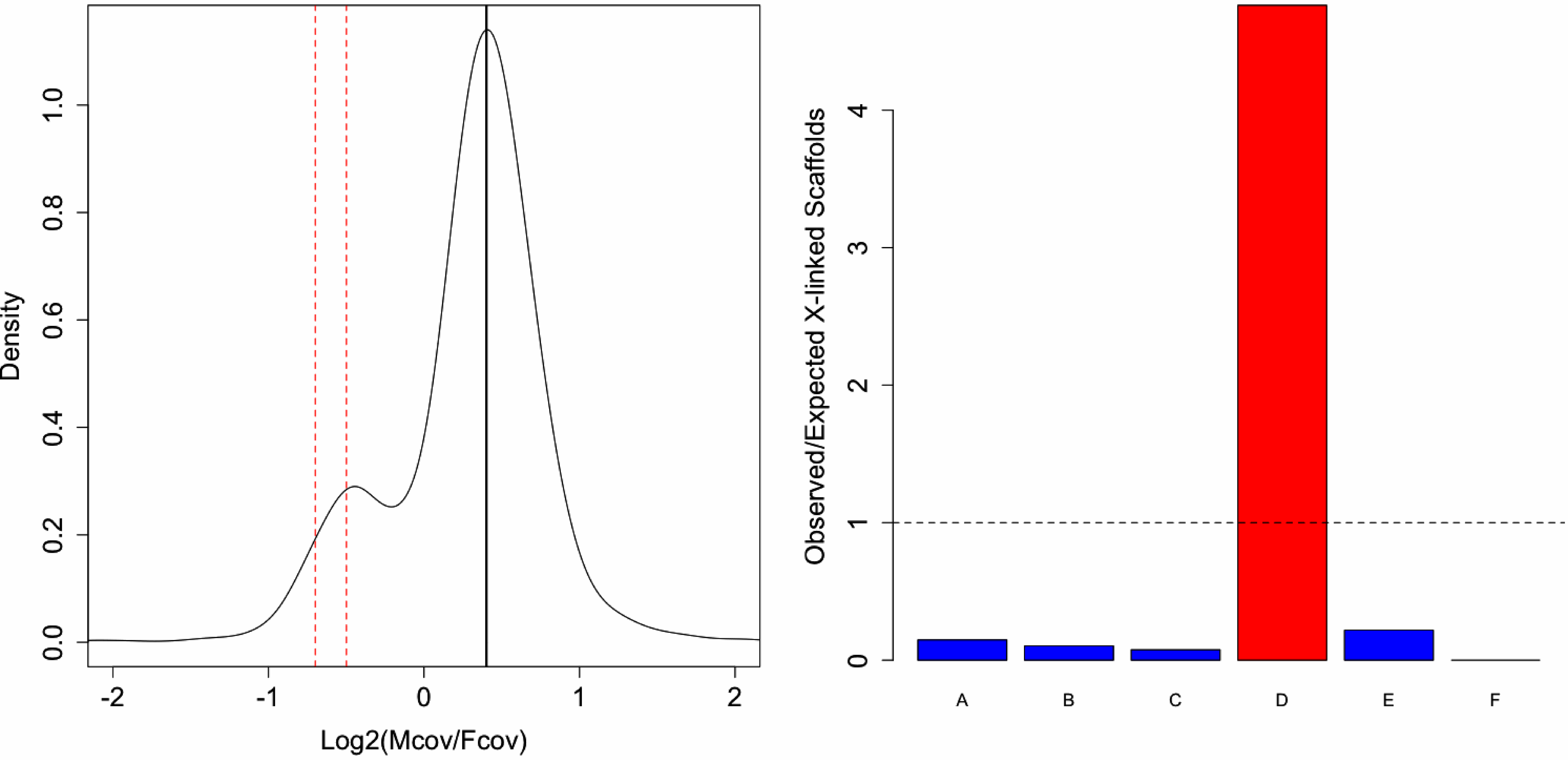

Figure S3

S3.17 Eutreta diana

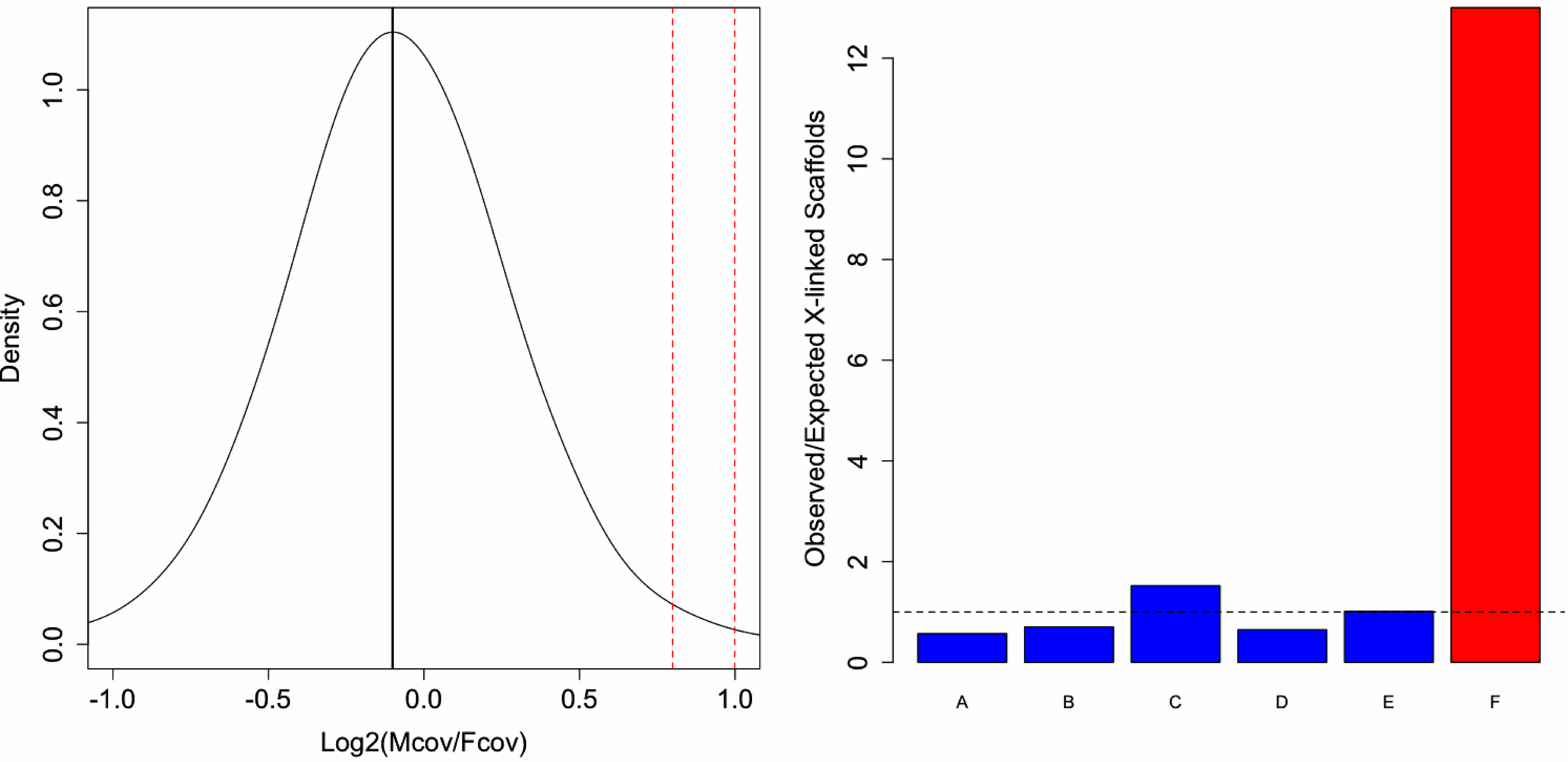

S3.18 Tephritis californica

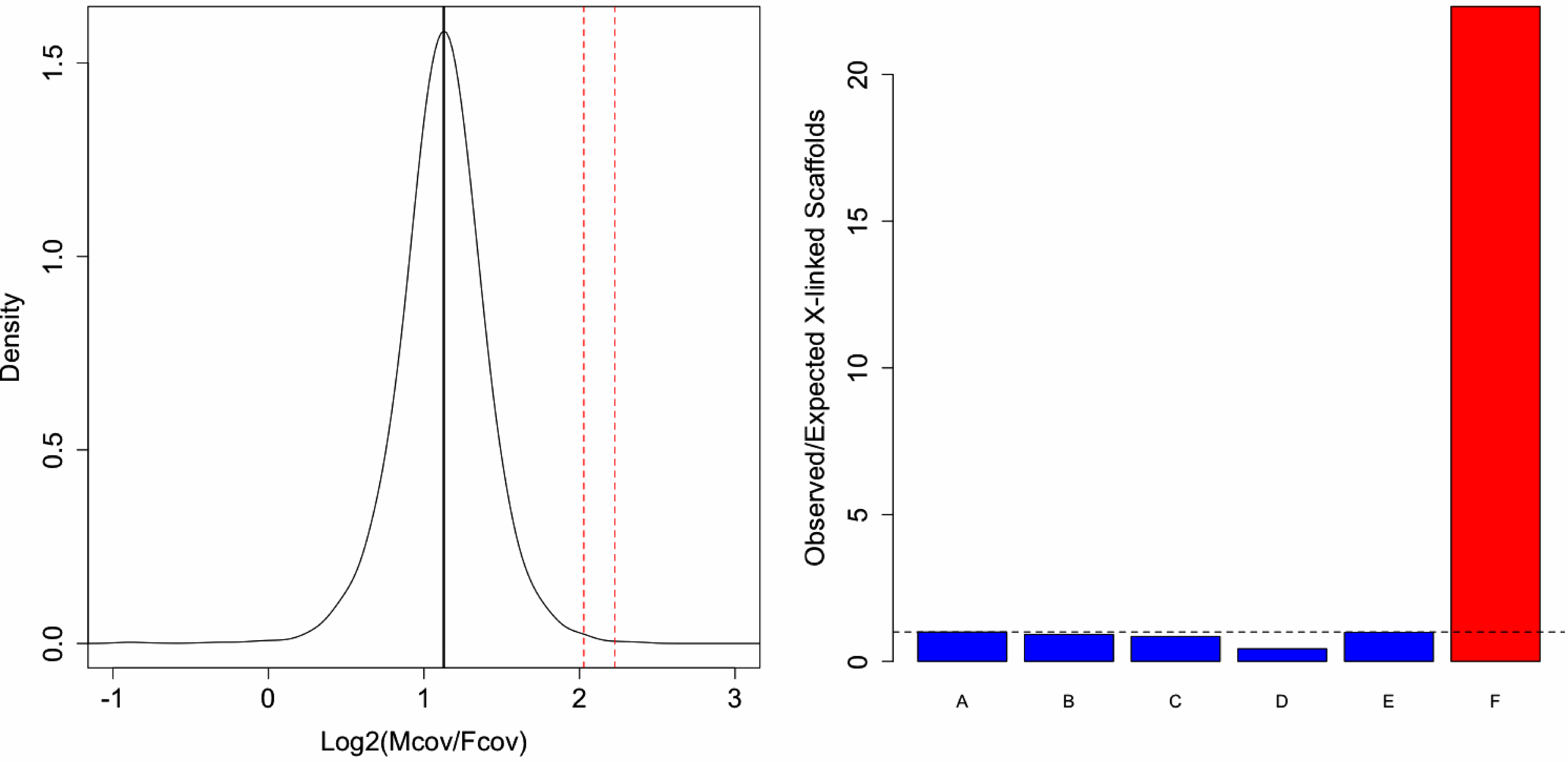

Figure S3

S3.19 Trupanea jonesi

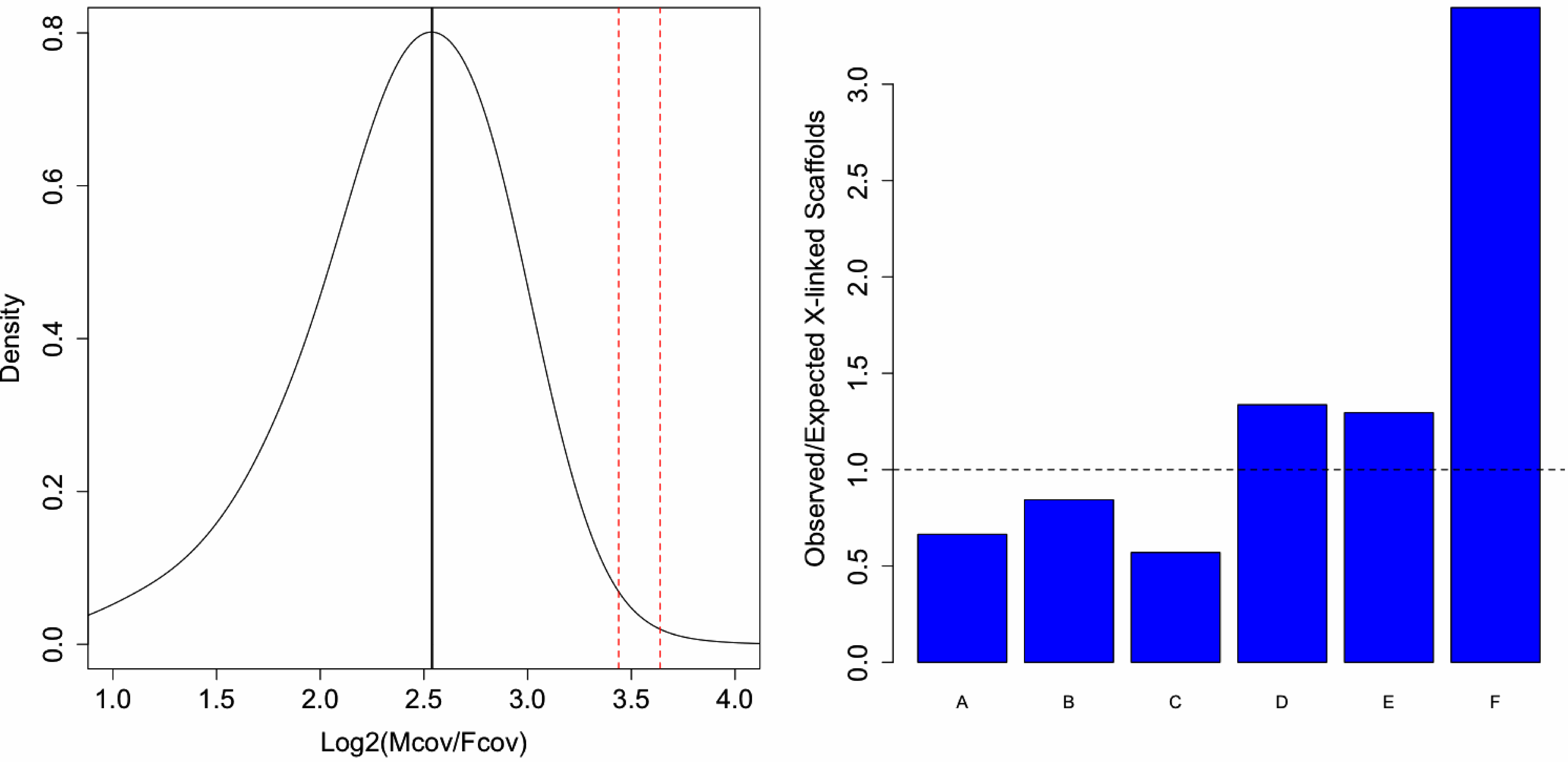

S3.20 Bactrocera oleae

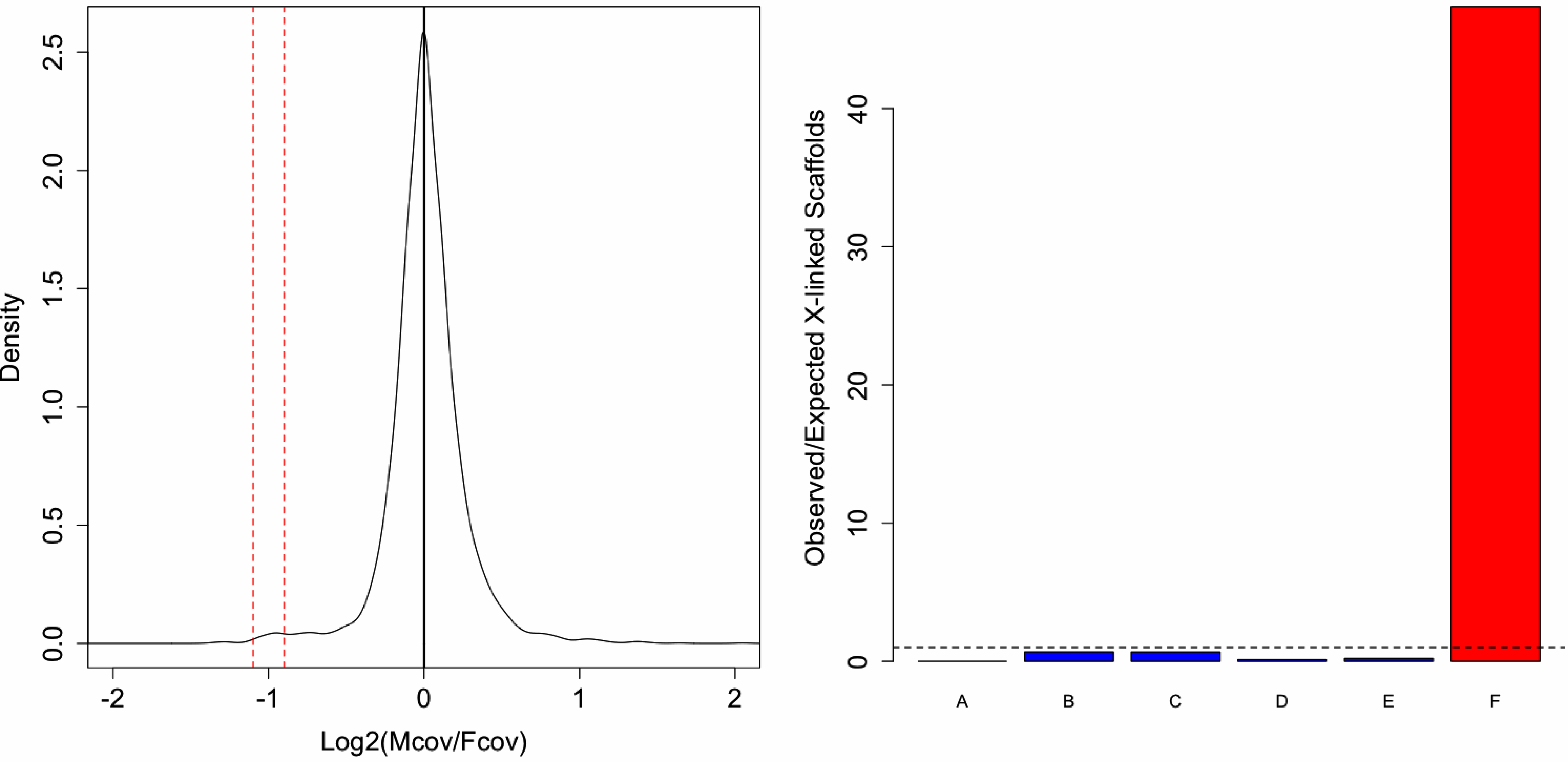

Figure S3

S3.21 Teleopsis dalmanni

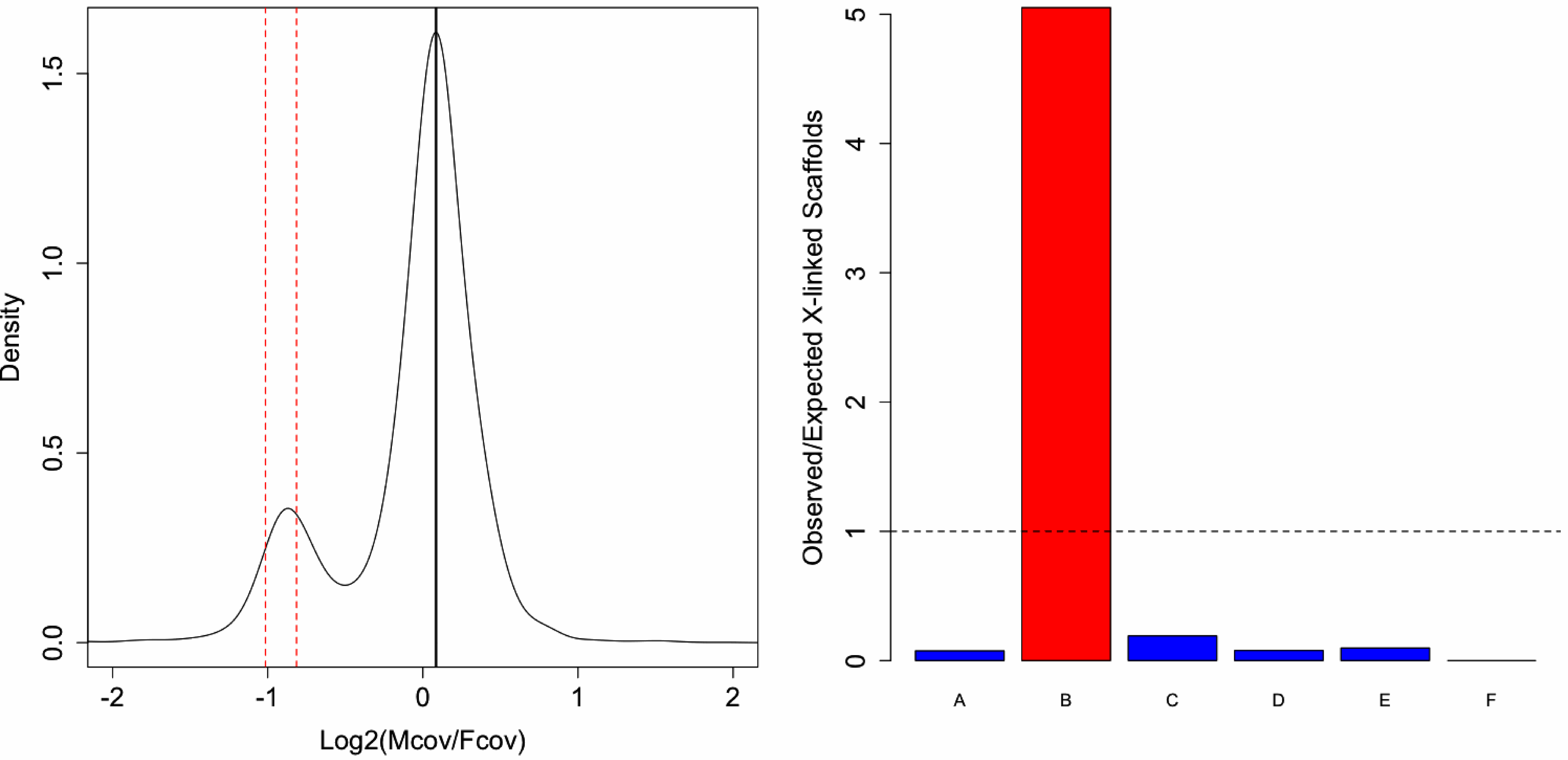

S3.22 Sphyracephala brevicornis

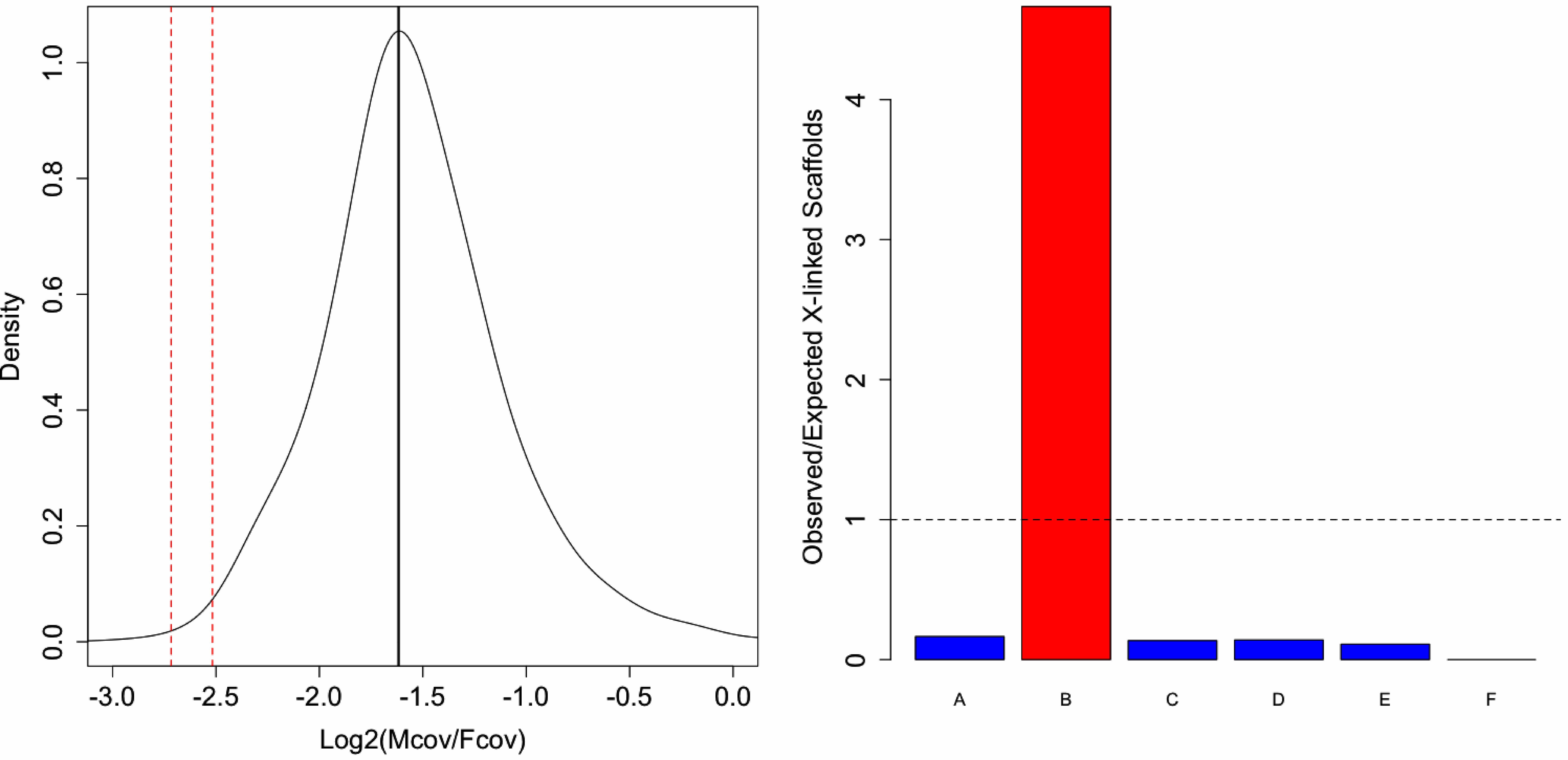

Figure S3

S3.23 Lyriomyza trifolii

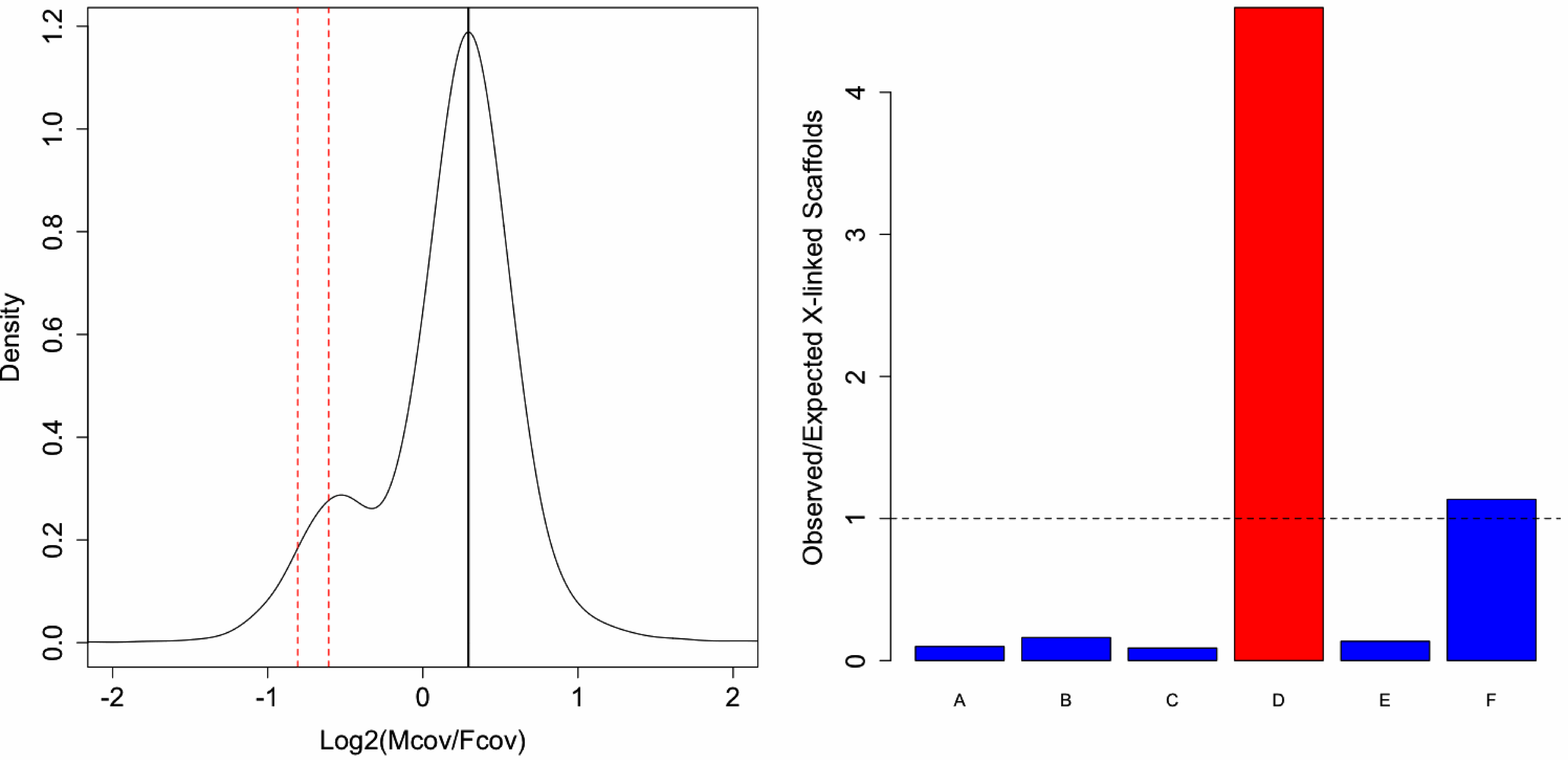

S3.24 Ephydra hians

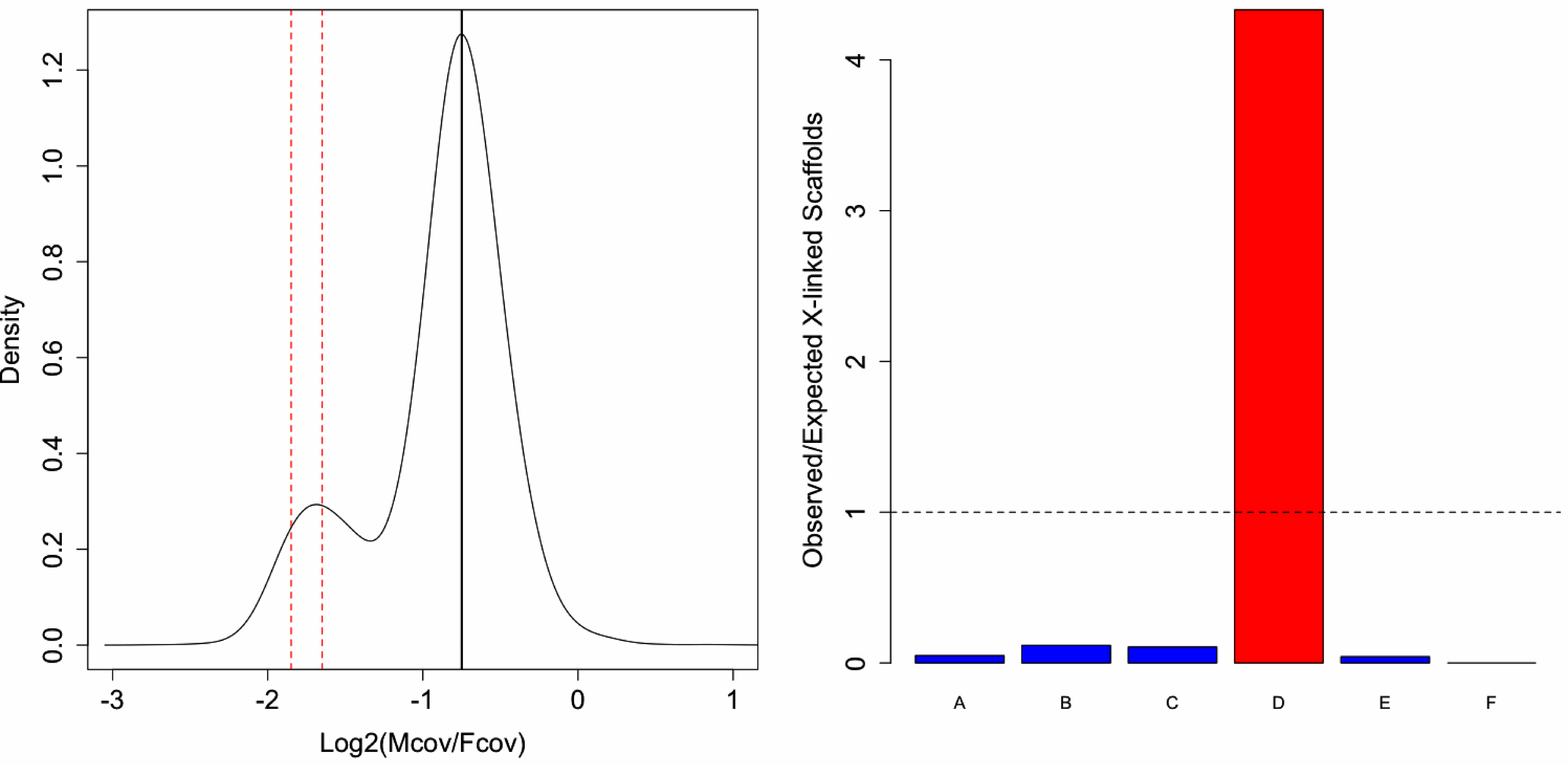

Figure S3

S3.25 Ephydra gracilis

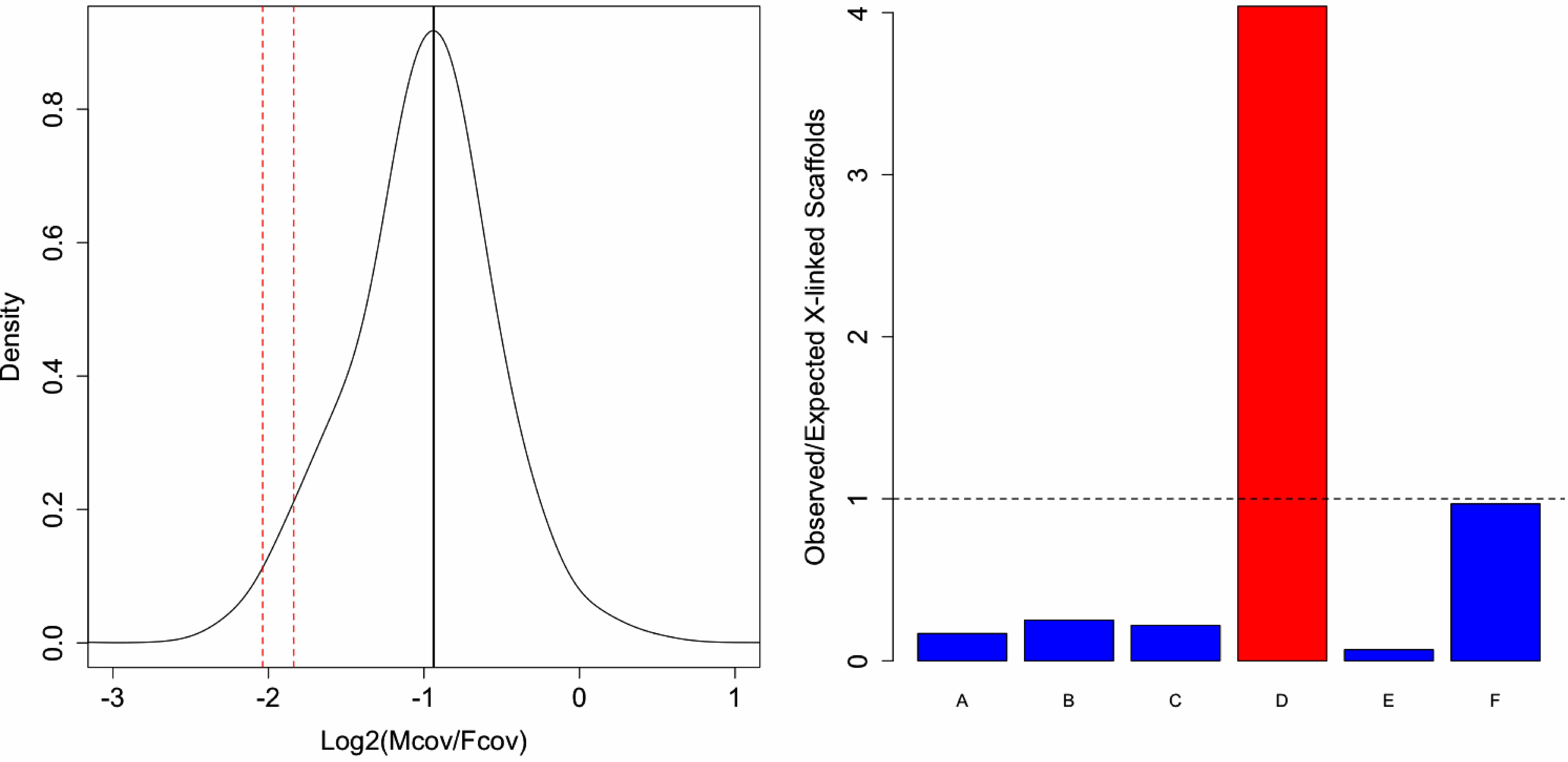

S3.26 Phortica variegata

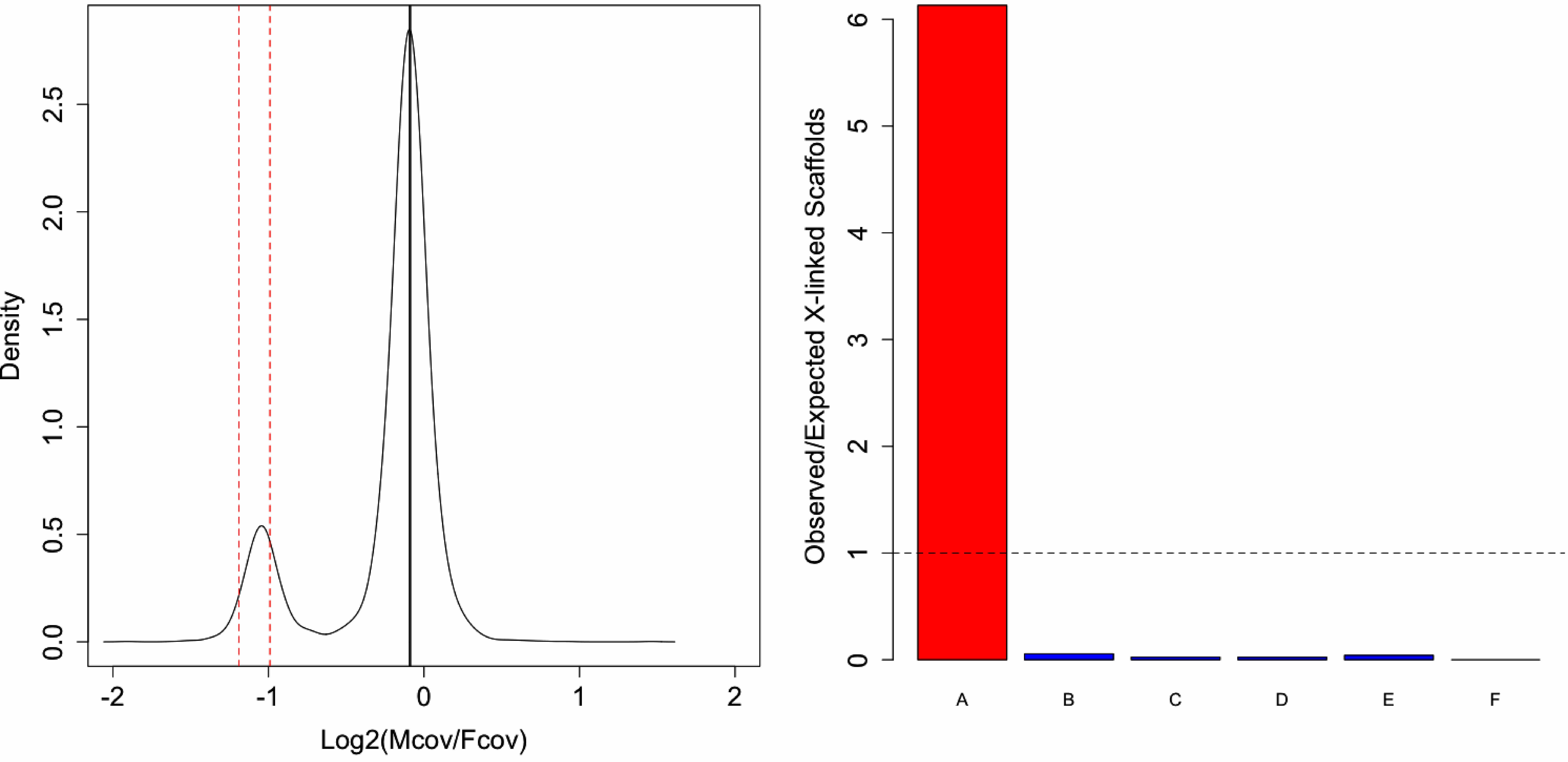

Figure S3

S3.27 Drosophila albomicans

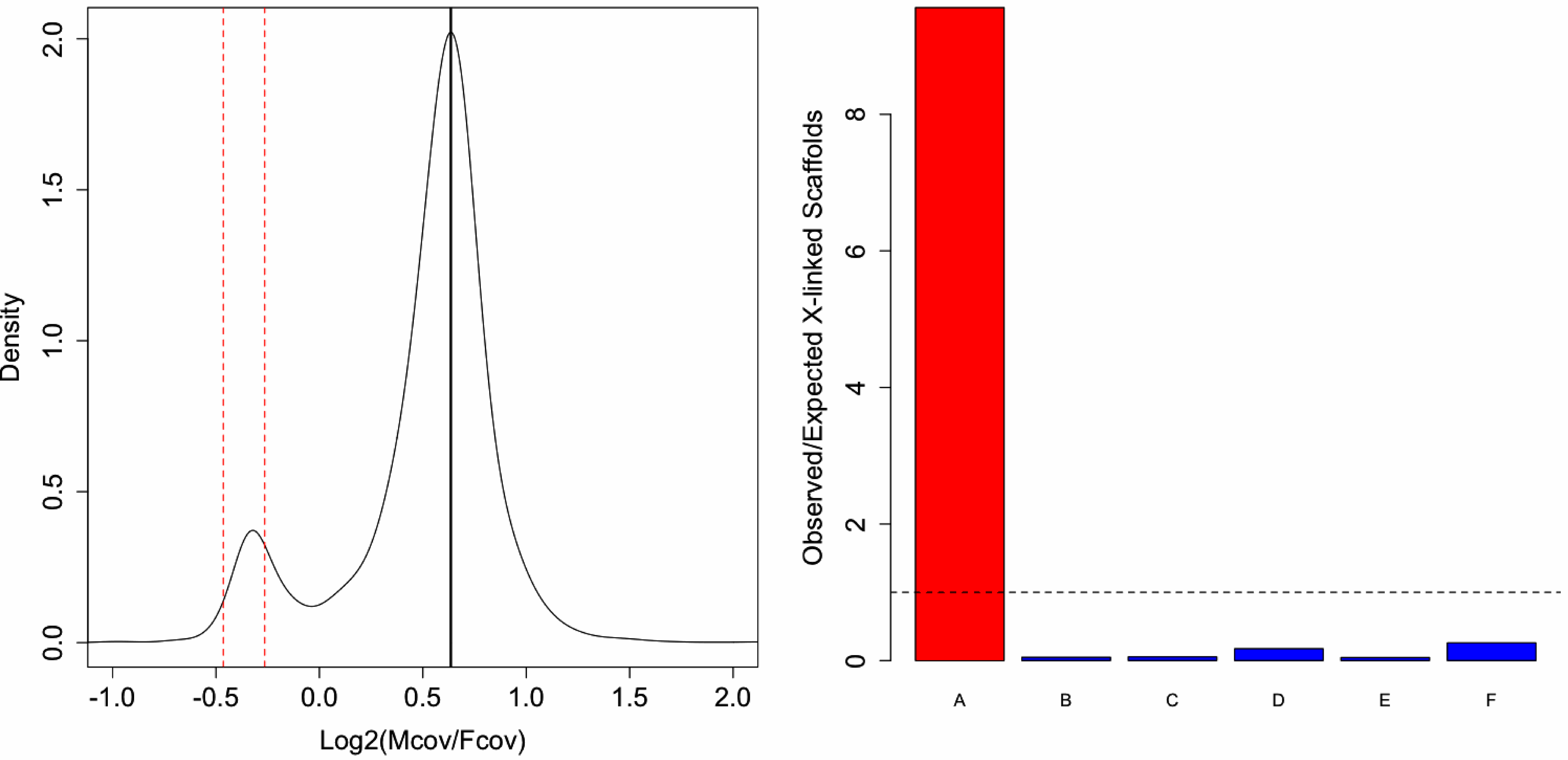

S3.28 Drosophila busckii

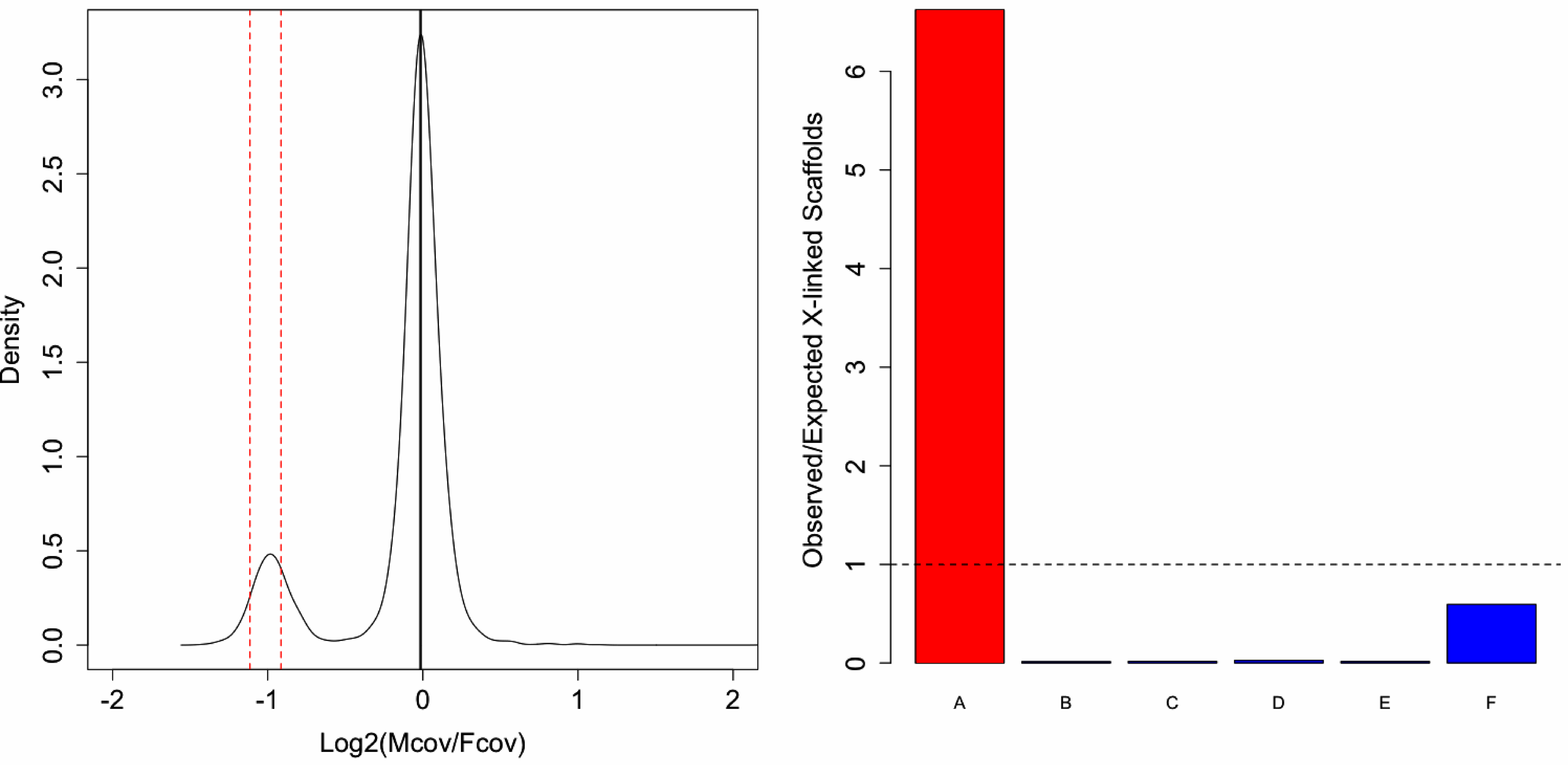

Figure S3

S3.29 Drosophila melanogaster

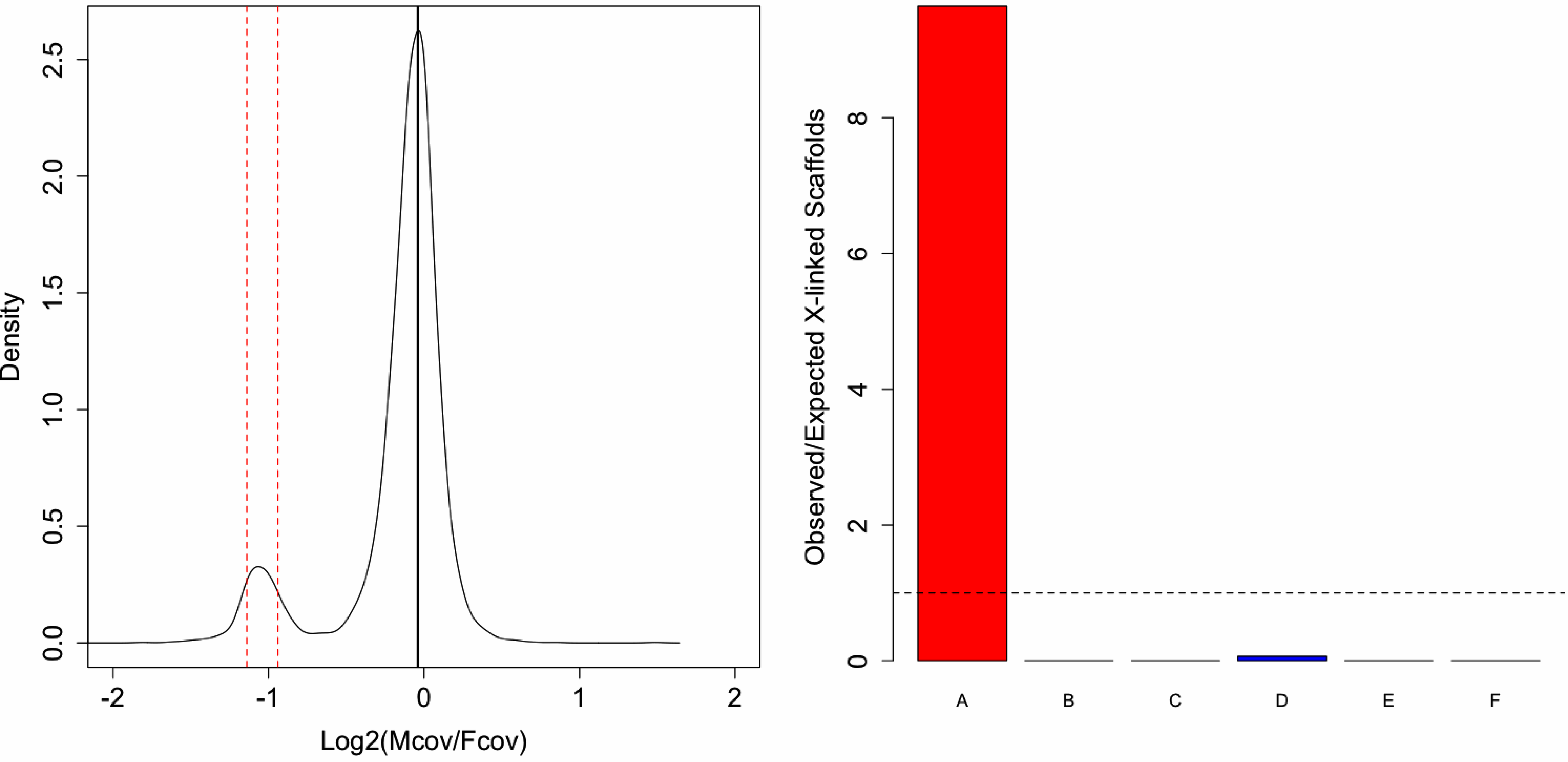

S3.30 Drosophila miranda

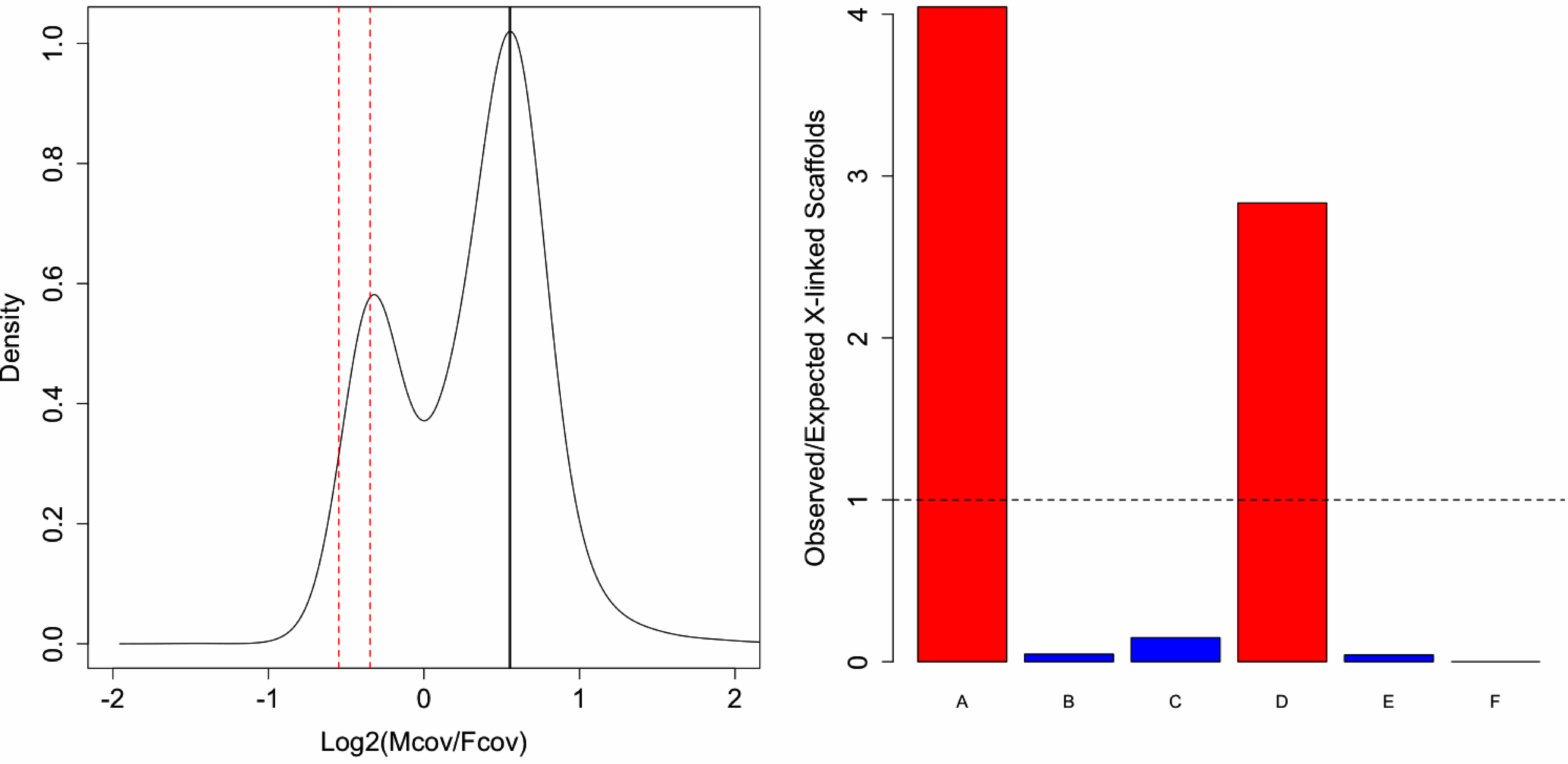

Figure S3

S3.31 Drosophila pseudoobscura

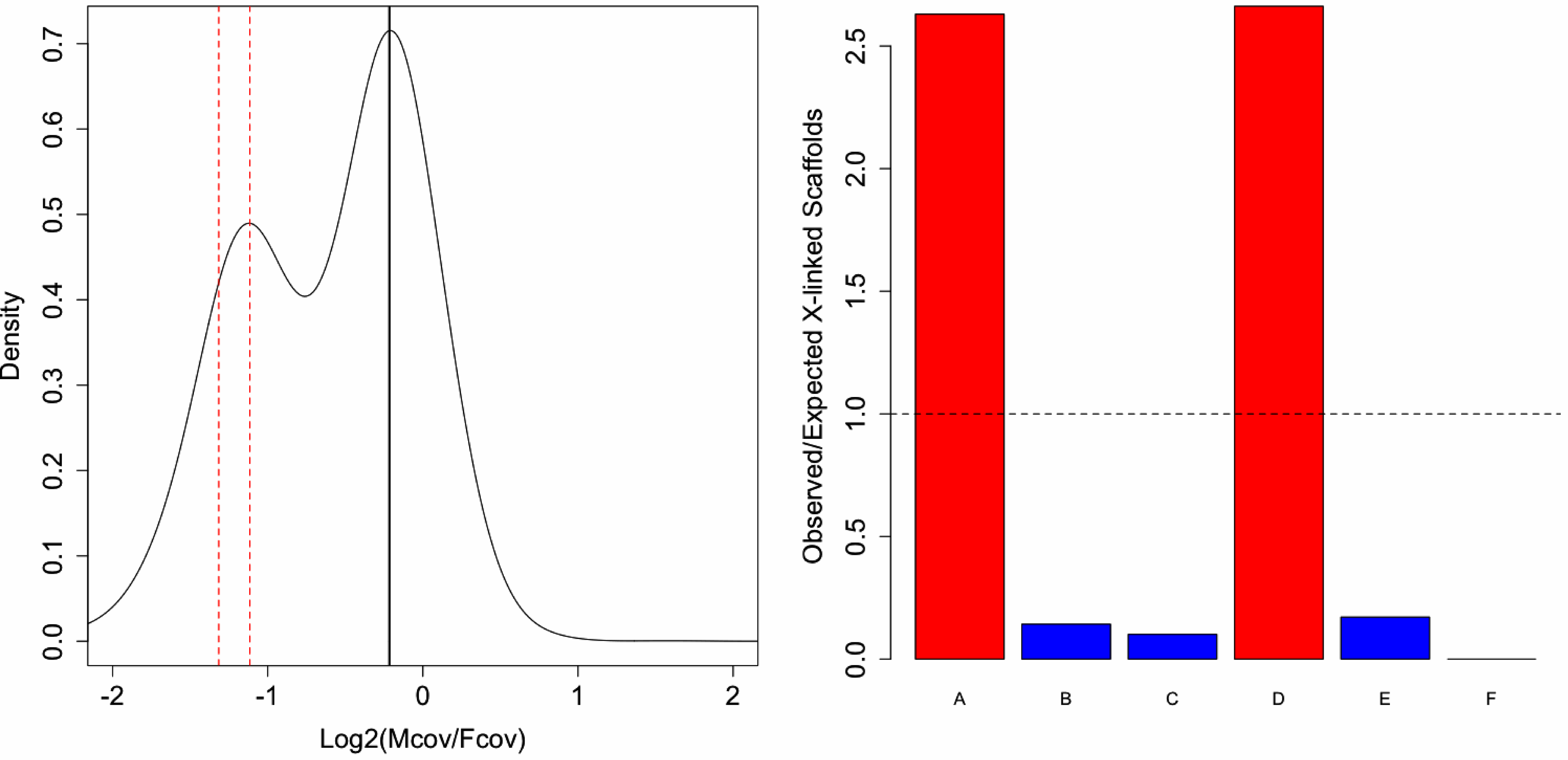

S3.32 Scaptodrosophila lebanonensis

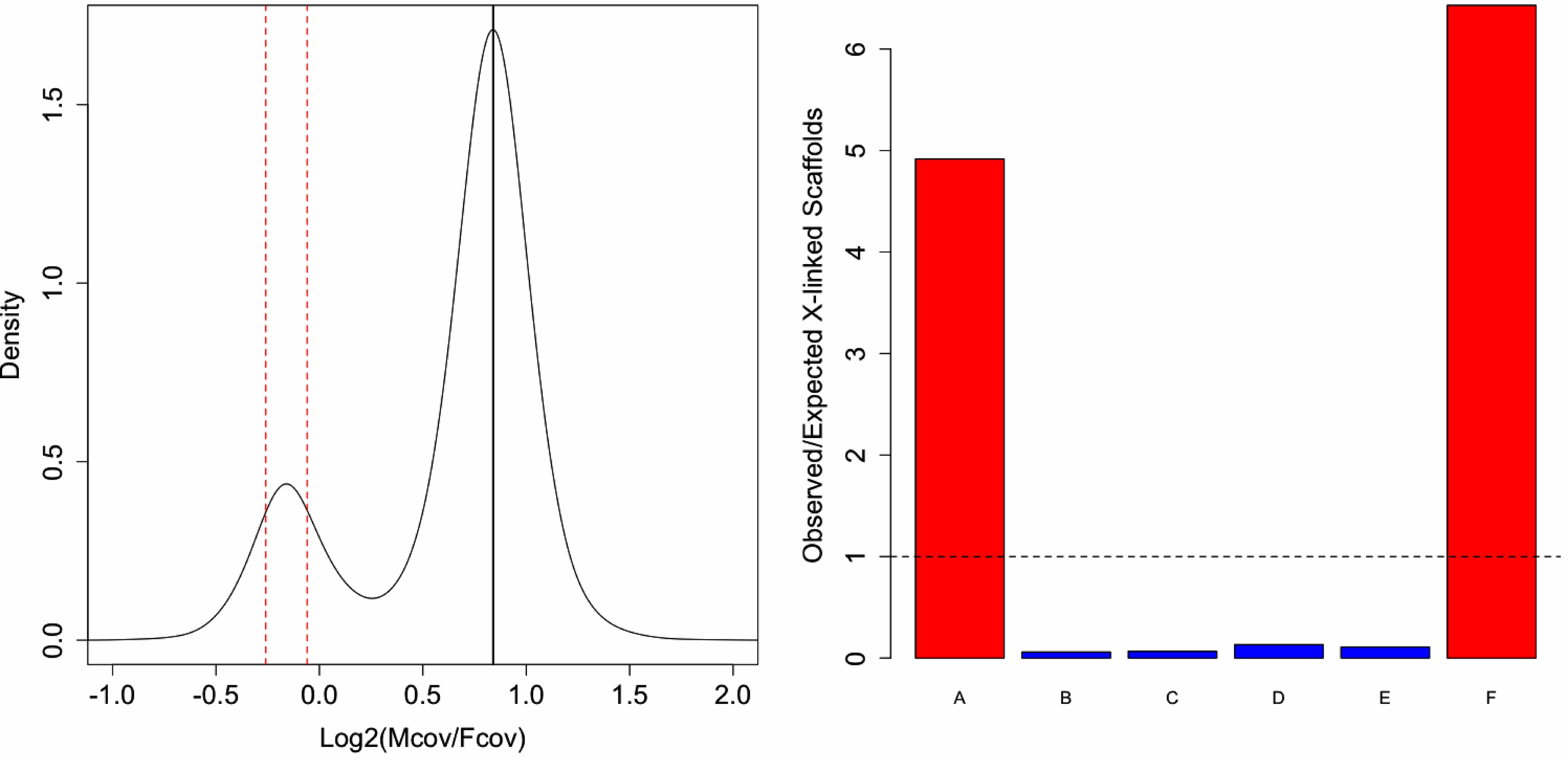

Figure S3

S3.33 Glossina morsitans

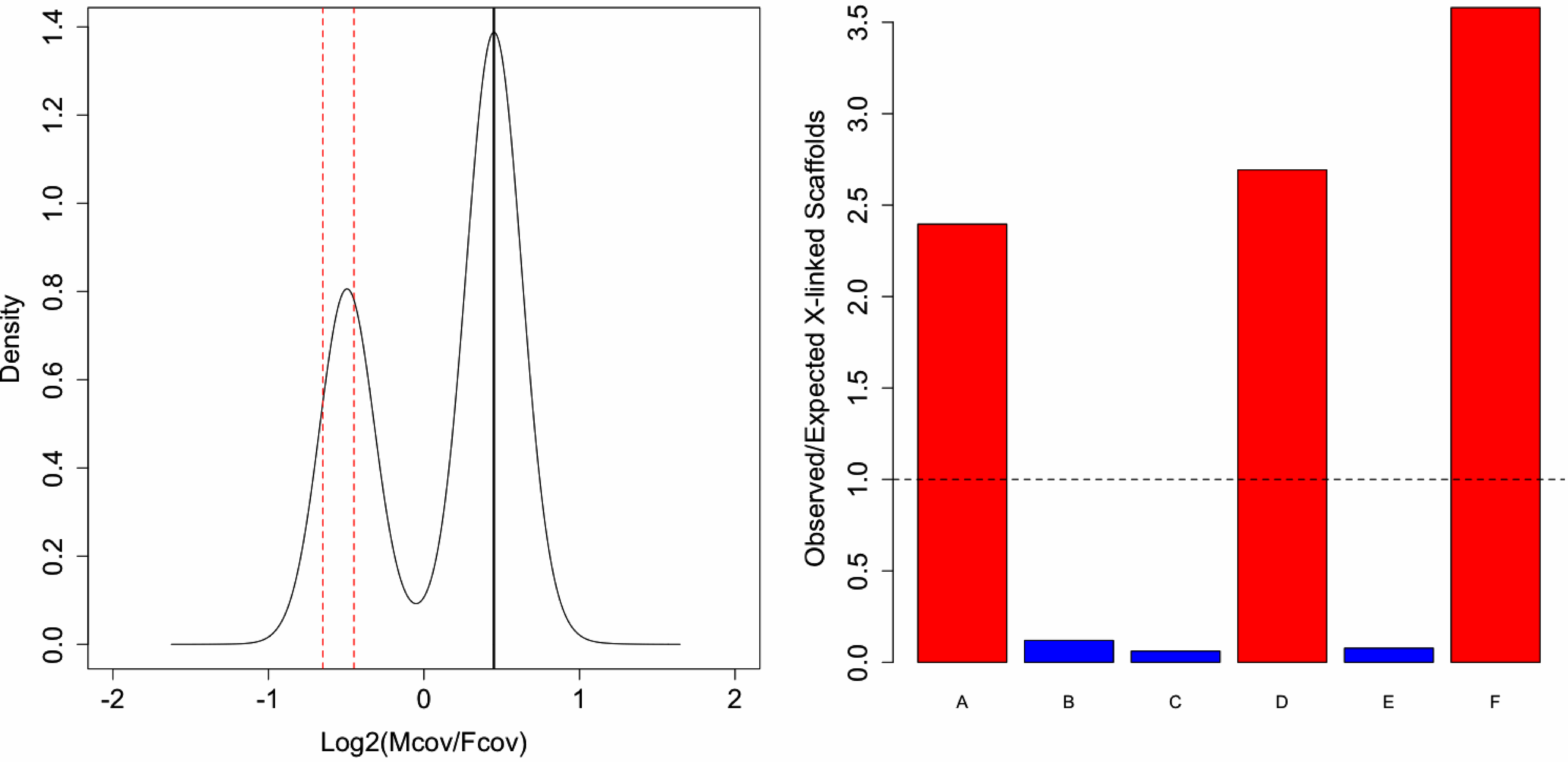

S3.34 Sarcophaga bullata

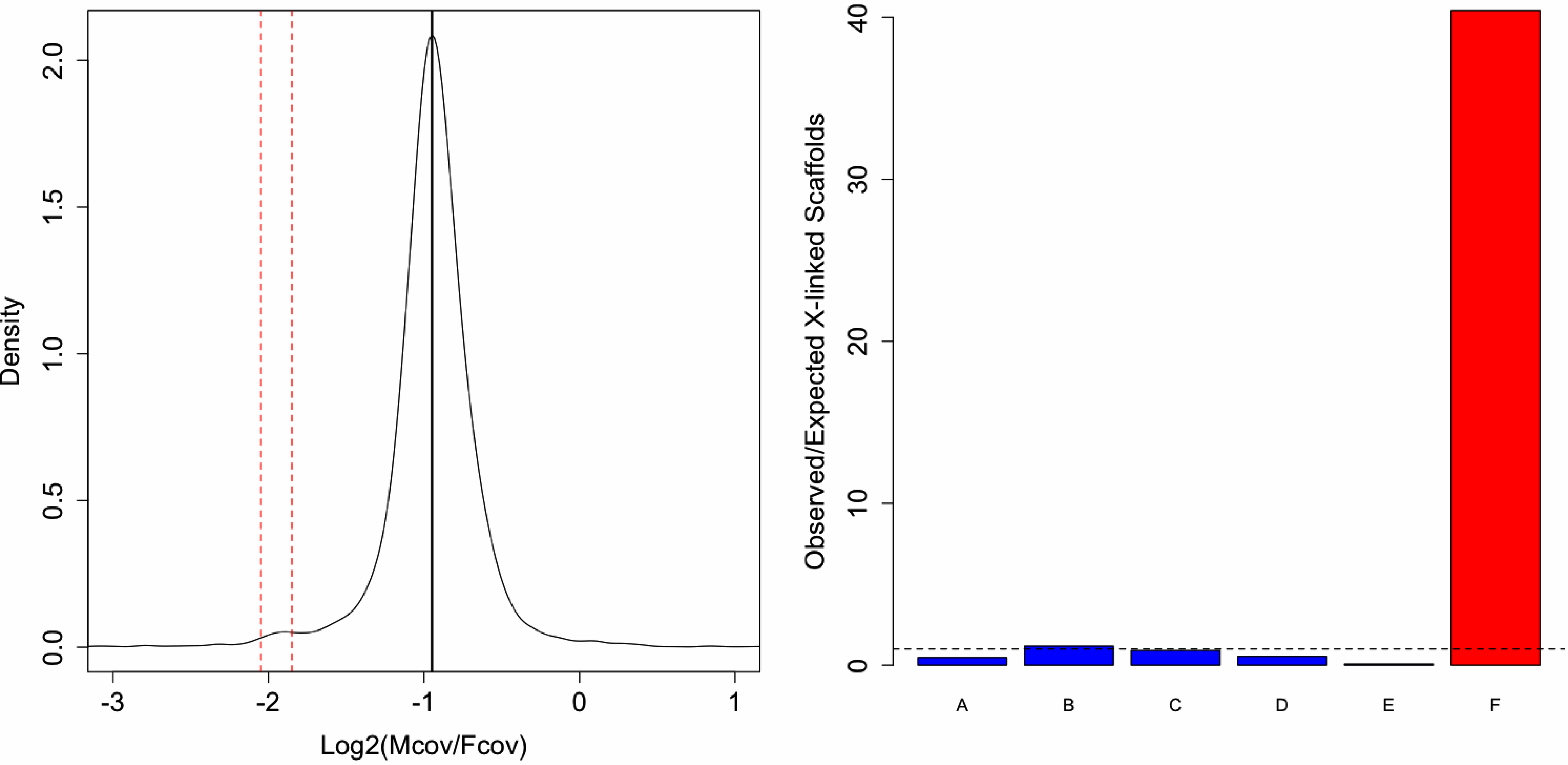

Figure S3

S3.35 Sarcophagidae sp.

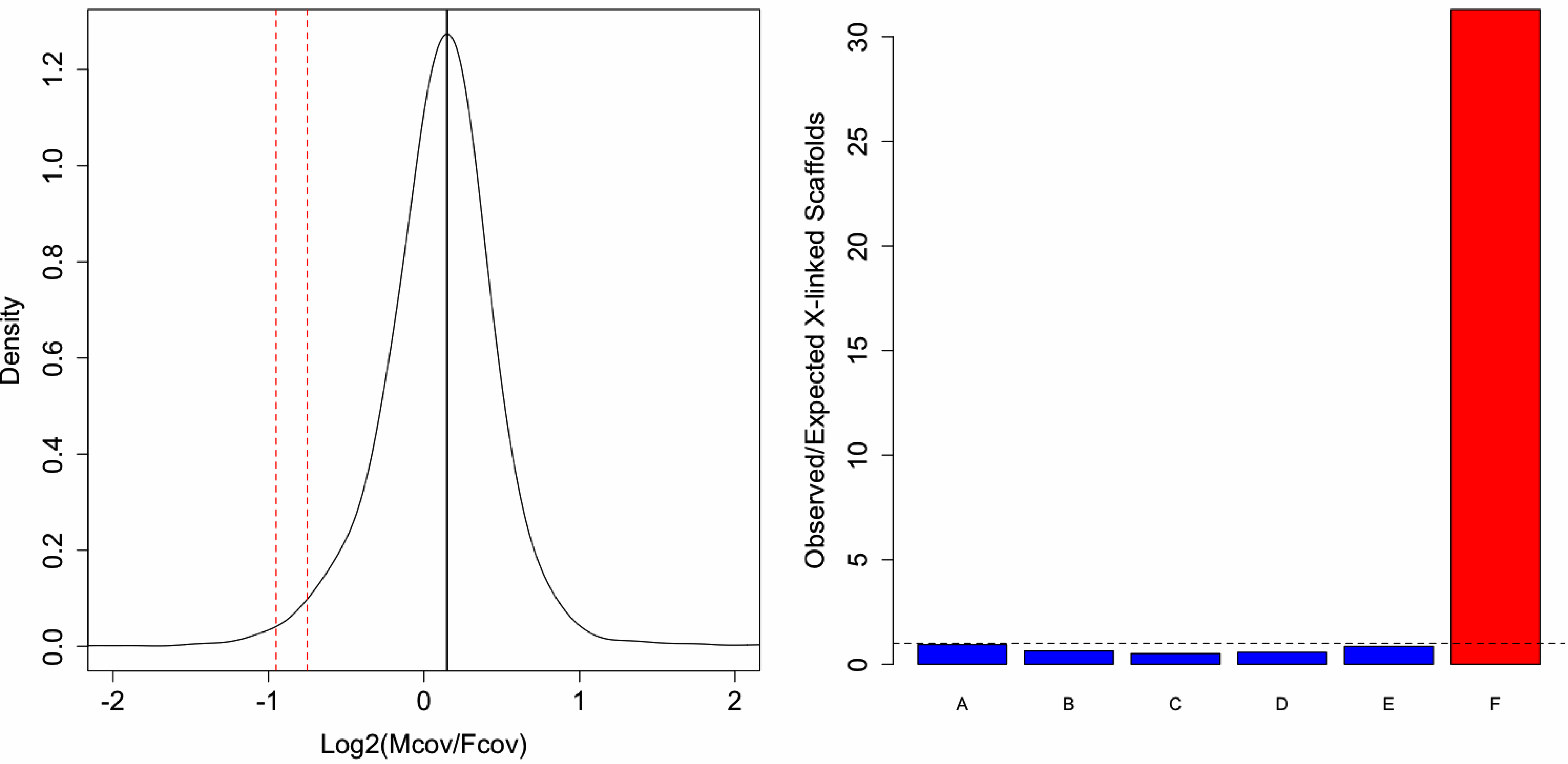

S3.36 Lucilia sericata

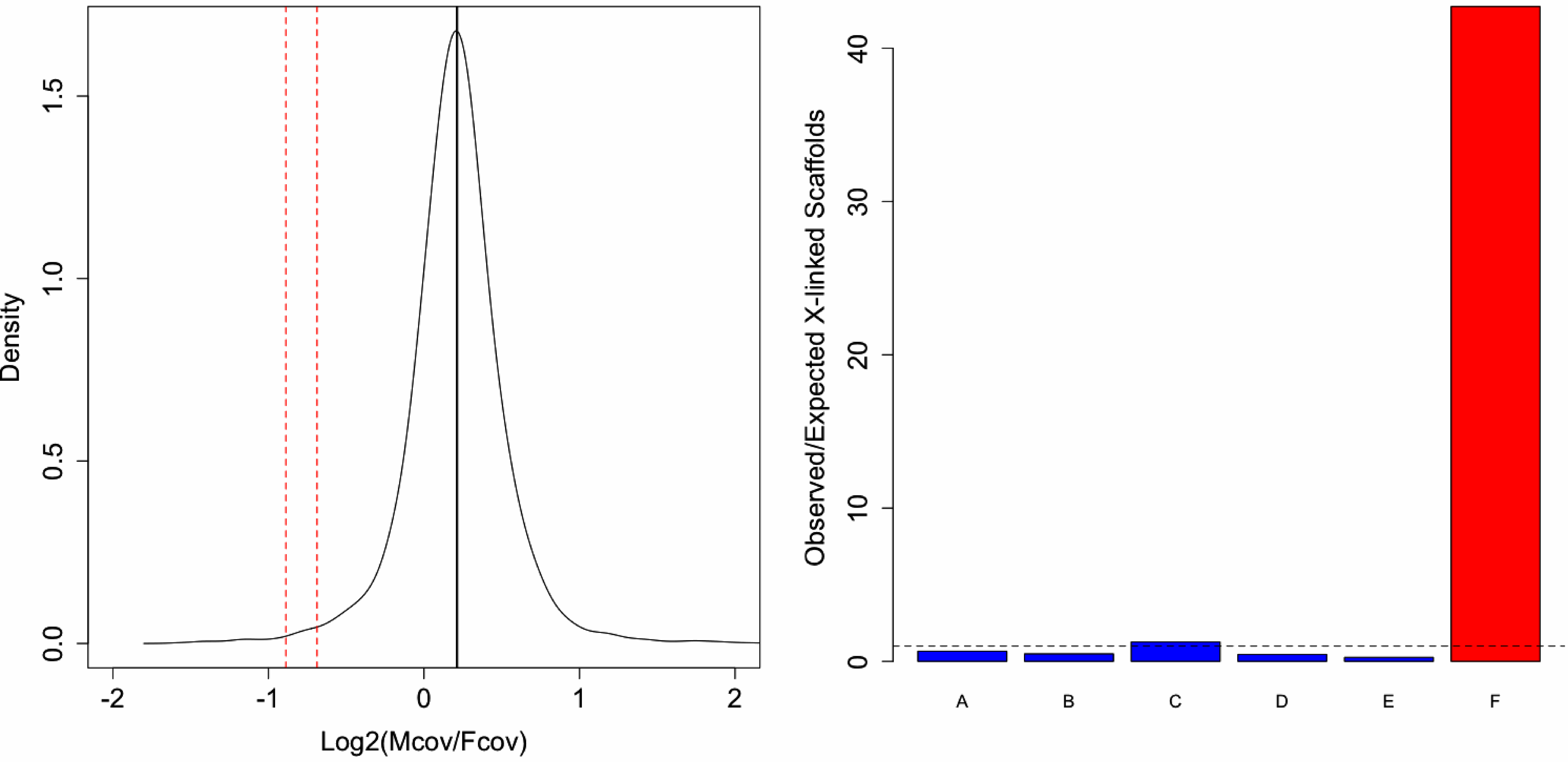

Figure S3

S3.37 Calliphora erythrocephala

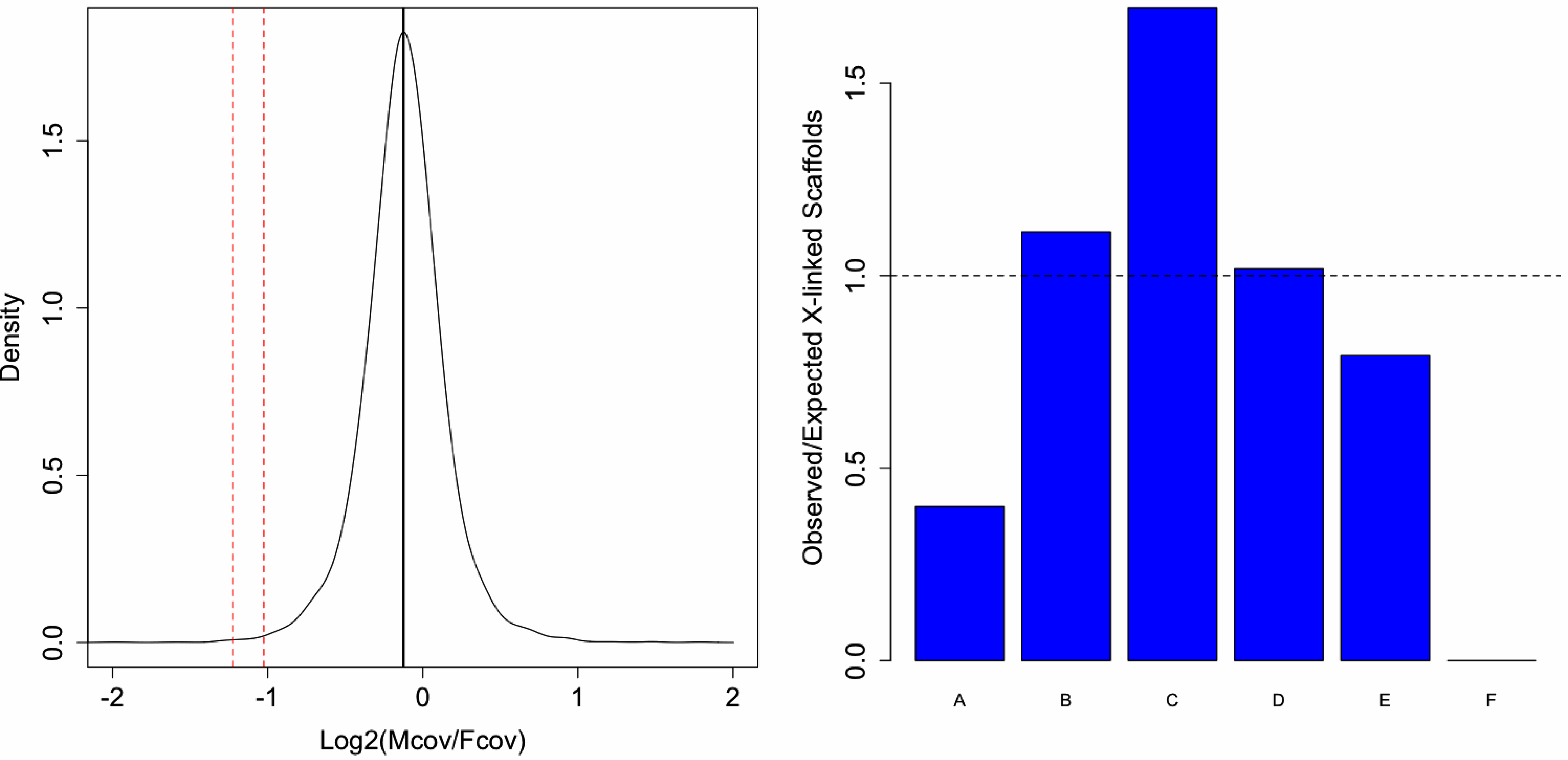

Figure S3
