# Supplementary figures and images for: Numerous Transitions of Sex Chromosomes in Diptera
Source: PLoS Biol. 2015 Apr 16;13(4):e1002078. doi: 10.1371/journal.pbio.1002078 (PMC4400102; doi:10.1371/journal.pbio.1002078)

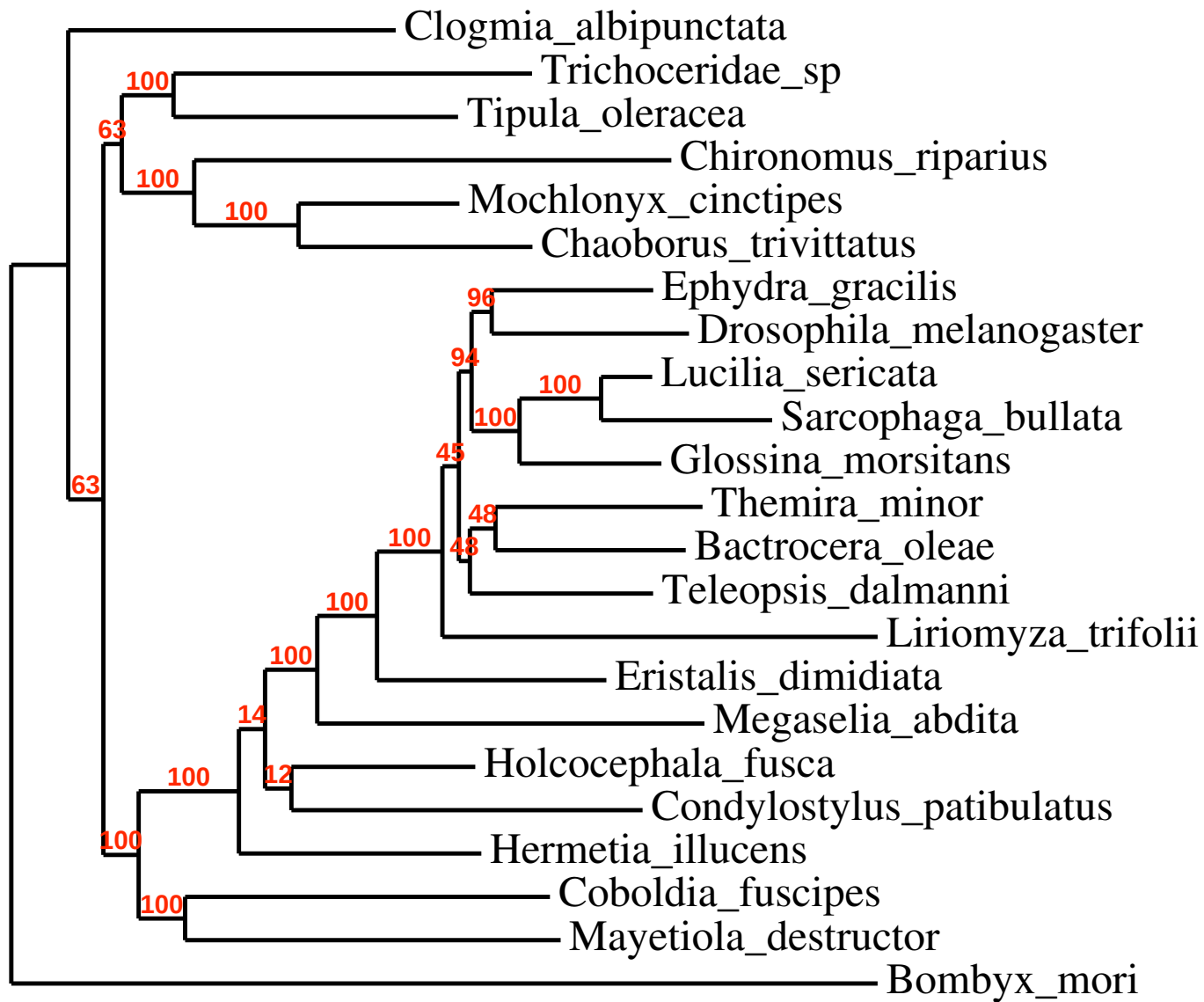

0.2

Supplement: S4 Fig — To minimize the issue of missing data, only one species was used per family, corresponding to the best genome assembly. Drosophila genes were queried against the genomes of all other species to find gene homologs, using a reciprocal best-hit approach. Genes present in all the families were aligned, concatenated, and low-quality sections of the alignment were filtered out. The tree was obtained from this final alignment using PhyML with default parameters, and 100 bootstraps (bootstrap support values are shown next to the nodes). Data to generate the phylogeny are to be found in file “S2 Text” and “S3 Text.” (PDF) [file pbio.1002078.s009.pdf]

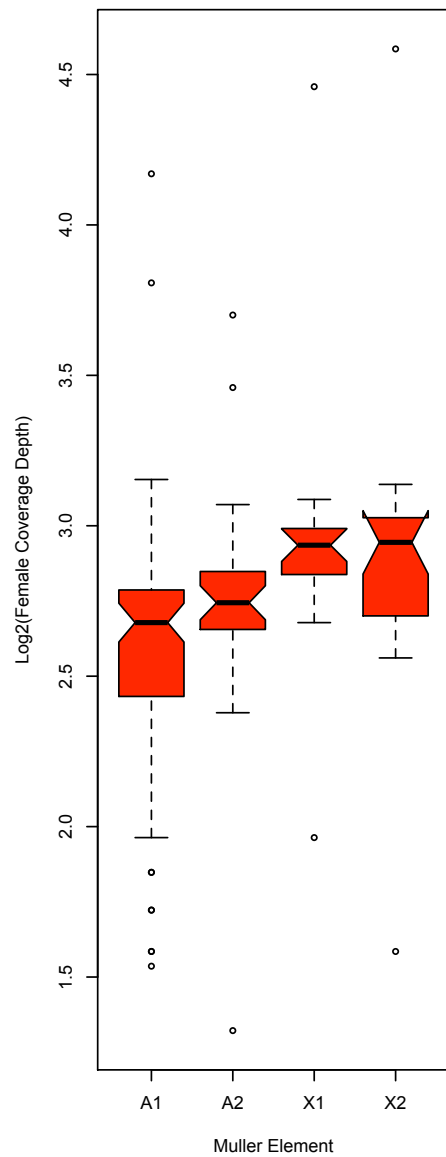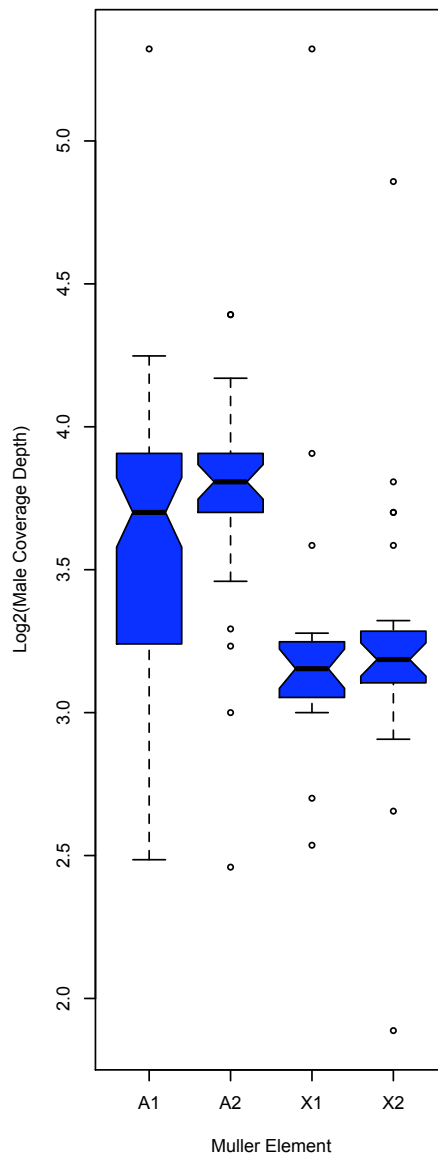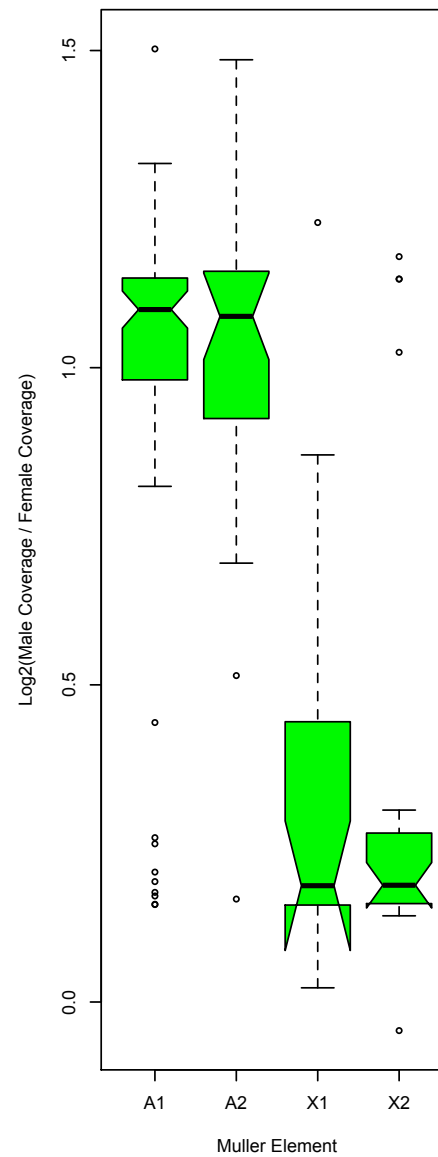

Figure S6

Supplement: S6 Fig — Data to generate this graph are to be found in file “S5 Data.” (PDF) [file pbio.1002078.s011.pdf]

A

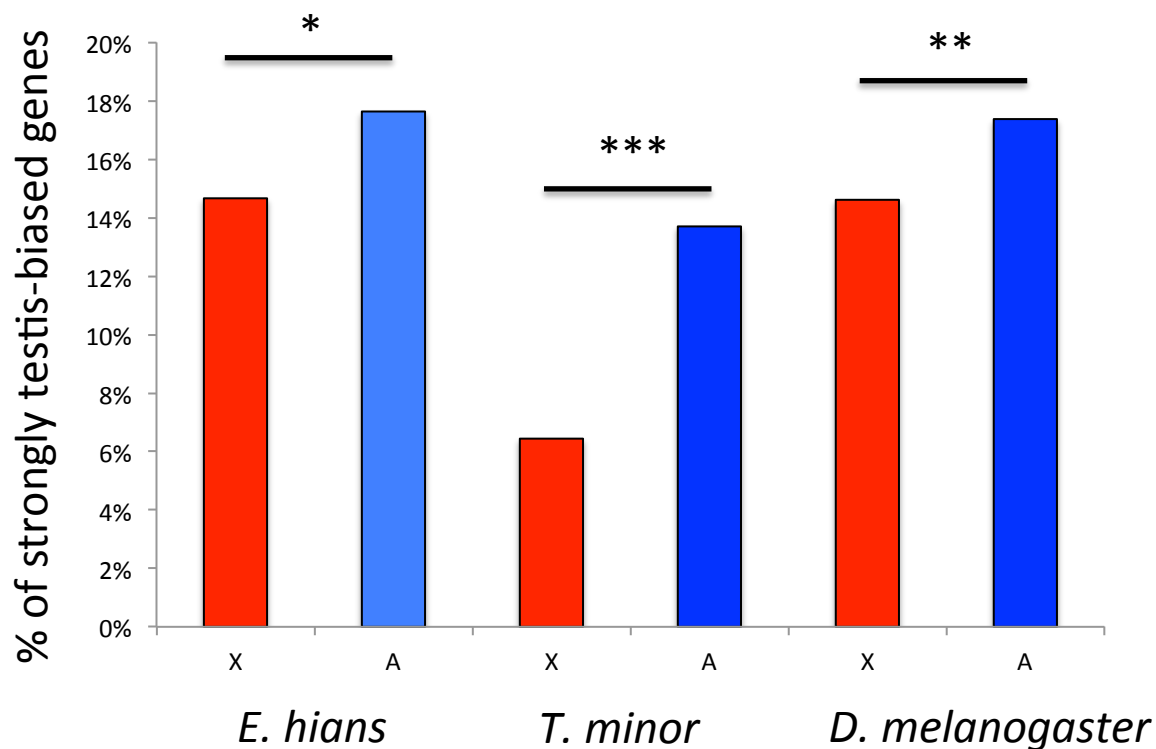

B

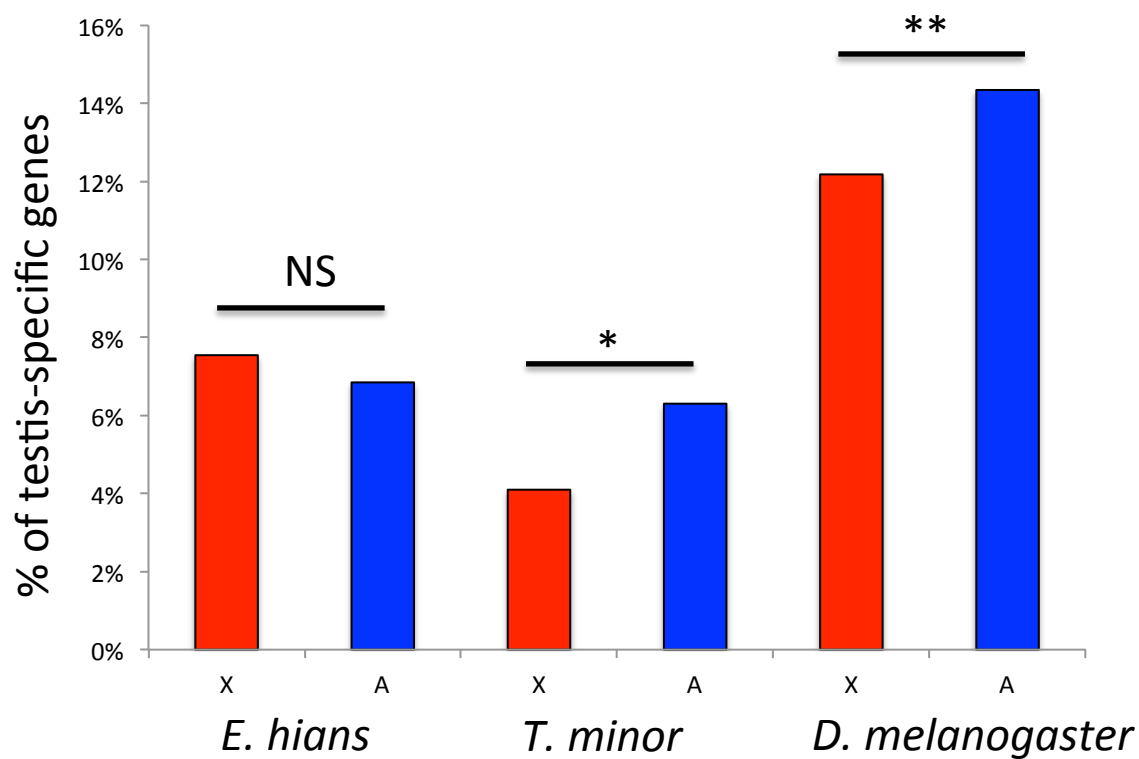

Figure S7

Supplement: S7 Fig — Genes were considered to be strongly testis-biased (A) if their expression was 3-fold higher, and testis-specific (B) if their expression was 10-fold higher in testis than in male head, female head, female body, and ovary. Significant differences in the proportion of testis-biased and testis-specific genes on the X and autosomes were assessed with Pearson Chi-Square tests. Significance levels are represented by asterisks: * p < 0.05, ** p < 0.01, *** p < 0.001. Data to generate this graph are to be found in file “S3 Data.” (PDF) [file pbio.1002078.s012.pdf]
