# Supplementary material for: Numerous Transitions of Sex Chromosomes in Diptera
Source: PLoS Biol. 2015 Apr 16;13(4):e1002078. doi: 10.1371/journal.pbio.1002078 (PMC4400102; doi:10.1371/journal.pbio.1002078)

A) Drosophila miranda

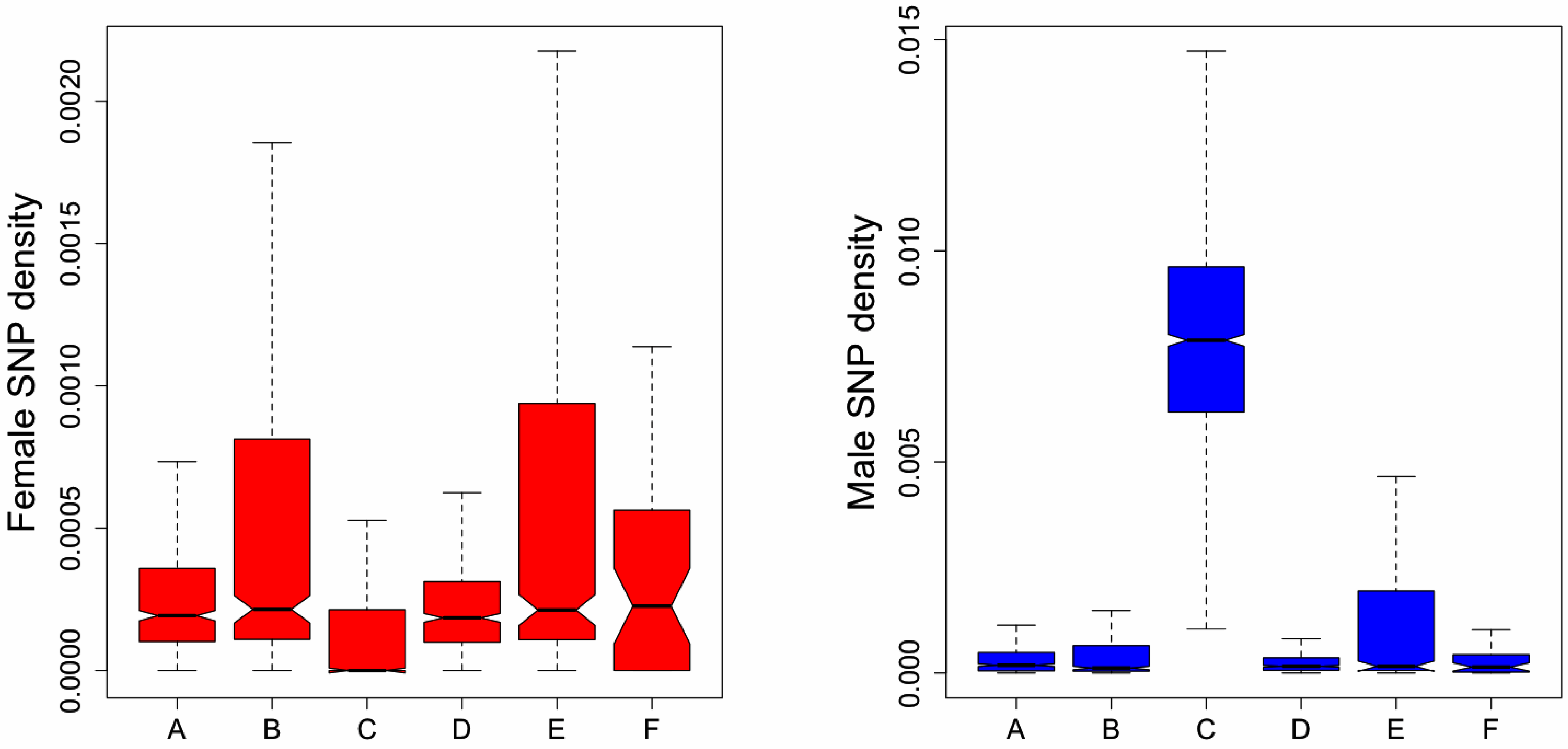

B) Drosophila albomicans

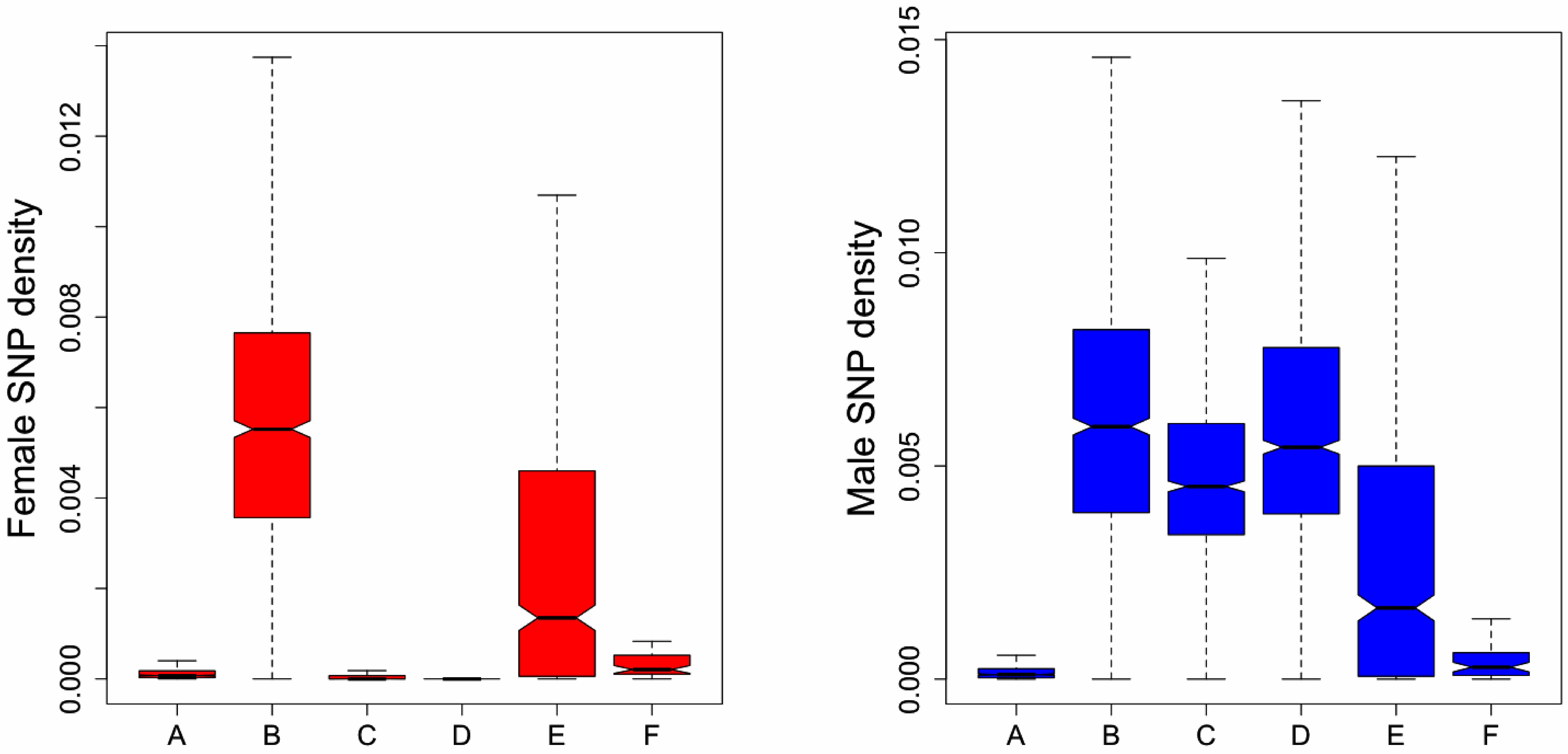

C) Drosophila busckii

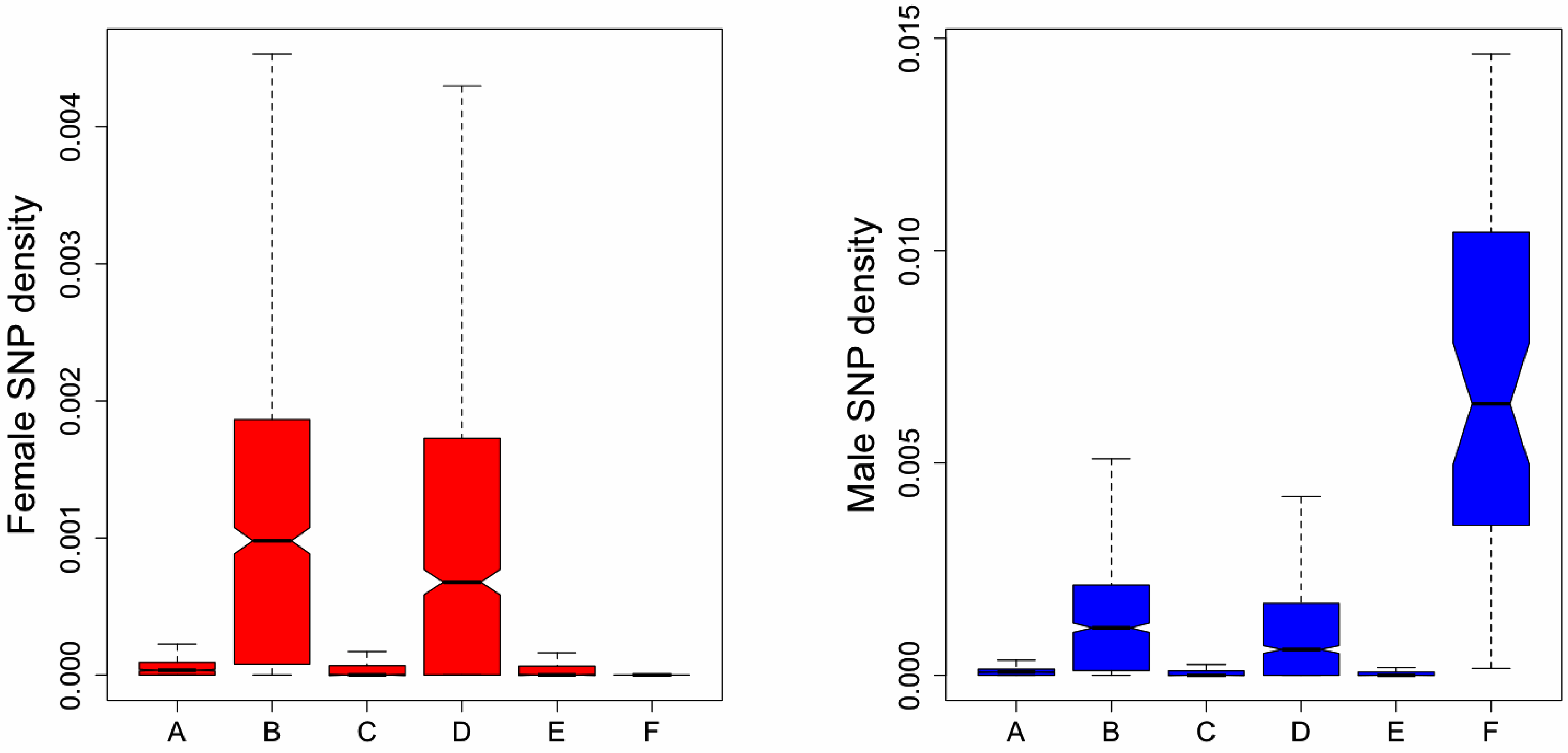

D) Scaptodrosophila lebanonensis

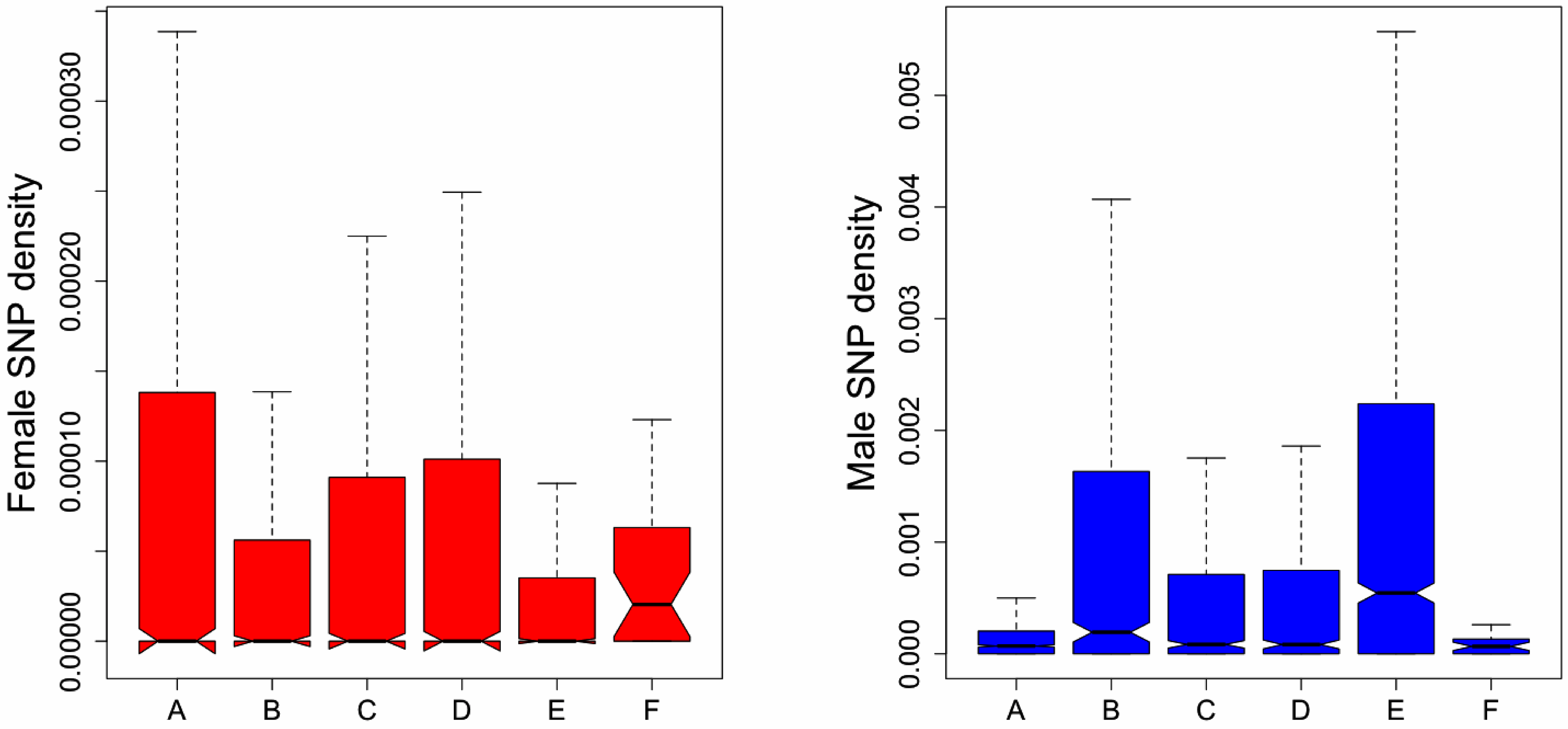

E) Holcocephala fusca

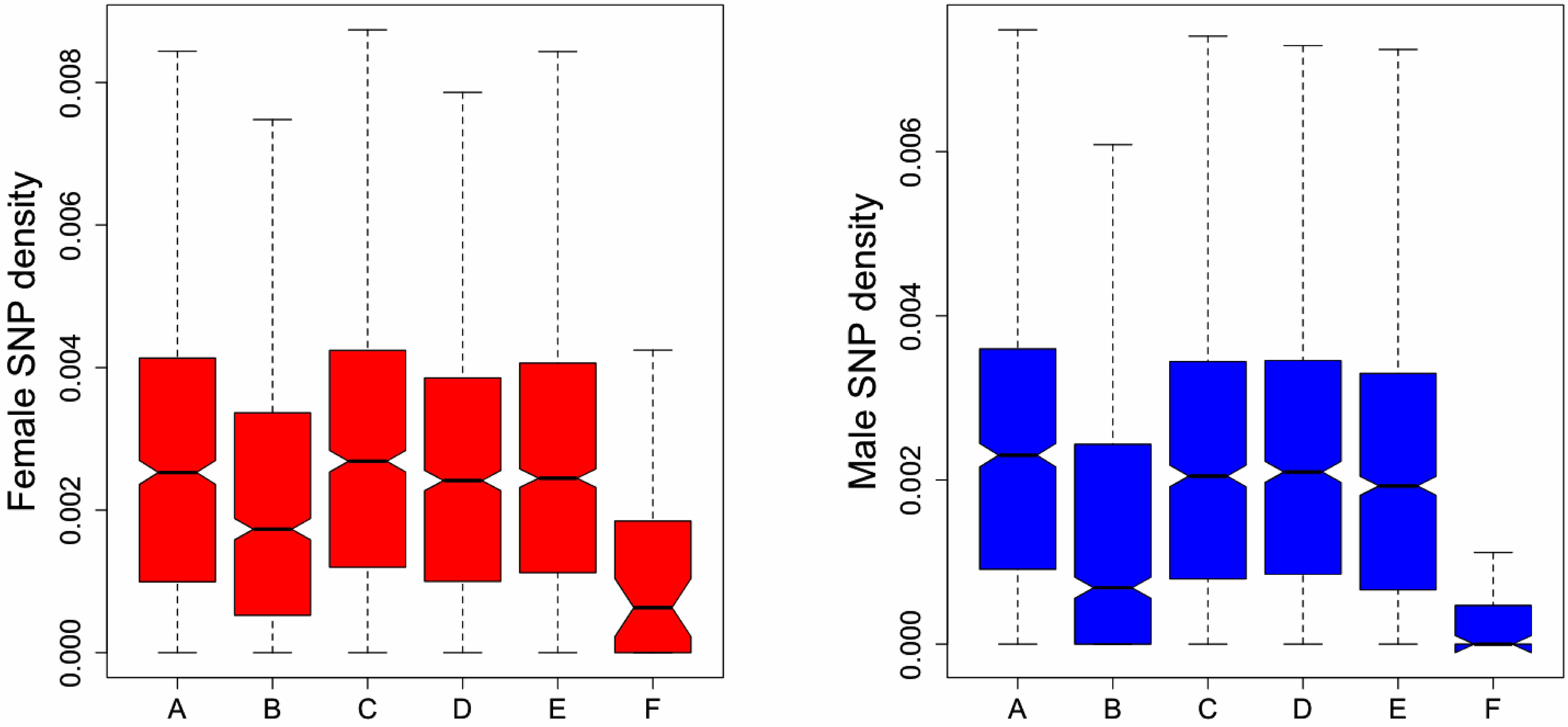

F) Mayetiola destructor

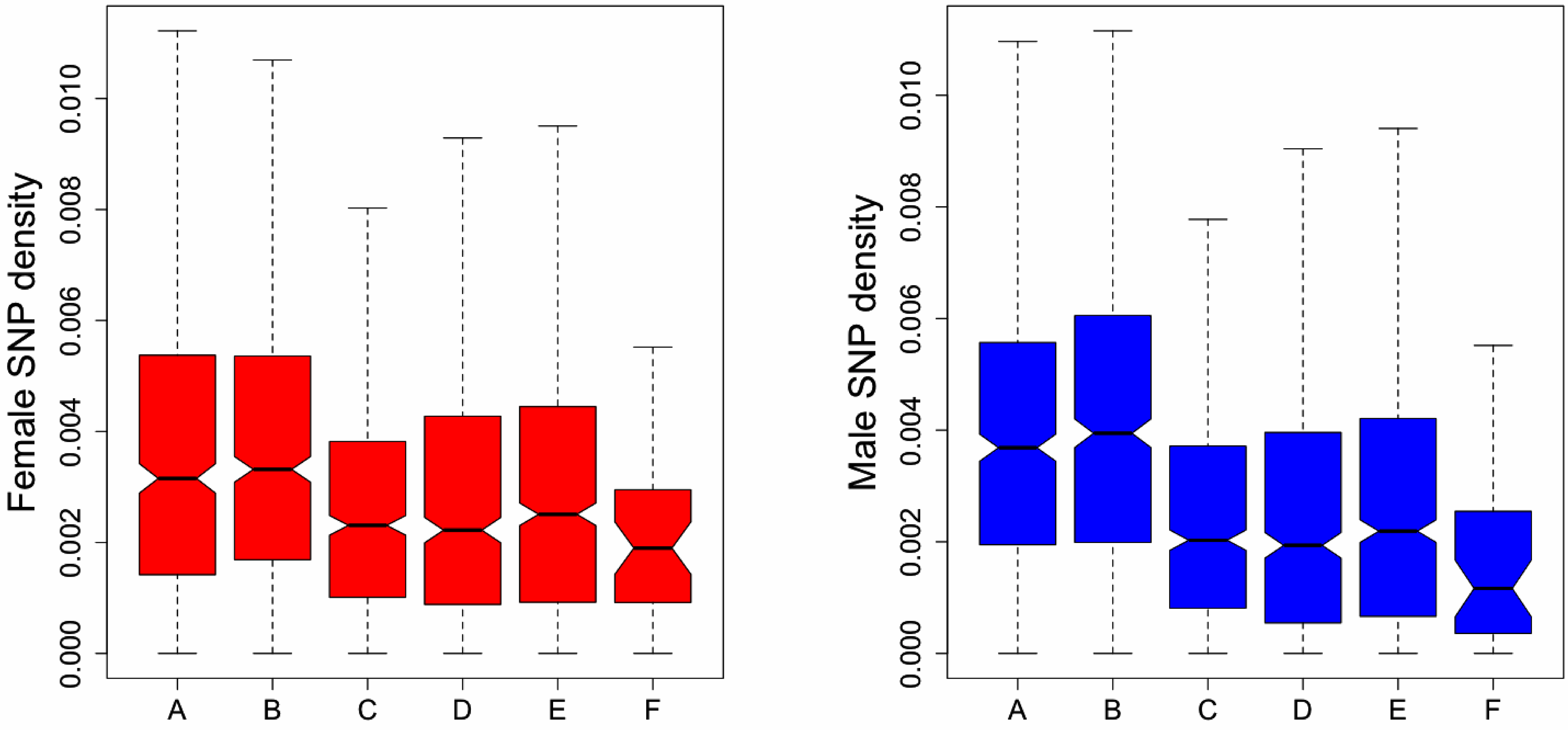

G) *Calliphora erythrocephala*

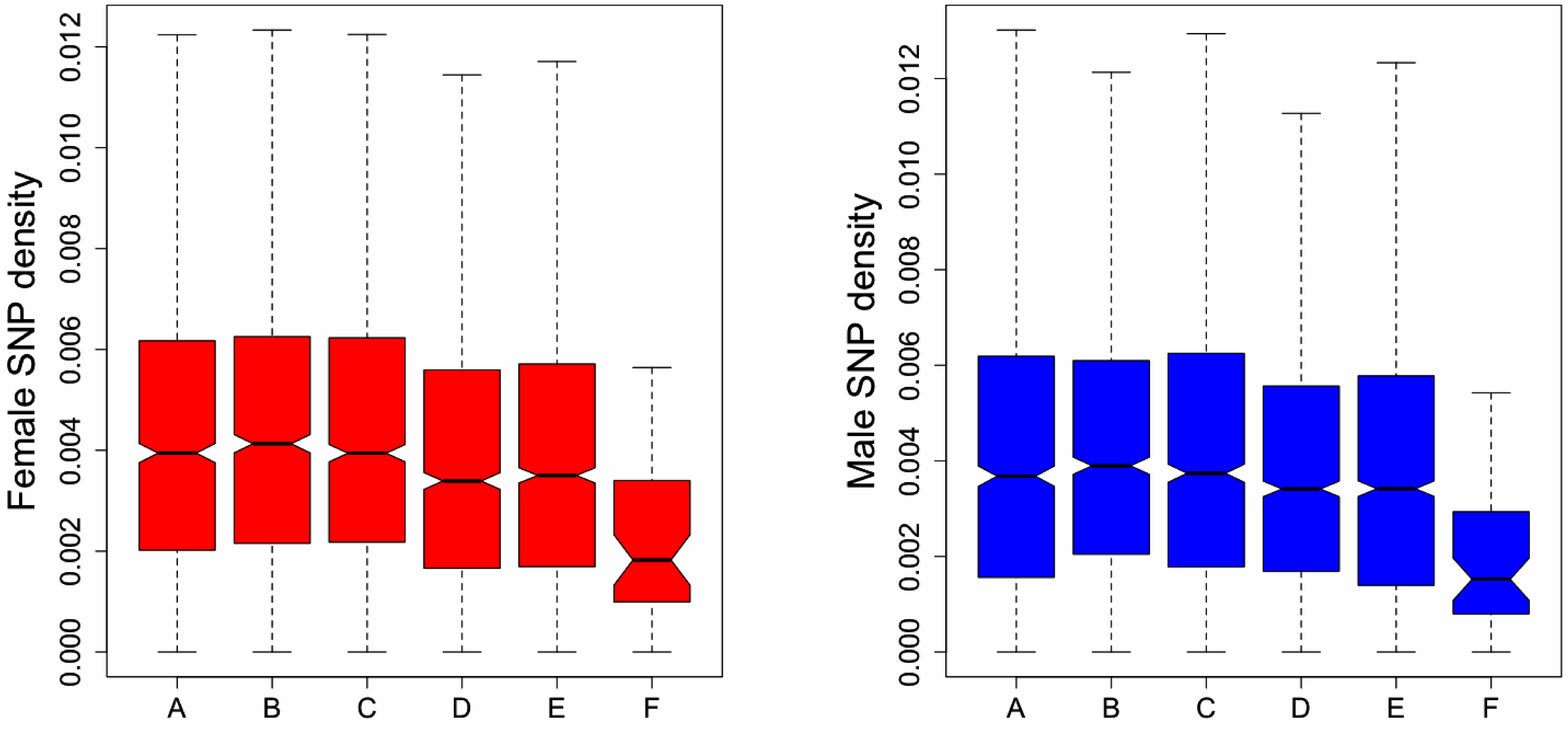

H) *Megaselia abdita*

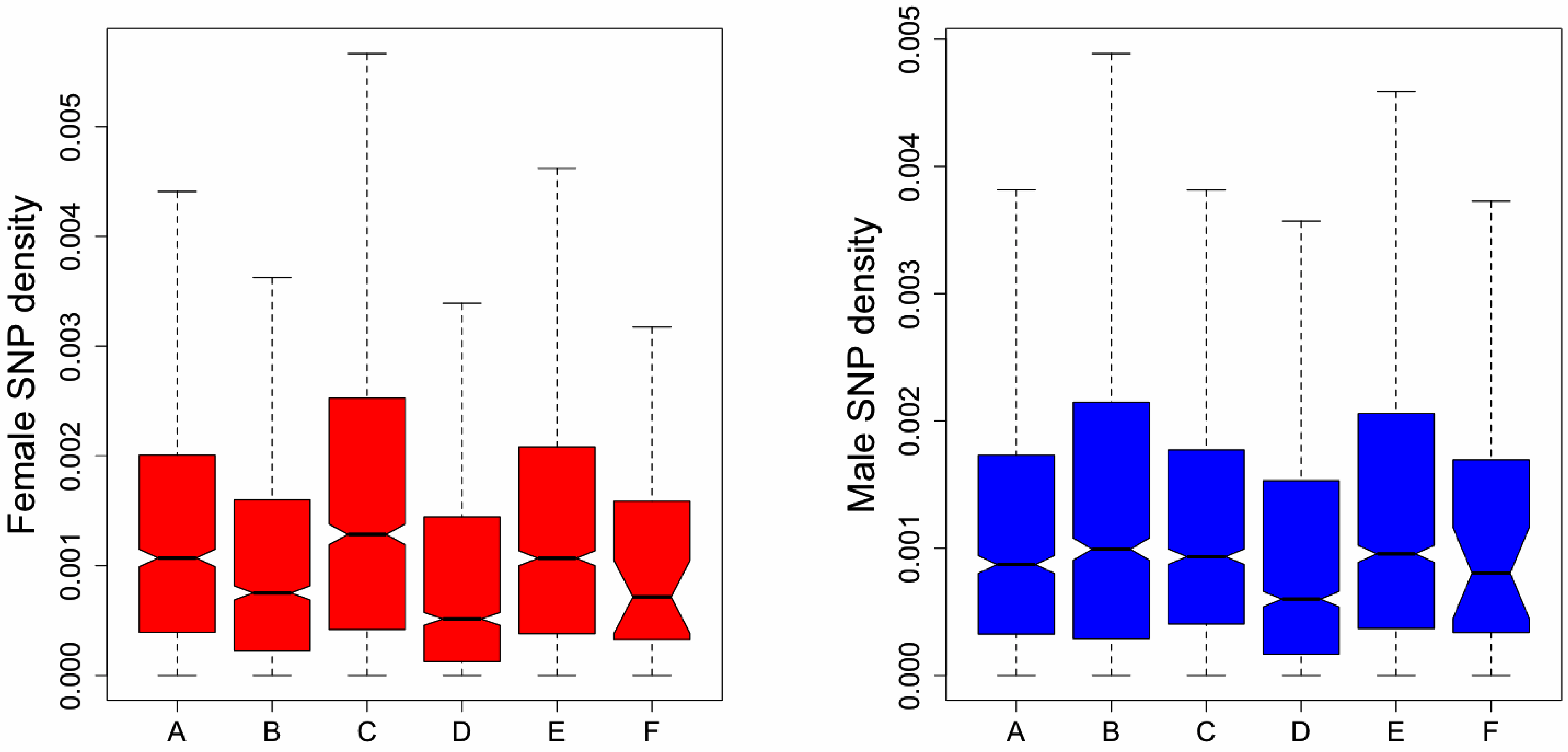

Supplement: S5 Fig — SNP densities are shown for: Drosophila miranda (A), Drosophila albomicans (B), Drosophila busckii (C), Scaptodrosophila lebanonensis (D), Holcocephala fusca (E), Mayetiola destructor (F), Calliphora erythrocephala (G), and Megaselia abdita (H). Data to generate this graph are to be found in file “S2 Data.” (PDF) [file pbio.1002078.s010.pdf]
