# Supplementary material for: Numerous Transitions of Sex Chromosomes in Diptera
Source: PLoS Biol. 2015 Apr 16;13(4):e1002078. doi: 10.1371/journal.pbio.1002078 (PMC4400102; doi:10.1371/journal.pbio.1002078)
Supplement: S1 Table — D. melanogaster genes were located on the M. destructor chromosomal scaffolds using blat (keeping only reciprocal best hits). The number of genes that maps to the different chromosomes of M. destructor is shown for each D. melanogaster Muller element. Significant excesses, detected using Pearson Chi-Square tests, are shown (***,p < 0.0001; * p < 0.05). (DOCX) [file pbio.1002078.s013.docx]

**Table S1.- *Drosophila melanogaster/Mayetiola destructor* synteny table.** *D. melanogaster* genes were located on the *M. destructor* chromosomal scaffolds using blat (keeping only reciprocal best hits). The number of genes that maps to the different chromosomes of *M. destructor* is shown for each *D. melanogaster* Muller element. Significant excesses, detected using Pearson Chi-Square tests, are shown (***, p<0.0001; * p<0.05).

|  | ***D. melanogaster* Muller element** | | | | | |
| --- | --- | --- | --- | --- | --- | --- |
| ***M. destructor***  **chromosome** | **A** | **B** | **C** | **D** | **E** | **F** |
| **A1** | 322*** | 520*** | 144 | 90 | 158 | 8 |
| **A2** | 248*** | 68 | 86 | 161 | 359*** | 4 |
| **X1** | 56 | 60 | 241 | 439*** | 498*** | 30*** |
| **X2** | 57 | 45 | 349*** | 164* | 73 | 6 |
