# Supplementary material for: Numerous Transitions of Sex Chromosomes in Diptera
Source: PLoS Biol. 2015 Apr 16;13(4):e1002078. doi: 10.1371/journal.pbio.1002078 (PMC4400102; doi:10.1371/journal.pbio.1002078)
Supplement: S2 Table — (DOCX) [file pbio.1002078.s014.docx]

**Table S2.- Origin and method of preservation of the specimens used.**

|  | Family | Common name | Provided by | | Material preservation |
| --- | --- | --- | --- | --- | --- |
| **Nematocera Group I** |  |  |  | |  |
| *Tipula oleracea* | Tipulidea | cranefly | Daniel C. Peck | | Ethanol |
| Trichoceridae sp. | Trichoceridae | winter crane fly | Michael W. Perry | | Frozen in ethanol |
|  |  |  |  | |  |
| **Nematocera Group II** |  |  |  | |  |
| *Chironomus riparius* | Chironomidae | harlequin fly | Urs Schmidt-Ott | | Frozen |
| *Chaoborus trivittatus* | Chaoboridae | phantom midge | David L. Woodward | | Frozen |
| *Mochlonyx cinctipes* | Culicidae |  | David L. Woodward | | Frozen |
| *Anopheles gambiae* | Culicidae | African malaria mosquito | David W. Severson | | Ethanol |
| *Aedes aegypti* | Culicidae | yellow fever mosquito | David W. Severson | | Ethanol |
| *Clogmia albipunctata* | Psychodidae | mothfly | Urs Schmidt-Ott | | Frozen |
|  |  |  |  | |  |
| **Nematocera Group III** |  |  |  | |  |
| *Coboldia fuscipes* | Scatopsidae | scavenger fly | Urs Schmidt-Ott | | Frozen |
|  |  |  |  | |  |
| **Nematocera Group IV** |  |  |  | |  |
| *Mayetiola destructor* | Cecidomyiidae | Hessian fly | Jeff Stuart | | Live individuals |
|  |  |  |  | |  |
| **Brachycera** |  |  |  | |  |
| *Hermetia illucens* | Stratiomyidae | black soldier fly | Urs Schmidt-Ott | | Frozen |
| *Megaselia abdita* | Phoridae | humpbacked fly | Urs Schmidt-Ott | | Frozen |
| *Eristalis dimidiata* | Syrphidae | hoverfly | Michael W. Perry | | Ethanol |
| *Holcocephala fusca* | Asioidea | robber fly | Michael W. Perry | | RNA later |
| *Condylostylus patibulatus* | Dolichopodidae | long-legged fly | Michael W. Perry | | Ethanol |
| *Bactrocera oleae* | Tephritidae | olive fly | Collected in Berkeley in 2012. | | Live individuals kept in the lab. |
| *Tephritis californica* | Tephritidae |  | Collected in Berkeley in 2011. | | Flash frozen. |
| *Eutreta diana* | Tephritidae |  | Collected in Anza Borrego in 2011. | | Flash frozen. |
| *Trupanea jonesi* | Tephritidae |  | Collected in Anza Borrego in 2011. | | Flash frozen. |
| *Ephydra hians* | Ephydridae | alkali fly | Collected by Mono Lake in April 2013 | | Flash frozen |
| *Ephydra gracilis* | Ephydridae |  | Jonathan Clark, Salt Lake | | Frozen |
| *Scaptodrosophila lebanonensis* | Drosophilidae |  | UCSD stock center | | Live individuals |
| *Drosophila miranda* | Drosophilidae |  | UCSD stock center |  | Live individuals |
| *Drosophila melanogaster* | Drosophilidae |  | UCSD stock center |  | Live individuals |
| *Drosophila busckii* | Drosophilidae |  | Jan Larrson | | Live individuals |
| *Drosophila albomicans* | Drosophilidae |  | UCSD stock center | | Live individuals |
| *Phortica variegata* | Drosophilidae | zoophilic fruitfly | Ilona Kadow, Italy | | Frozen |
| *Teleopsis dalmanni* | Diopsidae | stalk-eyed fly | Gerald S. Wilkinson | | Frozen |
| *Sphyracephala brevicornis* | Diopsidae | Short-horned Ankle-headed Fly | Karl Hillig | | Live individuals |
| *Themira minor* | Sepsidae | black scavenger fly | Collected in Sacramento in 2012 and 2013. | | Live individuals kept in the lab. |
| *Liriomyza trifolii* | Agromyzidae | American serpentine leafminer | Michael Parrella | | Live individuals |
| *Sarcophaga bullata* | Sarcophagidae | grey flesh fly | Carolina Biological | | Live individuals kept in the lab. |
| Sarcophagidae *sp* | Sarcophagidae |  | Collected in San Francisco, 2012. | | Live individuals |
| *Glossina morsitans* | Glossinidae | tsetse fly | Serap Aksoy | | DNA |
| *Lucilia sericata* | Calliphoridae | common green bottle fly | Aaron Tarone | | Frozen |
| *Calliphora erythrocephala* | Calliphoridae | blow fly | Alina Kokhanenko | | Ethanol |
