# Supplementary material for: Numerous Transitions of Sex Chromosomes in Diptera
Source: PLoS Biol. 2015 Apr 16;13(4):e1002078. doi: 10.1371/journal.pbio.1002078 (PMC4400102; doi:10.1371/journal.pbio.1002078)
Supplement: S1 Text — (DOCX) [file pbio.1002078.s015.docx]

**Text S1.** R code used to detect differentiated X-linked elements (1), Z-linked elements (2) and X-linked elements in M. destructor, where the majority of the genome is X-linked.

The coverage input files for each are in the following format:

scaf Fper Fcov Mper Mcov chrom

C12817355 96 13 99 18 3L

C12817697 1e+02 12 98 11 3L

C12817867 98 14 98 13 3L

Where scaf is the scaffold name, Fper is the percentage of the scaffold that has coverage in the female, Fcov is the coverage depth in the female, Mper is the percentage of the scaffold that has coverage in the male, Fcov is the coverage depth in the male, chrom is the Muller element that the scaffold has been assigned to (following the D. melanogaster nomenclature).

1. Code for male-heterogamety (finds X-linked elements)

#first, let's read the coverage table, plot the Mcov/Fcov density

cov2<-read.table("/Users/Beatriz/Documents/dosage_comp/2014Sept_Revisions1_PlosBio/NewCoverage/CovFiles/Megaselia_FMcov_location.txt", head=T)

cov<-subset(cov2, chrom!="U")

dev.new(width=15, height=8)

par(mfrow=c(1,2))

plot(density(log(cov$Mcov/cov$Fcov, base=2), adjust=2, na.rm = T), xlim=c(-2,2), main="", xlab="Log2(Mcov/Fcov)", cex.axis=1.5, cex.lab=1.5)

print(density(log(cov$Mcov/cov$Fcov, base=2), adjust=2, na.rm = T))

#--> find the maximum peak in the density distribution, and its corresponding x (xmax, which should correspond to the median Mcov/Fcov for the autosomes)

bubba <- data.frame(first=(density(log(cov$Mcov/cov$Fcov, base=2), adjust=2, na.rm = T)$x), second=(density(log(cov$Mcov/cov$Fcov, base=2), adjust=2, na.rm = T)$y))

xmax<-(subset(bubba, second==max(density(log(cov$Mcov/cov$Fcov, base=2), adjust=2, na.rm = T)$y))$first)

abline(v=xmax, lwd=2)

#--> that gives us the x-coordinate for our main peak

#so we can calculate the x-coordinate for our minor (X) peak:

xcoord<-(xmax-1)

abline(v=(xcoord-0.1), col="red", lty=2)

abline(v=(xcoord+0.1), col="red", lty=2)

#(or +1 for ZW)

xcandidates=subset(cov, (log(cov$Mcov/cov$Fcov, base=2))>((xcoord)-0.1) & (log(cov$Mcov/cov$Fcov, base=2))<((xcoord)+0.1))

#first we put all these values in a big data frame

chix<-aggregate(data.frame(count = xcandidates$chrom), list(value = xcandidates$chrom), length)

chiall<-aggregate(data.frame(count = cov$chrom), list(value = cov$chrom), length)

chi<-merge(chix, chiall, by="value", all=T)

chi[is.na(chi)] <- 0

#then we make the columns that we need to test each chromosome: x, a, controlx (how many are on X for other arms), controla (how many are on A for other chromosomes)

chi$x<-chi$count.x

chi$a<-(chi$count.y-chi$x)

chi$controlx<-(sum(chi$x)-chi$x)

chi$controla<-(sum(chi$a)-chi$a)

#then we get pvalues and plot barplot

#chi$pval<-chisq.test(matrix(c(chi$x, chi$a, chi$controlx, chi$controla),ncol=2), correct=T)$p.value

chrom2L<-fisher.test(rbind(c(subset(chi, value=="2L")$x, subset(chi, value=="2L")$a), c(subset(chi, value=="2L")$controlx, subset(chi, value=="2L")$controla)))$p.value

chrom2R<-fisher.test(rbind(c(subset(chi, value=="2R")$x, subset(chi, value=="2R")$a), c(subset(chi, value=="2R")$controlx, subset(chi, value=="2R")$controla)))$p.value

chrom3L<-fisher.test(rbind(c(subset(chi, value=="3L")$x, subset(chi, value=="3L")$a), c(subset(chi, value=="3L")$controlx, subset(chi, value=="3L")$controla)))$p.value

chrom3R<-fisher.test(rbind(c(subset(chi, value=="3R")$x, subset(chi, value=="3R")$a), c(subset(chi, value=="3R")$controlx, subset(chi, value=="3R")$controla)))$p.value

chrom4<-fisher.test(rbind(c(subset(chi, value=="4")$x, subset(chi, value=="4")$a), c(subset(chi, value=="4")$controlx, subset(chi, value=="4")$controla)))$p.value

chromX<-fisher.test(rbind(c(subset(chi, value=="X")$x, subset(chi, value=="X")$a), c(subset(chi, value=="X")$controlx, subset(chi, value=="X")$controla)))$p.value

pvals<-c(chrom2L, chrom2R, chrom3L, chrom3R, chrom4, chromX)

chi$pval<-pvals

chi$Xpect<-(chi$x/(((chi$controlx+chi$x)/(chi$x+chi$a+chi$controlx+chi$controla))*(chi$count.y)))

chi$col<-4

chi$col[chi$pval<0.01 & chi$Xpect>1] <- 2

#order by element before plotting

chi$Muller<-c("B", "C", "D", "E", "F", "A")

chi2<-chi[order(chi$Muller),]

print(chi2)

barplot(chi2$Xpect, names=c("A", "B", "C", "D", "E", "F"), col=chi2$col, ylab="Observed/Expected X-linked Scaffolds", cex.axis=1.5, cex.lab=1.5)

abline(h=1, lty=2)

2. Code for female-heterogamety (finds Z-linked elements)

#first, let's read the coverage table, plot the Mcov/Fcov density

cov2<-read.table("/Users/Beatriz/Documents/dosage_comp/2014Sept_Revisions1_PlosBio/NewCoverage/CovFiles/Trupanea_FMcov_location.txt", head=T)

cov<-subset(cov2, chrom!="U")

dev.new(width=15, height=8)

par(mfrow=c(1,2))

plot(density(log(cov$Mcov/cov$Fcov, base=2), adjust=2, na.rm = T), xlim=c(-1,1), main="", xlab="Log2(Mcov/Fcov)", cex.axis=1.5, cex.lab=1.5)

print(density(log(cov$Mcov/cov$Fcov, base=2), adjust=2, na.rm = T))

#--> find the maximum peak in the density distribution, and its corresponding x (xmax, which should correspond to the median Mcov/Fcov for the autosomes)

bubba <- data.frame(first=(density(log(cov$Mcov/cov$Fcov, base=2), adjust=2, na.rm = T)$x), second=(density(log(cov$Mcov/cov$Fcov, base=2), adjust=2, na.rm = T)$y))

xmax<-(subset(bubba, second==max(density(log(cov$Mcov/cov$Fcov, base=2), adjust=2, na.rm = T)$y))$first)

abline(v=xmax, lwd=2)

#--> that gives us the x-coordinate for our main peak

#so we can calculate the x-coordinate for our minor (X) peak:

xcoord<-(xmax+1)

abline(v=(xcoord-0.1), col="red", lty=2)

abline(v=(xcoord+0.1), col="red", lty=2)

#(or +1 for ZW)

xcandidates=subset(cov, (log(cov$Mcov/cov$Fcov, base=2))>((xcoord)-0.1) & (log(cov$Mcov/cov$Fcov, base=2))<((xcoord)+0.1))

#first we put all these values in a big data frame

chix<-aggregate(data.frame(count = xcandidates$chrom), list(value = xcandidates$chrom), length)

chiall<-aggregate(data.frame(count = cov$chrom), list(value = cov$chrom), length)

chi<-merge(chix, chiall, by="value", all=T)

chi[is.na(chi)] <- 0

#then we make the columns that we need to test each chromosome: x, a, controlx (how many are on X for other arms), controla (how many are on A for other chromosomes)

chi$x<-chi$count.x

chi$a<-(chi$count.y-chi$x)

chi$controlx<-(sum(chi$x)-chi$x)

chi$controla<-(sum(chi$a)-chi$a)

#then we get pvalues and plot barplot

#chi$pval<-chisq.test(matrix(c(chi$x, chi$a, chi$controlx, chi$controla),ncol=2), correct=T)$p.value

chrom2L<-fisher.test(rbind(c(subset(chi, value=="2L")$x, subset(chi, value=="2L")$a), c(subset(chi, value=="2L")$controlx, subset(chi, value=="2L")$controla)))$p.value

chrom2R<-fisher.test(rbind(c(subset(chi, value=="2R")$x, subset(chi, value=="2R")$a), c(subset(chi, value=="2R")$controlx, subset(chi, value=="2R")$controla)))$p.value

chrom3L<-fisher.test(rbind(c(subset(chi, value=="3L")$x, subset(chi, value=="3L")$a), c(subset(chi, value=="3L")$controlx, subset(chi, value=="3L")$controla)))$p.value

chrom3R<-fisher.test(rbind(c(subset(chi, value=="3R")$x, subset(chi, value=="3R")$a), c(subset(chi, value=="3R")$controlx, subset(chi, value=="3R")$controla)))$p.value

chrom4<-fisher.test(rbind(c(subset(chi, value=="4")$x, subset(chi, value=="4")$a), c(subset(chi, value=="4")$controlx, subset(chi, value=="4")$controla)))$p.value

chromX<-fisher.test(rbind(c(subset(chi, value=="X")$x, subset(chi, value=="X")$a), c(subset(chi, value=="X")$controlx, subset(chi, value=="X")$controla)))$p.value

pvals<-c(chrom2L, chrom2R, chrom3L, chrom3R, chrom4, chromX)

chi$pval<-pvals

chi$Xpect<-(chi$x/(((chi$controlx+chi$x)/(chi$x+chi$a+chi$controlx+chi$controla))*(chi$count.y)))

chi$col<-4

chi$col[chi$pval<0.01 & chi$Xpect>1] <- 2

#order by element before plotting

chi$Muller<-c("B", "C", "D", "E", "F", "A")

chi2<-chi[order(chi$Muller),]

print(chi2)

barplot(chi2$Xpect, names=c("A", "B", "C", "D", "E", "F"), col=chi2$col, ylab="Observed/Expected X-linked Scaffolds", cex.axis=1.5, cex.lab=1.5)

abline(h=1, lty=2)

2. Code for *Mayetiola destructor* (majority of the genome is X-linked)

#first, let's read the coverage table, plot the Mcov/Fcov density

cov2<-read.table("/Users/Beatriz/Documents/dosage_comp/2014Sept_Revisions1_PlosBio/NewCoverage/CovFiles/Mayetiola_FMcov_location.txt", head=T)

cov<-subset(cov2, chrom!="U")

cov1<-(subset(cov, cov$Mcov/cov$Fcov>1.5))

dev.new(width=15, height=8)

par(mfrow=c(1,2))

plot(density(log(cov$Mcov/cov$Fcov, base=2), adjust=2, na.rm = T), xlim=c(-1,2), main="", xlab="Log2(Mcov/Fcov)", cex.axis=1.5, cex.lab=1.5)

print(density(cov1$Mcov/cov1$Fcov, adjust=2, na.rm = T))

#--> find the maximum peak in the density distribution, and its corresponding x (xmax, which should correspond to the median Mcov/Fcov for the autosomes)

bubba <- data.frame(first=(density(log(cov1$Mcov/cov1$Fcov, base=2), adjust=2, na.rm = T)$x), second=(density(log(cov1$Mcov/cov1$Fcov, base=2), adjust=2, na.rm = T)$y))

xmax<-(subset(bubba, second==max(density(log(cov1$Mcov/cov1$Fcov, base=2), adjust=2, na.rm = T)$y))$first)

abline(v=xmax, lwd=2)

#--> that gives us the x-coordinate for our main peak

#so we can calculate the x-coordinate for our minor (X) peak:

xcoord<-(xmax-1)

abline(v=(xcoord-0.1), col="red", lty=2)

abline(v=(xcoord+0.1), col="red", lty=2)

#(or -1 for ZW)

xcandidates=subset(cov, (log(cov$Mcov/cov$Fcov, base=2))>((xcoord)-0.1) & (log(cov$Mcov/cov$Fcov, base=2))<((xcoord)+0.1))

#first we put all these values in a big data frame

chix<-aggregate(data.frame(count = xcandidates$chrom), list(value = xcandidates$chrom), length)

chiall<-aggregate(data.frame(count = cov$chrom), list(value = cov$chrom), length)

chi<-merge(chix, chiall, by="value", all=T)

chi[is.na(chi)] <- 0

#then we make the columns that we need to test each chromosome: x, a, controlx (how many are on X for other arms), controla (how many are on A for other chromosomes)

chi$x<-chi$count.x

chi$a<-(chi$count.y-chi$x)

chi$controlx<-(sum(chi$x)-chi$x)

chi$controla<-(sum(chi$a)-chi$a)

#then we get pvalues and plot barplot

#chi$pval<-chisq.test(matrix(c(chi$x, chi$a, chi$controlx, chi$controla),ncol=2), correct=T)$p.value

chrom2L<-fisher.test(rbind(c(subset(chi, value=="2L")$x, subset(chi, value=="2L")$a), c(subset(chi, value=="2L")$controlx, subset(chi, value=="2L")$controla)))$p.value

chrom2R<-fisher.test(rbind(c(subset(chi, value=="2R")$x, subset(chi, value=="2R")$a), c(subset(chi, value=="2R")$controlx, subset(chi, value=="2R")$controla)))$p.value

chrom3L<-fisher.test(rbind(c(subset(chi, value=="3L")$x, subset(chi, value=="3L")$a), c(subset(chi, value=="3L")$controlx, subset(chi, value=="3L")$controla)))$p.value

chrom3R<-fisher.test(rbind(c(subset(chi, value=="3R")$x, subset(chi, value=="3R")$a), c(subset(chi, value=="3R")$controlx, subset(chi, value=="3R")$controla)))$p.value

chrom4<-fisher.test(rbind(c(subset(chi, value=="4")$x, subset(chi, value=="4")$a), c(subset(chi, value=="4")$controlx, subset(chi, value=="4")$controla)))$p.value

chromX<-fisher.test(rbind(c(subset(chi, value=="X")$x, subset(chi, value=="X")$a), c(subset(chi, value=="X")$controlx, subset(chi, value=="X")$controla)))$p.value

pvals<-c(chrom2L, chrom2R, chrom3L, chrom3R, chrom4, chromX)

chi$pval<-pvals

chi$Xpect<-(chi$x/(((chi$controlx+chi$x)/(chi$x+chi$a+chi$controlx+chi$controla))*(chi$count.y)))

chi$col<-4

chi$col[chi$pval<0.01 & chi$Xpect>1] <- 2

#order by element before plotting

chi$Muller<-c("B", "C", "D", "E", "F", "A")

chi2<-chi[order(chi$Muller),]

print(chi2)

barplot(chi2$Xpect, names=c("A", "B", "C", "D", "E", "F"), col=chi2$col, ylab="Observed/Expected X-linked Scaffolds", cex.axis=1.5, cex.lab=1.5)

abline(h=1, lty=2)
